# Novel Isatin–Chalcone Hybrid Molecules: Design, Synthesis and Anti-Neuroinflammatory Activity Evaluation

**DOI:** 10.3390/molecules30071421

**Published:** 2025-03-22

**Authors:** Rongrong Wang, Zhili Zhang, Wei Jiang, Junyi Liu, Chao Tian, Meng Wang

**Affiliations:** 1 College of Pharmacy, Beihua University, Jilin 132013, China; rongrwang@126.com (R.W.); jiangjwei@126.com (W.J.); 2 Department of Chemical Biology, School of Pharmaceutical Sciences, Peking University, Beijing 100191, China; lilybmu@bjmu.edu.cn (Z.Z.); jyliu@bjmu.edu.cn (J.L.); 3 State Key Laboratory of Natural and Biomimetic Drugs, Peking University, Beijing 100191, China

**Keywords:** anti-neuroinflammatory, isatin, nitric oxide, TNF-α, IL-6

## Abstract

Neuroinflammation is considered a significant factor in triggering numerous neurodegenerative diseases. Hence, the development of effective anti-inflammatory drugs is of utmost urgency. In this study, three series of new isatin–chalcone hybrid derivatives were successfully designed and synthesized, and their anti-neuritis activities were explored using BV2 microglial cells. The results indicated that compound **4b** exhibited the most potent anti-inflammatory activity (IC_50_ = 1.6 μM; TI = 21.6). After being treated with compound **4b**, the production of TNF-α and IL-6 decreased significantly (*p* < 0.0001). *In silico* molecular modeling studies on inflammation proteins suggested that compound **4b** might bind to TLR4/MD2 and p38. Predicted by the software Molinspiration, the Log *p* value and Log BB of compound **4b** were 3.36 and −0.32, respectively.

## 1. Introduction

With the aging of the global population, the incidence of neurodegenerative diseases has shown a continuously rising trend [1]. Although the pathogenic mechanisms of neurodegenerative diseases are still unclear at present, more and more experiments have shown that neuroinflammation plays an important role in the occurrence and development of many neurodegenerative diseases, such as Alzheimer and Parkinson disease, Huntington’s chorea, etc. [2,3]. Neuroinflammation is an immune response activated by microglial cells and astrocyte cells under the stimulation of infection, toxic metabolites, trauma or autoimmunity [4,5]. It can cause damage to and inhibit neurons and nerve regeneration [6]. Therefore, there is an urgent need for a safe and effective preparation for anti-neuroinflammation.

**Figure 1:**
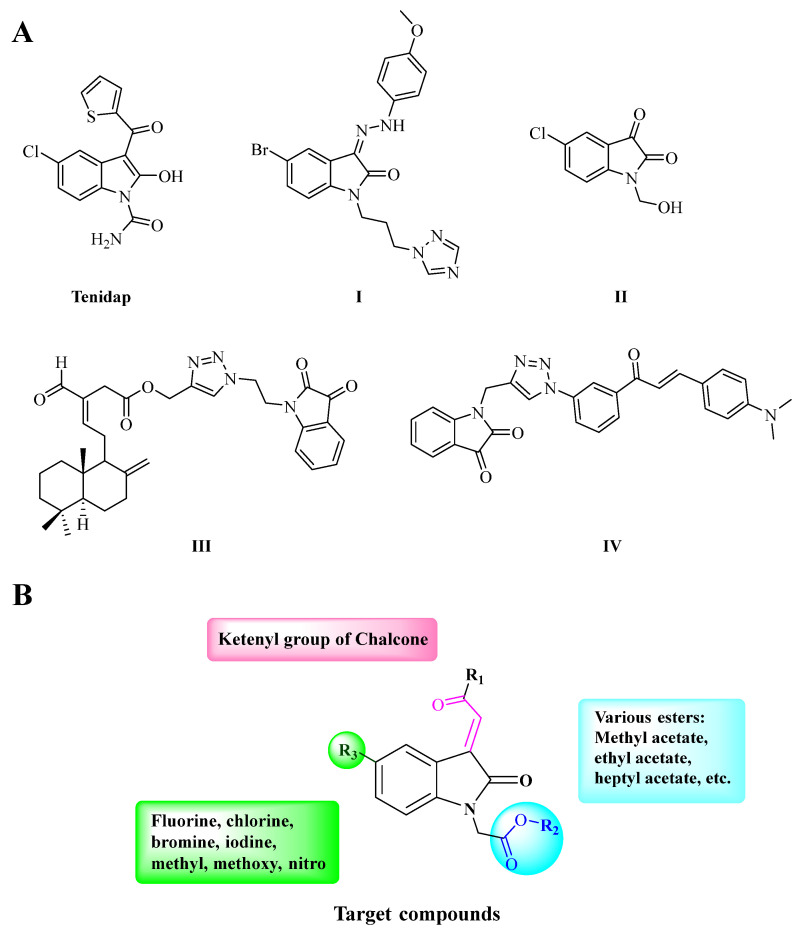
(**A**) Structures of Tenidap and isatin derivatives **I**–**IV** with anti-inflammatory activities; (**B**) the structures of target compounds.

Isatin (1*H*-indole-2,3-dione) is an endogenous heterocyclic compound existing in a variety of plants. Due to its numerous pharmacological activities, it is used as a precursor in many studies and plays a crucial role in medicinal chemistry [7,8]. Currently, a variety of anti-inflammatory compounds with isatin as the basic structure have been presented [6,8,9,10,11] (Figure 1A). In addition, chalcone is a type of secondary metabolite with the general structure of 1,3-diphenyl-2-propen-1-one [12]. These precursors of the flavonoid biosynthesis pathway are usually present in plants, fruits, vegetables and spices and have various biological activities, such as anti-inflammatory, anti-cancer and antibacterial effects [13].

Taking into consideration the aforementioned information, we designed and synthesized a series of new *N*^1^-(2-oxoethyl)isatin–chalcone hybrid compounds, (Figure 1B) envisaging them as having good anti-neuroinflammation activities and low cytotoxicities. To study the impact of electronic effects of istatin on anti-neuroinflammatory activity, electron-withdrawing and electron-donating substituents (e.g., alkyl, methoxy, halogen and nitro) were introduced to its 5-position. Because of the importance of solubility and membrane permeability in neuroprotectant research, lipophilicity ester groups were optimized for physicochemical balance: long or benzene-substituted aliphatic chains to enhance membrane permeability and biological activity. The anti-neuroinflammation activities of target compounds were evaluated using lipopolysaccharide (LPS)-activated BV2 microglia cells as a cellular model of murine neuroinflammation [14,15,16]. Furthermore, the plausible targets of these compounds were explored using molecular modeling studies.

## 2. Results and Discussion

### 2.1. Chemistry

**Scheme 1:**
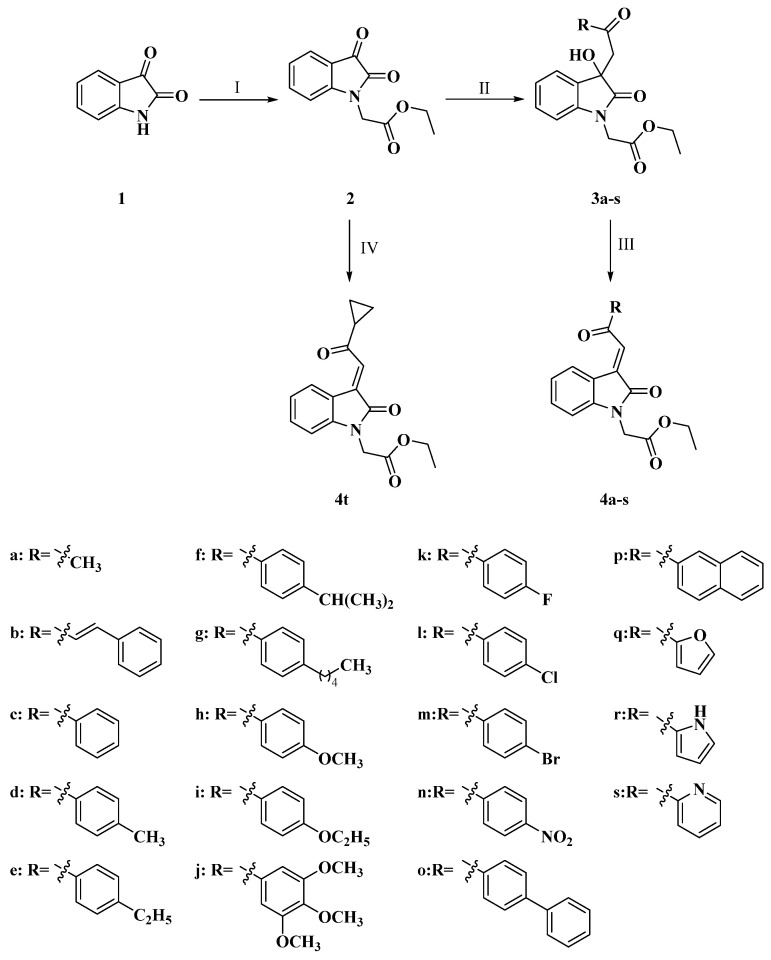
Synthetic route of compounds **4a**–**t**. Reagents and conditions: (I) ethyl bromoacetate, K_2_CO_3_, DMF, 85 °C, 63%; (II) corresponding ketones, K_2_CO_3_ or Et_2_NH, EtOH, 60 °C or r.t., 16–96%; (III) HCl (aq, conc), EtOH, reflux, 29–89%; (IV) (1) 1-cyclopropyl-2-bromoacetone, Ph_3_P, THF, reflux, 90%; (2) CH_2_Cl_2_, 1 N NaOH, r.t., 97%; (3) compound **2**, THF, 60 °C, 57%.

The synthetic routes to compounds **4a**–**t** were presented in Scheme 1. The ethyl 2-(2,3-dioxoindolin-1-yl) acetate prepared using a procedure reported by Inzaman Abbasi. Isatin was reacted with ethyl bromoacetate in DMF in the presence of K_2_CO_3_ at 85 °C for 3 h to afford compound **2** [17]. The base-catalyzed aldol reaction of compound **2** with acetone or substituted methyl ketones yielded the corresponding “tertiary alcohols” **3a**–**s**, which have not been reported in the literature before. Unfortunately, the presence of the *N*^1^-(2-oxoethyl) moiety of isatin resulted in yields lower than those previously reported similar reactions [18]. Then, the intermediates **3a**–**s** were converted into the corresponding isatin–chalcone hybrid compounds **4a**–**s** by acidic dehydration with HCl [19]. However, during the transformation, side reactions like ester bond hydrolysis and rearrangement presumably consumed some intermediates, impacting the overall yield of target compounds, especially compounds **4a**, **4q** and **4r** [20]. In the synthesis of the cyclopropane-containing compound **4t**, the Wittig reaction was employed to prevent the cleavage of the cyclopropyl ring. The key intermediate, (2-cyclopropyl-2-oxoethyl)triphenylphosphonium bromide, was efficiently afforded via the reaction of 1-cyclopropyl-2-bromoacetone with triphenylphosphine under reflux in THF. Subsequently, the treatment of this intermediate with sodium hydroxide led to the formation of 1-cyclopropyl-2-(triphenylphosphoranylidene)ethanone. The reaction of compound **2** with 1-cyclopropyl-2-(triphenylphosphoranylidene)ethanone culminated in the successful synthesis of compound **4t**, achieving a yield of 50% [21].

**Scheme 2:**
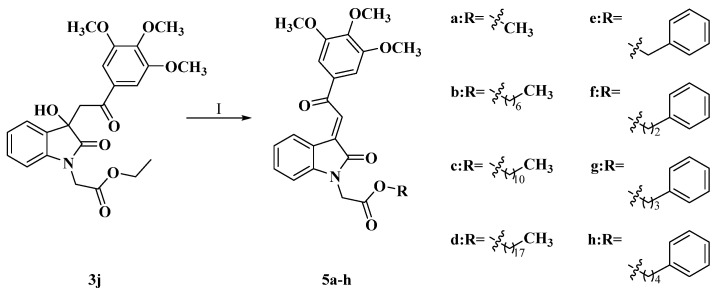
Synthetic route of compounds **5a**–**h**. Reagents and conditions: (I) HCl (aq, conc), corresponding alcohols, reflux, 21–77%.

Considering polymethoxy substituted benzene as a special moiety in natural products with anti-inflammatory activity such as erianin [22], colchicine [23] and schisandrine [24], we designed 3,4,5-trimethoxy compounds **5a**–**h** with aliphatic or benzene-substituted ester groups. They were synthesized through a “one pot” method including transesterification and dehydration elimination, as shown in Scheme 2. With the key intermediate **3j** prepared according to Scheme 1 in hand, transesterification was performed with the corresponding alcohols at reflux under the catalysis of HCl. Upon completion of the reaction, compounds **5a**–**d** with aliphatic chains were obtained with simple workup, whereas compounds **5e**–**h** with benzene-substituted aliphatic chains were purified through column chromatography.

**Scheme 3:**
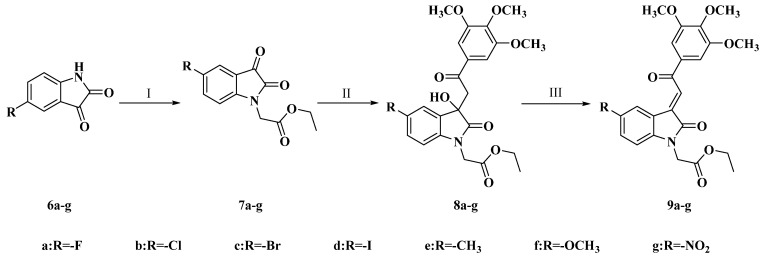
Synthetic route of compounds **9a**–**g**. Reagents and conditions: (I) ethyl bromoacetate, K_2_CO_3_, DMF, 85 °C, 58–79%; (II) 3,4,5-Trimethoxyacetophenone, Et_2_NH, EtOH, r.t., 29–73%; (III) HCl (aq, conc), EtOH, reflux, 57–75%.

Isatin–chalcone hybrid compounds **9a**–**g**, with different substituents at position 5, were synthesized using procedures as shown in Scheme 3. 5-Substituted isatin **6a**–**g** reacted with ethyl bromoacetate, then with 3,4,5-trimethoxyacetophenone. Following an elimination–dehydration reaction, compounds **9a**–**g** were obtained similar to Scheme 1.

### 2.2. Biological Activity

As a small-molecule messenger molecule, the continuous and appropriate production of nitric oxide (NO) is essential for maintaining normal physiological functions [13,25]. However, the massive production of NO may cause toxic reactions to cells. For example, in the state of infection or immune stress, inducible nitric oxide synthase (iNOS) is highly activated, producing excessive amounts of NO [26]. NO can interact with reactive oxygen species (ROS) to form substances with stronger oxidative activity, further damaging cells and tissues [27].

**Figure 2:**
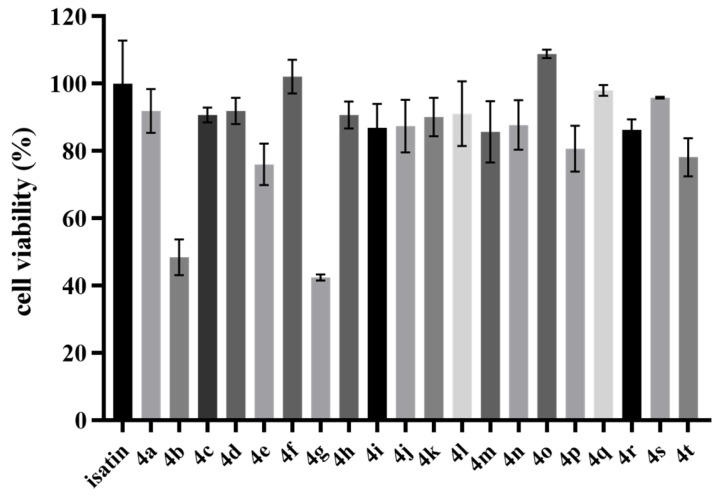
The influence of compounds **4a**–**t** on the viability of BV2 cells at 25 μM. In the positive control group, 25 μM isatin was added. Data are presented as mean values. Error bars denote the standard error of at least three independent experiments performed in triplicate.

**Figure 3:**
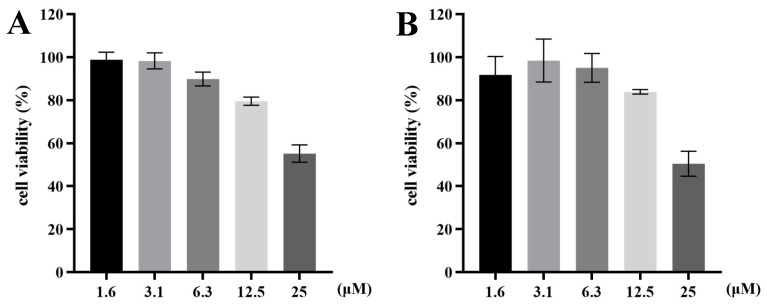
(**A**) The effect of compound **4b** on the viability of BV2 cells at different concentrations; (**B**) the effect of compound **4g** on the viability of BV2 cells at different concentrations. Data are presented as mean values. Error bars denote the standard error of at least three independent experiments performed in triplicate.

Lipopolysaccharide (LPS) can induce inflammatory responses through multiple mechanisms. The cell model established with it can effectively simulate the pathogenic mechanism of inflammation [28]. Prior to evaluating their anti-inflammatory activities, the cytotoxicities of all compounds against BV2 cells were assessed using the MTS assay, with isatin as a reference compound [29]. The results showed that at 25 μM, the cell viabilities of most compounds **4a**–**t** were greater than 90% (Figure 2). Among them, compounds **4f**, **4o**, **4q** and **4s** were nearly non-cytotoxic to BV2 microglia cells. However, compound **4b** containing *(E)*-1-phenylpenta-1,4-dien-3-one and compound **4g** containing 1-(4-pentylphenyl)prop-2-en-1-one reduced cell viability to 48% and 42%, respectively. To explore the safe concentrations of compounds **4b** and **4g**, we measured the viability of BV2 cells at concentrations of 12.5 μM, 6.3 μM and 3.1 μM. It turned out that regarding compound **4b**, when its concentration was 12.5 μM, the cell viability was 79.5%. As the concentration decreased to 6.23 μM, the cell survival rate increased to 89.9%. When it further dropped to 3.1 μM, the cell survival rate reached 98.3%. Similarly, for compound **4g**, at 12.5 μM, 6.3 μM and 3.1 μM, the cell viabilities were 83.9%, 95.1% and 98.4%, respectively (Figure 3). The result shows that, under the concentration of 12.5 μM, **4b** and **4g** did not show obvious cytotoxicity.

BV2 cells stimulated by lipopolysaccharide (LPS) lead to an increase in NO release. Therefore, we used the inhibitory ability of the target compounds on NO as a preliminary method to evaluate their anti-neuroinflammatory properties. We evaluated the inhibitory ability of isatin–chalcone hybrid derivatives **4a**–**t** on the release of NO from BV2 cells at 20 μM. The release amount of NO was detected by the Griess method, and the actual release amount of NO was calculated based on the experimental data [30].

**Figure 4:**
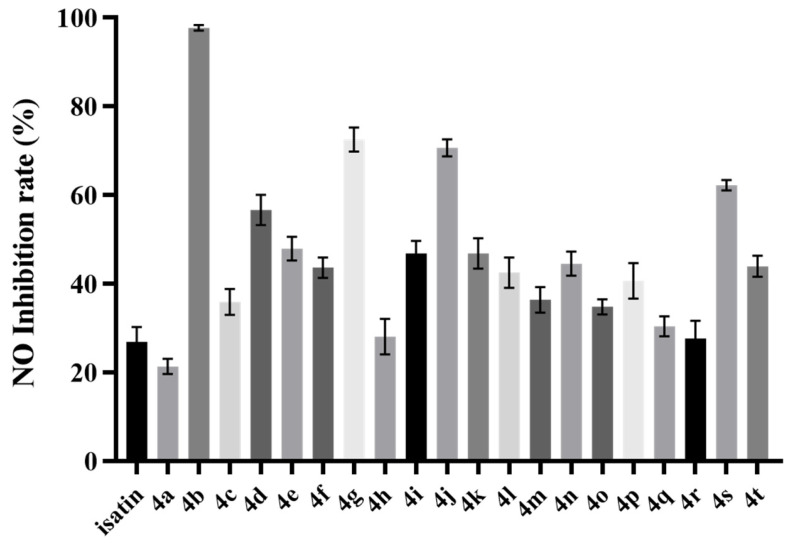
The inhibition rate of **4a**–**t** compounds on the production of NO by BV2 cells at 20 μM. Isatin was used as the control group and acted on the cells at 20 μM. Data are presented as mean values. Error bars denote the standard error of at least three independent experiments performed in triplicate.

As shown in Table 1, most compounds of **4a**–**t** had a moderate inhibitory effect on the release of NO at 20 µM. Compound **4a** is a compound with a methyl group as the *R*_1_-substituent on the ketenyl group of chalcone. Its NO release amount was 22.9 μM, and it was the least efficient in reducing NO release. Meanwhile, furan-substituted derivative **4q** and pyrrole-substituted **4r** could only inhibit 30% and 28% of NO release, respectively (Figure 4). It indicates that when the *R*_1_ substituent on the ketenyl group of chalcone moiety is a small group, it is not conducive to the exertion of anti-neuroinflammatory activity. Comparatively, compounds **4b**, **4g** and **4j** had good inhibitory capabilities, being able to reduce NO release by 98%, 73% and 71%, respectively.

To avoid the effect of activity induced by the cytotoxicity, therapeutic indexes of compounds **4b**, **4g** and **4j** were determined. The IC_50_s for NO release of **4b**, **4g** and **4j** were 1.6 μM, 10.8 μM and 15.7 μM, and the therapeutic indexes were 21.6, 2.8 and 4.6, respectively (Table 2). The above results suggest that compound **4b** is an anti-inflammatory drug with low toxicity and high efficiency.

**Figure 5:**
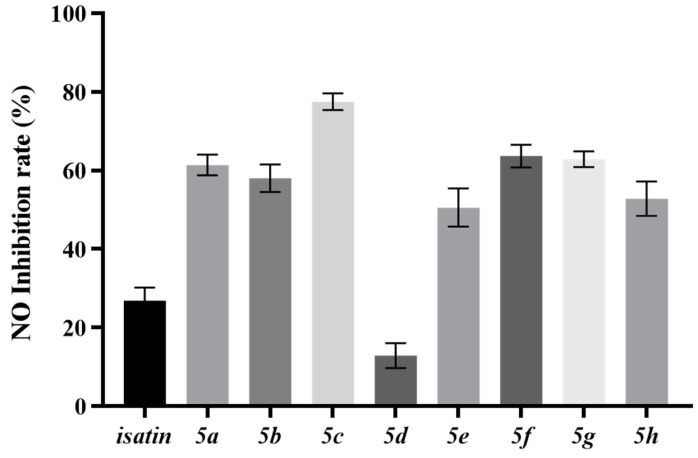
The inhibition rate of **5a**–**h** compounds on the production of NO by BV2 cells at 20 μM. Isatin was used as the control group and acted on the cells at 20 μM. Data are presented as mean values. Error bars denote the standard error of at least three independent experiments performed in triplicate.

As shown in Figure 5, compounds **5a**–**g** with aliphatic or benzene-substituted ester derivatives at *N*^1^ position demonstrated relatively strong NO-inhibiting abilities at 20 μM. Besides **5g**, all compounds exhibited potent inhibitory activities against NO release by 61%, 58%, 77%, 64% and 63%, respectively. Meanwhile, the IC_50_s of compounds **5a**, **5c**, **5f** and **5g** were 10.9 μM, 6.3 μM, 10.7 μM and 6.0 μM, respectively (Table 3). Phenylpropyl ester **5g** had a higher activity (6.0 μM) and therapeutic index (8.1) than ethyl ester compound **4j** (15.7 μM and 4.6, respectively). The modification of ester moiety can improve the inhibitory activity against NO release.

**Figure 6:**
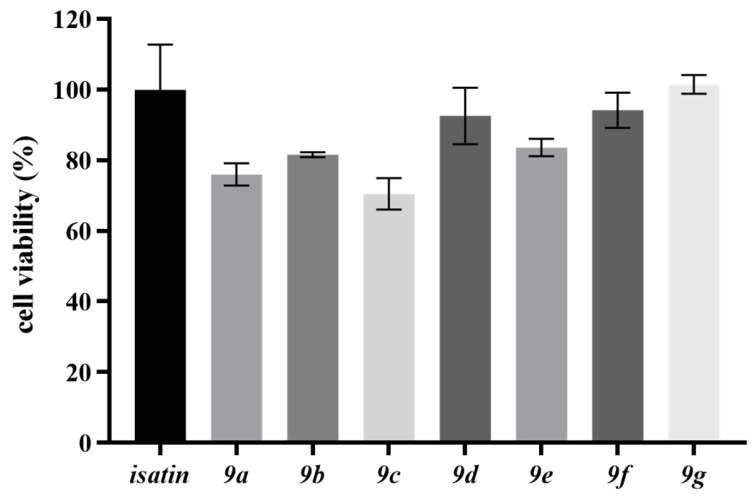
The influence of **9a**–**g** compounds at 25 μM on the viability of BV2 cells. In the positive control group, 25 μM isatin was added. Data are presented as mean values. Error bars denote the standard error of at least three independent experiments performed in triplicate.

As shown in Figure 6, compounds **9a**–**g** with different substituents at the 5-position of isatin exhibited good cell viability at 25 μM. The NO release inhibiting the experiment results indicated that compound **9d** with an iodine atom at the 5-position of isatin had the weakest ability to inhibit NO release, with an inhibition rate of only 22% at 20 μM. Besides **9d**, halogen-substituted derivatives **9a**–**c** also exhibited lower inhibitory ability than other compounds.

**Figure 7:**
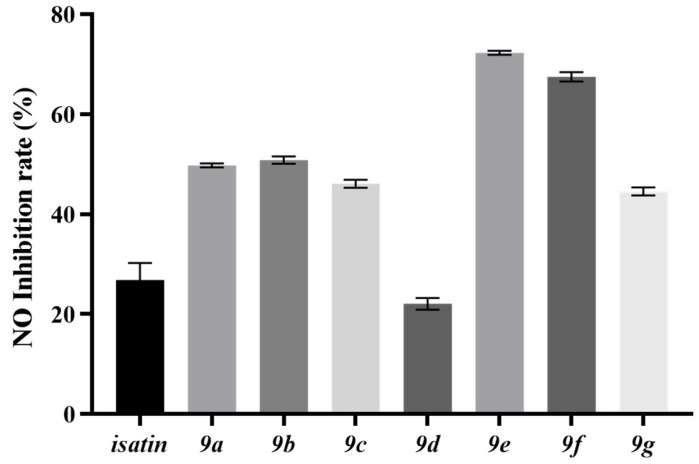
The inhibition rate of **9a**–**g** compounds on the production of NO by BV2 cells at 20 μM. Isatin was used as the control group and acted on the cells at 20 μM. Data are presented as mean values. Error bars denote the standard error of at least three independent experiments performed in triplicate.

The NO inhibition rates of compound **9e** and **9f**, with electron donor methyl and methoxyl group at the 5-position, respectively, were 72% and 68% at 20 μM, which were higher than that of compound **4j** without substituent (Figure 7). Meanwhile, the IC_50_s of compounds **9e** and **9f** were 10.3 μM and 10.4 μM, respectively, and their therapeutic indexes were higher than that of compound **4j** (TI = 4.6), being 7.8 and 7.5, respectively (Table 4). The result indicated that the introduction of an electron donor group in the 5-position of isatin benefits the activity of NO inhibition.

**Figure 8:**
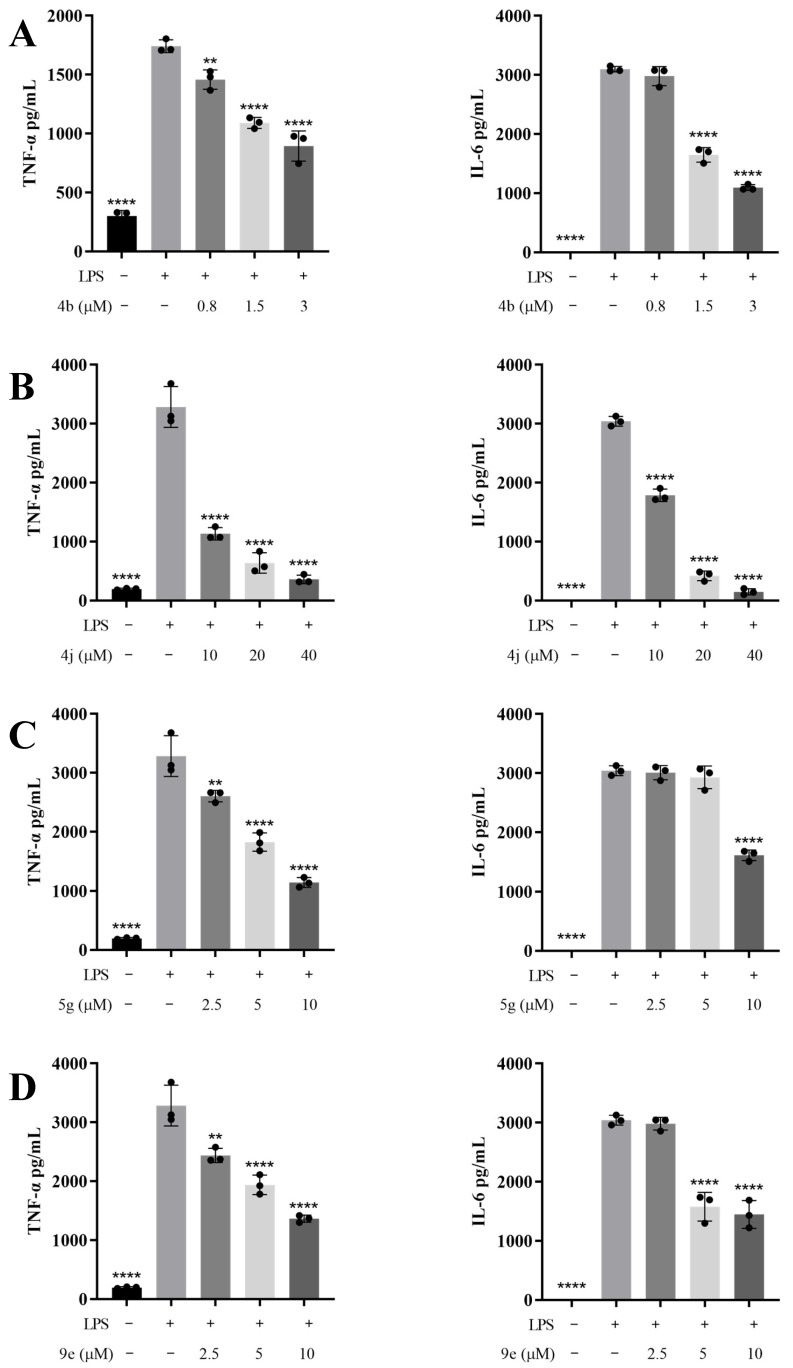
Effect of **4b** (**A**), **4j** (**B**), **5g** (**C**) and **9e** (**D**) on release of cytokine TNF-α and IL-6 by LPS stimulated BV2 cells. Error bars denote the standard error of at least three independent experiments performed in triplicate. One-way analysis of variance (ANOVA), compared to LPS, ** *p* < 0.01, **** *p* < 0.0001.

The cytokines TNF-α and IL-6 serve as powerful mediators of cellular communication and play essential roles in the regulation of innate and adaptive inflammatory responses [31]. Therefore, the abilities of isatin derivatives **4b**, **4j**, **5g** and **9e** reducing the production of proinflammatory mediators TNF-α and IL-6 were further evaluated (Figure 8).

The results of **4b**, **4j**, **5g** and **9e** showed that the LPS activation of BV2 cells led to a significant increase in the production of TNF-α and IL-6 compared with unstimulated cells, which is in agreement with recent results reported for LPS-activated BV2 cells [8]. All four compounds significantly reduced the levels of TNF-α and IL-6 and exhibited an obvious dose-dependent relationship. Among them, compound **4b** at a concentration of 1.5 μM demonstrated the best inhibitory activities against inflammatory factors, reducing TNF-α levels by 37% and IL-6 levels by 47% (*p* < 0.0001). In contrast, compounds **4j**, **5g** and **9e** exhibited relatively weaker inhibitory effects, which were only capable of exerting significant inhibition at the relatively high concentration of 5 μM or 10 μM.

### 2.3. Molecular Docking

We searched for a total of 20 proteins that are related to anti-inflammatory activity. Most of them play important roles in the inflammatory signaling pathways, such as the Toll-like receptor (TLR) pathway, mitogen-activated protein kinase (MAPK) pathway and cyclooxygenase (COX) pathway. After obtaining crystal structures of these candidate proteins from the RCSB Protein Data Bank (PDB), we conducted molecular docking experiments with compound **4b**. The molecular docking results of compound **4b** and the 20 proteins have been shown in Table 5. Among these 20 proteins, TLR4/MD2, p38, MD2, COX-2, COX-1, JNK3 and JNK1 showed high absolute values of docking scores, ranging from 7.26 to 10.36 (Table 5). Among them, TLR4/MD2, p38 and MD2 demonstrated the highest binding affinity, with docking scores of −10.36, −9.49 and −9.45 kcal/mol, respectively.

TLR4 is a key receptor in the innate immune system that plays a crucial role in recognizing pathogen-associated molecular patterns and initiating the inflammatory response [32]. MD2 is an accessory protein of TLR4 that is essential for the recognition of lipopolysaccharide (LPS) by TLR4 [33]. Compound **4b** could be well docked into the pocket between TLR4 and MD2. Specifically, the chalcone benzene ring of compound **4b** formed a π–π interaction with the residue PHE104 of MD2 (Figure S1A). The results suggested that compound **4b** may bind to the TLR4/MD2 complex via the protein MD2, thereby exerting an anti-inflammatory effect.

**Figure 9:**
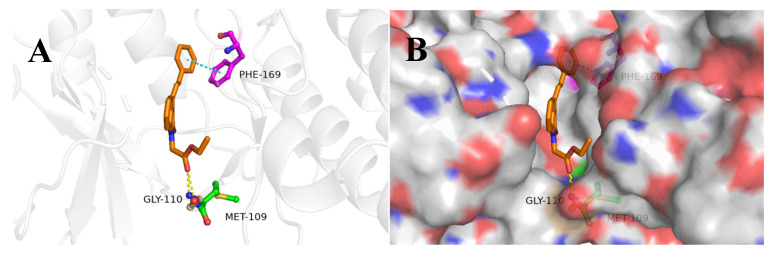
Three-dimensional visual representation of the complex of **4b** and p38 (PDB ID: 6OHD). (**A**) The 3D docking conformation of the ball-and-stick model of **4b** and p38. (**B**) The 3D conformation of the surface model of **4b** and p38.

P38 is a member of the MAPK family, which is involved in various cellular processes, including inflammation, cell proliferation and apoptosis [34]. The chalcone benzene ring of compound **4b** formed a π–π interaction with residue PHE169 of p38. Additionally, the oxygen atom of the carbonyl group formed hydrogen bonds with GLY110 and MET109, respectively (Figure 9A). The combination of these two types of interactions may block the activation of p38 kinase, prevent the phosphorylation of downstream substrates and ultimately inhibit the inflammatory response.

The molecular docking results of compound **4b** with inflammation proteins MD2 and p38 suggested the possible target, which will be confirmed further through *in vitro* and *in vivo* studies.

### 2.4. Prediction of Blood–Brain Barrier Permeability

The blood–brain barrier (BBB) is composed of the multicellular neurovascular unit (NVU), which can regulate the entry of substances from the blood into the brain and plays a role in some neurological diseases [35,36]. According to the rules of Hitchcock and Pennington, it was recommended that the Log *p* threshold be distributed between 2 and 5 [35]. Predicted by the website of Molinspiration (https://www.molinspiration.com/) accessed on 8 July 2024. The Log *p* values of most compounds were between 2 and 5. Among them, the Log *p* value of compound **4b** was 3.36. Subsequently, its Log BB was calculated to be −0.32. Since this value was greater than −1 [35], it indicated that compound **4b** had the ability to penetrate the blood–brain barrier.

## 3. Conclusions

To find more neuroprotective agents, 35 new compounds based on isatin–chalcone hybrid were designed and synthesized. They all exhibited inhibition activities against NO release. The structure–activity relationship on substituent of chalcone moiety, aliphatic or benzene-substituted ester moiety and the 5-position different substituents has been discussed. Compound **4b** can be considered as a lead compound or candidate in the treatment of neurodegenerative diseases because of its remarkable NO-inhibitory activity, potent inhibition against the release of TNF-α and IL-6 and possibly good permeability through the blood–brain barrier.

## 4. Methods

### 4.1. Materials and Methods

All chemicals used in this study were of reagent grade and were purchased from commercial suppliers and used as received without further purification. Solvents, reagents and isatins were purchased from Energy Chemical (Shanghai, China), Macklin (Shanghai, China), Beijing Chemical Plant (Beijing, China), Quanrui Reagent Co., Ltd., (Jinzhou, China) and Tongguang Fine Chemicals Company (Beijing, China). Thin-layer chromatography (TLC) analysis was performed using TLC silica gel 60 F_254_ 25 aluminum sheets 20 × 20 cm (supelco, Darmstadt, Germany), and column chromatography analysis was performed using 200–300 mesh silica gel (Qingdao Marine Chemical Co., Ltd., Qingdao, China). The ^1^H NMR and ^13^C NMR spectra were obtained on a Bruker AVANCE III-400 MHz instrument (Karlsruhe, Germany). The solvents were CDCl_3_ and deuterated DMSO, and the internal standard was TMS; all deuterated reagents were purchased from Energy Chemical (Shanghai, China). The high-resolution mass spectrometry was obtained on a Thermo Q Exactive HF-X (Thermo Fisher Scientific, MA, USA). An X_4_ microscopic melting point apparatus was used as the melting point instrument. It was purchased from Shanghai Precision Instrument Science Co., Ltd. (Shanghai, China). The thermometer was not corrected.

#### 4.1.1. Method for the Synthesis of Intermediate ethyl 2-(2,3-dioxoindolin-1-yl)acetate (**2**)

To a solution of isatin (3 g, 20.39 mmol) in DMF (1.5 mL), K_2_CO_3_ (3.4 g, 1.2 eq) and ethyl bromoacetate (2.7 mL, 1.2 eq) were added. The reaction mixture was stirred at 85 °C for 3 h until the complete disappearance of isatin was evidenced by TLC. The reaction mixture was cooled to room temperature, then poured into water. The resulting solid was filtered, washed with EtOH and dried, and these crude products were used directly in the next step. Yellow powder, 63% yield, m.p. 127.5–128.4 °C (lit. mp: 128–130 °C [17]). ^1^H-NMR was in agreement with those reported in the literature [17]. ^1^H NMR (400 MHz, CDCl_3_) δ 7.64 (d, *J* = 7.5 Hz, 1H, Ar-H^7^), 7.60 (td, *J* = 7.8, 1.2 Hz, 1H, Ar-H^5^), 7.16 (t, *J* = 7.5 Hz, 1H, Ar-H^6^), 6.81 (d, *J* = 7.9 Hz, 1H, Ar-H^4^), 4.50 (s, 2H, N-CH_2_-CO), 4.25 (q, *J* = 7.1 Hz, 2H, OCH_2_), 1.29 (t, *J* = 7.1 Hz, 3H, CH_3_). ^13^C NMR (101 MHz, CDCl_3_) δ 182.5, 166.8, 158.1, 150.3, 138.5, 125.6, 124.2, 117.6, 110.2, 62.2, 41.3, 14.1.

#### 4.1.2. Method for the Synthesis of Intermediate Ethyl 2-(3-hydroxy-2-oxo-3-(2-oxopropyl)indolin-1-yl)acetate (**3a**)

A mixture of ethyl 2-(2,3-dioxoindolin-1-yl)acetate (500 mg, 2.14 mmol) and K_2_CO_3_ (296 mg, 1 eq) in acetone (8 mL) was stirred at 60 °C for 3 h until the disappearance of compound **2** was evidenced by TLC. The solvent was removed under vacuum, and 5 mL of water was added to the residue and extracted with CH_2_Cl_2_ (3 × 10 mL). The organic fractions were combined, dried over Na_2_SO_4_, filtered and concentrated. The crude product was purified by column chromatography (silica gel, petroleum ether/ethyl acetate = 1:1). White powder, 36% yield, m.p. 96.3–97.9 °C. ^1^H NMR (400 MHz, CDCl_3_) δ 7.41 (dd, *J* = 7.4, 0.7 Hz, 1H, Ar-H^4^), 7.32 (td, *J* = 7.8, 1.2 Hz, 1H, Ar-H^6^), 7.10 (td, *J* = 7.6, 0.7 Hz, 1H, Ar-H^5^), 6.74 (d, *J* = 7.8 Hz, 1H, Ar-H^7^), 4.56 (d, *J* = 17.6 Hz, 1H, N-CH_2_-CO), 4.35 (d, *J* = 17.6 Hz, 1H, N-CH_2_-CO), 4.24 (q, *J* = 7.1 Hz, 2H, OCH_2_), 3.20 (d, *J* = 17.0 Hz, 1H, CO-CH_2_-C), 2.98 (d, *J* = 17.0 Hz, 1H, CO-CH_2_-C), 2.21 (s, 3H, COCH_3_), 1.29 (t, *J* = 7.1 Hz, 3H, CH_3_). ^13^C NMR (101 MHz, CDCl_3_) δ 208.0, 176.0, 167.5, 142.0, 130.0, 129.7, 124.2, 123.5, 108.6, 74.4, 61.9, 48.5, 41.4, 31.5, 14.1. HRMS *m*/*z*: 292.1170 [M + H]^+^, calcd for C_15_H_18_NO_5_: 292.1179.

#### 4.1.3. Method for the Synthesis of Intermediate **3b**–**s**

To a solution of ethyl 2-(2,3-dioxoindolin-1-yl)acetate (500 mg, 2.14 mmol) in EtOH (1 mL), Et_2_NH (265 μL, 1.2 eq) and the corresponding ketones (1.2 eq) were added. The reaction mixture was stirred at room temperature until the complete disappearance of compound **2** as evidenced by TLC. The solid crude products **3b**–**s** were filtered, washed with EtOH and dried, and these crude products were used directly in the next step.

##### Ethyl (*E*)-2-(3-hydroxy-2-oxo-3-(2-oxo-4-phenylbut-3-en-1-yl)indolin-1-yl)acetate (**3b**)

White powder, 55% yield, m.p. 90.7–91.2 °C. ^1^H NMR (400 MHz, CDCl_3_) δ 7.56 (d, *J* = 16.3 Hz, 1H, Ph-CH=), 7.53–7.48 (m, 2H, Ar-H^2′^, Ar-H^6′^), 7.44 (d, *J* = 7.4 Hz, 1H, Ar-H^4^), 7.42–7.35 (m, 3H, Ar-H^3′^, Ar-H^4′^, Ar-H^5′^), 7.29 (t, *J* = 7.8 Hz, 1H, Ar-H^6^), 7.07 (t, *J* = 7.5 Hz, 1H, Ar-H^5^), δ 6.73 (d, *J* = 7.9 Hz, 1H, Ar-H^7^), 6.70 (d, *J* = 16.2 Hz, 1H, CO-CH=CH), 4.93 (s, 1H, OH), 4.57 (d, *J* = 17.6 Hz, 1H, N-CH_2_-CO), 4.35 (d, *J* = 17.6 Hz, 1H, N-CH_2_-CO), 4.22 (q, *J* = 7.1 Hz, 2H, OCH_2_), 3.45 (d, *J* = 16.7 Hz, 1H, CO-CH_2_-C), 3.18 (d, *J* = 16.7 Hz, 1H, CO-CH_2_-C), 1.27 (t, *J* = 7.1 Hz, 3H, CH_3_). ^13^C NMR (101 MHz, CDCl_3_) δ 198.9, 176.1, 167.6, 144.8, 142.0, 134.0, 131.1, 129.9, 129.0, 128.6, 126.1, 124.4, 123.5, 108.6, 74.8, 61.9, 45.7, 41.4, 14.2. HRMS *m*/*z*: 380.1477 [M + H]^+^, calcd for C_22_H_22_NO_5_: 380.1492.

##### Ethyl 2-(3-hydroxy-2-oxo-3-(2-oxo-2-phenylethyl)indolin-1-yl)acetate (**3c**)

White powder, 83% yield, m.p. 162.8–163.4 °C. ^1^H NMR (400 MHz, CDCl_3_) δ 7.91 (d, *J* = 7.1 Hz, 2H, Ar-H^3′^, Ar-H^5′^), 7.58 (t, *J* = 7.4 Hz, 1H, Ar-H^4^), 7.49–7.41 (m, 3H, Ar-H^2′^, Ar-H^4′^, Ar-H^6′^), 7.31 (t, *J* = 7.8 Hz, 1H, Ar-H^6^), 7.06 (t, *J* = 7.3 Hz, 1H, Ar-H^5^), 6.76 (d, *J* = 7.8 Hz, 1H, Ar-H^7^), 4.59–4.42 (m, 2H, N-CH_2_-CO), 4.25 (q, *J* = 7.1 Hz, 2H, OCH_2_), 3.85 (d, *J* = 17.4 Hz, 1H, CO-CH_2_-C), 3.57 (d, *J* = 17.4 Hz, 1H, CO-CH_2_-C), 1.30 (t, *J* = 7.2 Hz, 3H, CH_3_). ^13^C NMR (101 MHz, CDCl_3_) δ 198.5, 176.2, 167.7, 142.3, 136.4, 133.9, 130.0, 129.9, 128.7, 128.3, 124.3, 123.4, 108.6, 74.5, 61.9, 44.4, 41.4, 14.1. HRMS *m*/*z*: 354.1322 [M + H]^+^, calcd for C_20_H_20_NO_5_: 354.1336.

##### Ethyl 2-(3-hydroxy-2-oxo-3-(2-oxo-2-(p-tolyl)ethyl)indolin-1-yl)acetate (**3d**)

White powder, 63% yield, m.p. 143.6–144.4 °C. ^1^H NMR (400 MHz, CDCl_3_) δ 7.82 (d, *J* = 8.2 Hz, 2H, Ar-H^3′^, Ar-H^5′^), 7.45 (d, *J* = 7.4 Hz, 1H, Ar-H^4^), 7.30 (t, *J* = 7.6 Hz, 1H, Ar-H^6^), 7.25 (d, *J* = 8.1 Hz, 2H, Ar-H^2′^, Ar-H^6′^), 7.06 (t, *J* = 7.5 Hz, 1H, Ar-H^5^), 6.76 (d, *J* = 7.8 Hz, 1H, Ar-H^7^), 5.05 (s, 1H, OH), 4.56 (d, *J* = 17.6 Hz, 1H, N-CH_2_-CO), 4.44 (d, *J* = 17.6 Hz, 1H, N-CH_2_-CO), 4.25 (q, *J* = 7.1 Hz, 1H, OCH_2_), 3.80 (d, *J* = 17.3 Hz, 1H, CO-CH_2_-C), 3.52 (d, *J* = 17.3 Hz, 1H, CO-CH_2_-C), 2.41 (s, 3H, CH_3_), 1.30 (t, *J* = 7.1 Hz, 3H, CH_2_CH_3_). ^13^C NMR (101 MHz, CDCl_3_) δ 198.4, 176.2, 167.7, 144.9, 142.2, 134.0, 130.1, 129.8, 129.4, 128.4, 124.3, 123.4, 108.5, 74.7, 61.9, 44.0, 41.4, 21.7, 14.2. HRMS *m*/*z*: 368.1479 [M + H]^+^, calcd for C_21_H_22_NO_5_: 368.1492.

##### Ethyl 2-(3-(2-(4-ethylphenyl)-2-oxoethyl)-3-hydroxy-2-oxoindolin-1-yl)acetate (**3e**)

White powder, 63% yield, m.p. 139.3–140.0 °C. ^1^H NMR (400 MHz, CDCl_3_) δ 7.84 (d, *J* = 8.0 Hz, 2H, Ar-H^3′^, Ar-H^5′^), 7.45 (d, *J* = 7.3 Hz, 1H, Ar-H^4^), 7.36–7.21 (m, 3H, Ar-H^2′^, Ar-H^6′^, Ar-H^6^), 7.06 (t, *J* = 7.5 Hz, 1H, Ar-H^5^), 6.76 (d, *J* = 7.8 Hz, 1H, Ar-H^7^), 5.01 (s, 1H, OH), 4.57 (d, *J* = 17.6 Hz, 1H, N-CH_2_-CO), 4.42 (d, *J* = 17.6 Hz, 1H, N-CH_2_-CO), 4.24 (q, *J* = 7.0 Hz, 2H, OCH_2_), 3.80 (d, *J* = 17.3 Hz, 1H, CO-CH_2_-C), 3.50 (d, *J* = 17.3 Hz, 1H, CO-CH_2_-C), 2.70 (q, *J* = 7.5 Hz, 2H, CH_2_), 1.35–1.19 (m, 6H, (CH_3_)_2_). ^13^C NMR (101 MHz, CDCl_3_) δ 198.5, 176.1, 167.6, 151.1, 142.2, 134.2, 130.1, 129.9, 128.5, 128.3, 124.4, 123.4, 108.6, 74.7, 61.9, 44.0, 41.4, 29.0, 15.1, 14.2. HRMS *m*/*z*: 382.1635 [M + H]^+^, calcd for C_22_H_24_NO_5_: 382.1649.

##### Ethyl 2-(3-hydroxy-3-(2-(4-isopropylphenyl)-2-oxoethyl)-2-oxoindolin-1-yl)acetate (**3f**)

White powder, 26% yield, m.p. 128.7–129.5 °C. ^1^H NMR (400 MHz, CDCl_3_) δ 7.86 (d, *J* = 8.3 Hz, 2H, Ar-H^3′^, Ar-H^5′^), 7.45 (d, *J* = 7.3 Hz, 1H, Ar-H^4^), 7.39–7.25 (m, 3H, Ar-H^2′^, Ar-H^6′^, Ar-H^6^), 7.06 (t, *J* = 7.5 Hz, 1H, Ar-H^5^), 6.76 (d, *J* = 7.8 Hz, 1H, Ar-H^7^), 5.01 (s, 1H, OH), 4.58 (d, *J* = 17.6 Hz, 1H, N-CH_2_-CO), 4.42 (d, *J* = 17.6 Hz, 1H, N-CH_2_-CO), 4.25 (q, *J* = 7.1 Hz, 2H, OCH_2_), 3.80 (d, *J* = 17.3 Hz, 1H, CO-CH_2_-C), 3.50 (d, *J* = 17.3 Hz, 1H, CO-CH_2_-C), 2.97 (hept, *J* = 6.9 Hz, 1H, CH), 1.29 (m, *J* = 9H, (CH_3_)_3_). ^13^C NMR (101 MHz, CDCl_3_) δ 198.6, 176.1, 167.6, 155.6, 142.2, 134.3, 130.1, 129.9, 128.6, 126.8, 124.4, 123.4, 108.6, 74.7, 61.9, 43.9, 41.4, 34.3, 23.6, 14.2. HRMS *m*/*z*: 396.1789 [M + H]^+^, calcd for C_23_H_26_NO_5_: 396.1805.

##### Ethyl 2-(3-hydroxy-2-oxo-3-(2-oxo-2-(4-pentylphenyl)ethyl)indolin-1-yl)acetate (**3g**)

White powder, 16% yield, m.p. 75.2–76.8 °C. ^1^H NMR (400 MHz, CDCl_3_) δ 7.84 (d, *J* = 8.0 Hz, 2H, Ar-H^3′^, Ar-H^5′^), 7.46 (d, *J* = 7.4 Hz, 1H, Ar-H^4^), 7.29 (dd, *J* = 14.9, 6.9 Hz, 3H, Ar-H^6^, Ar-H^2′^, Ar-H^6′^), 7.06 (t, *J* = 7.5 Hz, 1H, Ar-H^5^), 6.76 (d, *J* = 7.8 Hz, 1H, Ar-H^7^), 4.98 (s, 1H, OH), 4.58 (d, *J* = 17.6 Hz, 1H, N-CH_2_-CO), 4.41 (d, *J* = 17.6 Hz, 1H, N-CH_2_-CO), 4.25 (q, *J* = 7.1 Hz, 2H, OCH_2_), 3.79 (d, *J* = 17.3 Hz, 1H, CO-CH_2_-C), 3.49 (d, *J* = 17.3 Hz, 1H, CO-CH_2_-C), 2.66 (t, *J* = 7.7 Hz, 2H, Ar-CH_2_), 1.63 (p, *J* = 7.1 Hz, 2H, Ar-CH_2_CH_2_), 1.31 (q, *J* = 7.0 Hz, 7H, CH_2_CH_2_CH_3_, CH_2_CH_2_CH_3_, OCH_2_CH_3_), 0.90 (t, *J* = 6.8 Hz, 3H, CH_2_CH_2_CH_3_). ^13^C NMR (101 MHz, CDCl_3_) δ 198.7, 176.1, 167.6, 149.9, 142.2, 134.2, 130.1, 129.9, 128.8, 128.5, 124.4, 123.4, 108.6, 74.8, 61.9, 43.9, 41.4, 36.0, 31.4, 30.7, 22.5, 14.1, 14.0. HRMS *m*/*z*: 424.2103 [M + H]^+^, calcd for C_25_H_30_NO_5_: 424.2118.

##### Ethyl 2-(3-hydroxy-3-(2-(4-methoxyphenyl)-2-oxoethyl)-2-oxoindolin-1-yl)acetate (**3h**)

White powder, 31% yield, m.p. 113.6–114.3 °C. ^1^H NMR (400 MHz, CDCl_3_) δ 7.90 (d, *J* = 8.8 Hz, 2H, Ar-H^2′^, Ar-H^6′^), 7.46 (d, *J* = 7.3 Hz, 1H, Ar-H^4^), 7.30 (t, *J* = 7.6 Hz, 1H, Ar-H^6^), 7.06 (t, *J* = 7.5 Hz, 1H, Ar-H^5^), 6.92 (d, *J* = 8.8 Hz, 2H, Ar-H^3′^, Ar-H^5′^), 6.76 (d, *J* = 7.8 Hz, 1H, Ar-H^7^), 5.14 (s, 1H, OH), 4.59 (d, *J* = 17.6 Hz, 1H, N-CH_2_-CO), 4.39 (d, *J* = 17.6 Hz, 1H, N-CH_2_-CO), 4.24 (q, *J* = 7.1 Hz, 2H, OCH_2_), 3.87 (s, 3H, OCH_3_), 3.72 (t, *J* = 17.1 Hz, 1H, CO-CH_2_-C), 3.45 (d, *J* = 17.1 Hz, 1H, CO-CH_2_-C), 1.30 (t, *J* = 7.1 Hz, 3H, CH_3_). ^13^C NMR (101 MHz, CDCl_3_) δ 197.5, 176.1, 167.6, 164.2, 142.1, 130.7, 130.2, 129.8, 129.5, 124.4, 123.4, 113.9, 108.5, 74.8, 61.9, 55.6, 43.5, 41.4, 14.2. HRMS *m*/*z*: 384.1427 [M + H]^+^, calcd for C_21_H_22_NO_6_: 384.1442.

##### Ethyl 2-(3-(2-(4-ethoxyphenyl)-2-oxoethyl)-3-hydroxy-2-oxoindolin-1-yl)acetate (**3i**)

White powder, 84% yield, m.p. 135.7–136.7 °C. ^1^H NMR (400 MHz, CDCl_3_) δ 7.89 (d, *J* = 8.8 Hz, 2H, Ar-H^2′^, Ar-H^6′^), 7.46 (d, *J* = 7.3 Hz, 1H, Ar-H^4^), 7.30 (d, *J* = 7.8 Hz, 1H, Ar-H^6^), 7.06 (t, *J* = 7.5 Hz, 1H, Ar-H^5^), 6.90 (d, *J* = 8.9 Hz, 2H, Ar-H^3′^, Ar-H^5′^), 6.75 (d, *J* = 7.8 Hz, 1H, Ar-H^7^), 5.16 (s, 1H, OH), 4.59 (d, *J* = 17.6 Hz, 1H, N-CH_2_-CO), 4.39 (d, *J* = 17.6 Hz, 1H, N-CH_2_-CO), 4.24 (q, *J* = 7.1 Hz, 2H, OCH_2_), 4.10 (q, *J* = 7.0 Hz, 2H, OCH_2_), 3.73 (d, *J* = 17.1 Hz, 1H, CO-CH_2_-C), 3.44 (d, *J* = 17.1 Hz, 1H, CO-CH_2_-C), 1.44 (t, *J* = 7.0 Hz, 3H, CH_3_), 1.29 (t, *J* = 7.1 Hz, 3H, CH_3_). ^13^C NMR (101 MHz, CDCl_3_) δ 197.5, 176.1, 167.6, 163.6, 142.1, 130.7, 130.2, 129.8, 129.3, 124.4, 123.4, 114.3, 108.5, 74.8, 63.9, 61.9, 43.4, 41.4, 14.6, 14.2. HRMS *m*/*z*: 398.1584 [M + H]^+^, calcd for C_22_H_24_NO_6_: 398.1598.

##### Ethyl 2-(3-hydroxy-2-oxo-3-(2-oxo-2-(3,4,5-trimethoxyphenyl)ethyl)indolin-1-yl)acetate (**3j**)

White powder, 65% yield, m.p. 121.7–122.4 °C. ^1^H NMR (400 MHz, CDCl_3_) δ 7.43 (d, *J* = 7.1 Hz, 1H, Ar-H^4^), 7.30 (td, *J* = 7.8, 0.9 Hz, 1H, Ar-H^6^), 7.15 (s, 2H, Ar-H^2′^, Ar-H^6′^), 7.06 (t, *J* = 7.5 Hz, 1H, Ar-H^5^), 6.75 (d, *J* = 7.8 Hz, 1H, Ar-H^7^), 4.93 (s, 1H, OH), 4.55 (d, *J* = 17.6 Hz, 1H, N-CH_2_-CO), 4.40 (d, *J* = 17.6 Hz, 1H, N-CH_2_-CO), 4.21 (q, *J* = 7.1 Hz, 2H, OCH_2_), 3.88 (d, 9H, 3′, 4′, 5′-(OCH_3_)_3_), 3.79 (d, *J* = 17.1 Hz, 1H, C-CH_2_-CO), 3.50 (d, *J* = 7.1 Hz, 1H, C-CH_2_-CO), 1.28 (t, *J* = 7.1 Hz, 3H, CH_3_). ^13^C NMR (101 MHz, CDCl_3_) δ 197.5, 176.3, 167.6, 153.1, 143.2, 142.2, 131.5, 130.0, 129.9, 124.3, 123.5, 108.6, 105.7, 74.7, 61.9, 61.0, 56.3, 43.9, 41.4, 14.1. HRMS *m*/*z*: 444.1637 [M + H]^+^, calcd for C_23_H_26_NO_8_: 444.1653.

##### Ethyl 2-(3-(2-(4-fluorophenyl)-2-oxoethyl)-3-hydroxy-2-oxoindolin-1-yl)acetate (**3k**)

White powder, 96% yield, m.p. 154.6–155.4 °C. ^1^H NMR (400 MHz, CDCl_3_) δ 7.94 (dd, *J* = 8.7, 5.4 Hz, 2H, Ar-H^2′^, Ar-H^6^), 7.43 (d, *J* = 7.3 Hz, 1H, Ar-H^4^), 7.31 (t, *J* = 6.2 Hz, 1H, Ar-H^6^), 7.12 (t, *J* = 8.6 Hz, 2H, Ar-H^3′^, Ar-H^5′^), 7.07 (t, *J* = 7.6 Hz, 1H, Ar-H^5^), 6.76 (d, *J* = 7.8 Hz, 1H, Ar-H^7^), 4.53 (d, *J* = 17.6 Hz, 1H, N-CH_2_-CO), 4.47 (d, *J* = 17.7 Hz, 1H, N-CH_2_-CO), 4.25 (q, *J* = 7.1 Hz, 2H, OCH_2_), 3.82 (d, *J* = 17.3 Hz, 1H, N-CH_2_-CO), 3.54 (d, *J* = 17.3 Hz, 1H, N-CH_2_-CO), 1.31 (t, *J* = 7.1 Hz, 3H, CH_3_). ^13^C NMR (101 MHz, CDCl_3_) δ 196.8, 176.3, 167.8, 166.2 (*J* = 256.0 Hz), 142.3, 132.8 (*J* = 3.0 Hz), 131.0 (*J* = 9.5 Hz), 129.9, 124.2, 123.5, 115.9 (*J* = 22.0 Hz), 108.6, 74.4, 60.0, 44.4, 41.4, 14.1. HRMS *m*/*z*: 372.1228 [M + H]^+^, calcd for C_20_H_19_FNO_5_: 372.1242.

##### Ethyl 2-(3-(2-(4-chlorophenyl)-2-oxoethyl)-3-hydroxy-2-oxoindolin-1-yl)acetate (**3l**)

White powder, 86% yield, m.p. 152.7–153.5 °C. ^1^H NMR (400 MHz, CDCl_3_) δ 7.85 (d, *J* = 8.5 Hz, 2H, Ar-H^2′^, Ar-H^6′^), 7.46–7.39 (m, 3H, Ar-H^3′^, Ar-H^5′^, Ar-H^4^), 7.32 (d, *J* = 7.7 Hz, 1H, Ar-H^6^), 7.07 (t, *J* = 7.5 Hz, 1H, Ar-H^5^), 6.76 (d, *J* = 7.8 Hz, 1H, Ar-H^7^), 4.52 (d, *J* = 17.8 Hz, 1H, N-CH_2_-CO), 4.47 (d, *J* = 17.9 Hz, 1H, N-CH_2_-CO), 4.25 (q, *J* = 7.1 Hz, 2H, OCH_2_), 3.82 (d, *J* = 17.3 Hz, 1H, CO-CH_2_-C), 3.54 (d, *J* = 17.3 Hz, 1H, CO-CH_2_-C), 1.30 (t, *J* = 7.1 Hz, 3H, CH_3_). ^13^C NMR (101 MHz, CDCl_3_) δ 197.1, 176.3, 167.9, 142.3, 140.4, 134.7, 130.0, 129.9, 129.7, 129.0, 124.2, 123.5, 108.6, 74.4, 62.0, 44.5, 41.4, 14.2. HRMS *m*/*z*: 388.0933 [M + H]^+^, calcd for C_20_H_19_ClNO_5_: 388.0946.

##### Ethyl 2-(3-(2-(4-bromophenyl)-2-oxoethyl)-3-hydroxy-2-oxoindolin-1-yl)acetate (**3m**)

White powder, 75% yield, m.p. 145.4–146.8 °C. ^1^H NMR (400 MHz, CDCl_3_) δ 7.77 (d, *J* = 8.5 Hz, 2H, Ar-H^3′^, Ar-H^5′^), 7.59 (d, *J* = 8.5 Hz, 2H, Ar-H^2′^, Ar-H^6′^), 7.43 (d, *J* = 7.4 Hz, 1H, Ar-H^4^), 7.31 (t, *J* = 7.8 Hz, 1H, Ar-H^6^), 7.07 (t, *J* = 7.5 Hz, 1H, Ar-H^5^), 6.77 (d, *J* = 7.8 Hz, 1H, Ar-H^7^), 4.55 (d, *J* = 17.6 Hz, 1H, N-CH_2_-CO), 4.43 (d, *J* = 17.6 Hz, 1H, N-CH_2_-CO), 4.25 (q, *J* = 7.1 Hz, 2H, OCH_2_), 3.79 (d, *J* = 17.3 Hz, 1H, CO-CH_2_-C), 3.50 (d, *J* = 17.3 Hz, 1H, CO-CH_2_-C), 1.30 (t, *J* = 7.1 Hz, 3H, CH_3_). ^13^C NMR (101 MHz, CDCl_3_) δ 197.5, 176.1, 167.6, 142.3, 135.1, 132.1, 130.0, 129.8, 129.7, 129.2, 124.3, 123.5, 108.6, 74.5, 62.0, 44.3, 41.4, 14.2. HRMS *m*/*z*: 432.0428 [M + H]^+^, calcd for C_20_H_19_BrNO_5_: 432.0441.

##### Ethyl 2-(3-hydroxy-3-(2-(4-nitrophenyl)-2-oxoethyl)-2-oxoindolin-1-yl)acetate (**3n**)

White powder, 90% yield, m.p. 153.9–154.2 °C. ^1^H NMR (400 MHz, CDCl_3_) δ 8.28 (d, *J* = 8.6 Hz, 2H, Ar-H^3′^, Ar-H^5′^), 8.07 (d, *J* = 8.6 Hz, 2H, Ar-H^2′^, Ar-H^6′^), 7.43 (d, *J* = 7.3 Hz, 1H, Ar-H^4^), 7.33 (t, *J* = 7.7 Hz, 1H, Ar-H^6^), 7.08 (t, *J* = 7.5 Hz, 1H, Ar-H^5^), 6.79 (d, *J* = 7.8 Hz, 1H, Ar-H^7^), 4.53 (d, *J* = 17.6 Hz, 1H, N-CH_2_-CO), 4.45 (d, *J* = 17.6 Hz, 1H, N-CH_2_-CO), 4.25 (q, *J* = 7.1 Hz, 2H, OCH_2_), 3.90 (d, *J* = 17.2 Hz, 1H, CO-CH_2_-C), 3.61 (d, *J* = 17.2 Hz, 1H, CO-CH_2_-C), 1.31 (t, *J* = 7.1 Hz, 3H, CH_3_). ^13^C NMR (101 MHz, CDCl_3_) δ 196.6, 176.2, 167.6, 150.6, 142.4, 140.7, 130.2, 129.4, 129.3, 124.2, 123.9, 123.6, 108.8, 74.4, 62.1, 45.3, 41.4, 14.2. HRMS *m*/*z*: 399.1172 [M + H]^+^, calcd for C_20_H_19_N_2_O_7_: 399.1187.

##### Ethyl 2-(3-(2-([1,1′-biphenyl]-4-yl)-2-oxoethyl)-3-hydroxy-2-oxoindolin-1-yl)acetate (**3o**)

White powder, 20% yield, m.p. 127.9–128.9 °C. ^1^H NMR (400 MHz, CDCl_3_) δ 8.00 (d, *J* = 6.9 Hz, 2H, Diphenyl-H^2^, H^6^), 7.65 (dd, *J* = 21.9, 7.7 Hz, 4H, Diphenyl-H^3^, H^5^, H^2′^, H^6′^), 7.52–7.38 (m, 4H, Ar-H^4^, Diphenyl-H^3′^, H^4′^, H^5′^), 7.32 (t, *J* = 7.2 Hz, 1H, Ar-H^6^), 7.09 (t, *J* = 7.5 Hz, 1H, Ar-H^5^), 6.79 (d, *J* = 7.8 Hz, 1H, Ar-H^7^), 4.59 (d, *J* = 17.6 Hz, 1H, N-CH_2_-CO), 4.44 (d, *J* = 17.5 Hz, 1H, N-CH_2_-CO), 4.26 (q, *J* = 7.1 Hz, 2H, OCH_2_), 3.87 (d, *J* = 17.3 Hz, 1H, CO-CH_2_-C), 3.56 (d, *J* = 17.3 Hz, 1H, CO-CH_2_-C), 1.31 (t, *J* = 7.1 Hz, 3H, CH_3_). ^13^C NMR (101 MHz, CDCl_3_) δ 198.4, 176.2, 167.6, 146.6, 142.2, 139.6, 135.1, 130.0, 129.9, 129.0, 128.9, 128.4, 127.4, 127.3, 124.4, 123.5, 108.6, 74.7, 61.9, 44.2, 41.5, 14.2. HRMS *m*/*z*: 430.1634 [M + H]^+^, calcd for C_26_H_24_NO_5_: 430.1649.

##### Ethyl 2-(3-hydroxy-3-(2-(naphthalen-2-yl)-2-oxoethyl)-2-oxoindolin-1-yl)acetate (**3p**)

White powder, 84% yield, m.p. 138.6–139.9 °C. ^1^H NMR (400 MHz, CDCl_3_) δ 8.45 (s, 1H, Napht-H^1^), 7.97 (dd, *J* = 8.7, 1.4 Hz, 1H, Napht-H^4^), 7.94 (d, *J* = 8.1 Hz, 1H, Napht-H^8^), 7.87 (d, *J* = 8.9 Hz, 1H, Napht-H^3^), 7.86 (d, *J* = 8.0 Hz, 1H, Napht-H^5^), 7.62 (t, *J* = 7.1 Hz, 1H, Napht-H^7^), 7.55 (t, *J* = 7.2 Hz, 1H, Napht-H^6^), 7.50 (d, *J* = 7.3 Hz, 1H, Ar-H^4^), 7.32 (t, *J* = 7.7 Hz, 1H, Ar-H^6^), 7.07 (t, *J* = 7.5 Hz, 1H, Ar-H^5^), 6.78 (d, *J* = 7.8 Hz, 1H, Ar-H^7^), 4.57 (d, *J* = 17.6 Hz, 1H, N-CH_2_-CO), 4.48 (d, *J* = 17.6 Hz, 1H, N-CH_2_-CO), 4.26 (q, *J* = 7.1 Hz, 2H, OCH_2_), 4.00 (d, *J* = 17.2 Hz, 1H, CO-CH_2_-C), 3.69 (d, *J* = 17.3 Hz, 1H, CO-CH_2_-C), 1.31 (t, *J* = 7.1 Hz, 3H, CH_3_). ^13^C NMR (101 MHz, CDCl_3_) δ 198.6, 176.3, 167.9, 142.3, 135.9, 133.7, 132.4, 130.6, 130.1, 129.9, 129.9, 128.9, 128.6, 127.8, 127.0, 124.3, 123.5, 108.6, 74.7, 62.0, 44.4, 41.4, 14.2. HRMS *m*/*z*: 404.1479 [M + H]^+^, calcd for C_24_H_22_NO_5_: 404.1492.

##### Ethyl 2-(3-(2-(furan-2-yl)-2-oxoethyl)-3-hydroxy-2-oxoindolin-1-yl)acetate (**3q**)

White powder, 44% yield, m.p. 136.2–137.7 °C. ^1^H NMR (400 MHz, CDCl_3_) δ 7.59 (s, 1H, furan-H^5^), 7.44 (d, *J* = 7.4 Hz, 1H, Ar-H^4^), 7.30 (t, *J* = 7.6 Hz, 1H, Ar-H^6^), 7.23 (d, *J* = 3.5 Hz, 1H, furan-H^4^), 7.06 (t, *J* = 7.5 Hz, 1H, Ar-H^5^), 6.74 (d, *J* = 7.8 Hz, 1H, Ar-H^7^), 6.54 (d, *J* = 2.0 Hz, 1H, furan-H^3^), 4.88 (s, 1H, OH), 4.55 (d, *J* = 17.6 Hz, 1H, N-CH_2_-CO), 4.39 (d, *J* = 17.6 Hz, 1H, N-CH_2_-CO), 4.23 (q, *J* = 7.1 Hz, 2H, OCH_2_), 3.64 (d, *J* = 16.8 Hz, 1H, CO-CH_2_-C), 3.34 (d, *J* = 16.8 Hz, 1H, CO-CH_2_-C), 1.29 (t, *J* = 7.1 Hz, 3H, CH_3_). ^13^C NMR (101 MHz, CDCl_3_) δ 187.0, 175.98, 167.6, 152.1, 147.4, 142.1, 130.0, 129.6, 124.4, 123.5, 118.8, 112.7, 108.6, 74.7, 61.9, 43.8, 41.4, 14.1. HRMS *m*/*z*: 344.1118 [M + H]^+^, calcd for C_18_H_18_NO_6_: 344.1129.

##### Ethyl 2-(3-hydroxy-2-oxo-3-(2-oxo-2-(1H-pyrrol-2-yl)ethyl)indolin-1-yl)acetate (**3r**)

White powder, 76% yield, m.p. 149.9–151.1 °C. ^1^H NMR (400 MHz, CDCl_3_) δ 9.92 (s, 1H, NH), 7.42 (d, *J* = 7.3 Hz, 1H, Ar-H^4^), 7.30 (t, *J* = 7.7 Hz, 1H, Ar-H^6^), 7.07 (m, 2H, Ar-H^5^, pyrrol-H^5^), 6.89 (m, 1H, pyrrol-H^3^), 6.74 (d, *J* = 7.8 Hz, 1H, Ar-H^7^), 6.25 (d, *J* = 2.4 Hz, 1H, pyrrol-H^4^), 4.56 (d, *J* = 17.6 Hz, 1H, N-CH_2_-CO), 4.37 (d, *J* = 17.6 Hz, 1H, N-CH_2_-CO), 4.23 (q, *J* = 7.1 Hz, 2H, OCH_2_), 3.52 (d, *J* = 16.2 Hz, 1H, CO-CH_2_-C), 3.29 (d, *J* = 16.2 Hz, 1H, CO-CH_2_-C), 1.28 (t, *J* = 7.1 Hz, 3H, CH_3_). ^13^C NMR (101 MHz, CDCl_3_) δ 188.0, 176.4, 167.7, 142.0, 131.6, 129.9, 129.8, 126.5, 124.3, 123.5, 118.5, 111.2, 108.5, 75.0, 61.9, 42.9, 41.4, 14.1. HRMS *m*/*z*: 343.1277 [M + H]^+^, calcd for C_18_H_19_N_2_O_5_: 343.1288.

##### Ethyl 2-(3-hydroxy-2-oxo-3-(2-oxo-2-(pyridin-2-yl)ethyl)indolin-1-yl)acetate (**3s**)

White powder, 93% yield, m.p. 160.4–161.7 °C. ^1^H NMR (400 MHz, CDCl_3_) δ 8.70 (d, *J* = 4.4 Hz, 1H, Pyr-H^6^), 8.11 (d, *J* = 7.8 Hz, 1H, Pyr-H^3^), 7.93 (t, *J* = 7.3 Hz, 1H, Pyr-H^4^), 7.59–7.53 (m, 1H, Pyr-H^5^), 7.34 (t, *J* = 7.4 Hz, 1H, Ar-H^4^), 7.30 (t, *J* = 7.7 Hz, 1H, Ar-H^6^), 7.07 (t, *J* = 7.5 Hz, 1H, Ar-H^5^), 6.76 (d, *J* = 7.8 Hz, 1H, Ar-H^7^), 4.53 (d, *J* = 17.5 Hz, 1H, N-CH_2_-CO), 4.38 (d, *J* = 17.5 Hz, 1H, N-CH_2_-CO), 4.23 (q, *J* = 7.1 Hz, 2H, OCH_2_), 3.90 (d, *J* = 15.3 Hz, 1H, CO-CH_2_-C), 3.59 (d, *J* = 15.3 Hz, 1H, CO-CH_2_-C), 1.27 (t, *J* = 7.1 Hz, 3H, CH_3_). ^13^C NMR (101 MHz, CDCl_3_) δ 198.3, 176.8, 167.5, 152.7, 148.4, 141.9, 137.9, 130.3, 129.8, 127.7, 124.1, 123.4, 122.6, 108.6, 74.5, 61.9, 46.9, 41.5, 14.1. HRMS *m*/*z*: 355.1275 [M + H]^+^, calcd for C_19_H_19_N_2_O_5_: 355.1288.

#### 4.1.4. Method for the Synthesis of Target Compounds **4a**–**q**

A mixture of the corresponding intermediate of **3a**–**q** (200 mg) and HCl (aq, conc, 400 μL) in EtOH (2 mL) was stirred at reflux until the disappearance of the intermediate was evidenced by TLC. After the reaction mixture was cooled to room temperature, the resulting solid was filtered. The solid products were purified by silica gel column chromatography (silica gel, petroleum ether/ethyl acetate = 3:1).

##### Ethyl (*E*)-2-(2-oxo-3-(2-oxopropylidene)indolin-1-yl)acetate (**4a**)

Orange powder, 31% yield, m.p. 111.3–112.4 °C. ^1^H NMR (400 MHz, CDCl_3_) δ 8.54 (d, *J* = 7.7 Hz, 1H, Ar-H^4^), 7.36 (td, *J* = 7.8, 1.1 Hz, 1H, Ar-H^6^), 7.22 (s, 1H, -CO-CH=C), 7.07 (td, *J* = 7.7, 0.8 Hz, 1H, Ar-H^5^), 6.68 (d, *J* = 7.8 Hz, 1H, Ar-H^7^), 4.48 (s, 2H, N-CH_2_-CO), 4.22 (q, *J* = 7.1 Hz, 2H, OCH_2_), 2.49 (s, 3H, COCH_3_), 1.26 (t, *J* = 7.1 Hz, 3H, CH_3_). ^13^C NMR (101 MHz, CDCl_3_) δ 198.4, 168.3, 167.3, 144.8, 134.7, 132.9, 128.4, 128.2, 123.3, 120.2, 108.2, 61.9, 41.5, 32.3, 14.1. HRMS *m*/*z*: 274.1067 [M + H]^+^, calcd for C_15_H_16_NO_4_: 274.1074.

##### Ethyl 2-((*E*)-2-oxo-3-((*E*)-2-oxo-4-phenylbut-3-en-1-ylidene)indolin-1-yl)acetate (**4b**)

Orange powder, 73% yield, m.p. 131.4–132.2 °C. ^1^H NMR (400 MHz, CDCl_3_) δ 8.57 (d, *J* = 7.7 Hz, 1H, Ar-H^4^), 7.80 (d, *J* = 16.2 Hz, 1H, Ph-CH=), 7.69–7.61 (m, 2H, Ar-H^2′^, Ar-H^6′^), 7.58 (s, 1H, CO-CH=), 7.50–7.42 (m, 3H, Ar-H^3′^, Ar-H^4′^, Ar-H^5′^), 7.38 (t, *J* = 7.7 Hz, 1H, Ar-H^6^), 7.10 (t, *J* = 7.8 Hz, 1H, Ar-H^5^), 7.08 (d, *J* = 16.2 Hz, 1H, CO-CH=CH), 6.72 (d, *J* = 7.8 Hz, 1H, Ar-H^7^), 4.53 (s, 2H, N-CH_2_-CO), 4.25 (q, *J* = 7.1 Hz, 2H, OCH_2_), 1.29 (t, *J* = 7.1 Hz, 3H, CH_3_). ^13^C NMR (101 MHz, CDCl_3_) δ 189.9, 168.3, 167.4, 145.4, 144.7, 135.5, 134.3, 132.7, 131.2, 129.1, 128.7, 128.4, 128.3, 127.5, 123.3, 120.4, 108.3, 61.9, 41.5, 14.2. HRMS *m*/*z*: 362.1377 [M + H]^+^, calcd for C_22_H_20_NO_4_: 362.1387.

##### Ethyl (*E*)-2-(2-oxo-3-(2-oxo-2-phenylethylidene)indolin-1-yl)acetate (**4c**)

Orange powder, 60% yield, m.p. 99.1–100.3 °C. ^1^H NMR (400 MHz, CDCl_3_) δ 8.34 (d, *J* = 7.7 Hz, 1H, Ar-H^4^), 8.21–8.05 (m, 2H, Ar-H^2′^, Ar-H^6′^), 7.92 (s, 1H, Ar-CO-CH=C), 7.64 (t, *J* = 7.4 Hz, 1H, Ar-H^4′^), 7.54 (t, *J* = 7.6 Hz, 2H, Ar-H^3′^, Ar-H^5′^), 7.36 (t, *J* = 7.7 Hz, 1H, Ar-H^6^), 7.05 (t, *J* = 7.7 Hz, 1H, Ar-H^5^), 6.71 (d, *J* = 7.8 Hz, 1H, Ar-H^7^), 4.53 (s, 2H, N-CH_2_-CO), 4.24 (q, *J* = 7.1 Hz, 2H, OCH_2_), 1.28 (t, *J* = 7.1 Hz, 3H, CH_3_). ^13^C NMR (101 MHz, CDCl_3_) δ 191.1, 168.0, 167.4, 144.7, 137.5, 135.9, 133.9, 132.6, 129.0, 128.9, 127.9, 126.9, 123.2, 120.2, 108.3, 61.9, 41.5, 14.2. HRMS *m*/*z*: 336.1222 [M + H]^+^, calcd for C_20_H_18_NO_4_: 336.1230.

##### Ethyl (*E*)-2-(2-oxo-3-(2-oxo-2-(p-tolyl)ethylidene)indolin-1-yl)acetate (**4d**)

Orange powder, 64% yield, m.p. 150.8–151.2 °C. ^1^H NMR (400 MHz, CDCl_3_) δ 8.30 (d, *J* = 7.7 Hz, 1H, Ar-H^4^), 8.01 (d, *J* = 8.2 Hz, 2H, Ar-H^2′^, Ar-H^6′^), 7.90 (s, 1H, Ar-CO-CH=C), 7.34 (m, 3H, Ar-H^6^, Ar-H^3′^, Ar-H^5′^), 7.04 (t, *J* = 7.8 Hz, 1H, Ar-H^5^), 6.70 (d, *J* = 7.8 Hz, 1H, Ar-H^7^), 4.52 (s, 2H, N-CH_2_-CO), 4.24 (q, *J* = 7.1 Hz, 2H, OCH_2_), 2.44 (s, 3H, CH_3_-Ar), 1.27 (t, *J* = 7.1 Hz, 3H, CH_3_). ^13^C NMR (101 MHz, CDCl_3_) δ 190.7, 168.0, 167.4, 145.0, 144.6, 135.5, 135.1, 132.4, 129.7, 129.0, 127.8, 127.3, 123.2, 120.2, 108.3, 61.9, 41.5, 21.8, 14.2. HRMS *m*/*z*: 350.1379 [M + H]^+^, calcd for C_21_H_20_NO_4_: 350.1387.

##### Ethyl (*E*)-2-(3-(2-(4-ethylphenyl)-2-oxoethylidene)-2-oxoindolin-1-yl)acetate (**4e**)

Orange powder, 66% yield, m.p. 109.4–110.2 °C. ^1^H NMR (400 MHz, CDCl_3_) δ 8.33 (d, *J* = 7.7 Hz, 1H, Ar-H^4^), 8.06 (d, *J* = 8.3 Hz, 2H, Ar-H^2′^, Ar-H^6′^), 7.93 (s, 1H, Ar-CO-CH=C), 7.36 (m, 3H, Ar-H^6^, Ar-H^3′^, Ar-H^5′^), 7.06 (td, *J* = 7.7, 0.9 Hz, 1H, Ar-H^5^), 6.73 (d, *J* = 7.8 Hz, 1H, Ar-H^7^), 4.55 (s, 2H, N-CH_2_-CO), 4.26 (q, *J* = 7.1 Hz, 2H, OCH_2_), 2.76 (q, *J* = 7.6 Hz, 2H, CH_2_-Ar), 1.37–1.23 (m, 6H, (CH_3_)_2_). ^13^C NMR (101 MHz, CDCl_3_) δ 190.8, 168.0, 167.4, 151.2, 144.6, 135.5, 135.3, 132.4, 129.1, 128.5, 127.8, 127.4, 123.2, 120.2, 108.3, 61.9, 41.5, 29.1, 15.2, 14.2. HRMS *m*/*z*: 364.1535 [M + H]^+^, calcd for C_22_H_22_NO_4_: 364.1543.

##### Ethyl (*E*)-2-(3-(2-(4-isopropylphenyl)-2-oxoethylidene)-2-oxoindolin-1-yl)acetate (**4f**)

Orange powder, 55% yield, m.p. 97.3–98.2 °C. ^1^H NMR (400 MHz, CDCl_3_) δ 8.31 (d, *J* = 7.7 Hz, 1H, Ar-H^4^), 8.05 (d, *J* = 8.3 Hz, 2H, Ar-H^2′^, Ar-H^6′^), 7.91 (s, 1H, Ar-CO-CH=C), 7.38 (d, *J* = 8.3 Hz, 2H, Ar-H^3′^, Ar-H^5′^), 7.34 (td, *J* = 7.8, 1.0 Hz, 1H, Ar-H^6^), 7.04 (td, *J* = 7.7, 0.7 Hz, 1H, Ar-H^5^), 6.71 (d, *J* = 7.8 Hz, 1H, Ar-H^7^), 4.53 (s, 2H, N-CH_2_-CO), 4.24 (q, *J* = 7.1 Hz, 2H, OCH_2_), 3.00 (m, 1H, CH), 1.45–1.18 (m, 9H, (CH_3_)_3_). ^13^C NMR (101 MHz, CDCl_3_) δ 190.7, 168.0, 167.4, 155.7, 144.6, 135.5, 135.4, 132.4, 129.2, 127.8, 127.4, 127.1, 123.2, 120.2, 108.3, 61.9, 41.5, 34.4, 23.7, 14.2. HRMS *m*/*z*: 378.1692 [M + H]^+^, calcd for C_23_H_24_NO_4_: 378.1700.

##### Ethyl (*E*)-2-(2-oxo-3-(2-oxo-2-(4-pentylphenyl)ethylidene)indolin-1-yl)acetate (**4g**)

Orange powder, 89% yield, m.p. 75.3–76.5 °C.^1^H NMR (400 MHz, CDCl_3_) δ 8.32 (d, *J* = 7.9 Hz, 1H, Ar-H^4^), 8.05 (d, *J* = 8.3 Hz, 2H, Ar-H^2′^, Ar-H^6′^), 7.93 (s, 1H, Ar-CO-CH=C), 7.38 (s, 1H, Ar-H^6^), 7.35 (d, *J* = 8.0 Hz, 2H, Ar-H^3′^, Ar-H^5′^), 7.06 (t, *J* = 7.2 Hz, 1H, Ar-H^5^), 6.73 (d, *J* = 7.8 Hz, 1H, Ar-H^7^), 4.55 (s, 2H, N-CH_2_-CO), 4.26 (q, *J* = 7.1 Hz, 2H, OCH_2_), 2.71 (t, *J* = 7.8 Hz, 2H, Ar-CH_2_), 1.67 (m, 2H, Ar-CH_2_CH_2_), 1.38–1.31 (m, 7H, CH_2_CH_2_CH_3_, CH_2_CH_2_CH_3_, OCH_2_CH_3_), 0.92 (t, *J* = 6.6 Hz, 3H, CH_2_CH_2_CH_3_). ^13^C NMR (101 MHz, CDCl_3_) δ 190.8, 168.0, 167.4, 150.0, 144.6, 135.5, 135.3, 132.4, 129.0, 129.0, 127.8, 127.5, 123.2, 120.2, 108.3, 61.9, 41.5, 36.1, 31.4, 30.7, 22.5, 14.2, 14.0. HRMS *m*/*z*: 406.2004 [M + H]^+^, calcd for C_25_H_28_NO_4_: 406.2013.

##### Ethyl (*E*)-2-(3-(2-(4-methoxyphenyl)-2-oxoethylidene)-2-oxoindolin-1-yl)acetate (**4h**)

Orange powder, 42% yield, m.p. 105.1–106.8 °C. ^1^H NMR (400 MHz, CDCl_3_) δ 8.26 (d, *J* = 7.7 Hz, 1H, Ar-H^4^), 8.10 (d, *J* = 8.8 Hz, 2H, Ar-H^2′^, Ar-H^6′^), 7.88 (s, 1H, Ar-CO-CH=C), 7.33 (t, *J* = 7.7 Hz, 1H, Ar-H^6^), 7.03 (t, *J* = 7.8 Hz, 1H, Ar-H^5^), 6.99 (d, *J* = 8.8 Hz, 2H, Ar-H^3′^, Ar-H^5′^), 6.70 (d, *J* = 7.8 Hz, 1H, Ar-H^7^), 4.53 (s, 2H, N-CH_2_-CO), 4.24 (q, *J* = 7.1 Hz, 2H, OCH_2_), 3.89 (s, 3H, OCH_3_), 1.28 (t, *J* = 7.1 Hz, 3H, CH_3_). ^13^C NMR (101 MHz, CDCl_3_) δ 189.6, 168.0, 167.5, 164.3, 144.4, 135.1, 132.3, 131.3, 130.6, 127.7, 127.6, 123.1, 120.2, 114.2, 108.3, 61.9, 55.6, 41.5, 14.2. HRMS *m*/*z*: 366.1330 [M + H]^+^, calcd for C_21_H_20_NO_5_: 366.1336.

##### Ethyl (*E*)-2-(3-(2-(4-ethoxyphenyl)-2-oxoethylidene)-2-oxoindolin-1-yl)acetate (**4i**)

Orange powder, 88% yield, m.p. 123.1–124.6 °C. ^1^H NMR (400 MHz, CDCl_3_) δ 8.26 (d, *J* = 7.6 Hz, 1H, Ar-H^4^), 8.10 (d, *J* = 8.8 Hz, 2H, Ar-H^2′^, Ar-H^6′^), 7.90 (s, 1H, Ar-CO-CH=C), 7.34 (t, *J* = 7.5 Hz, 1H, Ar-H^6^), 7.04 (t, *J* = 7.7 Hz, 1H, Ar-H^5^), 6.99 (d, *J* = 8.8 Hz, 2H, Ar-H^3′^, Ar-H^5′^), 6.72 (d, *J* = 7.8 Hz, 1H, Ar-H^7^), 4.54 (s, 2H, N-CH_2_-CO), 4.25 (q, *J* = 7.1 Hz, 2H, OCH_2_), 4.14 (q, *J* = 7.0 Hz, 2H, OCH_2_), 1.47 (t, *J* = 7.0 Hz, 3H, CH_3_), 1.29 (t, *J* = 7.1 Hz, 3H, CH_3_). ^13^C NMR (101 MHz, CDCl_3_) δ 189.6, 168.1, 167.5, 163.8, 144.4, 135.0, 132.2, 131.4, 130.4, 127.8, 127.7, 123.2, 120.2, 114.6, 108.3, 64.0, 61.9, 41.5, 14.7, 14.2. HRMS *m*/*z*: 380.1485 [M + H]^+^, calcd for C_22_H_22_NO_5_: 380.1492.

##### Ethyl (*E*)-2-(2-oxo-3-(2-oxo-2-(3,4,5-trimethoxyphenyl)ethylidene)indolin-1-yl)acetate (**4j**)

Orange powder, 84% yield, m.p. 127.1–128.6 °C. ^1^H NMR (400 MHz, CDCl_3_) δ 8.28 (d, *J* = 7.7 Hz, 1H, Ar-H^4^), 7.87 (s, 1H, Ar-CO-CH=C), 7.51–7.30 (m, 3H, Ar-H^6^, Ar-H^2′^, Ar-H^6′^), 7.05 (t, *J* = 7.7 Hz, 1H, Ar-H^5^), 6.71 (d, *J* = 7.8 Hz, 1H, Ar-H^7^), 4.53 (s, 2H, N-CH_2_-CO), 4.24 (q, *J* = 7.0 Hz, 2H, OCH_2_), 3.95 (s, 9H, 3′, 4′, 5′-(OCH_3_)_3_), 1.28 (t, *J* = 7.1 Hz, 3H, CH_3_). ^13^C NMR (101 MHz, CDCl_3_) δ 189.9, 168.0, 167.4, 153.3, 144.6, 143.4, 135.9, 132.7, 132.6, 127.8, 127.0, 123.2, 120.1, 108.3, 106.2, 62.0, 61.1, 56.4, 41.5, 14.2. HRMS *m*/*z*: 426.1536 [M + H]^+^, calcd for C_23_H_24_NO_7_: 426.1547.

##### Ethyl (*E*)-2-(3-(2-(4-fluorophenyl)-2-oxoethylidene)-2-oxoindolin-1-yl)acetate (**4k**)

Orange powder, 64% yield, m.p. 123.4–124.7 °C. ^1^H NMR (400 MHz, CDCl_3_) δ 8.34 (d, *J* = 7.6 Hz, 1H, Ar-H^4^), 8.16 (dd, *J* = 7.5, 5.8 Hz, 2H, Ar-H^2′^, Ar-H^6′^), 7.88 (s, 1H, Ar-CO-CH=C), 7.37 (t, *J* = 7.6 Hz, 1H, Ar-H^6^), 7.22 (t, *J* = 8.3 Hz, 2H, Ar-H^3′^, Ar-H^5′^), 7.07 (t, *J* = 7.6 Hz, 1H, Ar-H^5^), 6.73 (d, *J* = 7.8 Hz, 1H, Ar-H^7^), 4.54 (s, 2H, N-CH_2_-CO), 4.26 (q, *J* = 7.0 Hz, 2H, OCH_2_), 1.30 (t, *J* = 7.0 Hz, 3H, CH_3_). ^13^C NMR (101 MHz, CDCl_3_) δ 189.4, 167.9, 167.4, 166.2 (*J* = 257.6 Hz), 144.7, 136.2, 134.0 (*J* = 3.0 Hz), 132.8, 131.6 (*J* = 10.1 Hz), 127.9, 126.3, 123.2, 120.1, 116.2 (*J* = 22.2 Hz), 108.4, 62.0, 41.5, 14.2. HRMS *m*/*z*: 354.1129 [M + H]^+^, calcd for C_20_H_17_FNO_4_: 354.1136.

##### Ethyl (*E*)-2-(3-(2-(4-chlorophenyl)-2-oxoethylidene)-2-oxoindolin-1-yl)acetate (**4l**)

Orange powder, 64% yield, m.p. 130.3–131.6 °C. ^1^H NMR (400 MHz, CDCl_3_) δ 8.37 (d, *J* = 7.7 Hz, 1H, Ar-H^4^), 8.06 (d, *J* = 8.5 Hz, 2H, Ar-H^2′^, Ar-H^6′^), 7.86 (s, 1H, Ar-CO-CH=C), 7.51 (d, *J* = 8.4 Hz, 2H, Ar-H^3′^, Ar-H^5′^), 7.37 (t, *J* = 7.7 Hz, 1H, Ar-H^6^), 7.07 (t, *J* = 7.7 Hz, 1H, Ar-H^5^), 6.72 (d, *J* = 7.8 Hz, 1H, Ar-H^7^), 4.53 (s, 2H, N-CH_2_-CO), 4.25 (q, *J* = 7.1 Hz, 2H, OCH_2_), 1.29 (t, *J* = 7.1 Hz, 3H, CH_3_). ^13^C NMR (101 MHz, CDCl_3_) δ 189.67, 167.88, 167.36, 144.82, 140.42, 136.49, 135.91, 132.91, 130.22, 129.28, 128.06, 125.87, 123.26, 120.04, 108.38, 61.96, 41.50, 14.18. HRMS *m*/*z*: 370.0834 [M + H]^+^, calcd for C_20_H_17_ClNO_4_: 370.0841.

##### Ethyl (*E*)-2-(3-(2-(4-bromophenyl)-2-oxoethylidene)-2-oxoindolin-1-yl)acetate (**4m**)

Orange powder, 66% yield, m.p. 137.2–138.6 °C. ^1^H NMR (400 MHz, CDCl_3_) δ 8.37 (d, *J* = 7.7 Hz, 1H, Ar-H^4^), 7.97 (d, *J* = 8.4 Hz, 2H, Ar-H^2′^, Ar-H^6′^), 7.84 (s, 1H, Ar-CO-CH=C), 7.67 (d, *J* = 8.4 Hz, 2H Ar-H^3′^, Ar-H^5′^), 7.36 (t, *J* = 7.7 Hz, 1H, Ar-H^6^), 7.06 (t, *J* =7.7 Hz, 1H, Ar-H^5^), 6.71 (d, *J* = 7.9 Hz, 1H, Ar-H^7^), 4.52 (s, 2H, N-CH_2_-CO), 4.24 (q, *J* = 7.1 Hz, 2H, OCH_2_), 1.28 (t, *J* = 7.1 Hz, 3H, CH_3_). ^13^C NMR (101 MHz, CDCl_3_) δ 189.9, 167.9, 167.4, 144.8, 136.6, 136.3, 132.9, 132.3, 130.3, 129.3, 128.1, 125.8, 123.3, 120.1, 108.4, 62.0, 41.5, 14.2. HRMS *m*/*z*: 414.0327 [M + H]^+^, calcd for C_20_H_17_BrNO_4_: 414.0335.

##### Ethyl (*E*)-2-(3-(2-(4-nitrophenyl)-2-oxoethylidene)-2-oxoindolin-1-yl)acetate (**4n**)

Orange powder, 83% yield, m.p. 200.4–201.2 °C. ^1^H NMR (400 MHz, DMSO-*d*_6_) δ 8.41 (d, *J* = 9.0 Hz, 2H, Ar-H^3′^, Ar-H^5′^), 8.32 (d, *J* = 9.0 Hz, 2H, Ar-H^2′^, Ar-H^6′^), 8.18 (d, *J* = 7.5 Hz, 1H, Ar-H^4^), 7.85 (s, 1H, Ar-CO-CH=C), 7.46 (td, *J* = 7.8, 1.1 Hz, 1H, Ar-H^6^), 7.09 (m, 2H, Ar-H^5^, Ar-H^7^), 4.68 (s, 2H, N-CH_2_-CO), 4.17 (q, *J* = 7.1 Hz, 2H, OCH_2_), 1.22 (t, *J* = 7.1 Hz, 3H, CH_3_). ^13^C NMR (101 MHz, DMSO-*d*_6_) δ 190.5, 168.2, 167.5, 150.7, 145.6, 142.0, 136.2, 133.9, 130.6, 127.3, 126.6, 124.6, 123.2, 119.6, 110.1, 61.8, 41.7, 14.5. HRMS *m*/*z*: 381.1074 [M + H]^+^, calcd for C_20_H_17_N_2_O_6_: 381.1081.

##### Ethyl (*E*)-2-(3-(2-([1,1′-biphenyl]-4-yl)-2-oxoethylidene)-2-oxoindolin-1-yl)acetate (**4o**)

Orange powder, 50% yield, m.p. 132.4–133.4 °C. ^1^H NMR (400 MHz, CDCl_3_) δ 8.38 (d, *J* = 7.7 Hz, 1H, Ar-H^4^), 8.18 (d, *J* = 8.3 Hz, 2H, Diphenyl-H^2^, H^6^), 7.95 (s, 1H, Ar-CO-CH=C), 7.74 (d, *J* = 8.3 Hz, 2H, Diphenyl-H^3^, H^5^), 7.65 (d, *J* = 7.5 Hz, 2H, Diphenyl-H^2′^, H^6′^), 7.48 (t, *J* = 7.5 Hz, 2H, Diphenyl-H^3′^, H^5′^), 7.41 (t, *J* = 7.2 Hz, 1H, Diphenyl-H^4′^), 7.35 (t, *J* = 7.7 Hz, 1H, Ar-H^6^), 7.05 (t, *J* = 7.7 Hz, 1H, Ar-H^5^), 6.71 (d, *J* = 7.8 Hz, 1H, Ar-H^7^), 4.53 (s, 2H, N-CH_2_-CO), 4.24 (q, *J* = 7.1 Hz, 2H, OCH_2_), 1.28 (t, *J* = 7.1 Hz, 3H, CH_3_). ^13^C NMR (101 MHz, CDCl_3_) δ 190.5, 168.0, 167.4, 146.5, 144.7, 139.6, 136.3, 135.9, 132.7, 129.5, 129.1, 128.5, 128.0, 127.6, 127.4, 126.9, 123.2, 120.2, 108.3, 62.0, 41.5, 14.2. HRMS *m*/*z*: 412.1535 [M + H]^+^, calcd for C_26_H_22_NO_4_: 412.1543.

##### Ethyl (*E*)-2-(3-(2-(naphthalen-2-yl)-2-oxoethylidene)-2-oxoindolin-1-yl)acetate (**4p**)

Orange powder, 85% yield, m.p. 134.5–135.4 °C. ^1^H NMR (400 MHz, CDCl_3_) δ 8.65 (s, 1H, Napht-H^1^), 8.41 (d, *J* = 7.7 Hz, 1H, Ar-H^4^), 8.19 (dd, *J* = 8.6, 1.5 Hz, 1H, Napht-H^4^), 8.09 (s, 1H, Ar-CO-CH=C), 8.02 (d, *J* = 8.0 Hz, 1H, Napht-H^8^), 7.97 (d, *J* = 8.7 Hz, 1H, Napht-H^5^), 7.91 (d, *J* = 8.1 Hz, 1H, Napht-H^3^), 7.65 (t, *J* = 7.1 Hz, 1H, Napht-H^7^), 7.60 (t, *J* = 7.4 Hz, 1H, Napht-H^6^), 7.37 (t, *J* = 7.7 Hz, 1H, Ar-H^6^), 7.08 (t, *J* = 7.7 Hz, 1H, Ar-H^5^), 6.74 (d, *J* = 7.8 Hz, 1H, Ar-H^7^), 4.57 (s, 2H, N-CH_2_-CO), 4.28 (q, *J* = 7.1 Hz, 2H, OCH_2_), 1.31 (t, *J* = 7.1 Hz, 3H, CH_3_). ^13^C NMR (101 MHz, CDCl_3_) δ 190.9, 168.1, 167.5, 144.7, 135.9, 134.9, 132.6, 132.5, 131.3, 129.9, 129.1, 129.0, 127.9, 127.9, 127.1, 127.0, 123.9, 123.3, 120.2, 108.3, 62.0, 41.5, 14.2. HRMS *m*/*z*: 386.1379 [M + H]^+^, calcd for C_24_H_20_NO_4_: 386.1387.

##### Ethyl (*E*)-2-(3-(2-(furan-2-yl)-2-oxoethylidene)-2-oxoindolin-1-yl)acetate (**4q**)

Orange powder, 34% yield, m.p. 145.7–146.2 °C. ^1^H NMR (400 MHz, CDCl_3_) δ 8.76 (d, *J* = 7.7 Hz, 1H, Ar-H^4^), 7.81 (s, 1H, Ar-CO-CH=C), 7.71 (d, *J* = 0.7 Hz, 1H, furan-H^5^), 7.44 (d, *J* = 3.5 Hz, 1H, furan-H^3^), 7.38 (t, *J* = 7.5 Hz, 1H, Ar-H^6^), 7.10 (t, *J* = 7.6 Hz, 1H, Ar-H^5^), 6.70 (d, *J* = 7.8 Hz, 1H, Ar-H^7^), 6.64 (dd, *J* = 3.5, 1.5 Hz, 1H, furan-H^4^), 4.52 (s, 2H, N-CH_2_-CO), 4.23 (q, *J* = 7.1 Hz, 2H, OCH_2_), 1.27 (t, *J* = 7.1 Hz, 3H, CH_3_). ^13^C NMR (101 MHz, CDCl_3_) δ 177.7, 168.1, 167.4, 154.0, 147.7, 145.0, 137.0, 133.1, 129.2, 124.9, 123.3, 120.3, 119.2, 113.0, 108.2, 61.9, 41.5, 14.2. HRMS *m*/*z*: 326.1017 [M + H]^+^, calcd for C_18_H_16_NO_5_: 326.1023.

#### 4.1.5. Ethyl (*E*)-2-(2-oxo-3-(2-oxo-2-(1H-pyrrol-2-yl)ethylidene)indolin-1-yl)acetate (**4r**)

A mixture of **3r** (200 mg) and HCl (aq, conc, 400 μL) in EtOH (2 mL) was stirred at reflux until the disappearance of **3r** was evidenced by TLC. After the reaction mixture was cooled to room temperature, the mixture was neutralized to a pH value of 7 with a saturated solution of NaHCO_3_ 2 mL of water was added, and the resulting solid was filtered. The solid product was purified by silica gel column chromatography (silica gel, petroleum ether/ethyl acetate = 3:1). Orange powder, 29% yield, m.p. 194.5–195.8 °C. ^1^H NMR (400 MHz, DMSO-*d*_6_) δ 12.28 (s, 1H, NH), 8.56 (d, *J* = 7.6 Hz, 1H, Ar-H^4^), 7.67 (s, 1H, Ar-CO-CH=C), 7.43 (t, *J* = 7.7 Hz, 1H, Ar-H^6^), 7.31 (m, 1H, pyrrol-H^5^), 7.23 (m, 1H, pyrrol-H^3^), 7.10 (t, *J* = 7.9 Hz, 2H, Ar-H^5^, Ar-H^7^), 6.33 (dt, *J* = 4.1, 2.2 Hz, 1H, pyrrol-H^4^), 4.67 (s, 2H, N-CH_2_-CO), 4.18 (q, *J* = 7.1 Hz, 2H, OCH_2_), 1.22 (t, *J* = 7.1 Hz, 3H, CH_3_). ^13^C NMR (101 MHz, DMSO-*d*_6_) δ 178.2, 168.3, 167.9, 145.2, 134.7, 134.2, 133.0, 128.8, 128.2, 127.7, 123.0, 120.1, 119.1, 111.6, 109.7, 61.7, 41.7, 14.5. HRMS *m*/*z*: 325.1176 [M + H]^+^, calcd for C_18_H_17_N_2_O_4_: 325.1183.

#### 4.1.6. Ethyl (*E*)-2-(2-oxo-3-(2-oxo-2-(pyridin-2-yl)ethylidene)indolin-1-yl)acetate (**4s**)

Using the synthesis method of compound **4s** as mentioned above, taking **3s** as the reactant, the target compound **4s** was obtained. Orange powder, 44% yield, m.p. 154.2–155.6 °C. ^1^H NMR (400 MHz, CDCl_3_) δ 8.80 (d, *J* = 4.6 Hz, 1H, Pyr-H^6^), 8.72 (d, *J* = 7.7 Hz, 1H, Pyr-H^3^), 8.69 (s, 1H, Ar-CO-CH=C), 8.23 (d, *J* = 7.8 Hz, 1H, Ar-H^4^), 7.94 (td, *J* = 7.7, 1.6 Hz, 1H, Pyr-H^4^), 7.55 (td, *J* = 7.5, 4.8 Hz, 1H, Pyr-H^5^), 7.40 (t, *J* = 7.7 Hz, 1H, Ar-H^6^), 7.12 (t, *J* = 7.7 Hz, 1H, Ar-H^5^), 6.73 (d, *J* = 7.8 Hz, 1H, Ar-H^7^), 4.55 (s, 2H, N-CH_2_-CO), 4.25 (q, *J* = 7.1 Hz, 2H, OCH_2_), 1.29 (t, *J* = 7.1 Hz, 3H, CH_3_). ^13^C NMR (101 MHz, CDCl_3_) δ 190.7, 168.1, 167.5, 154.1, 149.2, 145.1, 137.2, 137.0, 132.9, 128.6, 127.4, 126.1, 123.1, 122.7, 120.6, 108.2, 61.9, 41.5, 14.2. HRMS *m*/*z*: 337.1175 [M + H]^+^, calcd for C_19_H_17_N_2_O_4_: 337.1183.

#### 4.1.7. Ethyl (*E*)-2-(3-(2-cyclopropyl-2-oxoethylidene)-2-oxoindolin-1-yl)acetate (**4t**)

Step 1: 1-Cyclopropyl-2-bromoacetone (476 μL, 5.0 mmol) was dissolved in 5 mL of THF in a round-bottomed flask. Then, triphenylphosphine (786 mg, 3.0 mmol) was added. The reaction mixture was stirred under reflux at 80 °C for 2 h until the reaction was complete. Subsequently, the solvent was removed under reduced pressure. The residue was washed with ethyl acetate and dried to afford (2-cyclopropyl-2-oxoethyl)triphenylphosphonium bromide (1274 mg, 90%). These crude products were used directly in the next step.

Step 2: To a stirred solution of (2-cyclopropyl-2-oxoethyl)triphenylphosphonium bromide (200 mg, 0.47 mmol) in 10 mL of CH_2_Cl_2_ was added 3 mL of a 1 mol/L sodium hydroxide solution. After stirring for 12 h at room temperature, the reaction mixture was extracted with dichloromethane. The organic layer was dried over anhydrous sodium sulfate, and the solvent was evaporated under reduced pressure to obtain 1-cyclopropyl-2-(triphenylphosphoranylidene)ethanone (157 mg, 97%). These crude products were used directly in the next step.

Step 3: To a stirred solution of ethyl 2-(2,3-dioxoindolin-1-yl) acetate (80 mg, 0.34 mmol) in 2 mL of THF was added 1-cyclopropyl-2-(triphenylphosphoranylidene)ethanone (130 mg, 1.1 eq). After stirring for 0.5 h at 60 °C, the precipitate was filtered, washed with ethanol and dried, affording the pure compound **4t** (59 mg, 57%). Orange powder, m.p. 161.2–162.4 °C. ^1^H NMR (400 MHz, CDCl_3_) δ 8.53 (d, *J* = 7.7 Hz, 1H, Ar-H^4^), 7.39–7.31 (m, 2H, Ar-H^5^, Ar-CO-CH=C), 7.06 (t, *J* = 7.7 Hz, 1H, Ar-H^6^), 6.69 (d, *J* = 7.8 Hz, 1H, Ar-H^7^), 4.50 (s, 2H, N-CH_2_-CO), 4.23 (q, *J* = 7.1 Hz, 2H, OCH_2_), 2.32 (m, *J* = 8.1, 4.5 Hz, 1H, CH_2_CHCH_2_), 1.27 (t, *J* = 7.1 Hz, 5H, CH_3_, CH_2_CHCH_2_), 1.10 (m, *J* = 7.5, 3.8 Hz, 2H, CH_2_CHCH_2_). ^13^C NMR (101 MHz, CDCl_3_) δ 200.8, 168.3, 167.4, 144.7, 134.0, 132.7, 128.6, 128.3, 123.2, 120.3, 108.1, 61.9, 41.5, 23.5, 14.1, 12.8. HRMS *m*/*z*: 300.1224 [M + H]^+^, calcd for C_17_H_18_NO_4_: 300.1230.

#### 4.1.8. Methyl (*E*)-2-(2-oxo-3-(2-oxo-2-(3,4,5-trimethoxyphenyl)ethylidene)indolin-1-yl)acetate (**5a**)

A mixture of **3j** (200 mg) and HCl (aq, conc, 400 μL) in methanol (2 mL) was stirred at reflux until the disappearance of **3j** was evidenced by TLC. After the reaction mixture was cooled to room temperature, the resulting solid was filtered, washed with EtOH and dried. Orange powder, 74% yield, m.p. 143.1–144.6 °C. ^1^H NMR (400 MHz, CDCl_3_) δ 8.30 (d, *J* = 7.6 Hz, 1H, Ar-H^4^), 7.89 (s, 1H, Ar-CO-CH=C), 7.45–7.31 (m, 3H, Ar-H^6^, Ar-H^2′^, Ar-H^6^), 7.07 (t, *J* = 7.6 Hz, 1H, Ar-H^5^), 6.73 (d, *J* = 7.8 Hz, 1H, Ar-H^7^), 4.57 (s, 2H, N-CH_2_-CO), 3.97 (s, 9H, 3′, 4′, 5′-(OCH_3_)_3_), 3.80 (s, 3H, CH_3_). ^13^C NMR (101 MHz, CDCl_3_) δ 189.9, 168.0, 167.9, 153.3, 144.5, 143.4, 135.8, 132.7, 132.6, 127.8, 127.0, 123.3, 120.1, 108.3, 106.2, 61.1, 56.4, 52.8, 41.3. HRMS *m*/*z*: 412.1381 [M + H]^+^, calcd for C_22_H_22_NO_7_: 412.1391.

#### 4.1.9. Heptyl (*E*)-2-(2-oxo-3-(2-oxo-2-(3,4,5-trimethoxyphenyl)ethylidene)indolin-1-yl)acetate (**5b**)

A mixture of **3j** (200 mg) and HCl (aq, conc, 400 μL) in n-heptanol (2 mL) was stirred at reflux until the disappearance of **3j** was evidenced by TLC. After the reaction mixture was cooled to room temperature, 20 mL of deionized water was added, and the resulting solid product was filtered, washed with EtOH and dried. Orange powder, 21% yield, m.p. 82.5–83.4 °C. ^1^H NMR (400 MHz, CDCl_3_) δ 8.30 (d, *J* = 7.6 Hz, 1H, Ar-H^4^), 7.88 (s, 1H, Ar-CO-CH=C), 7.42–7.31 (m, 3H, Ar-H^6^, Ar-H^2′^, Ar-H^6′^), 7.05 (t, *J* = 7.4 Hz, 1H, Ar-H^5^), 6.72 (d, *J* = 7.8 Hz, 1H, Ar-H^7^), 4.54 (s, 2H, N-CH_2_-CO), 4.16 (q, *J* = 6.9 Hz, 2H, OCH_2_), 3.96 (d, 9H, 3′, 4′, 5′-(OCH_3_)_3_), 1.70–1.51 (m, 2H, CH_2_), 1.36–1.17 (m, 8H, (CH_2_)_4_), 0.87 (t, *J* = 6.9 Hz, 3H, CH_3_). ^13^C NMR (101 MHz, CDCl_3_) δ 189.9, 168.0, 167.5, 153.3, 144.6, 143.4, 135.9, 132.7, 132.6, 127.8, 126.9, 123.2, 120.1, 108.3, 106.2, 77.4, 77.1, 76.8, 66.1, 61.1, 56.4, 41.5, 31.7, 28.8, 28.5, 25.7, 22.6, 14.1. HRMS *m*/*z*: 496.2321 [M + H]^+^, calcd for C_28_H_34_NO_7_: 496.2330.

#### 4.1.10. Undecyl (*E*)-2-(2-oxo-3-(2-oxo-2-(3,4,5-trimethoxyphenyl)ethylidene)indolin-1-yl)acetate (**5c**)

Using the synthesis method of compound **5b** as mentioned above, taking undecanol as the solvent, the target compound **5c** was obtained. Orange powder, 52% yield, m.p. 82.4–83.7 °C. ^1^H NMR (400 MHz, CDCl_3_) δ 8.31 (d, *J* = 7.7 Hz, 1H, Ar-H^4^), 7.89 (s, 1H, Ar-CO-CH=C), 7.44–7.31 (m, 3H, Ar-H^6^, Ar-H^2′^, Ar-H^6′^), 7.06 (t, *J* = 7.7 Hz, 1H, Ar-H^5^), 6.73 (d, *J* = 7.8 Hz, 1H, Ar-H^7^), 4.55 (s, 2H, N-CH_2_-CO), 4.18 (t, *J* = 6.7 Hz, 2H, OCH_2_), 3.97 (d, 9H, 3′, 4′, 5′-(OCH_3_)_3_), 1.72–1.54 (m, 2H, CH_2_), 1.26 (s, 16H, (CH_2_)_8_), 0.89 (t, *J* = 6.7 Hz, 3H, CH_3_). ^13^C NMR (101 MHz, CDCl_3_) δ 189.8, 168.0, 167.5, 153.3, 144.6, 135.9, 132.7, 132.5, 127.8, 126.9, 123.2, 120.1, 108.3, 106.3, 66.0, 61.1, 56.4, 41.5, 31.9, 29.6, 29.6, 29.5, 29.3, 29.2, 28.5, 25.8, 22.7, 14.1. HRMS *m*/*z*: 552.2947 [M + H]^+^, calcd for C_32_H_42_NO_7_: 552.2956.

#### 4.1.11. Octadecyl (*E*)-2-(2-oxo-3-(2-oxo-2-(3,4,5-trimethoxyphenyl)ethylidene)indolin-1-yl)acetate (**5d**)

A mixture of **3j** (200 mg) and HCl (aq, conc, 400 μL) in heated liquid octadecanol (500 mg) was stirred at reflux until the disappearance of **3j** was evidenced by TLC. After the reaction mixture was cooled to room temperature, the resulting solid was filtered, washed with 1 mL of EtOH and dried. Orange powder, 60% yield, m.p. 96.5–97.2 °C. ^1^H NMR (400 MHz, CDCl_3_) δ 8.31 (d, *J* = 7.6 Hz, 1H, Ar-H^4^), 7.89 (s, 1H, Ar-CO-CH=C), 7.46–7.32 (m, 3H, Ar-H^6^, Ar-H^2′^, Ar-H^6′^), 7.06 (t, *J* = 7.4 Hz, 1H, Ar-H^5^), 6.73 (d, *J* = 7.8 Hz, 1H, Ar-H^7^), 4.55 (s, 2H, N-CH_2_-CO), 4.18 (t, *J* = 6.7 Hz, 2H, OCH_2_), 3.97 (d, 9H, 3′, 4′, 5′-(OCH_3_)_3_), 1.76–1.50 (m, 2H, CH_2_), 1.27 (s, 30H, (CH_2_)_15_), 0.89 (t, *J* = 6.8 Hz, 3H, CH_3_). ^13^C NMR (101 MHz, CDCl_3_) δ 189.8, 168.0, 167.5, 153.3, 144.6, 143.4, 135.9, 132.7, 132.5, 127.8, 126.9, 123.2, 120.1, 108.3, 106.3, 100.0, 66.1, 61.1, 56.4, 41.5, 31.9, 29.7, 29.7, 29.7, 29.7, 29.6, 29.5, 29.4, 29.2, 28.5, 25.8, 22.7, 14.2. HRMS *m*/*z*: 650.4041 [M + H]^+^, calcd for C_39_H_56_NO_7_: 650.4051.

#### 4.1.12. Benzyl (*E*)-2-(2-oxo-3-(2-oxo-2-(3,4,5-trimethoxyphenyl)ethylidene)indolin-1-yl)acetate (**5e**)

A mixture of **3j** (200 mg) and HCl (aq, conc, 400 μL) in benzyl alcohol (2 mL) was stirred at reflux until the disappearance of **3j** was evidenced by TLC. Then, 5 mL of water was added to the mixture, and the mixture was then extracted with ethyl acetate (3 × 10 mL). The organic fractions were combined, dried over Na_2_SO_4_, filtered and concentrated. The crude product was purified by column chromatography (silica gel, petroleum ether/ethyl acetate = 3:1). Orange powder, 59% yield, m.p. 131.7–132.9 °C. ^1^H NMR (400 MHz, CDCl_3_) δ 8.31 (d, *J* = 7.6 Hz, 1H, Ar-H^4^), 7.89 (s, 1H, Ar-CO-CH=C), 7.42–7.29 (m, 8H, Ar-H^2′^, Ar-H^6′^, Ar-H^6^, Ar-H^2″^, Ar-H^3″^, Ar-H^4″^, Ar-H^5″^, Ar-H^6″^), 7.07 (td, *J* = 7.7, 0.8 Hz, 1H, Ar-H^5^), 6.69 (d, *J* = 7.8 Hz, 1H, Ar-H^7^), 5.23 (s, 2H, OCH_2_Ar), 4.60 (s, 2H, N-CH_2_-CO), 3.97 (d, 9H, 3′, 4′, 5′-(OCH_3_)_3_). ^13^C NMR (101 MHz, CDCl_3_) δ 189.8, 168.0, 167.3, 153.4, 144.5, 143.5, 135.8, 135.0, 132.7, 132.6, 128.7, 128.6, 128.4, 127.8, 127.0, 123.2, 120.2, 108.4, 106.3, 67.6, 61.1, 56.4, 41.5. HRMS *m*/*z*: 488.1693 [M + H]^+^, calcd for C_28_H_26_NO_7_: 488.1704.

#### 4.1.13. Phenethyl (*E*)-2-(2-oxo-3-(2-oxo-2-(3,4,5-trimethoxyphenyl)ethylidene)indolin-1-yl)acetate (**5f**)

Using the synthesis method of compound **5e** as mentioned above, taking 2-phenylethanol as the solvent, the target compound **5f** was obtained. Orange powder, 57% yield, m.p. 106.5–107.8 °C. ^1^H NMR (400 MHz, CDCl_3_) δ 8.31 (d, *J* = 7.7 Hz, 1H, Ar-H^4^), 7.88 (s, 1H, Ar-CO-CH=C), 7.38 (s, 2H, Ar-H^2′^, Ar-H^6′^), 7.34 (t, *J* = 7.7 Hz, 1H, Ar-H^6^), 7.28 (t, *J* = 7.1 Hz, 2H, Ar-H^3′′^, Ar-H^5′′^), 7.22 (t, *J* = 7.2 Hz, 1H, Ar-H^4′^), 7.15 (d, *J* = 7.0 Hz, 2H, Ar-H^2′′^, Ar-H^6″^), 7.07 (t, *J* = 7.7 Hz, 1H, Ar-H^5^), 6.61 (d, *J* = 7.8 Hz, 1H, Ar-H^7^), 4.52 (s, 2H, N-CH_2_-CO), 4.42 (t, *J* = 6.9 Hz, 2H, OCH_2_), 3.97 (d, 9H, 3′, 4′, 5′-(OCH_3_)_3_), 2.95 (t, *J* = 6.8 Hz, 2H, CH_2_Ar). ^13^C NMR (101 MHz, CDCl_3_) δ 189.8, 167.9, 167.3, 153.4, 144.5, 143.5, 137.2, 135.8, 132.7, 132.6, 128.7, 128.6, 127.8, 126.9, 126.7, 123.2, 120.1, 108.3, 106.3, 66.2, 61.1, 56.5, 41.4, 35.0. HRMS *m*/*z*: 502.1849 [M + H]^+^, calcd for C_29_H_28_NO_7_: 502.1860.

#### 4.1.14. 3-Phenylpropyl (*E*)-2-(2-oxo-3-(2-oxo-2-(3,4,5-trimethoxyphenyl)ethylidene)indolin-1-yl)acetate (**5g**)

Using the synthesis method of compound **5e** as mentioned above, taking 3-phenyl-1-propanol as the solvent, the target compound **5g** was obtained. Orange powder, 77% yield, m.p. 103.2–104.4 °C. ^1^H NMR (400 MHz, CDCl_3_) δ 8.33 (d, *J* = 7.7 Hz, 1H, Ar-H^4^), 7.91 (s, 1H, Ar-CO-CH=C), 7.43–7.33 (m, 3H, Ar-H^6^, Ar-H^2′^, Ar-H^6′^), 7.29 (t, *J* = 7.4 Hz, 2H, Ar-H^3″^, Ar-H^5″^), 7.20 (t, *J* = 7.3 Hz, 1H, Ar-H^4″^), 7.13 (d, *J* = 7.5 Hz, 2H, Ar-H^2″^, Ar-H^6″^), 7.08 (t, *J* = 7.7 Hz, 1H, Ar-H^5^), 6.74 (d, *J* = 7.8 Hz, 1H, Ar-H^7^), 4.56 (s, 2H, N-CH_2_-CO), 4.21 (t, *J* = 6.4 Hz, 2H, OCH_2_), 3.97 (d, 9H, 3′, 4′, 5′-(OCH_3_)_3_), 2.64 (t, *J* = 7.6 Hz, 2H, CH_2_Ar), 2.10–1.84 (m, 2H, CCH_2_C). ^13^C NMR (101 MHz, CDCl_3_) δ 189.9, 168.0, 167.5, 153.3, 144.6, 143.3, 140.8, 135.9, 132.7, 132.6, 128.5, 128.4, 127.9, 127.0, 126.2, 123.3, 120.1, 108.4, 106.1, 65.2, 61.1, 58.5, 41.5, 32.0, 30.1. HRMS *m*/*z*: 516.2007 [M + H]^+^, calcd for C_30_H_30_NO_7_: 516.2017.

#### 4.1.15. 4-Phenylbutyl (*E*)-2-(2-oxo-3-(2-oxo-2-(3,4,5-trimethoxyphenyl)ethylidene)indolin-1-yl)acetate (**5h**)

Using the synthesis method of compound **5e** as mentioned above, taking 4-phenyl-1-butanol as the solvent, the target compound **5h** was obtained. Orange powder, 68% yield, m.p. 109.5–110.3 °C. ^1^H NMR (400 MHz, CDCl_3_) δ 8.30 (d, *J* = 7.7 Hz, 1H, Ar-H^4^), 7.88 (s, 1H, Ar-CO-CH=C), 7.39–7.30 (m, 3H, Ar-H^6^, Ar-H^2′^, Ar-H^6′^), 7.27 (t, *J* = 7.4 Hz, 2H, Ar-H^3″^, Ar-H^5″^), 7.18 (d, *J* = 7.3 Hz, 1H, Ar-H^4″^), 7.13 (d, *J* = 7.3 Hz, 2H, Ar-H^2″^, Ar-H^6″^), 7.05 (t, *J* = 7.7 Hz, 1H, Ar-H^5^), 6.70 (d, *J* = 7.8 Hz, 1H, Ar-H^7^), 4.53 (s, 2H, N-CH_2_-CO), 4.19 (t, *J* = 6.1 Hz, 2H, OCH_2_), 3.95 (d, 9H, 3′, 4′, 5′-(OCH_3_)_3_), 2.60 (t, *J* = 7.1 Hz, 2H, CH_2_Ar), 1.77–1.48 (m, 4H, CCH_2_, CH_2_C). ^13^C NMR (101 MHz, CDCl_3_) δ 189.9, 168.0, 167.5, 153.3, 144.6, 143.3, 141.8, 135.9, 132.7, 132.6, 128.4, 127.9, 126.9, 125.9, 123.3, 120.1, 108.3, 106.2, 65.8, 61.1, 56.4, 41.5, 35.3, 28.1, 27.6. HRMS *m*/*z*: 530.2163 [M + H]^+^, calcd for C_31_H_32_NO_7_: 530.2173.

#### 4.1.16. Method for the Synthesis of Intermediate **7a**–**g**

To a solution of isatin with different substituents at the 5-position (500 mg) in DMF (400 μL), K_2_CO_3_ (1.2 eq) and ethyl bromoacetate (1.2 eq) were added. The reaction mixture was stirred at 85 °C until the complete disappearance of it was evidenced by TLC. The reaction mixture was cooled to room temperature and then poured into water. The resulting solid product was filtered, washed with EtOH and dried.

##### Ethyl 2-(5-fluoro-2,3-dioxoindolin-1-yl)acetate (**7a**)

Yellow powder, 62% yield, m.p. 137.5–138.1 °C. ^1^H-NMR was in agreement with those reported in the literature [37]. ^1^H NMR (400 MHz, CDCl_3_) δ 7.38–7.30 (m, 2H, Ar-H^4^, Ar-H^6^), 6.79 (dd, *J* = 8.5, 3.5 Hz, 1H, Ar-H^7^), 4.50 (s, 2H, N-CH_2_-CO), 4.26 (q, *J* = 7.1 Hz, 2H, OCH_2_), 1.30 (t, *J* = 7.1 Hz, 3H, CH_3_). ^13^C NMR (101 MHz, CDCl_3_) δ 181.9, 166.6, 159.5 (d, *J* = 246.4 Hz), 157.8, 146.4, 124.8 (d, *J* = 24.4 Hz), 118.2 (d, *J* = 7.1 Hz), 112.6 (d, *J* = 24.3 Hz), 111.5 (d, *J* = 7.3 Hz), 62.3, 41.4, 14.1. HRMS *m*/*z*: 252.0660 [M + H]^+^, calcd for C_12_H_11_FNO_4_: 252.0667.

##### Ethyl 2-(5-chloro-2,3-dioxoindolin-1-yl)acetate (**7b**)

Yellow powder, 68% yield, m.p. 139.8–140.3 °C. ^1^H-NMR was in agreement with those reported in the literature [37]. ^1^H NMR (400 MHz, CDCl_3_) δ 7.62 (d, *J* = 2.2 Hz, 1H, Ar-H^4^), 7.57 (dd, *J* = 8.4, 2.2 Hz, 1H, Ar-H^6^), 6.78 (d, *J* = 8.4 Hz, 1H, Ar-H^7^), 4.50 (s, 2H, N-CH_2_-CO), 4.26 (q, *J* = 7.1 Hz, 2H, OCH_2_), 1.30 (t, *J* = 7.1 Hz, 3H, CH_3_). ^13^C NMR (101 MHz, CDCl_3_) δ 181.5, 166.5, 157.5, 148.6, 137.8, 130.0, 125.5, 118.4, 111.6, 62.4, 41.4, 14.1. HRMS *m*/*z*: 268.0365 [M + H]^+^, calcd for C_12_H_11_ClNO_4_: 268.0371.

##### Ethyl 2-(5-bromo-2,3-dioxoindolin-1-yl)acetate (**7c**)

Yellow powder, 69% yield, m.p. 132.5–133.2 °C. ^1^H-NMR was in agreement with those reported in the literature [37]. ^1^H NMR (400 MHz, CDCl_3_) δ 7.76 (d, *J* = 1.9 Hz, 1H, Ar-H^4^), 7.72 (dd, *J* = 8.4, 2.0 Hz, 1H, Ar-H^6^), 6.73 (d, *J* = 8.4 Hz, 1H, Ar-H^7^), 4.51 (s, 2H, N-CH_2_-CO), 4.26 (q, *J* = 7.1 Hz, 2H, OCH_2_), 1.30 (t, *J* = 7.1 Hz, 3H, CH_3_). ^13^C NMR (101 MHz, CDCl_3_) δ 181.3, 166.5, 157.3, 149.1, 140.7, 128.3, 118.8, 117.0, 112.0, 62.4, 41.4, 14.1. HRMS *m*/*z*: 311.9859 [M + H]^+^, calcd for C_12_H_11_BrNO_4_: 311.9866.

##### Ethyl 2-(5-iodo-2,3-dioxoindolin-1-yl)acetate (**7d**)

Orange powder, 58% yield, m.p. 143.2–144.6 °C. ^1^H NMR (400 MHz, CDCl_3_) δ 7.94 (d, *J* = 1.7 Hz, 1H, Ar-H^4^), 7.90 (dd, *J* = 8.3, 1.8 Hz, 1H, Ar-H^6^), 6.63 (d, *J* = 8.3 Hz, 1H, Ar-H^7^), 4.49 (s, 2H, N-CH_2_-CO), 4.26 (q, *J* = 7.1 Hz, 2H, OCH_2_), 1.30 (t, *J* = 7.1 Hz, 3H, CH_3_). ^13^C NMR (101 MHz, CDCl_3_) δ 181.1, 166.5, 157.0, 149.7, 146.5, 134.0, 119.1, 112.3, 86.5, 62.4, 41.3, 14.1. HRMS *m*/*z*: 359.9715 [M + H]^+^, calcd for C_12_H_11_INO_4_: 359.9727.

##### Ethyl 2-(5-methyl-2,3-dioxoindolin-1-yl)acetate (**7e**)

Orange powder, 79% yield, m.p. 133.6–134.9 °C. ^1^H-NMR was in agreement with those reported in the literature [37]. ^1^H NMR (400 MHz, CDCl_3_) δ 7.45 (s, 1H, Ar-H^4^), 7.40 (d, *J* = 8.1 Hz, 1H, Ar-H^6^), 6.70 (d, *J* = 8.0 Hz, 1H, Ar-H^7^), 4.47 (s, 2H, N-CH_2_-CO), 4.24 (q, *J* = 7.1 Hz, 2H, OCH_2_), 2.35 (s, 3H, Ar-CH_3_), 1.29 (t, *J* = 7.1 Hz, 3H, CH_3_). ^13^C NMR (101 MHz, CDCl_3_) δ 182.7, 166.9, 158.2, 148.2, 138.9, 134.1, 125.9, 117.7, 110.0, 62.1, 41.3, 20.7, 14.1. HRMS *m*/*z*: 248.0910 [M + H]^+^, calcd for C_13_H_14_NO_4_: 248.0917.

##### Ethyl 2-(5-methoxy-2,3-dioxoindolin-1-yl)acetate (**7f**)

Red powder, 74% yield, m.p. 89.6–90.9 °C (lit. mp: 85–86 °C [38]). ^1^H-NMR was in agreement with those reported in the literature [38]. ^1^H NMR (400 MHz, CDCl_3_) δ 7.18 (d, *J* = 2.5 Hz, 1H, Ar-H^4^), 7.15 (dd, *J* = 8.5, 2.7 Hz, 1H, Ar-H^6^), 6.73 (d, *J* = 8.5 Hz, 1H, Ar-H^7^), 4.47 (s, 2H, N-CH_2_-CO), 4.25 (q, *J* = 7.1 Hz, 2H, OCH_2_), 3.81 (s, 3H, OCH_3_), 1.29 (t, *J* = 7.1 Hz, 3H, CH_3_). ^13^C NMR (101 MHz, CDCl_3_) δ 182.8, 166.9, 158.2, 156.8, 144.3, 124.9, 118.0, 111.2, 109.6, 62.2, 56.0, 41.3, 14.1. HRMS *m*/*z*: 264.0858 [M + H]^+^, calcd for C_13_H_14_NO_5_: 264.0866.

##### Ethyl 2-(5-nitro-2,3-dioxoindolin-1-yl)acetate (**7g**)

Yellow powder, 62% yield, m.p. 128.7–129.8 °C. ^1^H NMR (400 MHz, CDCl_3_) δ 8.58–8.48 (m, 2H, Ar-H^4^, Ar-H^6^), 7.00 (d, *J* = 8.7 Hz, 1H, Ar-H^7^), 4.60 (s, 2H, N-CH_2_-CO), 4.28 (q, *J* = 7.1 Hz, 2H, OCH_2_), 1.32 (t, *J* = 7.1 Hz, 3H, CH_3_). ^13^C NMR (101 MHz, CDCl_3_) δ 180.5, 166.1, 157.6, 154.4, 144.4, 133.6, 121.1, 117.3, 110.8, 62.7, 41.6, 14.1. HRMS *m*/*z*: 279.0605 [M + H]^+^, calcd for C_12_H_11_N_2_O_6_: 279.0612.

#### 4.1.17. Method for the Synthesis of Intermediate **8a**–**g**

To a solution of compounds **7a**–**g** (300 mg) in EtOH (400 μL), Et_2_NH (1.2 eq) and 3,4,5-Trimethoxyphenylethanone (1.2 eq) were added. The reaction mixture was stirred at room temperature until the complete disappearance of **7a**–**g** was evidenced by TLC. The solvent was removed under vacuum, 5 mL of water was added to the residue, and then the mixture was extracted with ethyl acetate (3 × 10 mL). The organic fractions were combined, dried over Na_2_SO_4_, filtered and concentrated. The crude product was purified by column chromatography (silica gel, petroleum ether/ethyl acetate = 1:1).

##### Ethyl 2-(5-fluoro-3-hydroxy-2-oxo-3-(2-oxo-2-(3,4,5-trimethoxyphenyl)ethyl)indolin-1-yl)acetate (**8a**)

White powder, 61% yield, m.p. 138.6–139.3 °C. ^1^H NMR (400 MHz, CDCl_3_) δ 7.23 (dd, *J* = 7.7, 2.6 Hz, 1H, Ar-H^4^), 7.18 (s, 2H, Ar-H^2′^, Ar-H^6′^), 7.01 (td, *J* = 8.8, 2.6 Hz, 1H, Ar-H^6^), 6.70 (dd, *J* = 8.6, 4.0 Hz, 1H, Ar-H^7^), 4.56 (d, *J* = 17.7 Hz, 1H, N-CH_2_-CO), 4.41 (d, *J* = 17.7 Hz, 1H, N-CH_2_-CO), 4.24 (q, *J* = 7.1 Hz, 2H, OCH_2_), 3.91 (d, *J* = 9.3 Hz, 9H, (OCH_3_)_3_), 3.81 (d, *J* = 17.3 Hz, 1H, CO-CH_2_-C), 3.50 (d, *J* = 17.3 Hz, 1H, CO-CH_2_-C), 1.30 (t, *J* = 7.1 Hz, 3H, CH_3_). ^13^C NMR (101 MHz, CDCl_3_) δ 197.2, 175.9, 167.5, 159.6 (d, *J* = 242.5 Hz), 153.1, 143.5, 138.1 (d, *J* = 2.2 Hz), 131.5 (d, *J* = 7.7 Hz), 131.3, 116.1 (d, *J* = 23.8 Hz), 112.7 (d, *J* = 24.9 Hz), 109.3 (d, *J* = 8.1 Hz), 105.8, 74.8, 62.0, 61.0, 56.3, 43.9, 41.5, 14.1. HRMS *m*/*z*: 462.1540 [M + H]^+^, calcd for C_23_H_25_FNO_8_: 462.1559.

##### Ethyl 2-(5-chloro-3-hydroxy-2-oxo-3-(2-oxo-2-(3,4,5-trimethoxyphenyl)ethyl)indolin-1-yl)acetate (**8b**)

White powder, 71% yield, m.p. 102.5–103.2 °C. ^1^H NMR (400 MHz, CDCl_3_) δ 7.44 (d, *J* = 2.1 Hz, 1H, Ar-H^4^), 7.29 (d, *J* = 3.3 Hz, 1H, Ar-H^6^), 7.17 (s, 2H, Ar-H^2′^, Ar-H^6′^), 6.70 (d, *J* = 8.4 Hz, 1H, Ar-H^7^), 4.54 (d, *J* = 17.7 Hz, 1H, N-CH_2_-CO), 4.42 (d, *J* = 17.6 Hz, 1H, N-CH_2_-CO), 4.24 (q, *J* = 7.1 Hz, 2H, OCH_2_), 3.91 (d, *J* = 9.6 Hz, 9H, (OCH_3_)_3_), 3.82 (d, *J* = 17.4 Hz, 1H, CO-CH_2_-C), 3.53 (d, *J* = 17.3 Hz, 1H, CO-CH_2_-C), 1.30 (t, *J* = 7.1 Hz, 3H, CH_3_). ^13^C NMR (101 MHz, CDCl_3_) δ 197.0, 175.8, 167.5, 153.1, 143.4, 140.9, 131.6, 131.2, 129.8, 128.8, 125.0, 109.7, 105.8, 74.6, 62.1, 61.0, 56.3, 44.0, 41.5, 14.1. HRMS *m*/*z*: 478.1247 [M + H]^+^, calcd for C_23_H_25_ClNO_8_: 478.1263.

##### Ethyl 2-(5-bromo-3-hydroxy-2-oxo-3-(2-oxo-2-(3,4,5-trimethoxyphenyl)ethyl)indolin-1-yl)acetate (**8c**)

White powder, 69% yield, m.p. 96.9–98.0 °C. ^1^H NMR (400 MHz, CDCl_3_) δ 7.58 (s, 1H, Ar-H^4^), 7.44 (d, *J* = 8.4 Hz, 1H, Ar-H^6^), 7.18 (s, 2H, Ar-H^2′^, Ar-H^6′^), 6.66 (d, *J* = 8.3 Hz, 1H, Ar-H^7^), 4.88 (s, 1H, OH), 4.54 (d, *J* = 17.6 Hz, 1H, N-CH_2_-CO), 4.42 (d, *J* = 17.6 Hz, 1H, N-CH_2_-CO), 4.24 (q, *J* = 7.1 Hz, 2H, OCH_2_), 3.92 (d, *J* = 8.9 Hz, 9H, (OCH_3_)_3_), 3.81 (d, *J* = 17.3 Hz, 1H, CO-CH_2_-C), 3.52 (d, *J* = 17.3 Hz, 1H, CO-CH_2_-C), 1.30 (t, *J* = 7.1 Hz, 3H, CH_3_). ^13^C NMR (101 MHz, CDCl_3_) δ 197.0, 175.7, 167.4, 153.1, 143.5, 141.4, 132.7, 131.9, 131.2, 127.7, 116.1, 110.2, 105.8, 74.5, 62.1, 61.0, 56.3, 44.0, 41.4, 14.1. HRMS *m*/*z*: 522.0741 [M + H]^+^, calcd for C_23_H_25_BrNO_8_: 522.0758.

##### Ethyl 2-(3-hydroxy-5-iodo-2-oxo-3-(2-oxo-2-(3,4,5-trimethoxyphenyl)ethyl)indolin-1-yl)acetate (**8d**)

White powder, 31% yield, m.p. 111.4–112.7 °C. ^1^H NMR (400 MHz, CDCl_3_) δ 7.73 (d, *J* = 1.7 Hz, 1H, Ar-H^4^), 7.62 (dd, *J* = 8.2, 1.7 Hz, 1H, Ar-H^6^), 7.17 (s, 2H, Ar-H^2′^, Ar-H^6′^), 6.56 (d, *J* = 8.2 Hz, 1H, Ar-H^7^), 4.87 (s, 1H, OH), 4.52 (d, *J* = 17.6 Hz, 1H, N-CH_2_-CO), 4.41 (d, *J* = 17.7 Hz, 1H, N-CH_2_-CO), 4.23 (q, *J* = 7.2 Hz, 2H, OCH_2_), 3.91 (d, *J* = 9.7 Hz, 9H, (OCH_3_)_3_), 3.80 (d, *J* = 17.3 Hz, 1H, CO-CH_2_-C), 3.53 (d, *J* = 17.4 Hz, 1H, CO-CH_2_-C), 1.30 (t, *J* = 7.2 Hz, 3H, CH_3_). ^13^C NMR (101 MHz, CDCl_3_) δ 197.0, 175.5, 167.4, 153.1, 143.4, 142.1, 138.7, 133.2, 132.2, 131.3, 110.7, 105.8, 86.0, 74.4, 62.1, 61.0, 56.3, 44.0, 41.4, 14.1. HRMS *m*/*z*: 570.0600 [M + H]^+^, calcd for C_23_H_25_INO_8_: 570.0619.

##### Ethyl 2-(3-hydroxy-5-methyl-2-oxo-3-(2-oxo-2-(3,4,5-trimethoxyphenyl)ethyl)indolin-1-yl)acetate (**8e**)

White powder, 29% yield, m.p. 131.7–132.2 °C. ^1^H NMR (400 MHz, CDCl_3_) δ 7.27 (d, *J* = 4.8 Hz, 1H, Ar-H^4^), 7.18 (s, 2H, Ar-H^2′^, Ar-H^6′^), 7.11 (d, *J* = 8.7 Hz, 1H, Ar-H^6^), 6.66 (d, *J* = 7.9 Hz, 1H, Ar-H^7^), 4.55 (d, *J* = 17.6 Hz, 1H, N-CH_2_-CO), 4.38 (d, *J* = 17.6 Hz, 1H, N-CH_2_-CO), 4.22 (q, *J* = 7.2 Hz, 2H, OCH_2_), 3.90 (d, *J* = 10.4 Hz, 9H, (OCH_3_)_3_), 3.79 (d, *J* = 17.1 Hz, 1H, CO-CH_2_-C), 3.49 (d, *J* = 17.1 Hz, 1H, CO-CH_2_-C), 2.31 (s, 3H, Ar-CH_3_), 1.29 (t, *J* = 7.1 Hz, 3H, CH_3_). ^13^C NMR (101 MHz, CDCl_3_) δ 197.6, 176.2, 167.7, 153.1, 143.2, 139.8, 133.1, 131.6, 130.2, 129.9, 125.1, 108.3, 105.8, 74.9, 61.8, 61.0, 56.3, 43.8, 41.4, 21.1, 14.1. HRMS *m*/*z*: 458.1796 [M + H]^+^, calcd for C_24_H_28_NO_8_: 458.1809.

##### Ethyl 2-(3-hydroxy-5-methoxy-2-oxo-3-(2-oxo-2-(3,4,5-trimethoxyphenyl)ethyl)indolin-1-yl)acetate (**8f**)

White powder, 34% yield, m.p. 131.7–132.9 °C. ^1^H NMR (400 MHz, CDCl_3_) δ 7.18 (s, 2H, Ar-H^2′^, Ar-H^6′^), 7.08 (d, *J* = 2.6 Hz, 1H, Ar-H^4^), 6.83 (dd, *J* = 8.5, 2.6 Hz, 1H, Ar-H^6^), 6.68 (d, *J* = 8.5 Hz, 1H, Ar-H^7^), 4.55 (d, *J* = 17.6 Hz, 1H, N-CH_2_-CO), 4.37 (d, *J* = 17.5 Hz, 1H, N-CH_2_-CO), 4.23 (q, *J* = 7.1 Hz, 2H, OCH_2_), 3.90 (d, *J* = 10.8 Hz, 9H, (OCH_3_)_3_), 3.76 (d, *J* = 17.1 Hz, 4H, CO-CH_2_-C, Ar-OCH_3_), 3.48 (d, *J* = 17.1 Hz, 1H, CO-CH_2_-C), 1.29 (t, *J* = 7.1 Hz, 3H, CH_3_). ^13^C NMR (101 MHz, CDCl_3_) δ 197.5, 176.1, 167.7, 156.5, 153.1, 143.3, 135.5, 131.5, 131.1, 114.3, 111.7, 109.1, 105.8, 75.1, 61.9, 61.0, 56.3, 55.8, 43.8, 41.5, 14.1. HRMS *m*/*z*: 474.1741 [M + H]^+^, calcd for C_24_H_28_NO_9_: 474.1759.

##### Ethyl 2-(3-hydroxy-5-nitro-2-oxo-3-(2-oxo-2-(3,4,5-trimethoxyphenyl)ethyl)indolin-1-yl)acetate (**8g**)

White powder, 73% yield, m.p. 156.4–157.6 °C. ^1^H NMR (400 MHz, CDCl_3_) δ 8.30 (d, *J* = 2.2 Hz, 1H, Ar-H^4^), 8.26 (d, *J* = 8.6 Hz, 1H, Ar-H^6^), 7.14 (s, 2H, Ar-H^2′^, Ar-H^6′^), 6.86 (d, *J* = 8.6 Hz, 1H, Ar-H^7^), 4.60 (d, *J* = 17.8 Hz, 2H, N-CH_2_-CO), 4.27 (q, *J* = 7.1 Hz, 2H, OCH_2_), 3.95 (d, *J* = 17.6 Hz, 1H, CO-CH_2_-C), 3.90 (d, *J* = 9.0 Hz, 9H, (OCH_3_)_3_), 3.70 (d, *J* = 17.6 Hz, 1H, CO-CH_2_-C), 1.32 (t, *J* = 7.1 Hz, 3H, CH_3_). ^13^C NMR (101 MHz, CDCl_3_) δ 196.2, 176.3, 167.2, 153.1, 148.2, 143.9, 143.5, 130.9, 130.8, 126.9, 120.1, 108.5, 105.8, 73.8, 62.4, 61.0, 56.3, 44.7, 41.6, 14.1. HRMS *m*/*z*: 489.1487 [M + H]^+^, calcd for C_23_H_25_N_2_O_10_: 489.1504.

#### 4.1.18. Method for the Synthesis of Target Compounds **9a**–**g**

A mixture of the corresponding intermediate of **8a**–**g** (200 mg) and HCl (aq, conc, 400 μL) in EtOH (2 mL) was stirred at reflux until its disappearance was evidenced by TLC. After the reaction mixture was cooled to room temperature, the resulting solid product was filtered. The solid products were purified by silica gel column chromatography (silica gel, petroleum ether/ethyl acetate = 3:1).

##### Ethyl (*E*)-2-(5-fluoro-2-oxo-3-(2-oxo-2-(3,4,5-trimethoxyphenyl)ethylidene)indolin-1-yl)acetate (**9a**)

Orange powder, 75% yield, m.p. 147.8–148.2 °C. ^1^H NMR (400 MHz, CDCl_3_) δ 8.15 (dd, *J* = 9.0, 2.7 Hz, 1H, Ar-H^4^), 7.95 (s, 1H, Ar-CO-CH=C), 7.36 (s, 2H, Ar-H^2′^, Ar-H^6′^), 7.09 (td, *J* = 8.6, 2.7 Hz, 1H, Ar-H^6^), 6.66 (dd, *J* = 8.6, 4.1 Hz, 1H, Ar-H^7^), 4.53 (s, 2H, N-CH_2_-CO), 4.26 (q, *J* = 7.1 Hz, 2H, OCH_2_), 3.97 (d, *J* = 2.1 Hz, 9H, 3′, 4′, 5′-(OCH_3_)_3_), 1.30 (t, *J* = 7.2 Hz, 3H, CH_3_). ^13^C NMR (101 MHz, CDCl_3_) δ 189.4, 167.8, 167.3, 159.1 (d, *J* = 240.3 Hz), 153.4, 143.7, 140.8, 135.6 (d, *J* = 3.3 Hz), 132.6, 128.0, 121.1 (d, *J* = 9.9 Hz), 118.9 (d, *J* = 24.2 Hz), 115.5 (d, *J* = 26.8 Hz), 108.8 (d, *J* = 8.1 Hz), 106.4, 62.0, 61.1, 56.5, 41.6, 14.1. HRMS *m*/*z*: 444.1446 [M + H]^+^, calcd for C_23_H_23_FNO_7_: 444.1453.

##### Ethyl (*E*)-2-(5-chloro-2-oxo-3-(2-oxo-2-(3,4,5-trimethoxyphenyl)ethylidene)indolin-1-yl)acetate (**9b**)

Orange powder, 57% yield, m.p. 166.5–167.1 °C. ^1^H NMR (400 MHz, CDCl_3_) δ 8.38 (s, 1H, Ar-H^4^), 7.93 (s, 1H, Ar-CO-CH=C), 7.36 (m, 3H, Ar-H^2′^, Ar-H^6′^, Ar-H^6^), 6.66 (d, *J* = 8.3 Hz, 1H, Ar-H^7^), 4.52 (s, 2H, N-CH_2_-CO), 4.25 (q, *J* = 7.2 Hz, 2H, OCH_2_), 3.97 (s, 9H, 3′, 4′, 5′-(OCH_3_)_3_), 1.29 (t, *J* = 7.1 Hz, 3H, CH_3_). ^13^C NMR (101 MHz, CDCl_3_) δ 189.3, 167.6, 167.1, 153.4, 143.7, 143.1, 135.1, 132.6, 132.2, 128.6, 128.1, 128.0, 121.3, 109.3, 106.4, 77.4, 77.1, 76.7, 62.0, 61.1, 56.5, 41.5, 14.1. HRMS *m*/*z*: 460.1149 [M + H]^+^, calcd for C_23_H_23_ClNO_7_: 460.1158.

##### Ethyl (*E*)-2-(5-bromo-2-oxo-3-(2-oxo-2-(3,4,5-trimethoxyphenyl)ethylidene)indolin-1-yl)acetate (**9c**)

Orange powder, 75% yield, m.p. 171.6–172.4 °C. ^1^H NMR (400 MHz, CDCl_3_) δ 8.52 (s, 1H, Ar-H^4^), 7.93 (s, 1H, Ar-CO-CH=C), 7.49 (dd, *J* = 8.3, 2.0 Hz, 1H, Ar-H^6^), 7.36 (s, 2H, Ar-H^2′^, Ar-H^6′^), 6.62 (d, *J* = 8.3 Hz, 1H, Ar-H^7^), 4.52 (s, 2H, N-CH_2_-CO), 4.25 (q, *J* = 7.1 Hz, 2H, OCH_2_), 3.97 (s, 9H, 3′, 4′, 5′-(OCH_3_)_3_), 1.30 (t, *J* = 7.1 Hz, 3H, CH_3_). ^13^C NMR (101 MHz, CDCl_3_) δ 189.2, 167.4, 167.1, 153.4, 143.7, 143.6, 135.0, 134.9, 132.6, 130.7, 128.1, 121.7, 115.9, 109.7, 106.4, 62.0, 61.1, 56.5, 41.5, 14.1. HRMS *m*/*z*: 504.0643 [M + H]^+^, calcd for C_23_H_23_BrNO_7_: 504.0652.

##### Ethyl (*E*)-2-(5-iodo-2-oxo-3-(2-oxo-2-(3,4,5-trimethoxyphenyl)ethylidene)indolin-1-yl)acetate (**9d**)

Orange powder, 60% yield, m.p. 172.0–173.3 °C. ^1^H NMR (400 MHz, CDCl_3_) δ 8.68 (s, 1H, Ar-H^4^), 7.91 (s, 1H, Ar-CO-CH=C), 7.69 (d, *J* = 8.3 Hz, 1H, Ar-H^6^), 7.36 (s, 2H, Ar-H^2′^, Ar-H^6′^), 6.52 (d, *J* = 8.2 Hz, 1H, Ar-H^7^), 4.52 (s, 2H, N-CH_2_-CO), 4.25 (q, *J* = 7.0 Hz, 2H, OCH_2_), 3.97 (s, 9H, 3′, 4′, 5′-(OCH_3_)_3_), 1.30 (t, *J* = 7.1 Hz, 3H, CH_3_). ^13^C NMR (101 MHz, CDCl_3_) δ 189.3, 167.3, 167.1, 153.4, 144.2, 143.7, 141.0, 136.3, 134.7, 132.6, 128.0, 122.1, 110.3, 106.4, 85.8, 62.1, 61.1, 56.5, 41.5, 14.2. HRMS *m*/*z*: 552.0504 [M + H]^+^, calcd for C_23_H_23_INO_7_: 552.0514.

##### Ethyl (*E*)-2-(5-methyl-2-oxo-3-(2-oxo-2-(3,4,5-trimethoxyphenyl)ethylidene)indolin-1-yl)acetate (**9e**)

Orange powder, 62% yield, m.p. 157.4–158.9 °C. ^1^H NMR (400 MHz, CDCl_3_) δ 8.14 (s, 1H, Ar-H^4^), 7.86 (s, 1H, Ar-CO-CH=C), 7.36 (s, 2H, Ar-H^2′^, Ar-H^6′^), 7.17 (d, *J* = 7.9 Hz, 1H, Ar-H^6^), 6.61 (d, *J* = 8.0 Hz, 1H, Ar-H^7^), 4.51 (s, 2H, N-CH_2_-CO), 4.25 (q, *J* = 7.1 Hz, 2H, OCH_2_), 3.96 (s, 9H, 3′, 4′, 5′-(OCH_3_)_3_), 2.34 (s, 3H, Ar-CH_3_), 1.29 (t, *J* = 7.2 Hz, 3H, CH_3_). ^13^C NMR (101 MHz, CDCl_3_) δ 189.9, 168.0, 167.5, 153.3, 143.4, 142.5, 136.2, 133.0, 132.9, 132.7, 128.4, 126.5, 120.1, 108.0, 106.3, 61.9, 61.0, 56.4, 41.5, 21.1, 14.1. HRMS *m*/*z*: 440.1695 [M + H]^+^, calcd for C_24_H_26_NO_7_: 440.1704.

##### Ethyl (*E*)-2-(5-methoxy-2-oxo-3-(2-oxo-2-(3,4,5-trimethoxyphenyl)ethylidene)indolin-1-yl)acetate (**9f**)

Brown powder, 69% yield, m.p. 157.9–158.2 °C. ^1^H NMR (400 MHz, CDCl_3_) δ 8.00 (d, *J* = 2.6 Hz, 1H, Ar-H^4^), 7.88 (s, 1H, Ar-CO-CH=C), 7.36 (s, 2H, Ar-H^2′^, Ar-H^6′^), 6.92 (dd, *J* = 8.6, 2.6 Hz, 1H, Ar-H^6^), 6.63 (d, *J* = 8.5 Hz, 1H, Ar-H^7^), 4.50 (s, 2H, N-CH_2_-CO), 4.25 (q, *J* = 7.1 Hz, 2H, OCH_2_), 3.97 (s, 9H, 3′, 4′, 5′-(OCH_3_)_3_), 3.82 (s, 3H, 5-OCH_3_), 1.29 (t, *J* = 7.1 Hz, 3H, CH_3_). ^13^C NMR (101 MHz, CDCl_3_) δ 189.7, 167.9, 167.5, 156.0, 153.3, 143.4, 138.6, 136.4, 132.9, 126.9, 120.8, 118.5, 113.7, 108.8, 106.3, 61.9, 61.0, 56.5, 55.9, 41.6, 14.1. HRMS *m*/*z*: 456.1642 [M + H]^+^, calcd for C_24_H_26_NO_8_: 456.1653.

##### Ethyl (*E*)-2-(5-nitro-2-oxo-3-(2-oxo-2-(3,4,5-trimethoxyphenyl)ethylidene)indolin-1-yl)acetate (**9g**)

Orange powder, 72% yield, m.p. 179.5–180.8 °C. ^1^H NMR (400 MHz, CDCl_3_) δ 9.29 (d, *J* = 2.3 Hz, 1H, Ar-H^4^), 8.33 (dd, *J* = 8.7, 2.3 Hz, 1H, Ar-H^6^), 8.04 (s, 1H, Ar-CO-CH=C), 7.38 (s, 2H, Ar-H^2′^, Ar-H^6′^), 6.85 (d, *J* = 8.7 Hz, 1H, Ar-H^7^), 4.61 (s, 2H, N-CH_2_-CO), 4.28 (q, *J* = 7.1 Hz, 2H, OCH_2_), 3.98 (s, 9H, 3′, 4′, 5′-(OCH_3_)_3_), 1.32 (t, *J* = 7.1 Hz, 3H, CH_3_). ^13^C NMR (101 MHz, CDCl_3_) δ 188.7, 167.9, 166.7, 153.4, 149.2, 144.0, 143.8, 133.9, 132.3, 129.8, 128.5, 123.7, 120.4, 108.2, 106.6, 62.3, 61.1, 56.5, 41.7, 14.1. HRMS *m*/*z*: 471.1391 [M + H]^+^, calcd for C_23_H_23_N_2_O_9_: 471.1398.

### 4.2. Biomaterial and Experimental Apparatus

All reagents were purchased from companies such as Thermo Fisher Scientific (MA, USA), Pricella (Wuhan, China) and Sigma (VT, USA). The NO detection kit was purchased from Beyotime (Shanghai, China). ELISA kits for mouse TNF-α and IL-6 were bought from ABclonal Technology Co., Ltd. (Wuhan, China). Centrifuge (BY-G10 medical centrifuge, Beijing Baiyang Medical Instrument Co., Ltd., Beijing, China), CO_2_ incubator (Thermo Forma, Thermo Fisher Scientific, MA, USA), inverted microscope (XD101, Nanjing Jiangnan Photoelectric Co., Ltd., Nanjing, China), microplate reader (Benchmark Plus, Bio-Rad, CA, USA), autoclave (MLS-3750, SANYO, Osaka, Japan), clean bench (Antai Company, Sujing Group, Suzhou, China), water bath (Tianjin Huabei Instrument Co., Ltd., Tianjin, China) and adjustable pipette (Thermo Fisher Scientific, MA, USA).

### 4.3. Cell Culture

BV2 microglial cells (SCSP-5208, Cell Bank, Chinese Academy of Sciences, Shanghai, China) were incubated in high-glucose DMEM supplemented with 10% fetal bovine serum and 1% penicillin and streptomycin in a humidified atmosphere of 5% CO_2_ at 37 °C.

### 4.4. Experiment on BV2 Cells

#### 4.4.1. Cell Viability Assay

BV2 cells were plated in 96-well microplates at 20,000 cells per well (100 μL/well) and incubated for 24 h. Then, 100 μL of compounds (diluted with medium to a final concentration of 1.56–100 μM) was added, and each concentration had 3 parallel wells. After 24 h, the cell viability was measured by MTS assay. The absorbance was detected at 490 nm. The percentage of viability was obtained from the following formula: OD_drug-treated_/OD_normal cells_ × 100. The IC_50_ values of test compounds were calculated by linear regression plots.

#### 4.4.2. Determination of NO Inhibitory Activity

BV2 cells were plated in 96-well microplates at 40,000 cells per well (100 μL/well) and incubated for 24 h. Then, 50 μL of LPS solution with a final concentration of 100 ng/mL and 50 μL of compounds (diluted with medium to a final concentration of 0.31–20 μM) were added, and each concentration had 3 parallel wells. The NO assay kit was used to carry out nitrite assays after 24 h. The 50 μL of culture medium was mixed with the GrisR_1_ (50 μL) and GrisR_2_ (50 μL) of the NO assay kit in another new 96-well plate. The absorbance was detected at 540 nm. The percentage of inhibition of NO was obtained from the following formula: [(R_lps_ − R_B_ − R_C_)/(R_lps_ − R_B_)] × 100, where R_lps_ is the release amount of only the LPS-treated group, R_B_ is the release amount of the normal control and R_C_ is the release amount of the test compound and LPS-treated group. The IC_50_ values of test compounds were calculated by linear regression plots.

### 4.5. Enzyme-Linked Immunosorbent Assay (ELISA)

BV2 cells were plated in 96-well microplates at 40,000 cells per well (100 μL/well) and incubated for 24 h. Then, 50 μL of LPS solution with a final concentration of 100 ng/mL and 50 μL of compounds (diluted with medium to a final concentration of 0.75–40 μM) were added, and each concentration had 3 parallel wells. The supernatant was collected after 24 h. Then, according to the manufacturer’s instructions, the ELISA assay kit was used to measure the concentrations of IL-6 and TNF-α. The absorbance was detected at 450 nm.

### 4.6. DOCKING Studies

The structures of the target proteins were acquired from the Protein Data Bank (PDB, https://www.rcsb.org/) accessed on 4 September 2024. PDB codes are IRAK 4, JNK, IKK β, TAK 1, p38, PI3K, MD 2, 15-LOX-1, PKC α, PKC θ, JNK 1, JNK 3, COX 1, COX 2, TLR 4, iNOS, KEAP1-NRF 2, PTGR 2 and AKR1C 3; HX 1 protein structures are 6RFJ, 1PMV, 3RZF, 7NTH, 6OHD, 1E7V, 2E56, 1LOX, 4RA4, 5F9E, 1UKI, 3TTI, 4O1Z, 3LN1, 7MLM, 3E6T, 4L7D, 2ZB8, 1S2A and 4C1M. Use Maestro to optimize the energy of small molecule ligands. Remove ligands and water molecules from protein structures and determine hydrogenation and docking positions. In the crystal structure, the center of the box is designated as the geometric center of the ligand. The default parameters of Schrödinger software were used if it was not mentioned. The docking results were visualized by using Pymol 2.3.4 [39].

### 4.7. Statistical Analysis

The data were analyzed by using GraphPad Prism 8.0 (GraphPad, San Diego, CA. USA). Differences between groups were compared through ANOVA multiple comparison. The data are presented as the mean ± standard deviation from three independent experiments. * *p* < 0.05, ** *p* < 0.01, *** *p* < 0.001 and **** *p* < 0.0001.

## Figures and Tables

**Table 1:** The NO release amount of BV2 microglial cells induced by LPS and affected by **4a**–**t** compounds ^a^.

Compound	Log *p* ^b^	Nitrite (μM) ^c^	Compound	Log *p* ^b^	Nitrite (μM) ^c^
Blank	-	-	**4j**	2.62	8.6 ± 0.1
LPS	-	29.4 ± 0.9	**4k**	3.15	15.2 ± 0.2
Isatin	0.83	20.8 ± 0.9	**4l**	3.67	17.5 ± 0.3
**4a**	1.39	22.9 ± 0.5	**4m**	3.80	18.6 ± 0.3
**4b**	3.36	0.69 ± 0.01	**4n**	2.12	16.7 ± 0.3
**4c**	2.99	19.3 ± 0.5	**4o**	4.79	19.0 ± 0.4
**4d**	3.44	12.5 ± 0.2	**4p**	4.17	17.0 ± 0.3
**4e**	3.90	15.2 ± 0.3	**4q**	2.25	20.2 ± 0.4
**4f**	4.50	17.1 ± 0.3	**4r**	2.14	20.5 ± 0.6
**4g**	5.36	8.5 ± 0.1	**4s**	1.82	11.1 ± 0.4
**4h**	3.05	20.6 ± 0.6	**4t**	1.88	16.7 ± 0.2
**4i**	3.42	15.3 ± 0.3			

^a^ Data are presented as mean values. Error bars denote the standard error of at least three independent experiments performed in triplicate. **^b^** Log *p* values were calculated using website of Molinspiration. **^c^** NO production was determined by the Griess test.

**Table 2:** The IC_50_ values and therapeutic indexes of compounds **4b**, **4g** and **4j** for NO inhibition and cell viability.

Compound	NO Inhibition IC_50_ (μM)	Cell Viability IC_50_ (μM)	Therapeutic Index (TI) ^a^
**4b**	1.6	34.6	21.6
**4g**	10.8	30.1	2.8
**4j**	15.7	71.8	4.6

**^a^** The calculation formula is cell viability IC_50_/NO inhibition IC_50_ = therapeutic index.

**Table 3:** The IC_50_ values and therapeutic indexes of compounds **5a**, **5b**, **5c**, **5f** and **5g** for NO inhibition and cell viability.

Compound	NO Inhibition IC_50_ (μM)	Cell Viability IC_50_ (μM)	Therapeutic Index (TI) ^a^	Log *p* ^b^
**5a**	10.9	78.6	7.2	2.25
**5b**	15.8	45.3	2.9	5.20
**5c**	6.3	19.8	3.1	7.22
**5f**	10.7	42.7	4.0	4.05
**5g**	6.0	48.5	8.1	4.57

**^a^** The calculation formula is cell viability IC_50_/NO inhibition IC_50_ = therapeutic index. **^b^** Log *p* values were calculated using Molinspiration software (https://www.molinspiration.com/).

**Table 4:** The IC_50_ values and therapeutic indexes of compounds **9e** and **9f** for NO inhibition and cell viability.

Compound	NO Inhibition IC_50_ (μM)	Cell Viability IC_50_ (μM)	Therapeutic Index (TI) ^a^	Log *p* ^b^
**9e**	10.3	80.6	7.8	3.05
**9f**	10.4	78.1	7.5	2.65

**^a^** The calculation formula is cell viability IC_50_/NO inhibition IC_50_ = therapeutic index. **^b^** Log *p* values were calculated using Molinspiration software.

**Table 5:** The docking scores of compound **4b** with different targets.

Protein	PDB ID	Docking Score (kcal/mol) ^a^	Protein	PDB ID	Docking Score (kcal/mol) ^a^
TLR4/MD2	7MLM	−10.36	IKK β	3RZF	−6.40
p38	6OHD	−9.49	JNK	1PMV	−5.90
MD2	2E56	−9.45	PKC θ	5F9E	−5.89
COX2	3LN1	−8.97	15-LOX−1	1LOX	−5.80
COX1	4O1Z	−8.18	HX1	4C1M	−5.61
JNK3	3TTI	−7.47	TAK1	7NTH	−5.55
JNK1	1UKI	−7.26	KEAP1-NRF2	4L7D	−5.35
IRAK4	6RFJ	−7.21	PKC α	4RA4	−4.50
PTGR2	2ZB8	−6.70	PI3K	1E7V	−4.30
AKR1C3	1S2A	−6.66	iNOS	3E6T	−3.43

**^a^** Molecular docking was performed using the Schrödinger Suites 2018.

## Data Availability

The original contributions presented in this study are included in the article/Supplementary Materials. Further inquiries can be directed to the corresponding author(s).

## Supplementary Materials

The following supporting information can be downloaded at: https://www.mdpi.com/article/10.3390/molecules30071421/s1.

## Abbreviations

LPS, lipopolysaccharide; DMF, *N*,*N*-dimethylformamide; THF, tetrahydrofuran; iNOS, inducible nitric oxide synthase; ROS, reactive oxygen species; SAR, structure–activity relationship; TNF-α, tumor necrosis factor-α; IL-6, Interleukin-6; ANOVA, analysis of variance; ELISA, enzyme-linked immunosorbent assay; NF-κB, nuclear factor-kappa B; MAPK, mitogen-activated protein kinase; IC_50_, half-maximal inhibitory concentration; MTS, 3-(4,5-Dimethylthiazol-2-yl)-5-(3-carboxymethoxyphenyl)-2-(4-sulfophenyl)-2H-tetrazolium; TLC, thin-layer chromatography.
