# Supplementary material for: Novel Isatin–Chalcone Hybrid Molecules: Design, Synthesis and Anti-Neuroinflammatory Activity Evaluation
Source: Molecules. 2025 Mar 22;30(7):1421. doi: 10.3390/molecules30071421 (PMC11990898; doi:10.3390/molecules30071421)
Supplement: Supplementary file 1 [file molecules-30-01421-s001.zip › molecules-3502404-supplementary.pdf]

Novel Isatin-Chalcone Hybrid Molecules: Design, Synthesis and Anti-Neuroinflammatory Activity Evaluation

Rongrong Wang [1,†], Zhili Zhang [2,†], Wei Jiang [1], Junyi Liu [2,3], Chao Tian [2,*], Meng Wang [1,*]

[1] College of Pharmacy, Beihua University, Jilin 132013, China

[2] Department of Chemical Biology, School of Pharmaceutical Sciences, Peking University, Beijing 100191, China

[3] State Key Laboratory of Natural and Biomimetic Drugs, Peking University, Beijing 100191, China

[*] Corresponding author. *E-mail addresses:* tianchao@bjmu.edu.cn (C. Tian), mengjinwang@126.com (M. Wang).

[†] These authors contributed equally to this work.

Contents

1. The molecular docking results of compound **4b**..1

2. Standard curve...3

3. ^{1}H NMR Spectra, ^{13}C NMR Spectra and HRMS Spectra..4

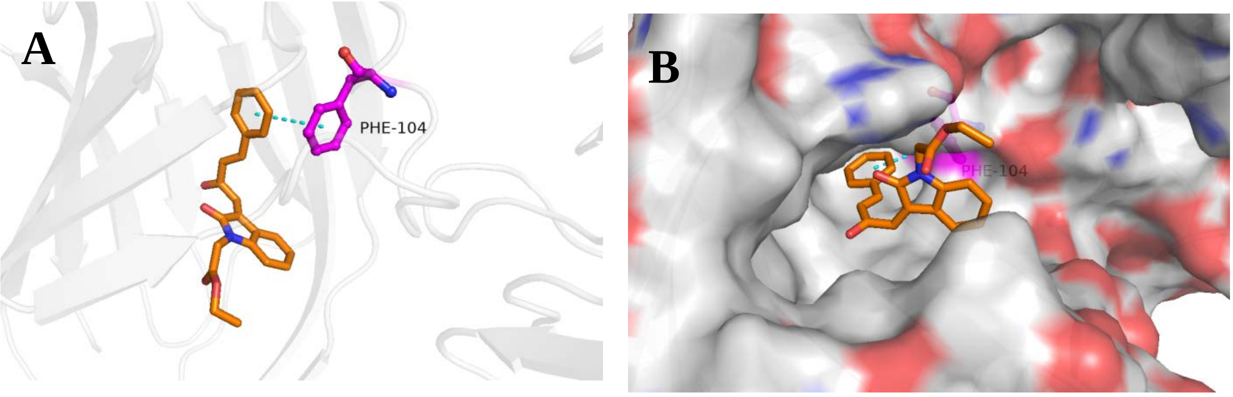

Figure S1. 3D Visual Representation of the Complex of **4b** and TLR4/MD2 (PDB ID: 7MLM). (A) The 3D docking conformation of the ball-and-stick model of **4b** and TLR4/MD2. (B) The 3D conformation of the surface model of **4b** and TLR4/MD2.

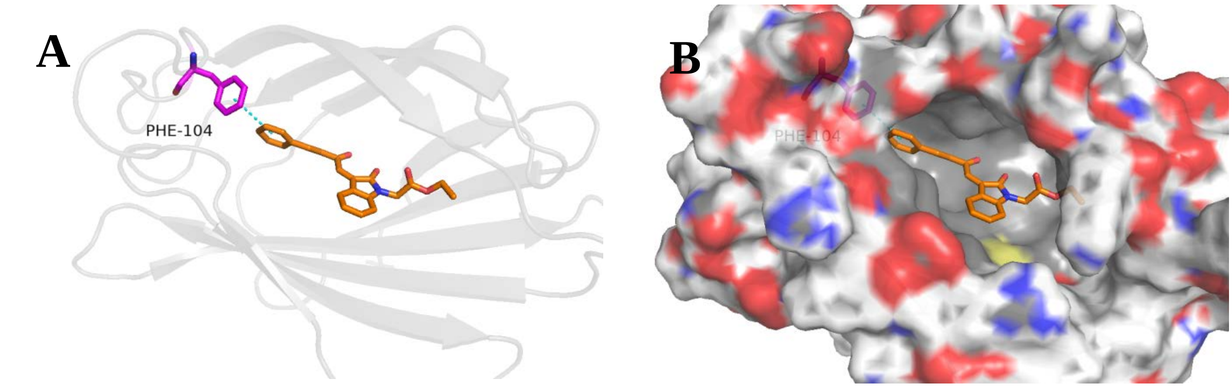

Figure S2. 3D Visual Representation of the Complex of **4b** and MD2 (PDB ID: 2E56). (A) The 3D docking conformation of the ball-and-stick model of **4b** and MD2. (B) The 3D conformation of the surface model of **4b** and MD2.

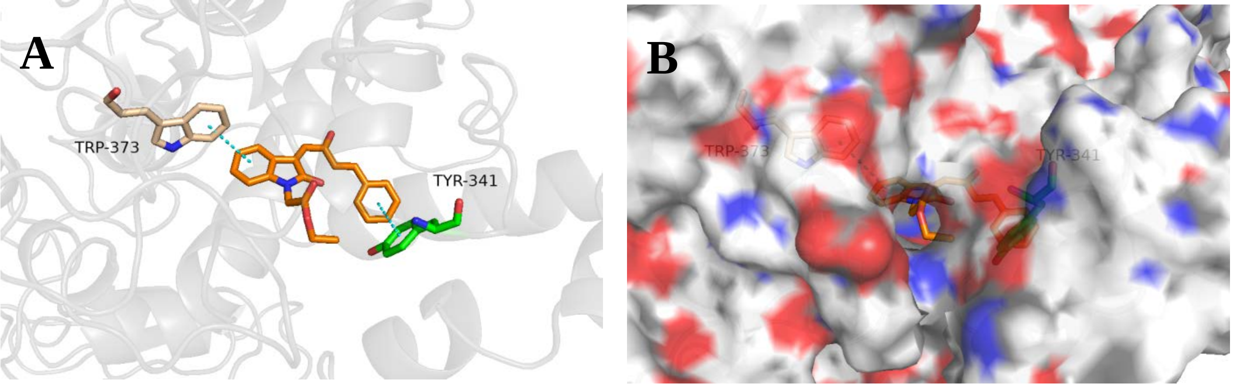

Figure S3. 3D Visual Representation of the Complex of **4b** and COX-2 (PDB ID: 3LN1). (A) The 3D docking conformation of the ball-and-stick model of **4b** and COX-2. (B) The 3D conformation of the surface model of **4b** and COX-2.

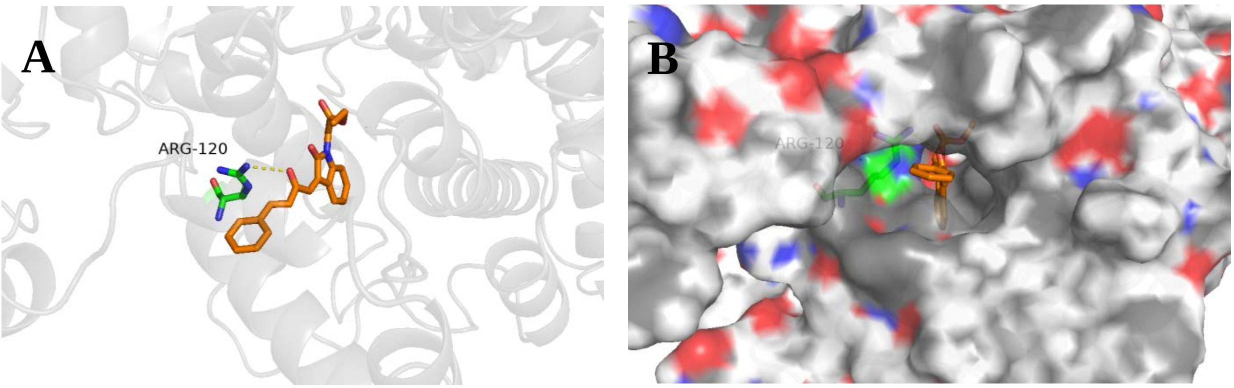

Figure S4. 3D Visual Representation of the Complex of **4b** and COX-1 (PDB ID: 4O1Z). (A) The 3D docking conformation of the ball-and-stick model of **4b** and COX-1. (B) The 3D conformation of the surface model of **4b** and COX-1.

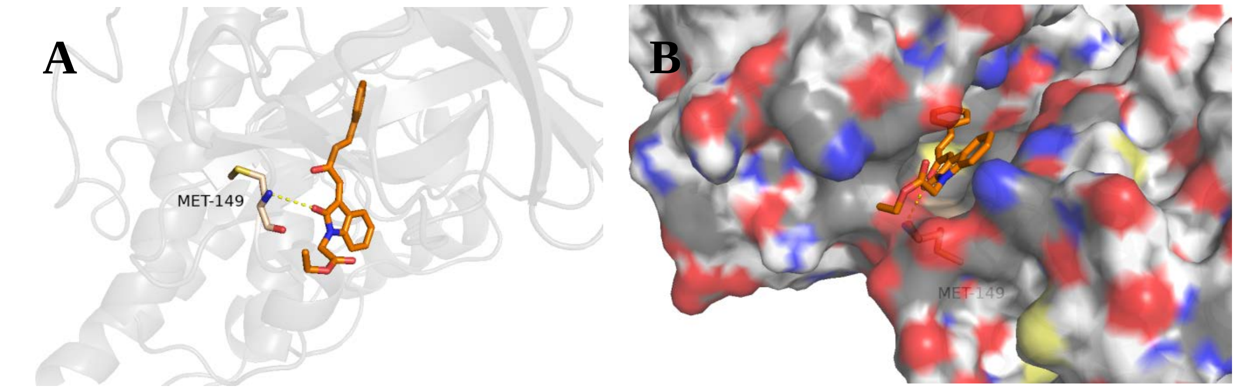

Figure S5. 3D Visual Representation of the Complex of **4b** and JNK3 (PDB ID: 3TTI). (A) The 3D docking conformation of the ball-and-stick model of **4b** and JNK3. (B) The 3D conformation of the surface model of **4b** and JNK3.

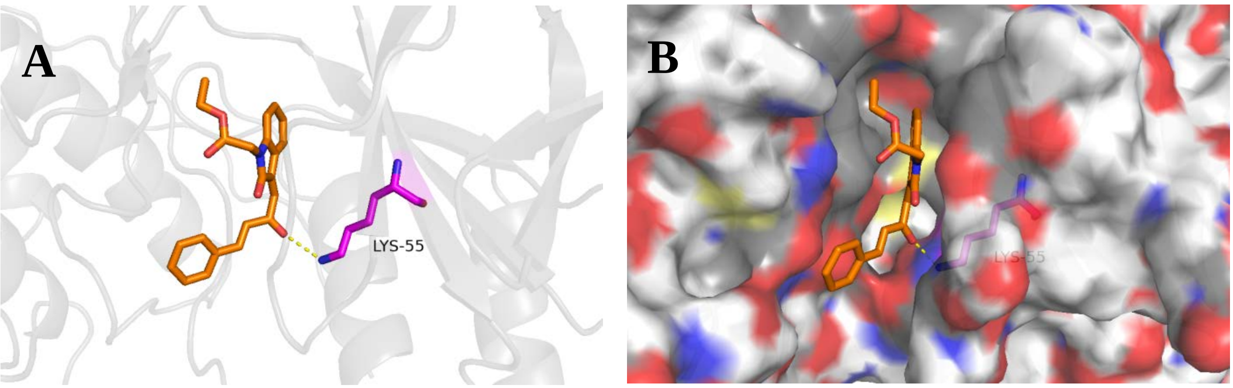

Figure S6. 3D Visual Representation of the Complex of **4b** and JNK1 (PDB ID: 1UKI). (A) The 3D docking conformation of the ball-and-stick model of **4b** and JNK1 . (B) The 3D conformation of the surface model of **4b** and JNK1.

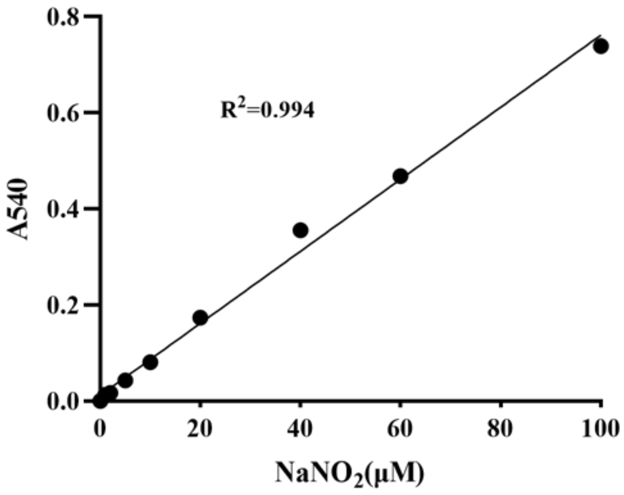

Figure S7. Standard curve of NO

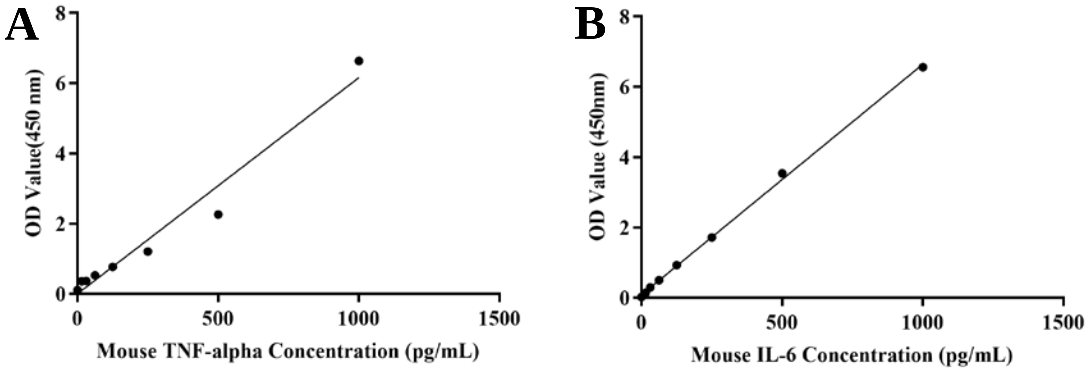

Figure S8. (A) Standard curve of mouse inflammatory factor TNF-α. (B) Standard curve of mouse inflammatory factor IL-6.

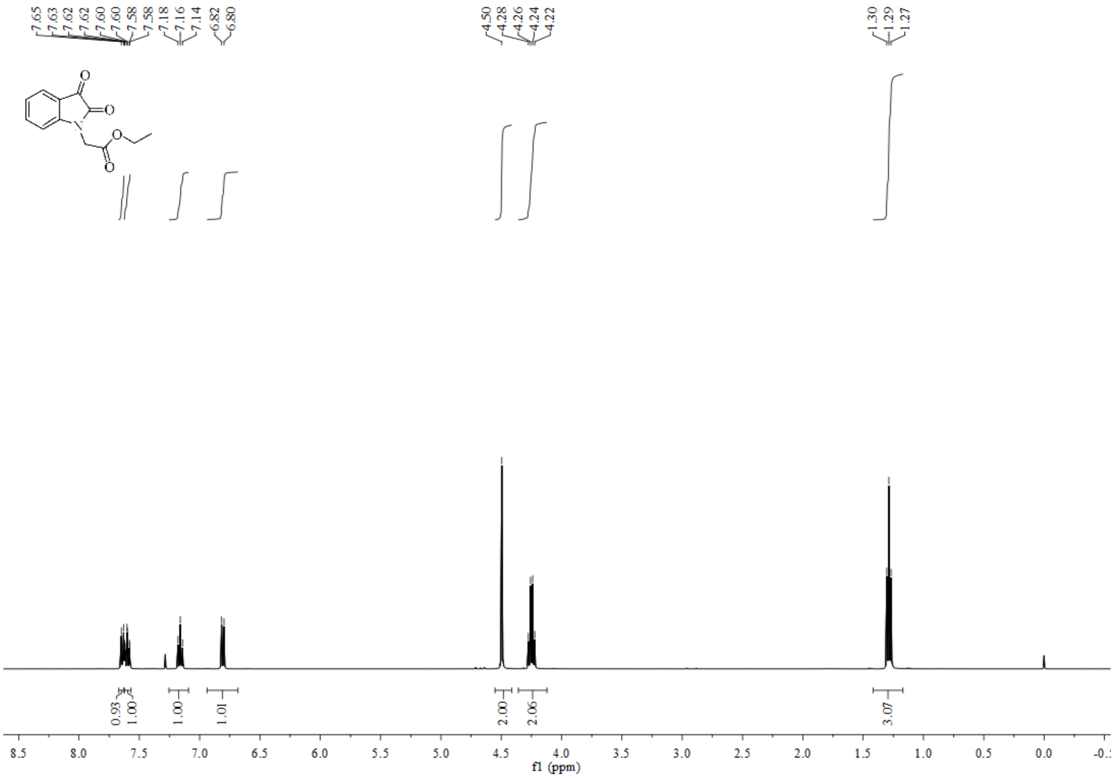

Figure S9. ^{1}H NMR Spectrum of compound **2**

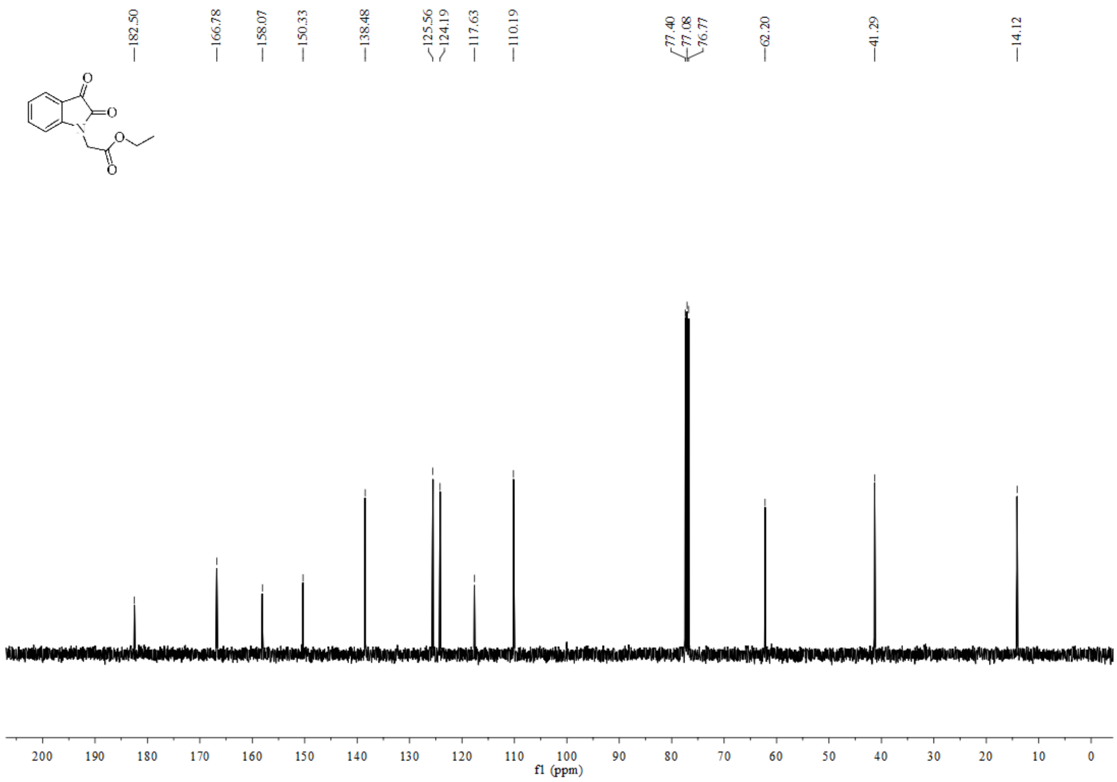

Figure S10. ^{13}C NMR Spectrum of compound **2**

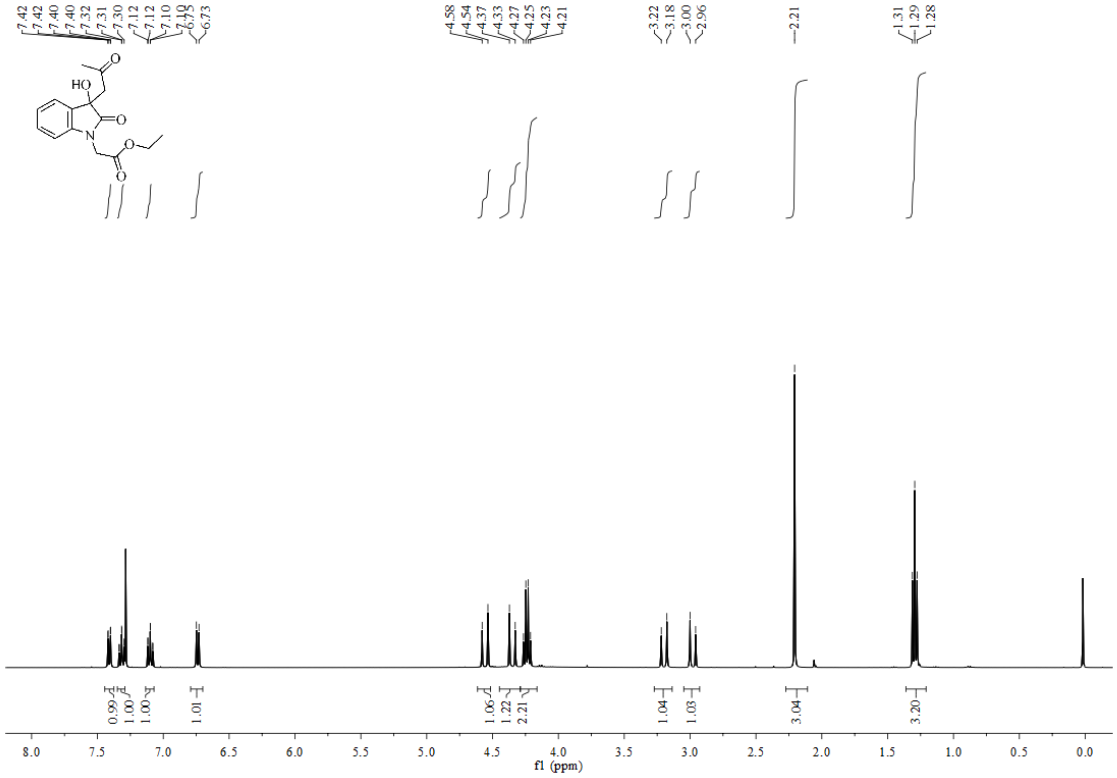

Figure S11. ^{1}H NMR Spectrum of compound **3a**

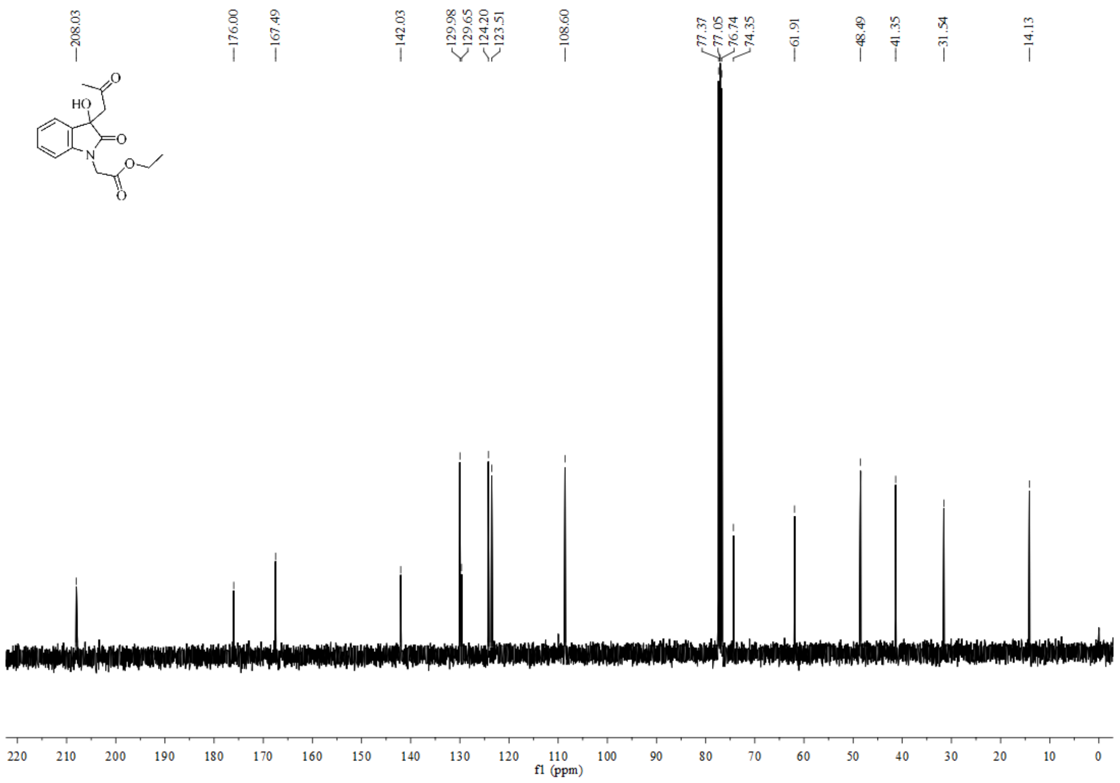

Figure S12. ^{13}C NMR Spectrum of compound **3a**

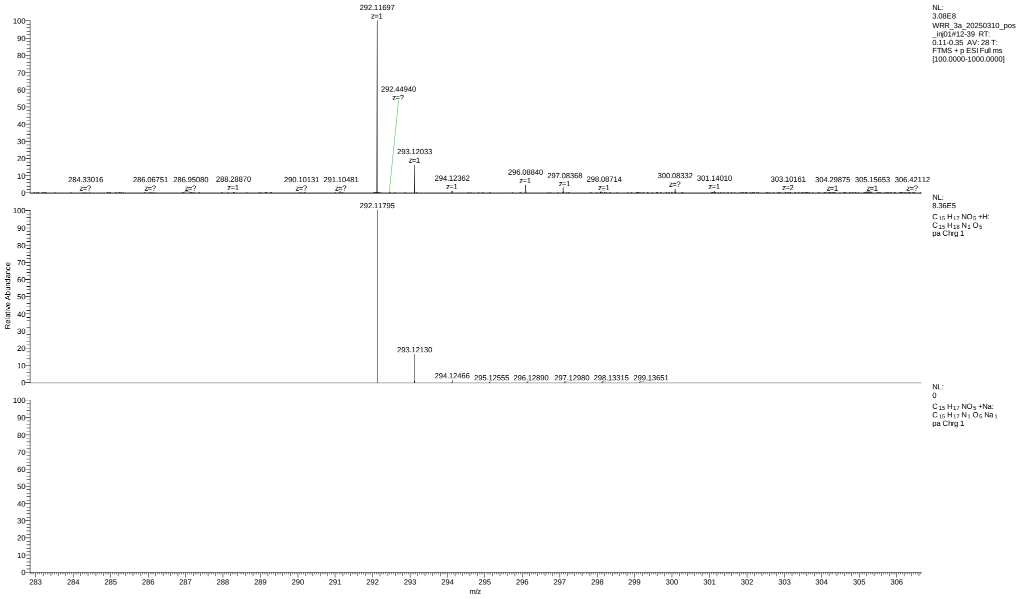

Figure S13. HRMS Spectrum of compound **3a**

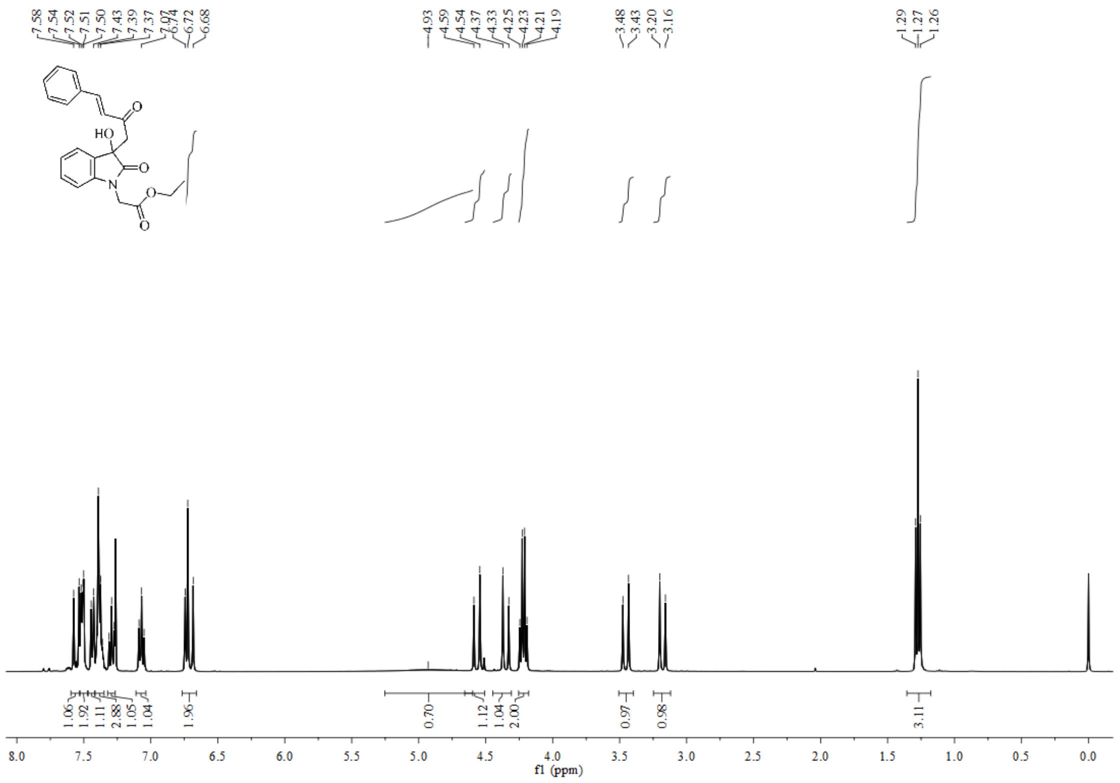

Figure S14. ^{1}H NMR Spectrum of compound **3b**

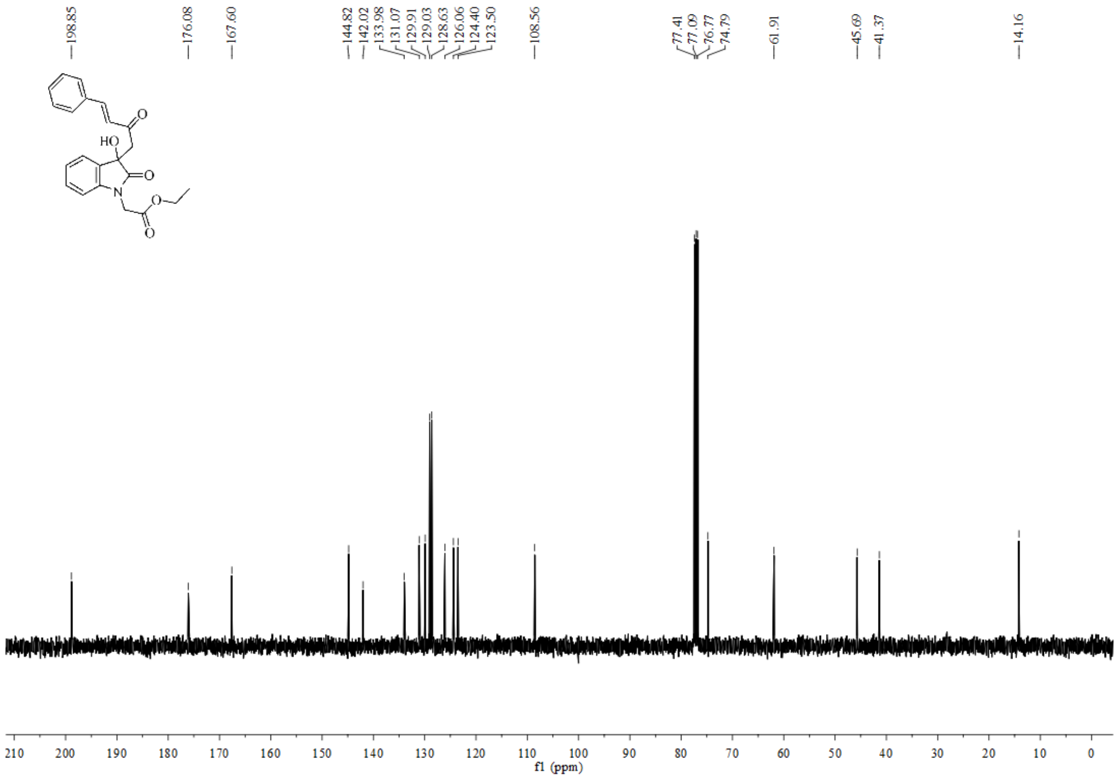

Figure S15. ^{13}C NMR Spectrum of compound **3b**

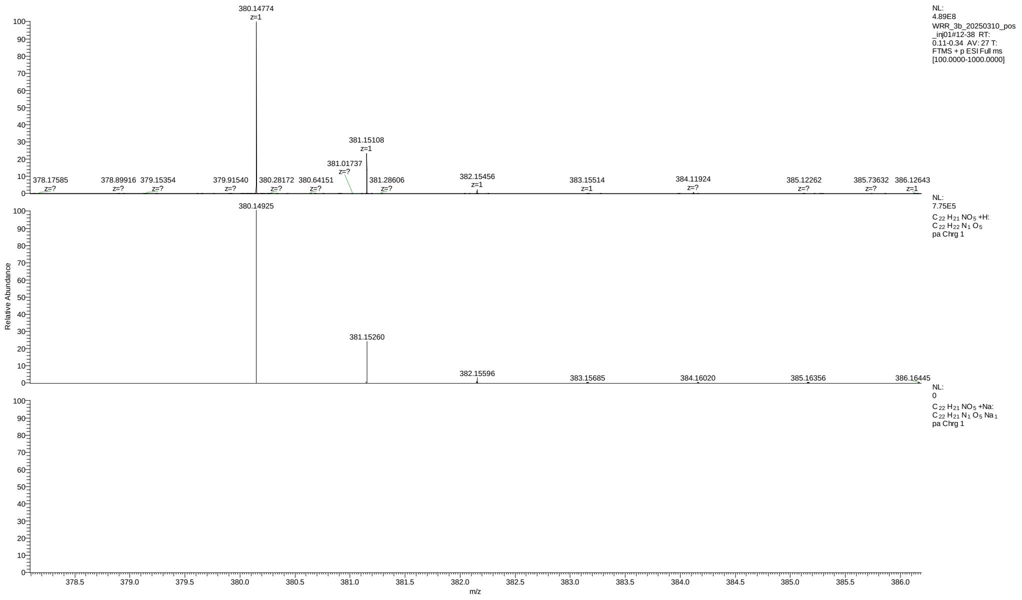

Figure S16. HRMS Spectrum of compound **3b**

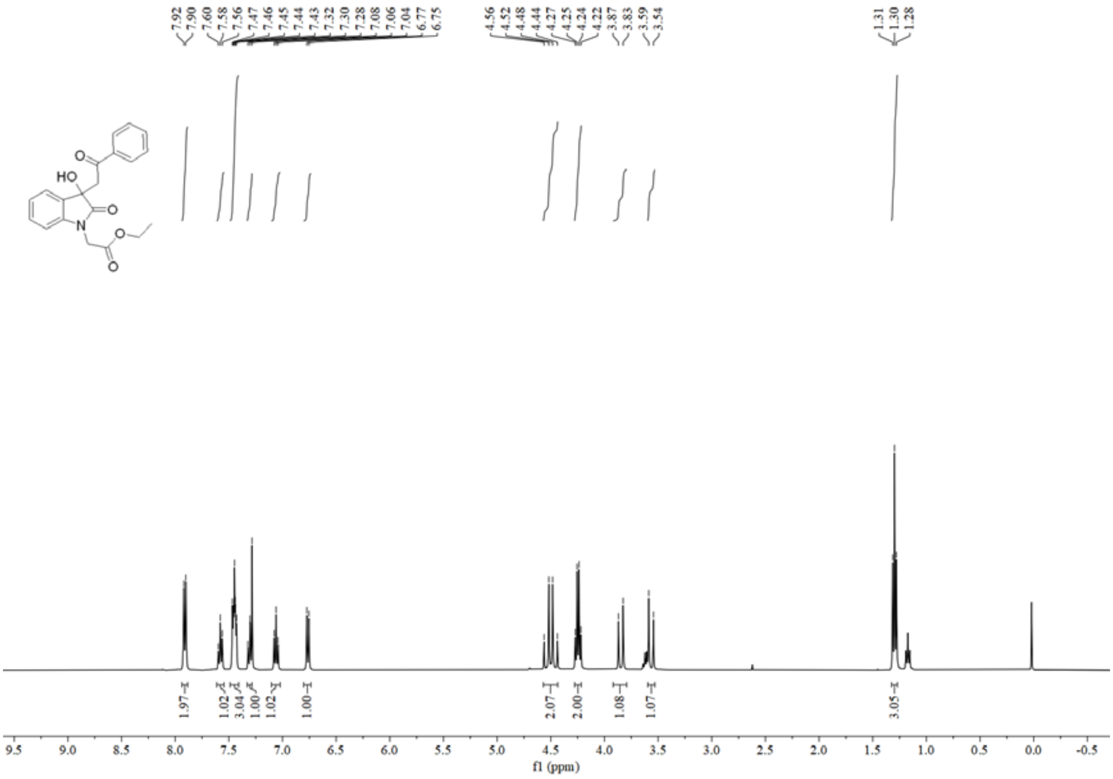

Figure S17. ^{1}H NMR Spectrum of compound **3c**

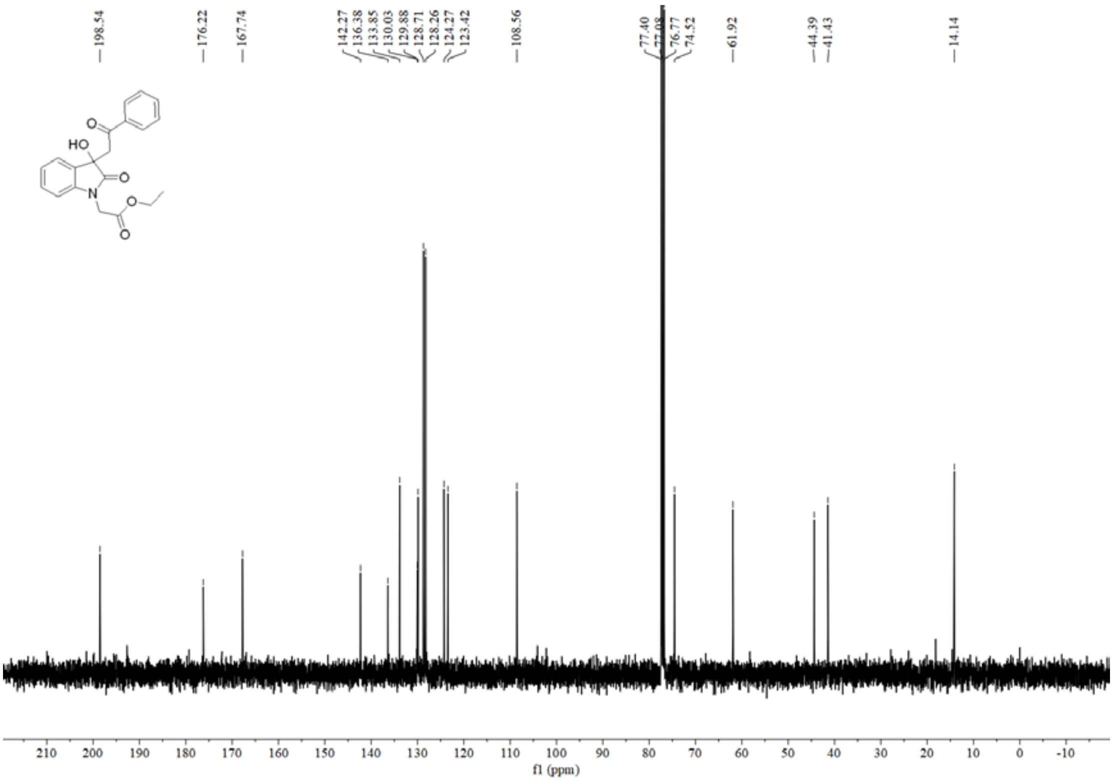

Figure S18. ^{13}C NMR Spectrum of compound **3c**

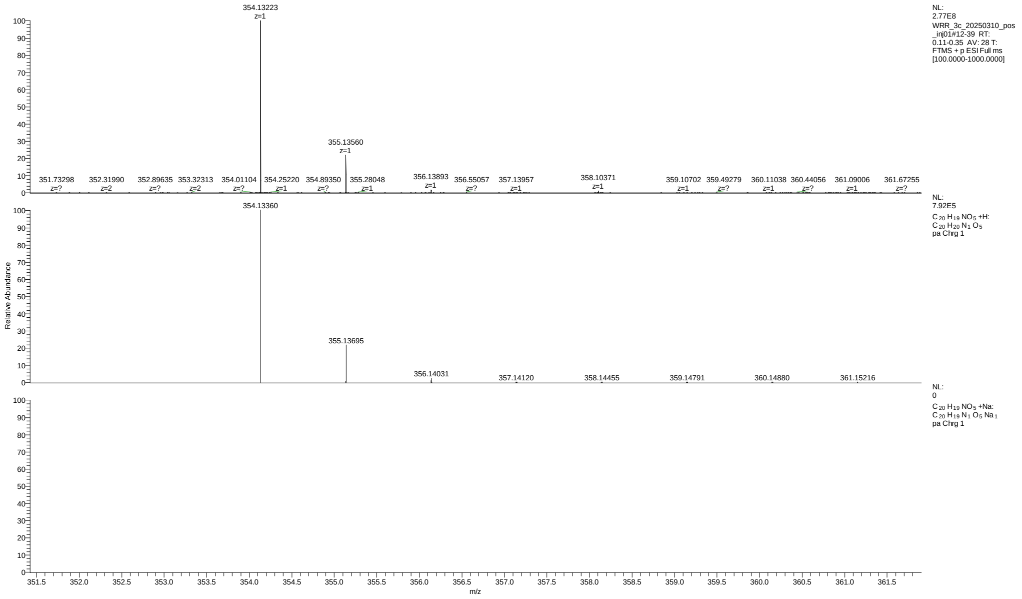

Figure S19. HRMS Spectrum of compound **3c**

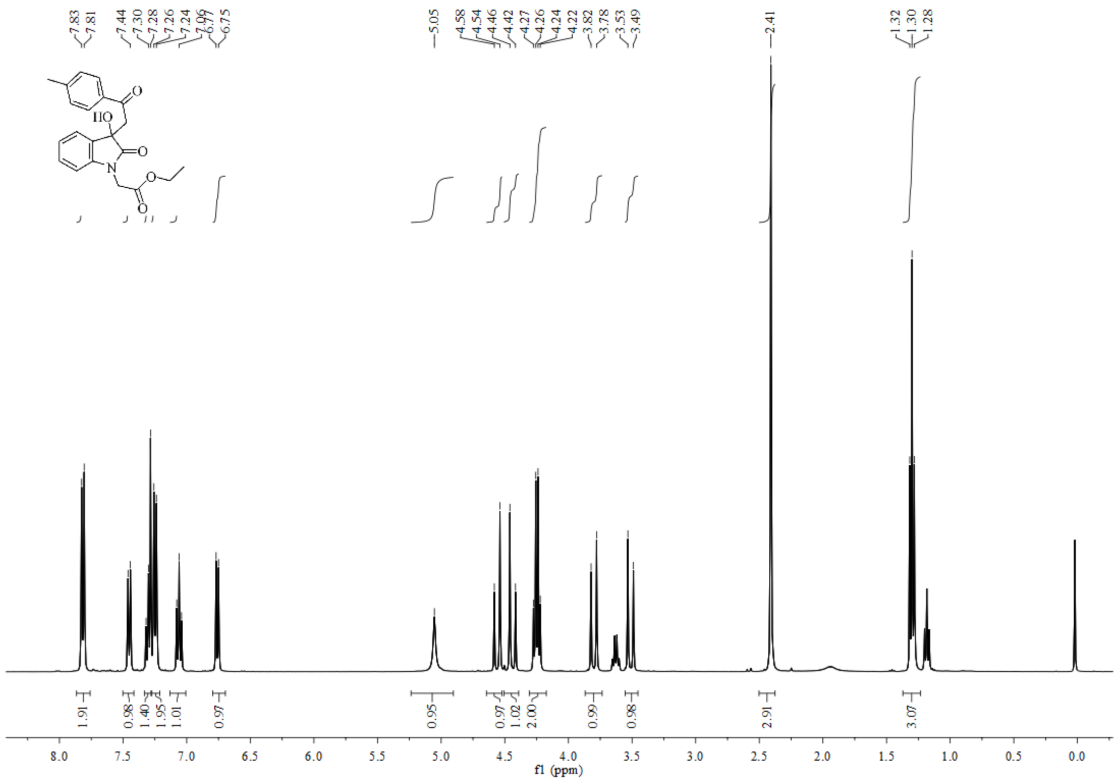

Figure S20. ^{1}H NMR Spectrum of compound **3d**

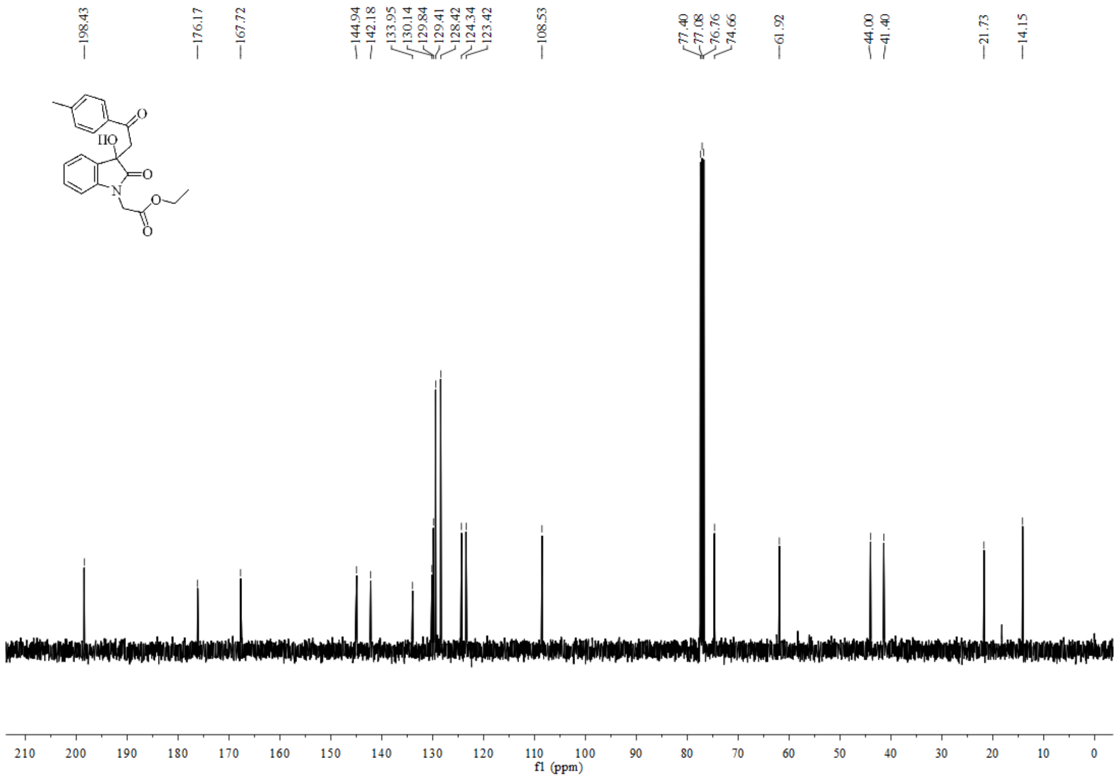

Figure S21. ^{13}C NMR Spectrum of compound **3d**

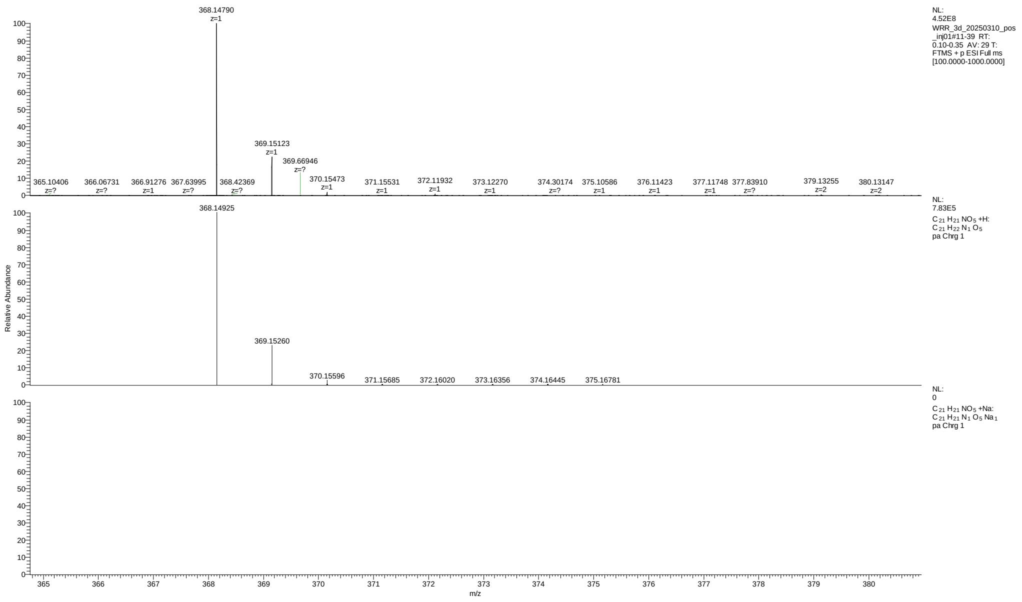

Figure S22. HRMS Spectrum of compound **3d**

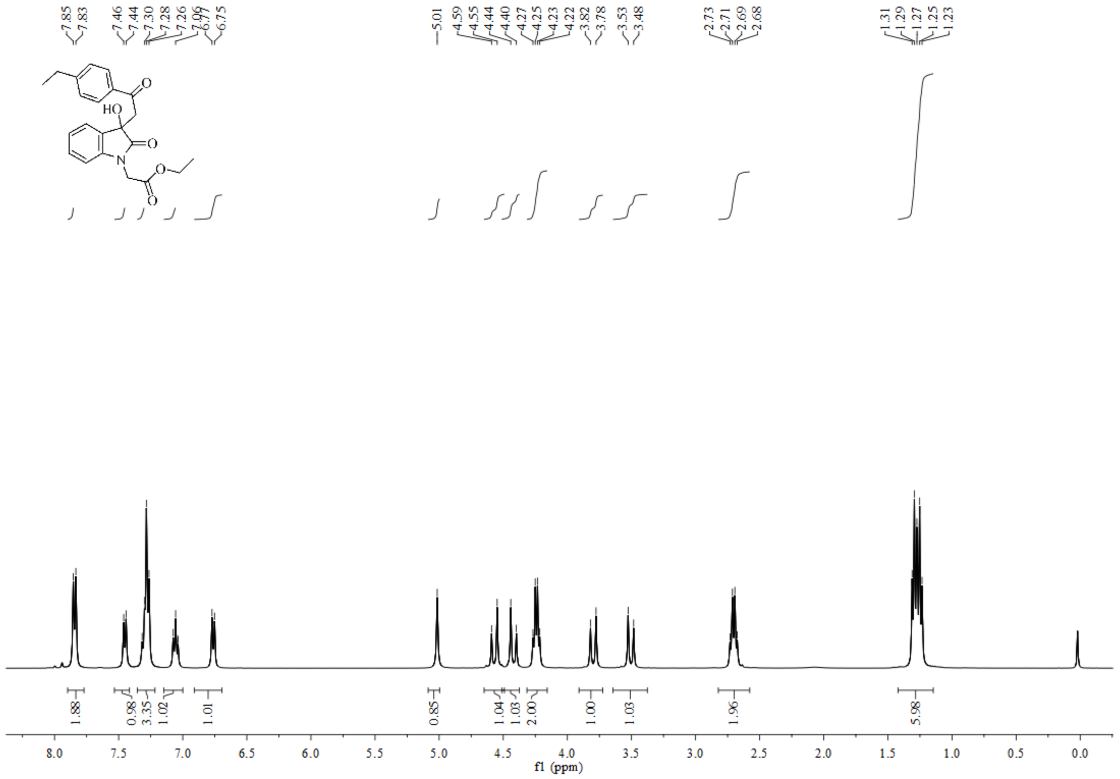

Figure S23. ^{1}H NMR Spectrum of compound **3e**

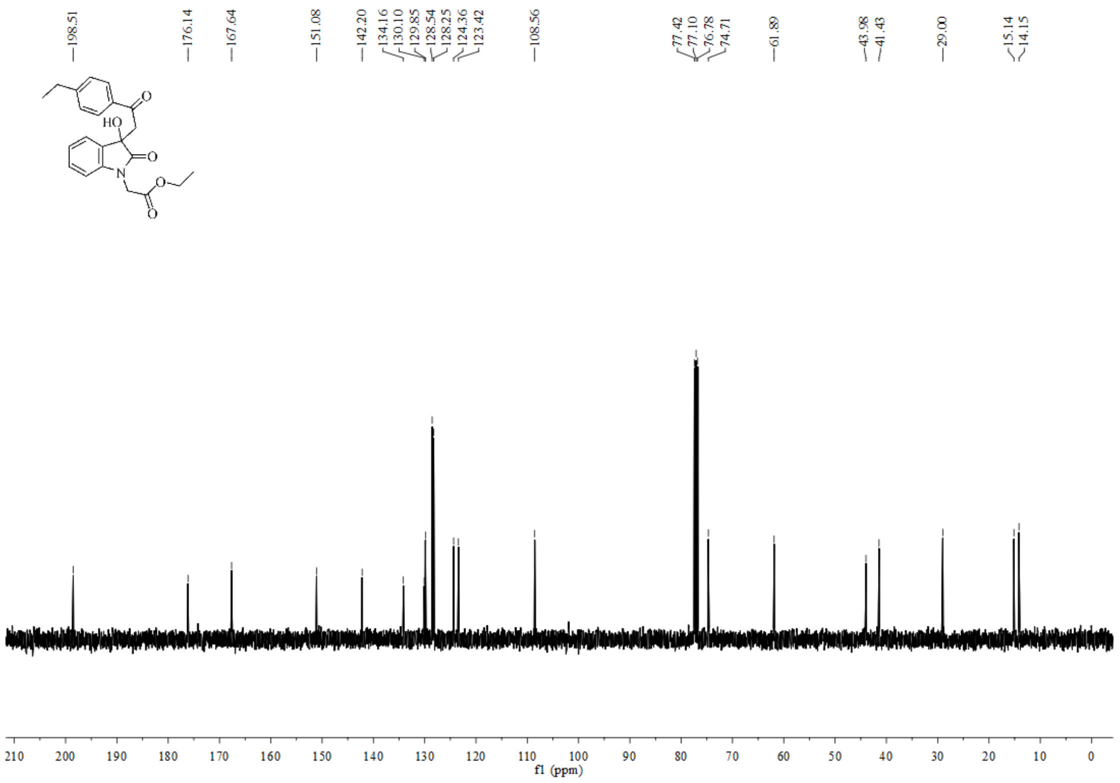

Figure S24. ^{13}C NMR Spectrum of compound **3e**

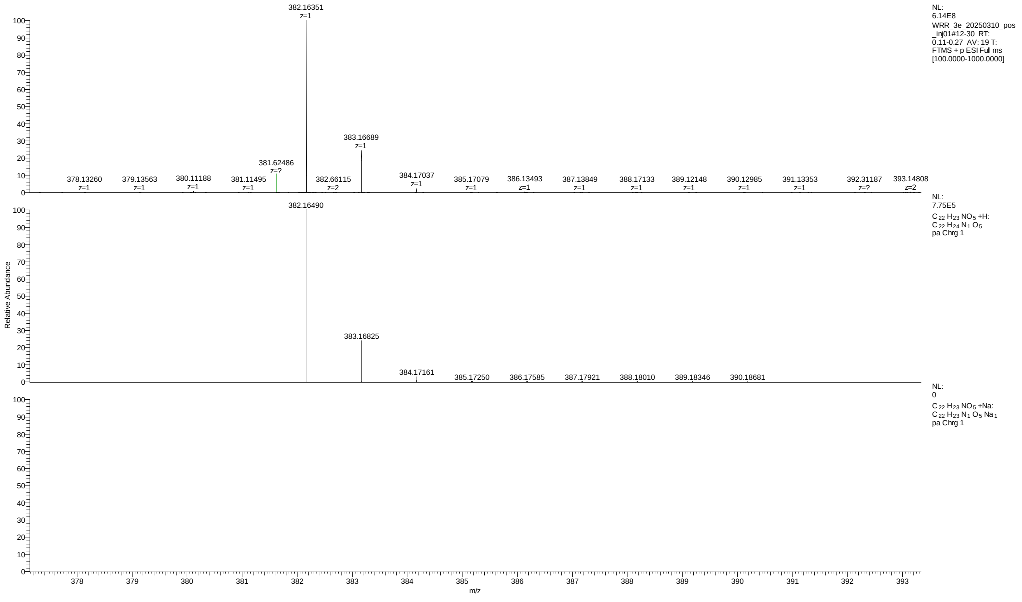

Figure S25. HRMS Spectrum of compound **3e**

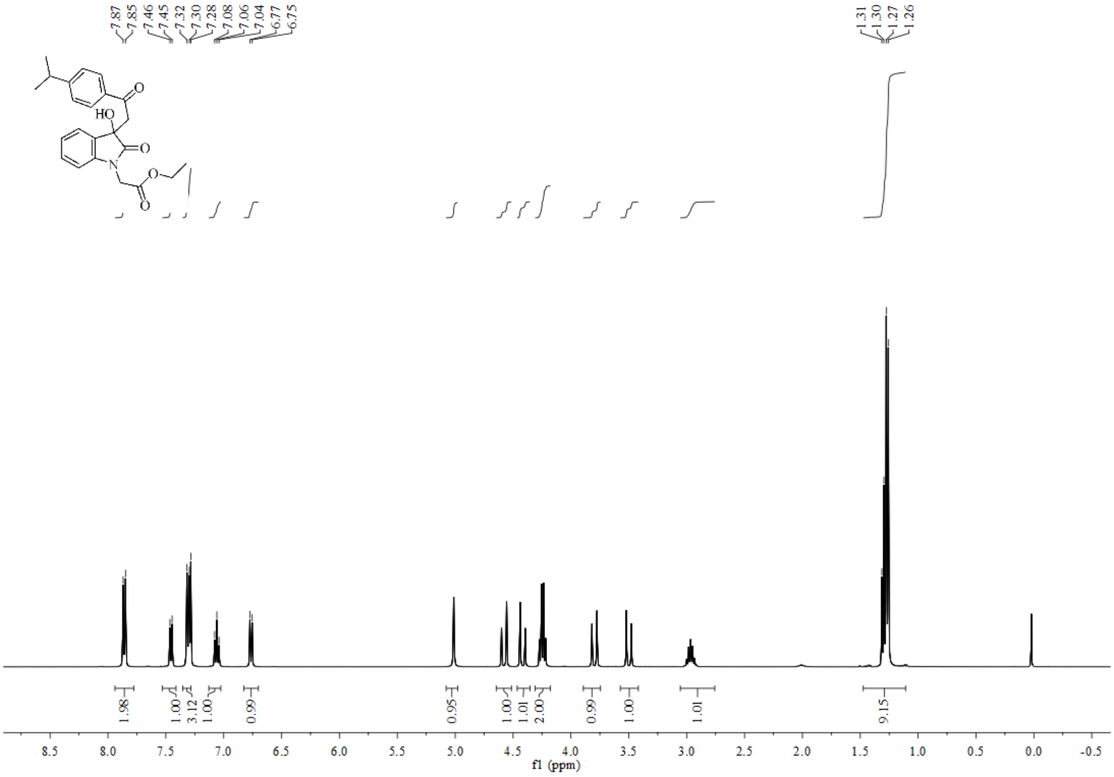

Figure S26. ^{1}H NMR Spectrum of compound **3f**

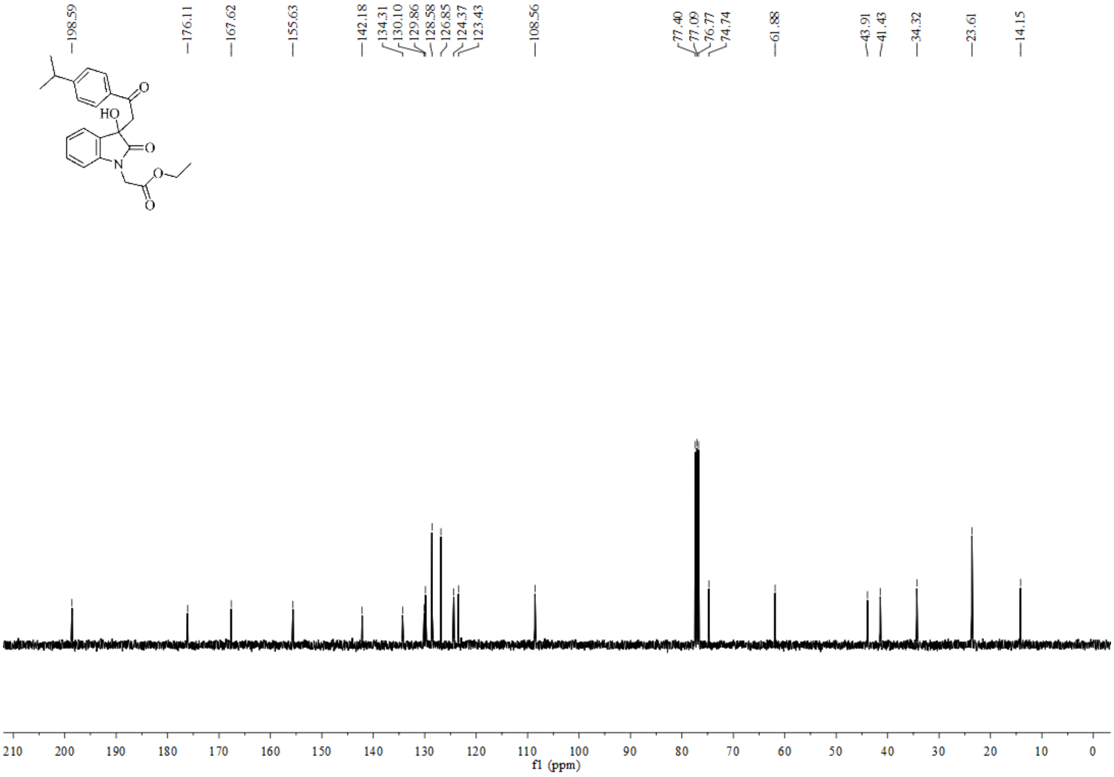

Figure S27. ^{13}C NMR Spectrum of compound **3f**

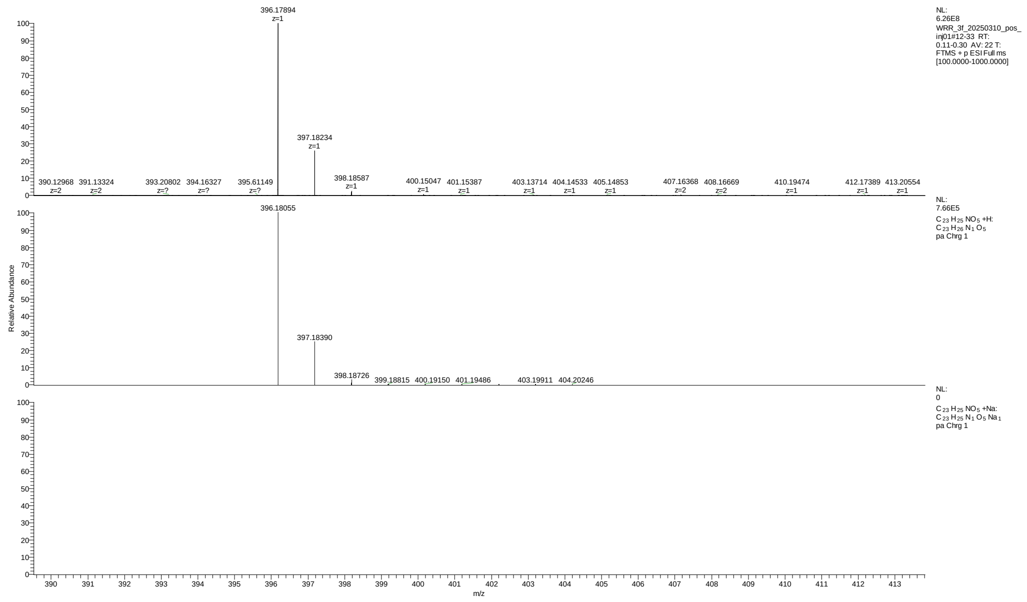

Figure S28. HRMS Spectrum of compound **3f**

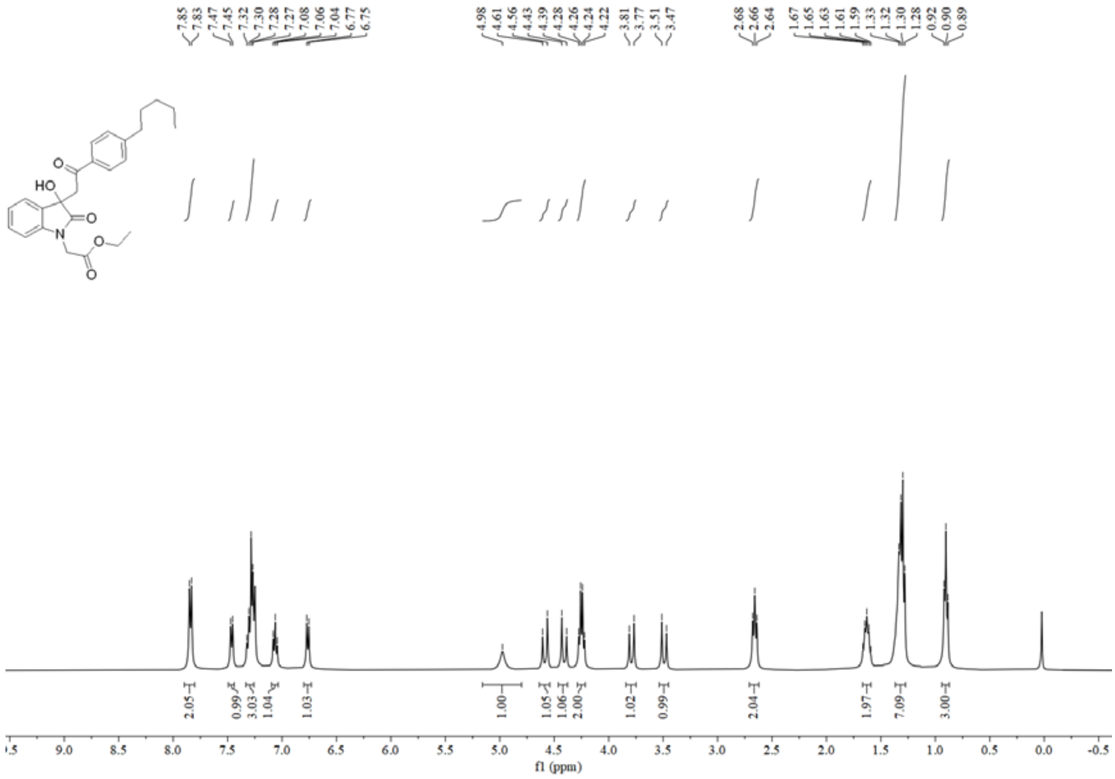

Figure S29. ^{1}H NMR Spectrum of compound **3g**

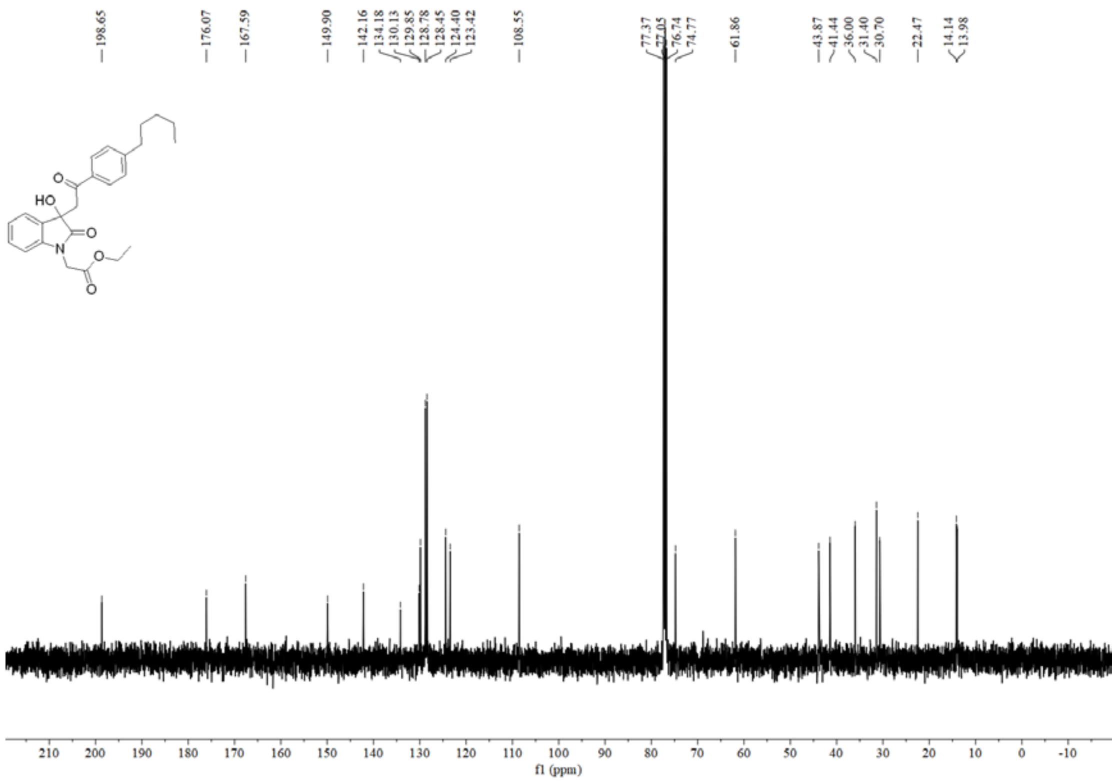

Figure S30. ^{13}C NMR Spectrum of compound **3g**

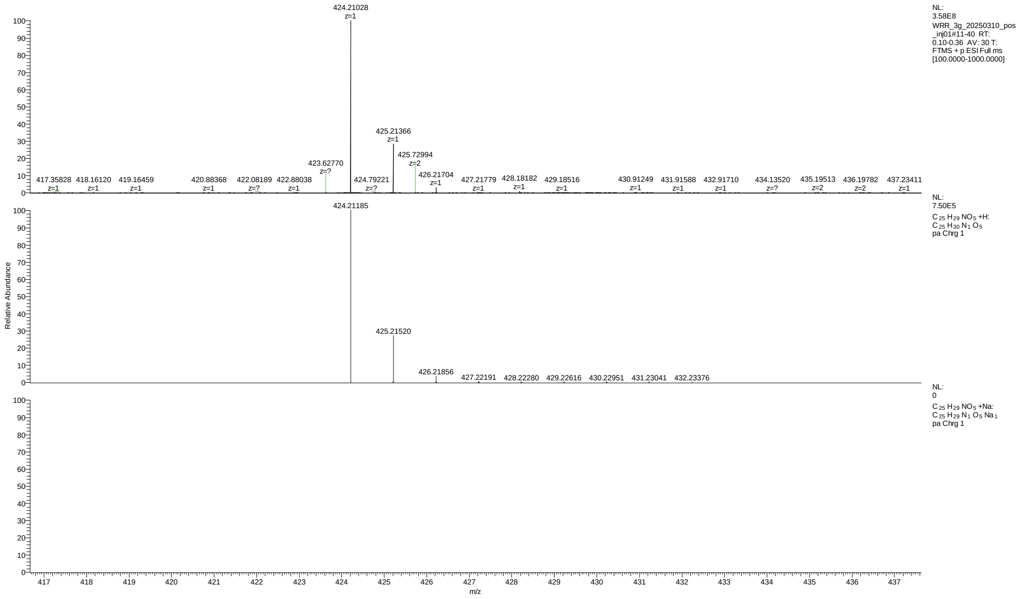

Figure S31. HRMS Spectrum of compound **3g**

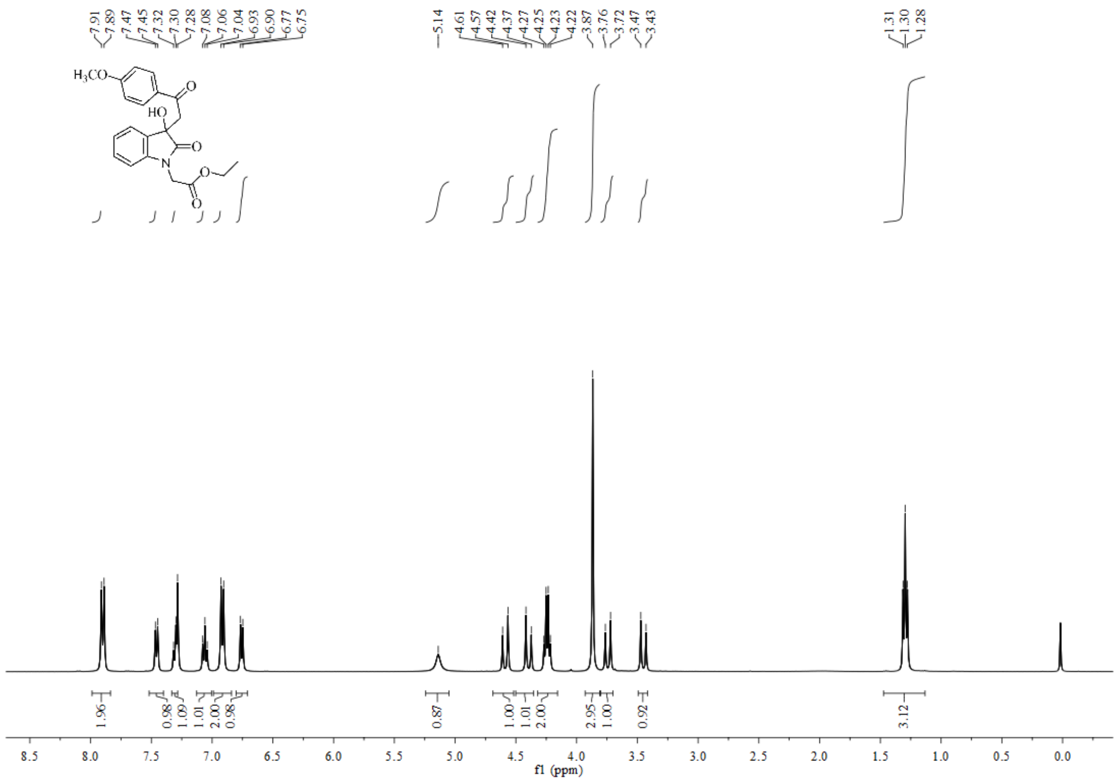

Figure S32. ^{1}H NMR Spectrum of compound **3h**

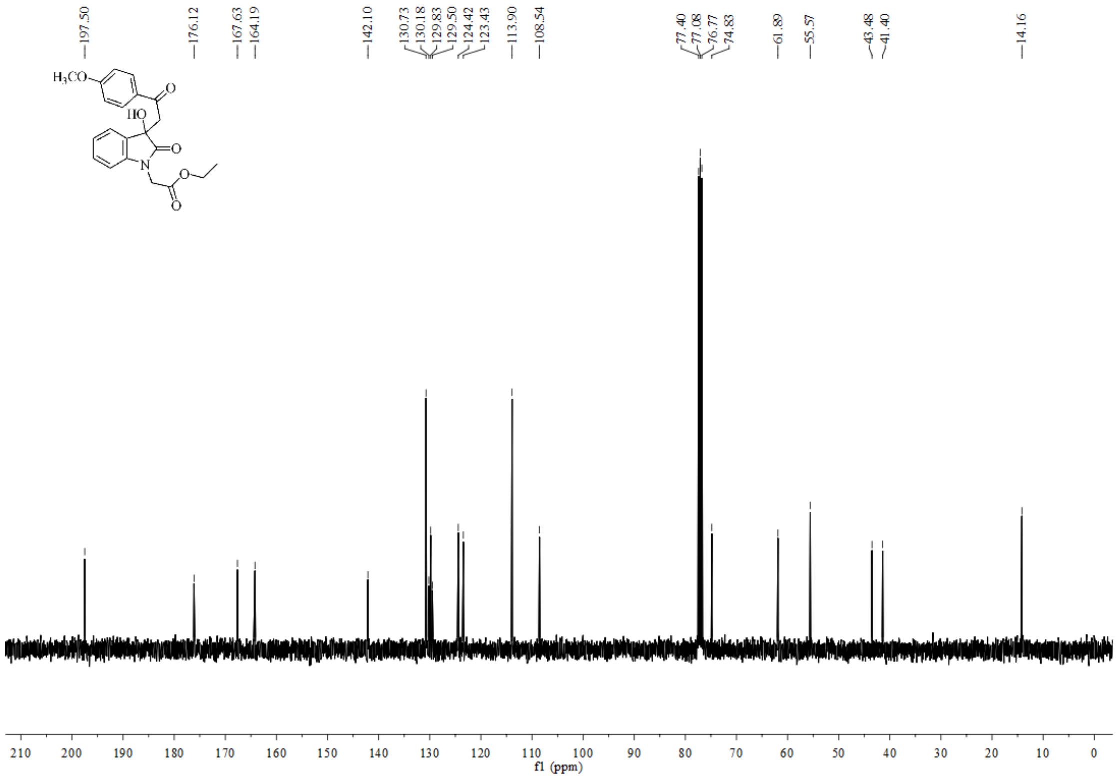

Figure S33. ^{13}C NMR Spectrum of compound **3h**

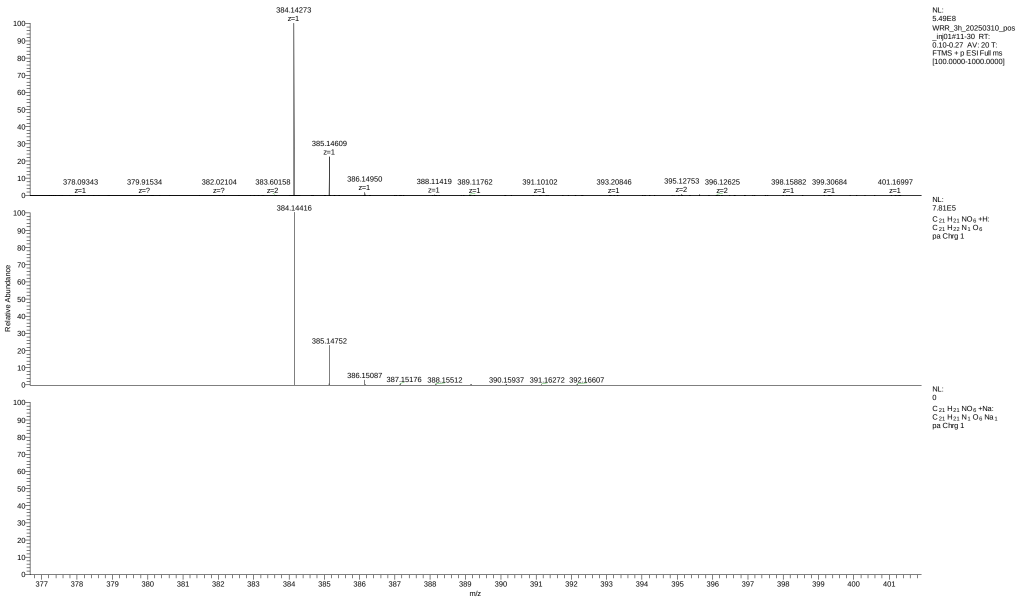

Figure S34. HRMS Spectrum of compound **3h**

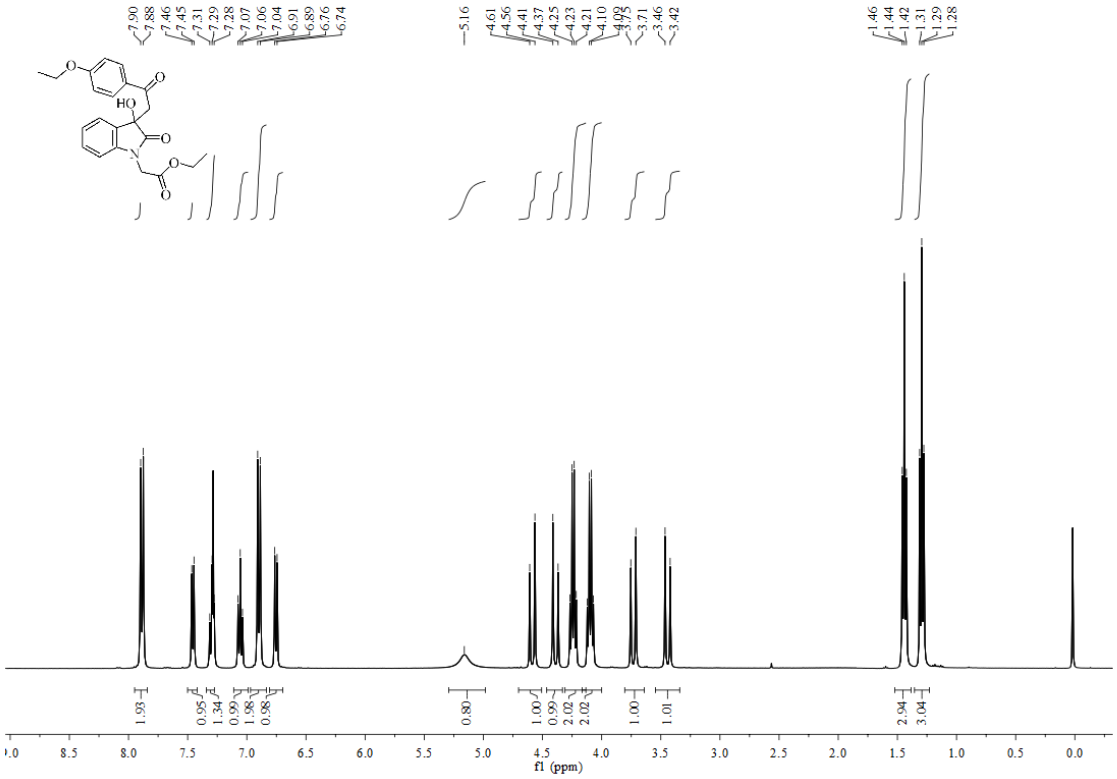

Figure S35. ^{1}H NMR Spectrum of compound **3i**

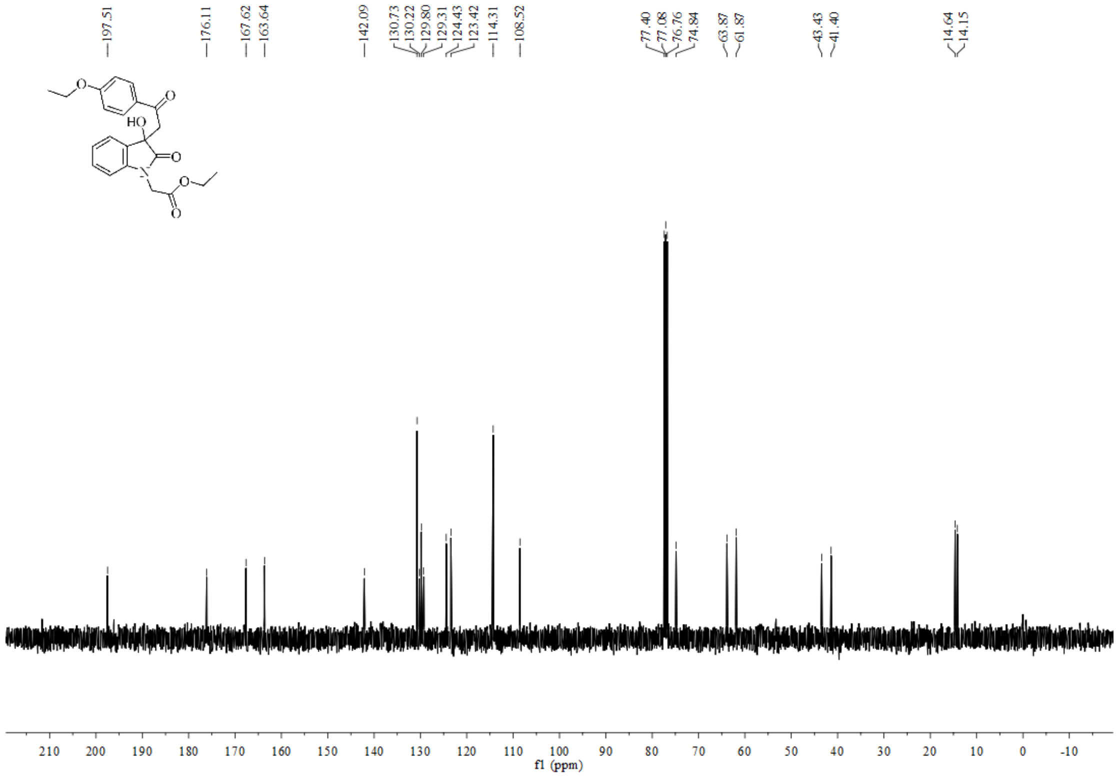

Figure S36. ^{13}C NMR Spectrum of compound **3i**

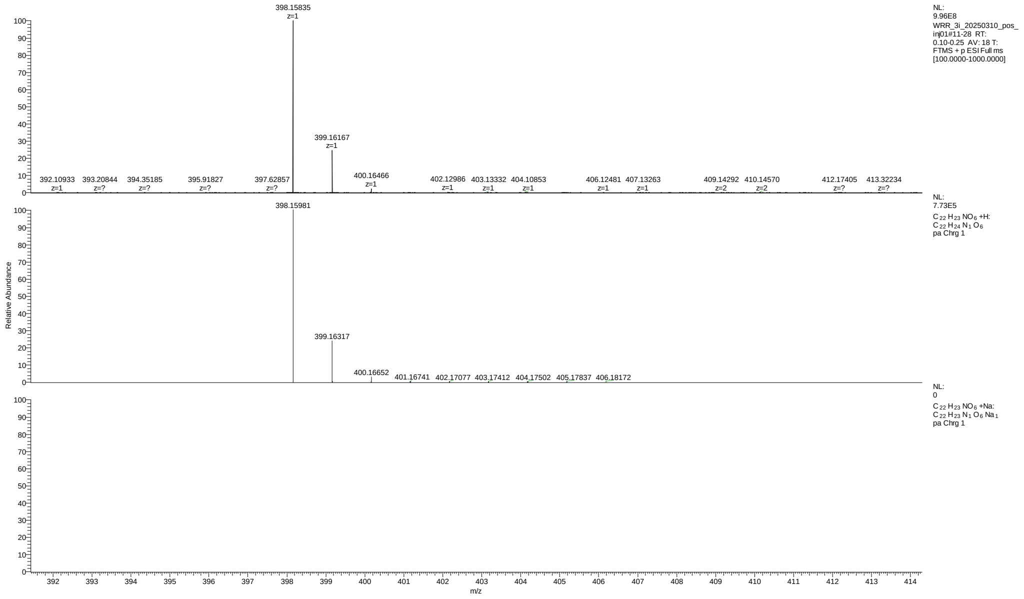

Figure S37. HRMS Spectrum of compound **3i**

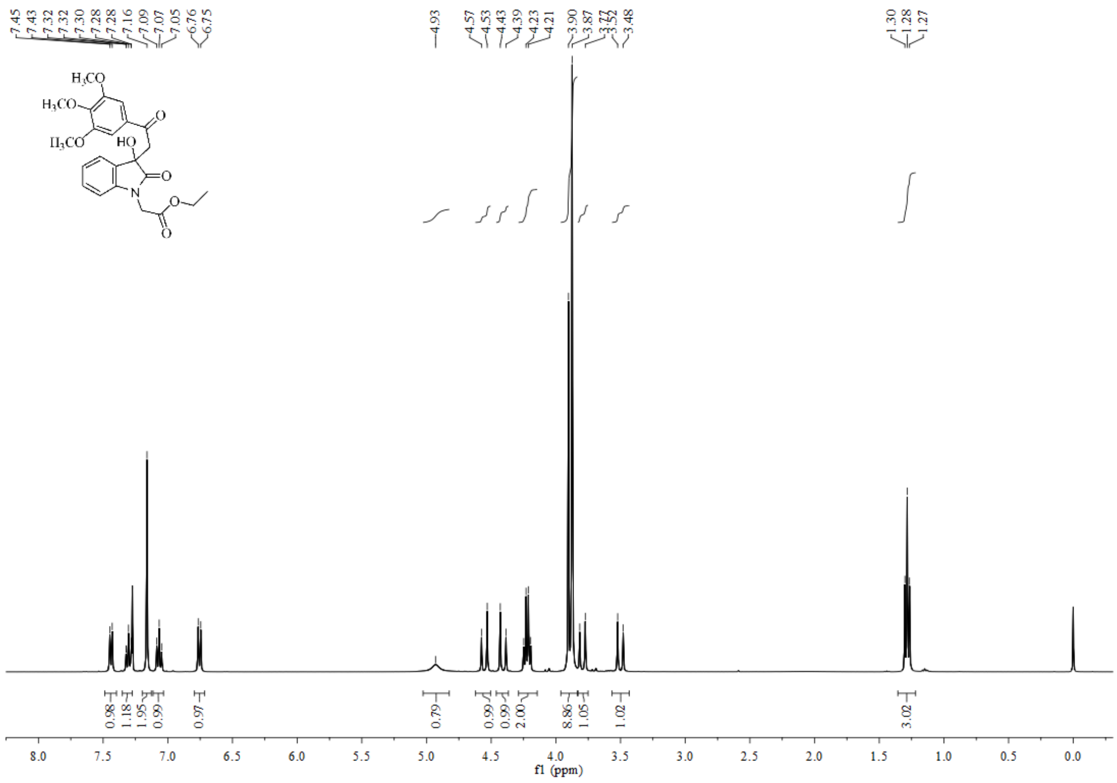

Figure S38. ^{1}H NMR Spectrum of compound **3j**

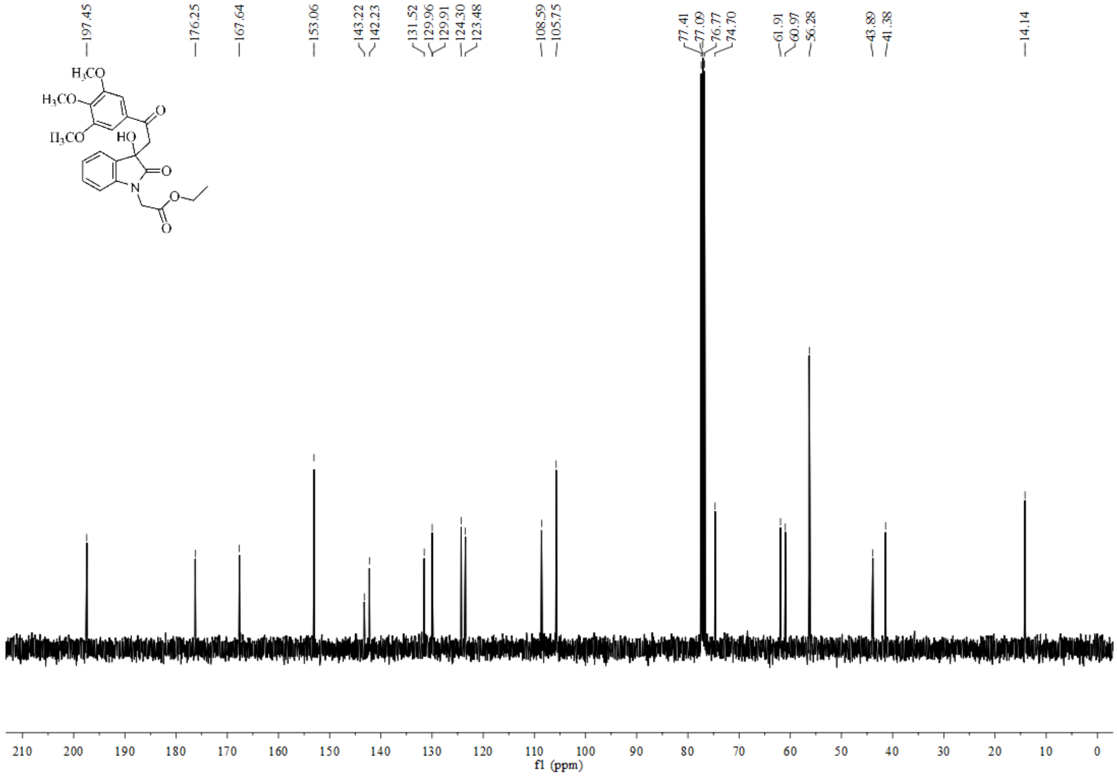

Figure S39. ^{13}C NMR Spectrum of compound **3j**

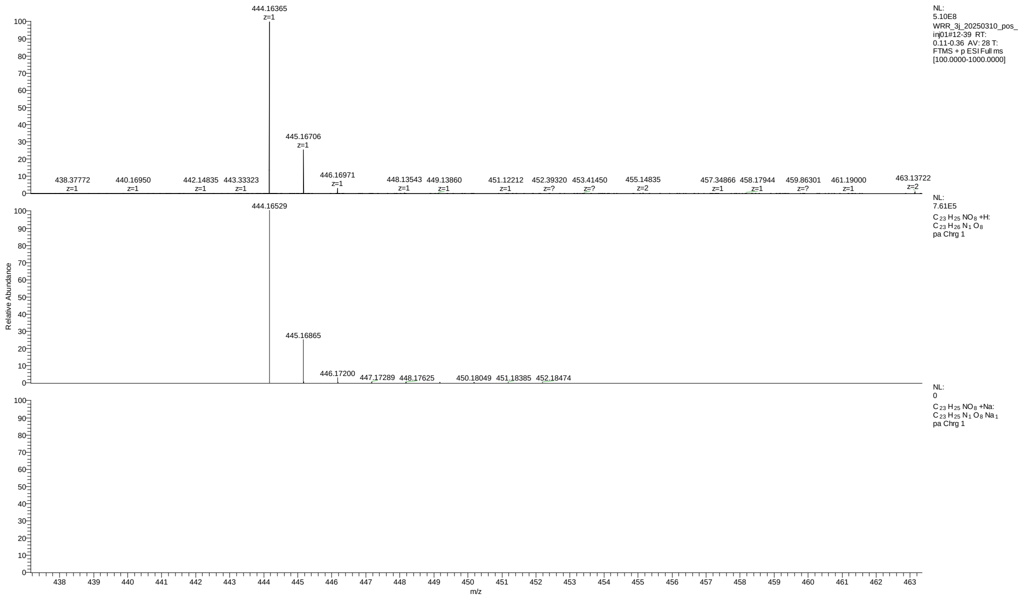

Figure S40. HRMS Spectrum of compound **3j**

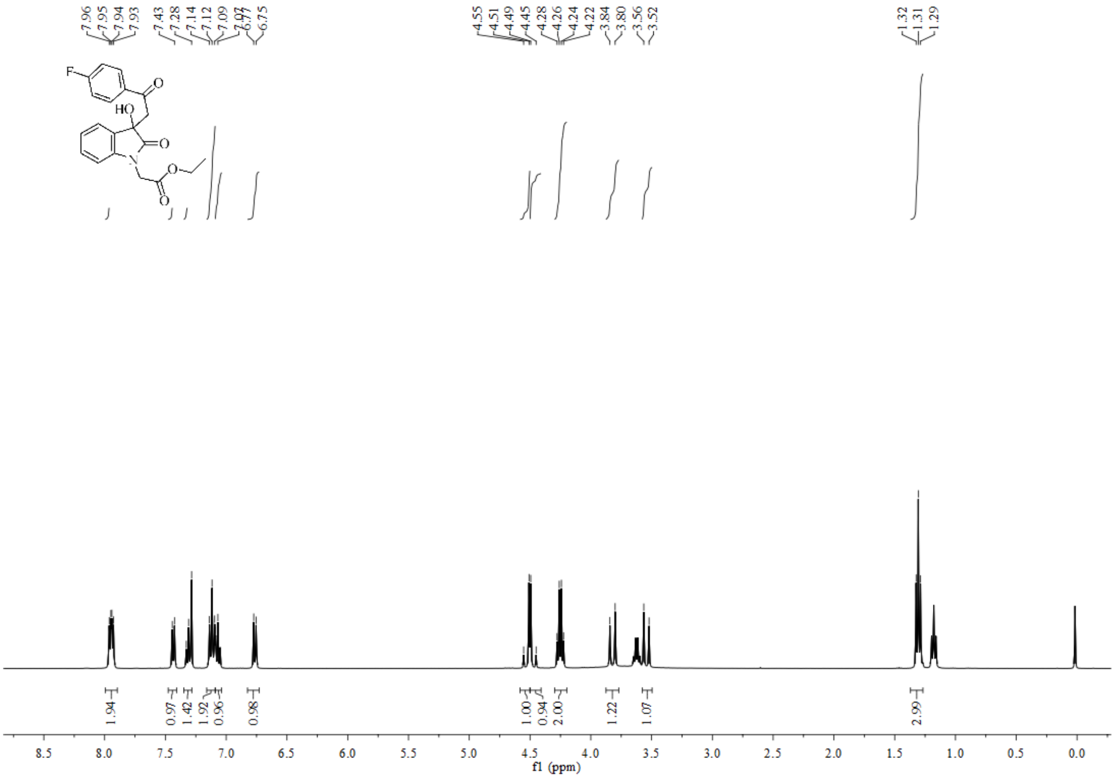

Figure S41. ^{1}H NMR Spectrum of compound **3k**

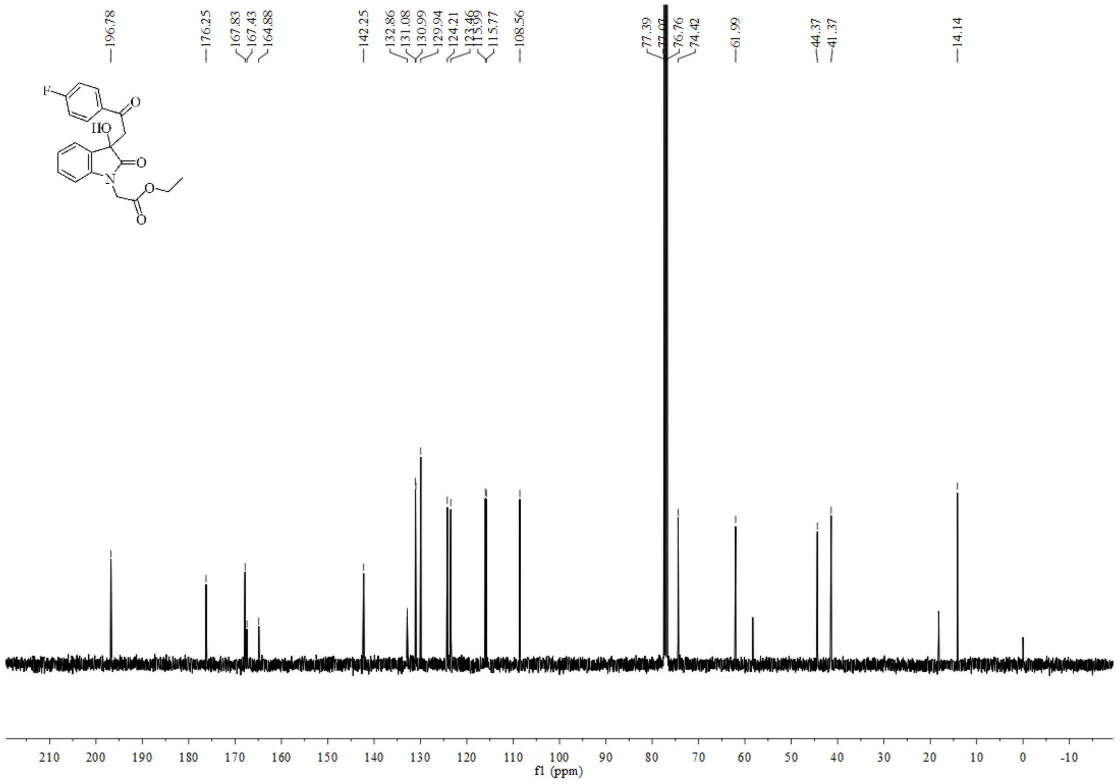

Figure S42. ^{13}C NMR Spectrum of compound **3k**

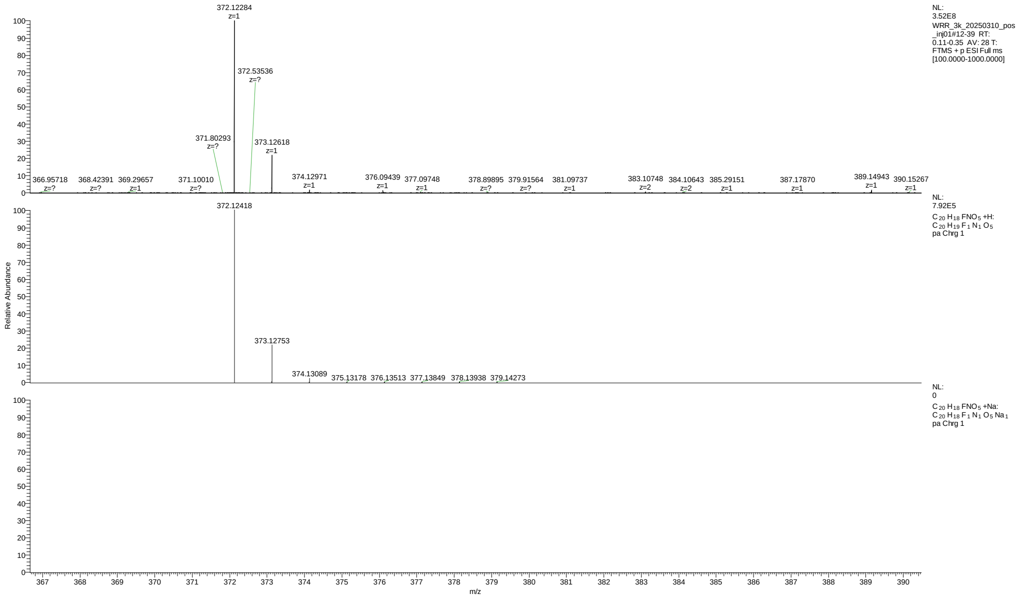

Figure S43. HRMS Spectrum of compound **3k**

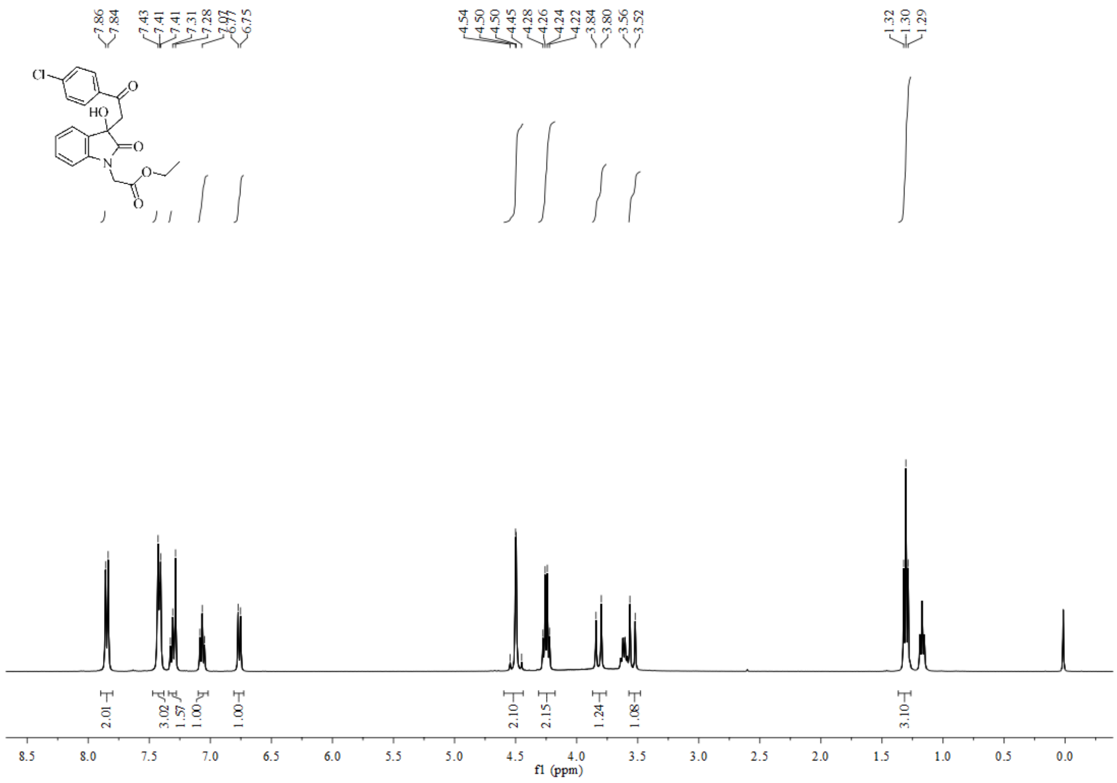

Figure S44. ^{1}H NMR Spectrum of compound **3l**

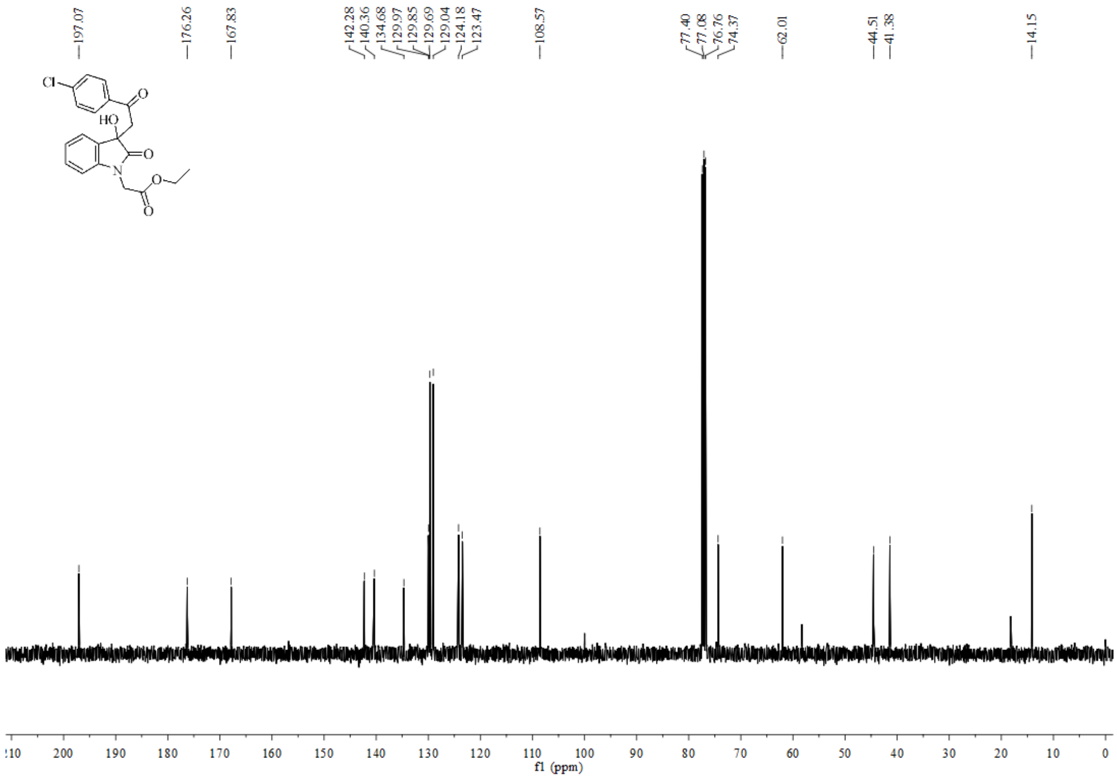

Figure S45. ^{13}C NMR Spectrum of compound **3l**

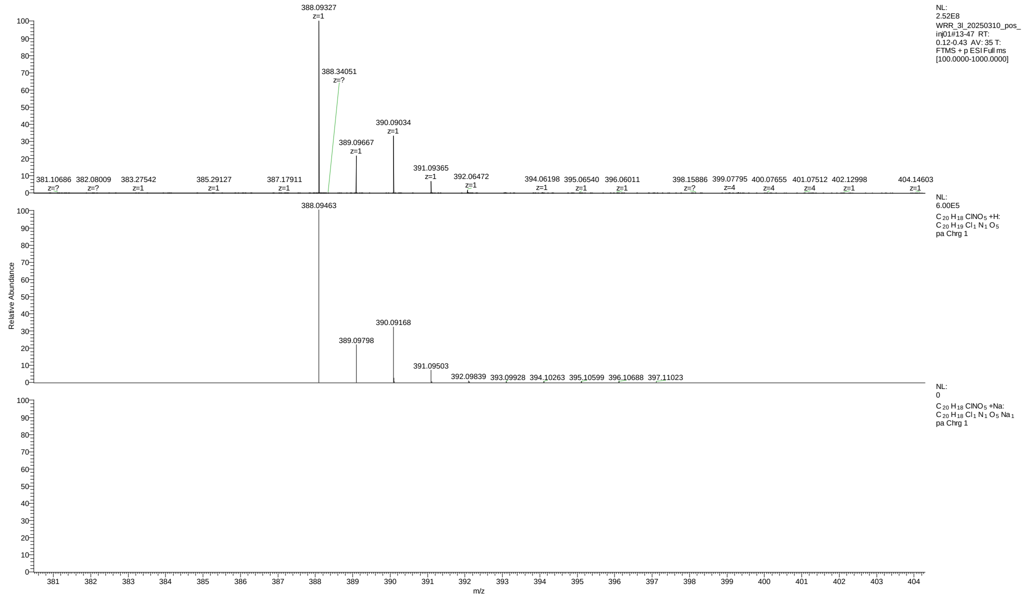

Figure S46. HRMS Spectrum of compound **3l**

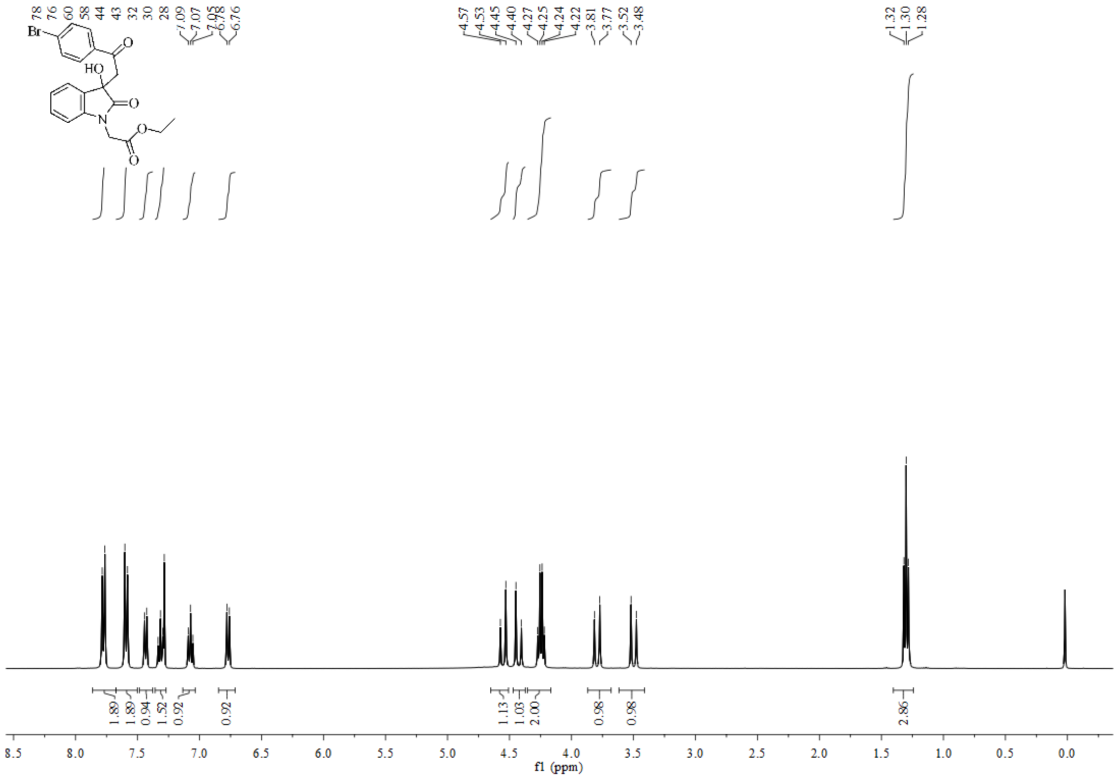

Figure S47. ^{1}H NMR Spectrum of compound **3m**

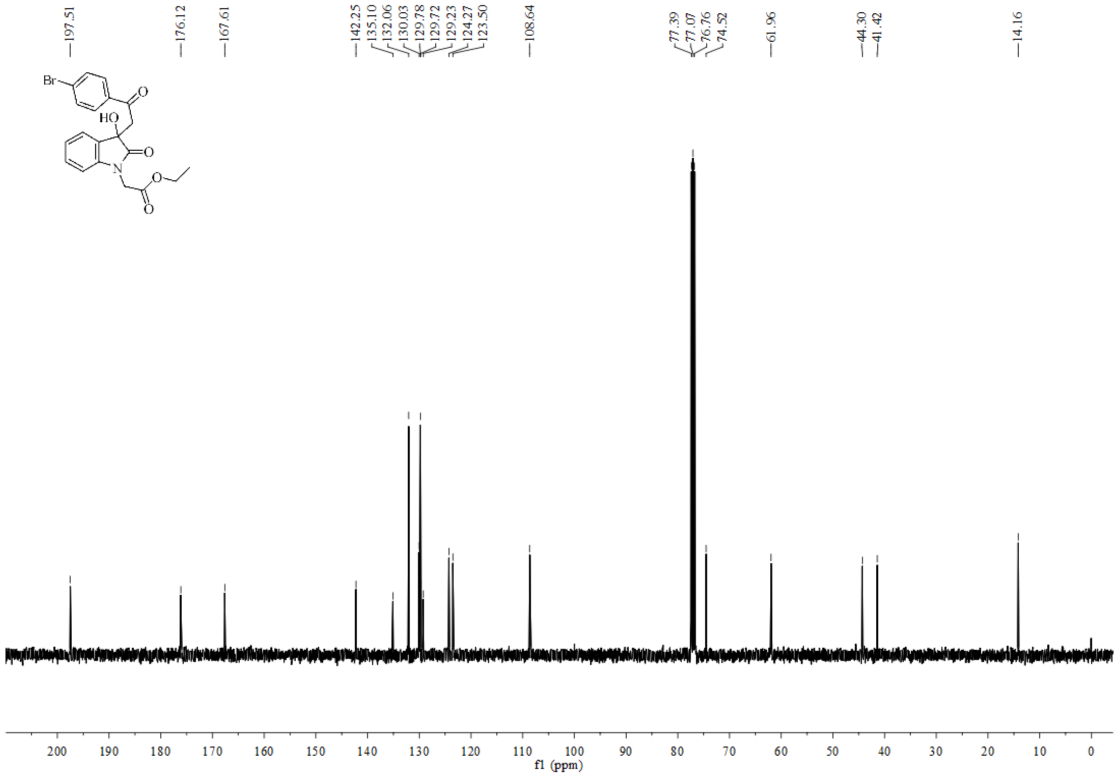

Figure S48. ^{13}C NMR Spectrum of compound **3m**

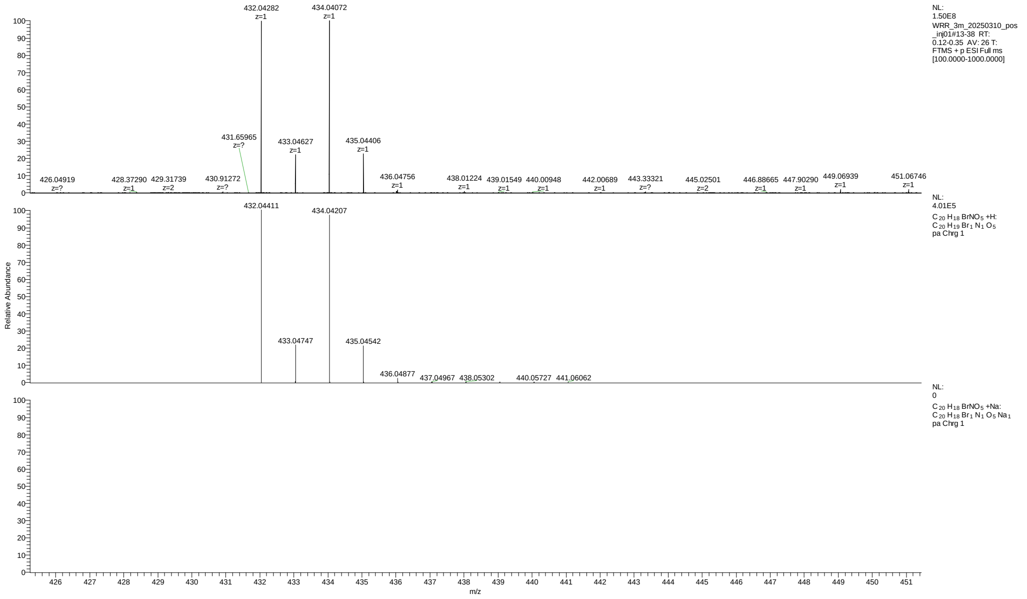

Figure S49. HRMS Spectrum of compound **3m**

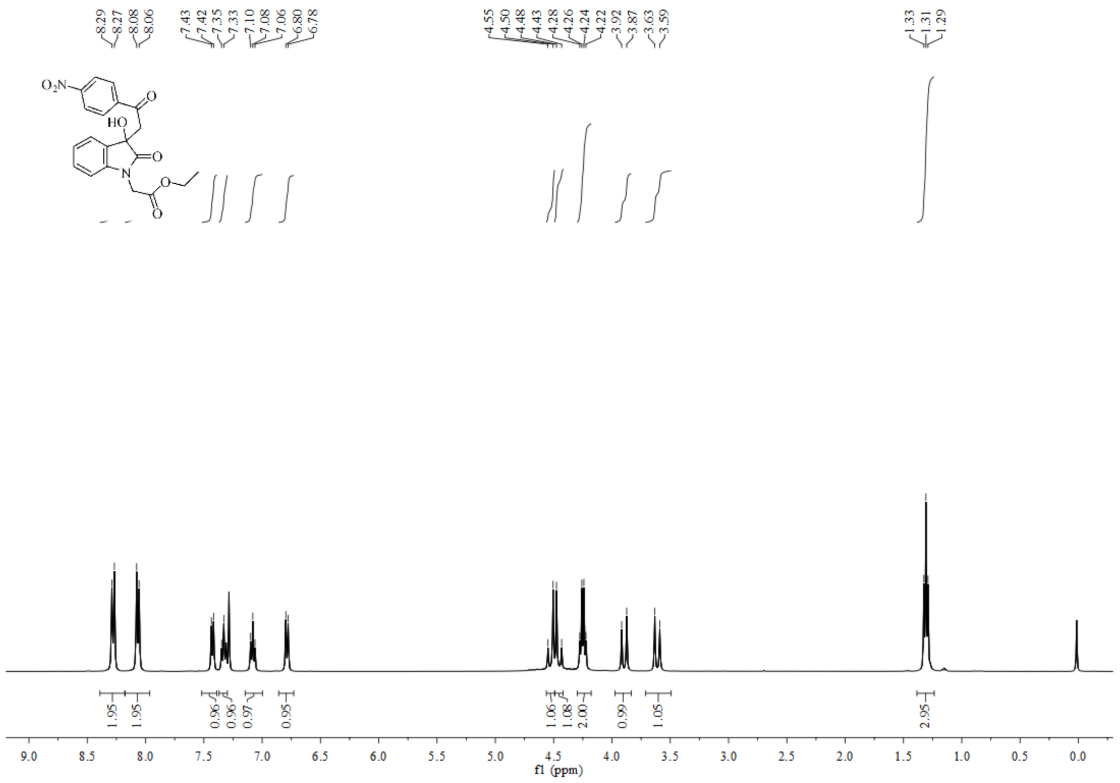

Figure S50. ^{1}H NMR Spectrum of compound **3n**

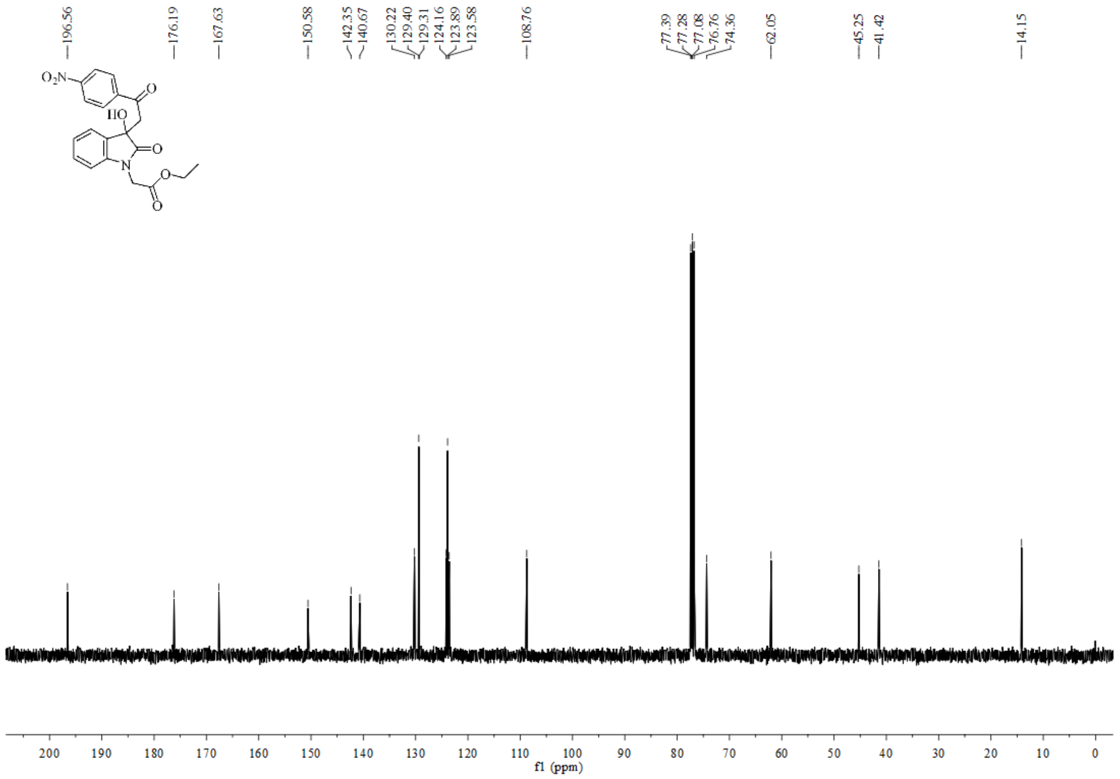

Figure S51. ^{13}C NMR Spectrum of compound **3n**

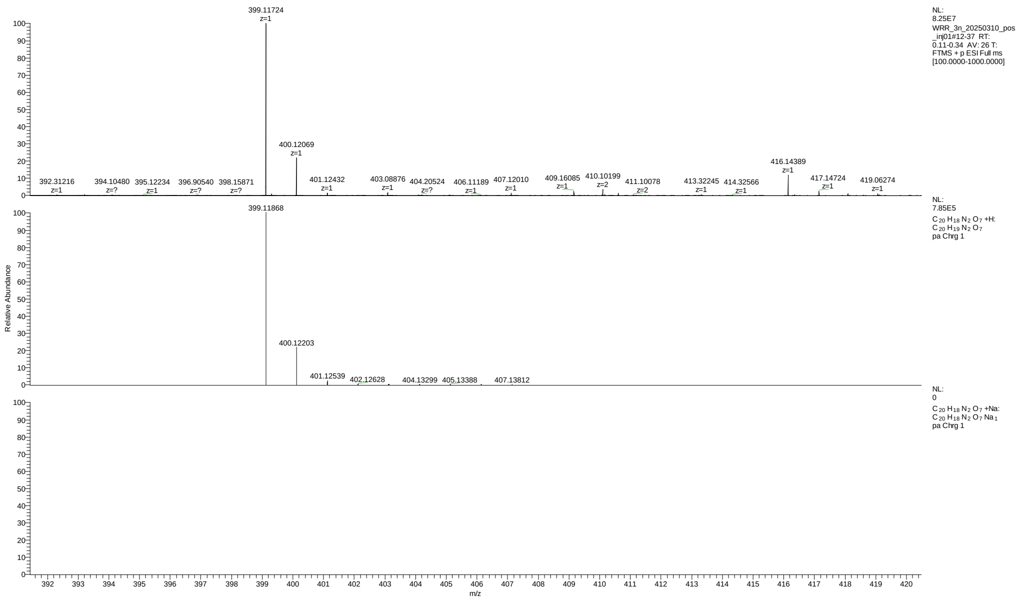

Figure S52. HRMS Spectrum of compound **3n**

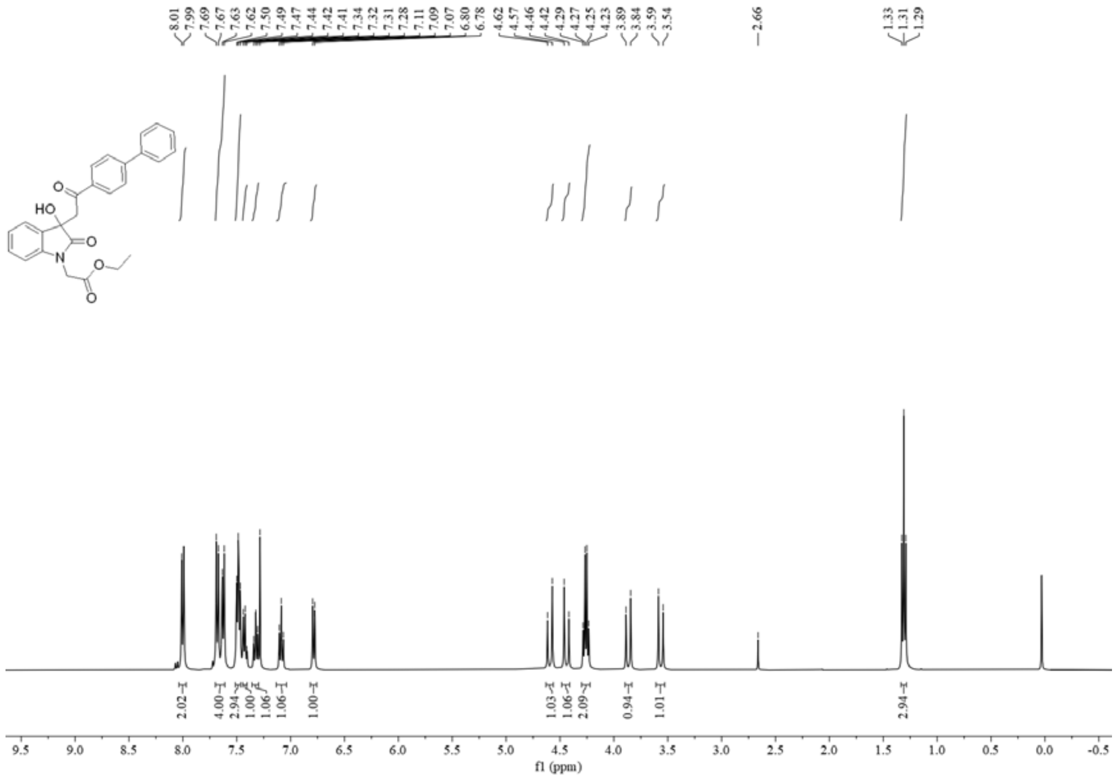

Figure S53. ^{1}H NMR Spectrum of compound **3o**

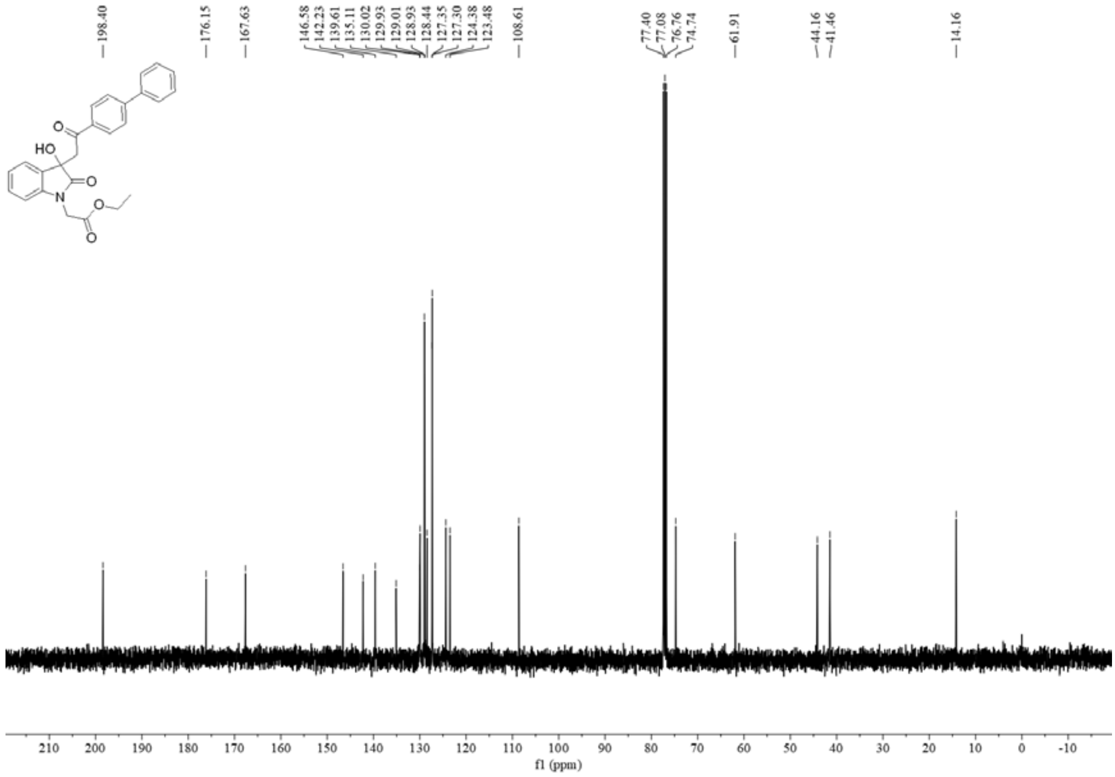

Figure S54. ^{13}C NMR Spectrum of compound **3o**

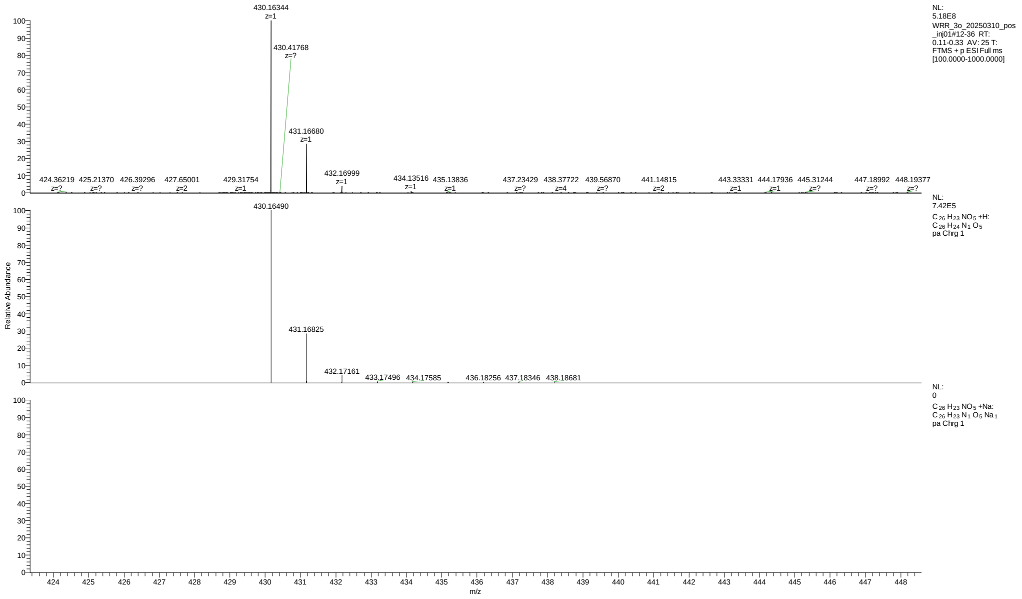

Figure S55. HRMS Spectrum of compound **3o**

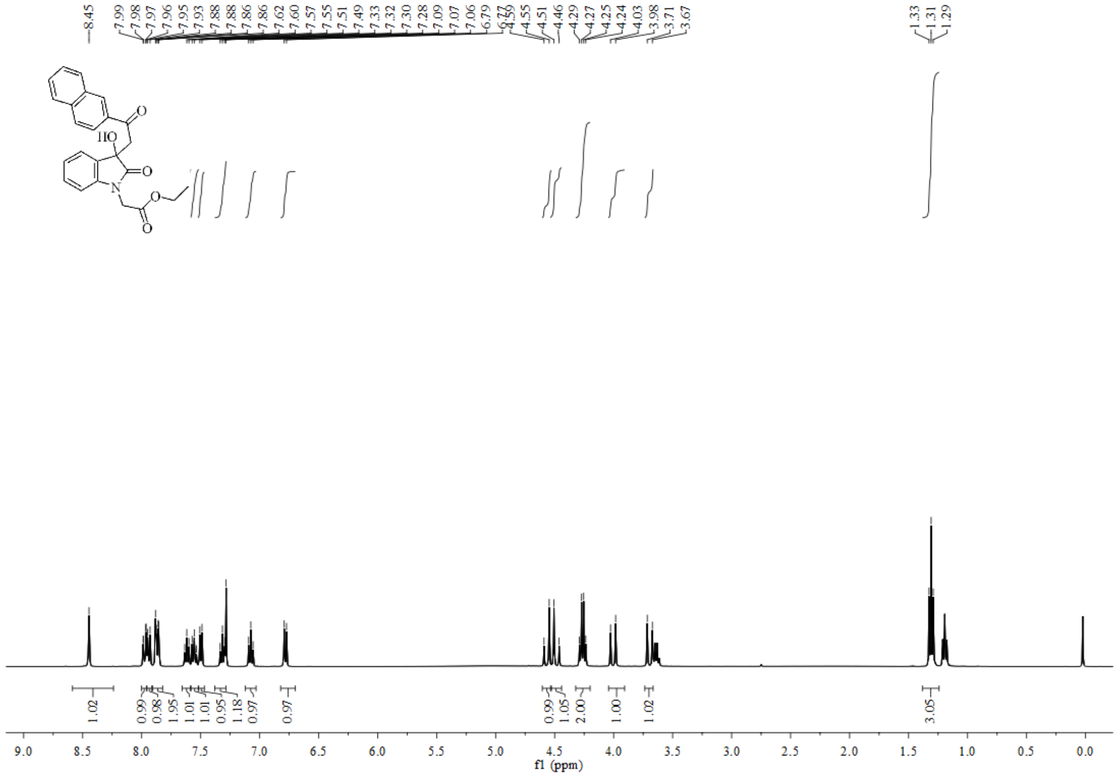

Figure S56. ^{1}H NMR Spectrum of compound **3p**

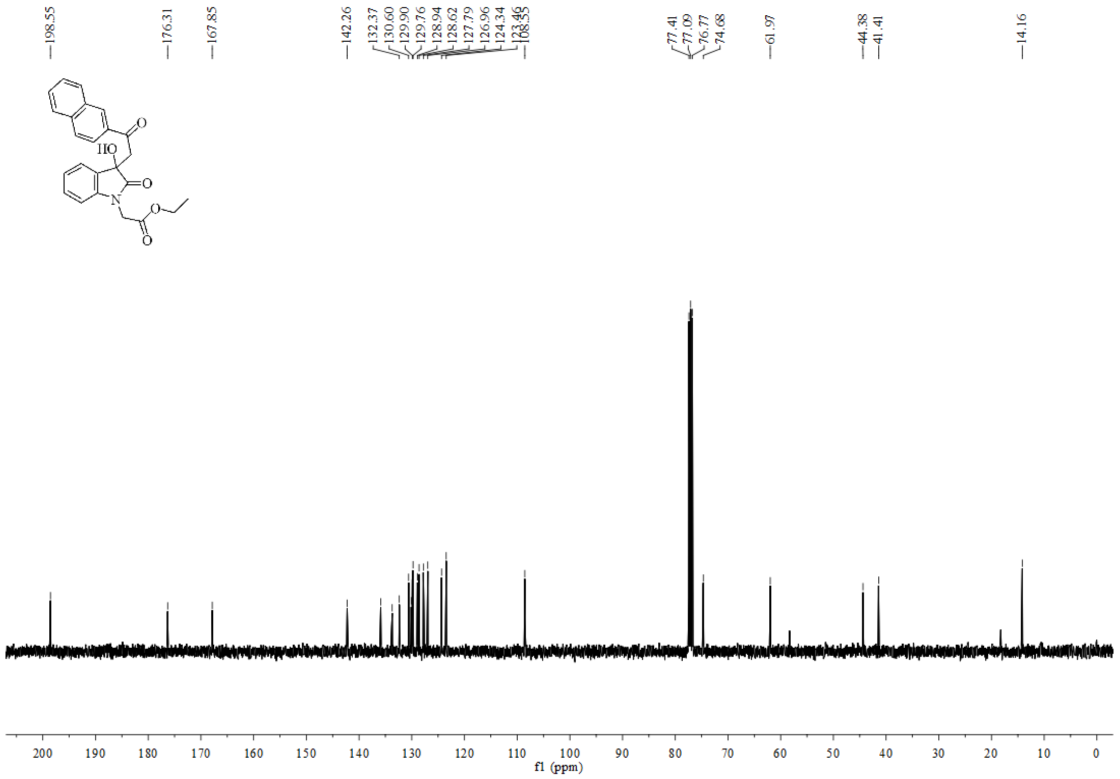

Figure S57. ^{13}C NMR Spectrum of compound **3p**

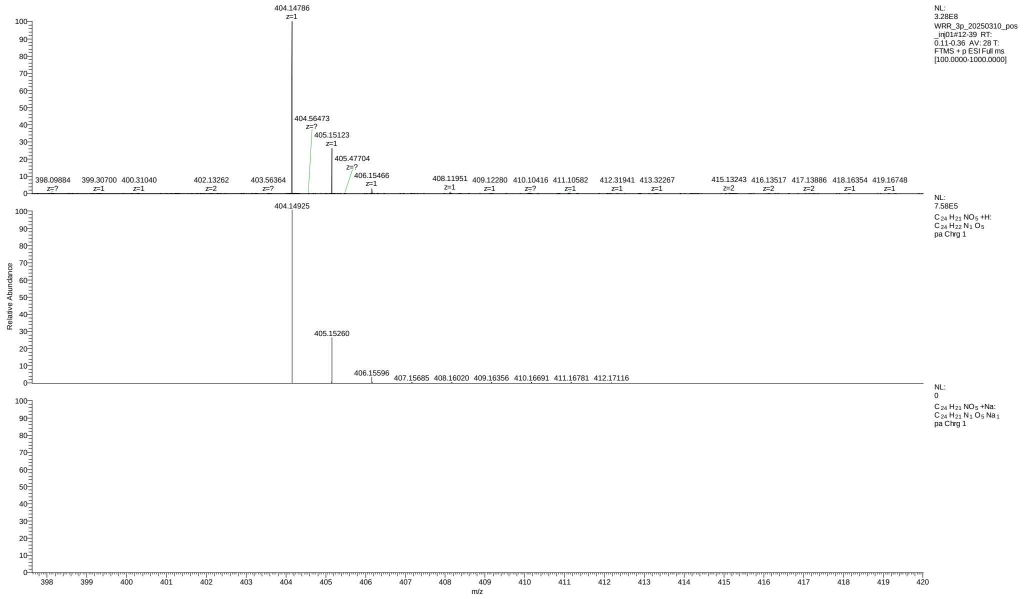

Figure S58. HRMS Spectrum of compound **3p**

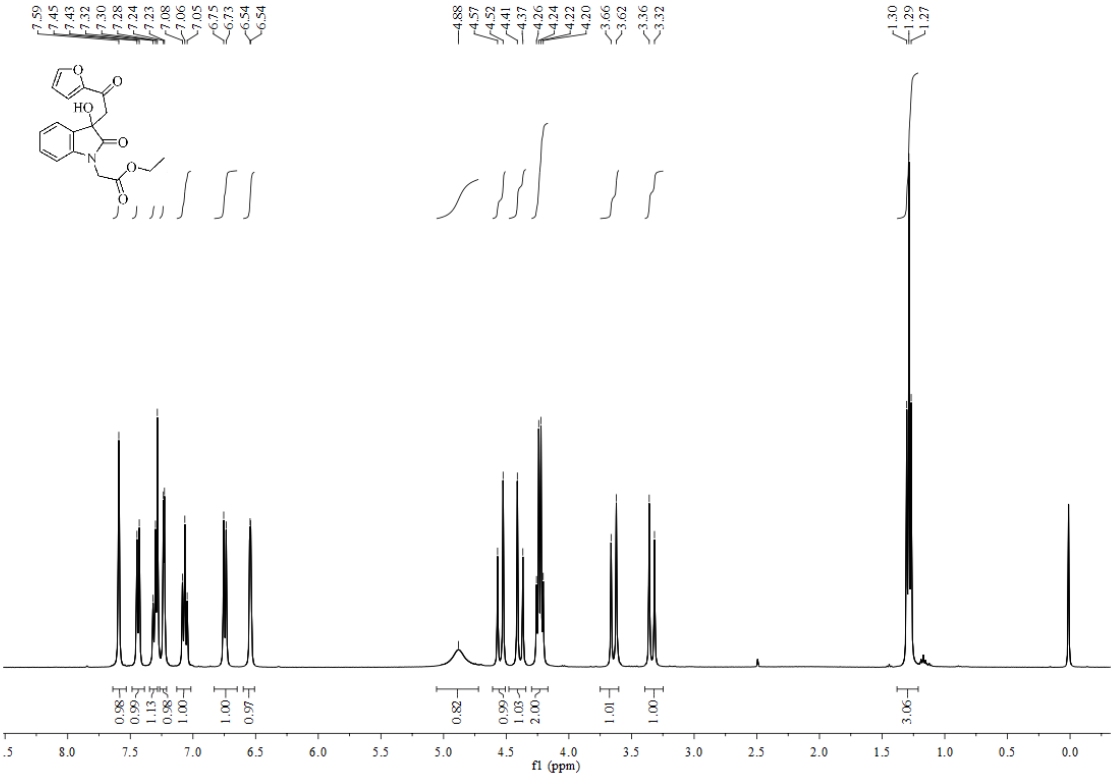

Figure S59. ^{1}H NMR Spectrum of compound **3q**

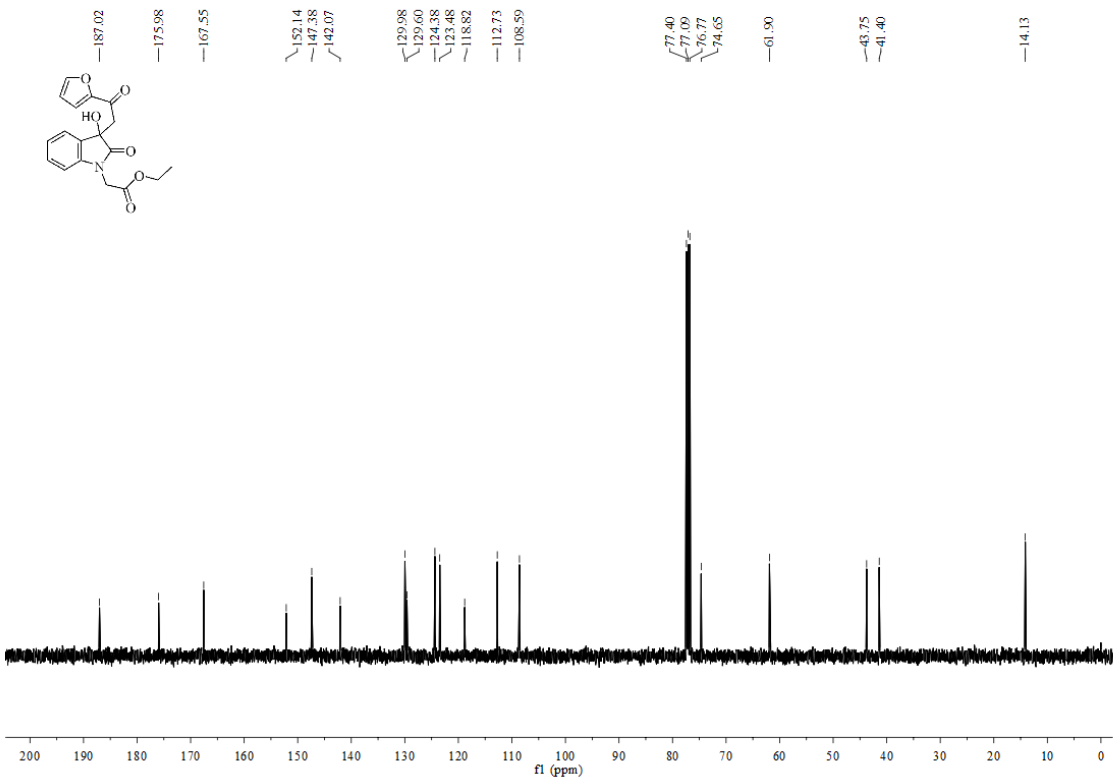

Figure S60. ^{13}C NMR Spectrum of compound **3q**

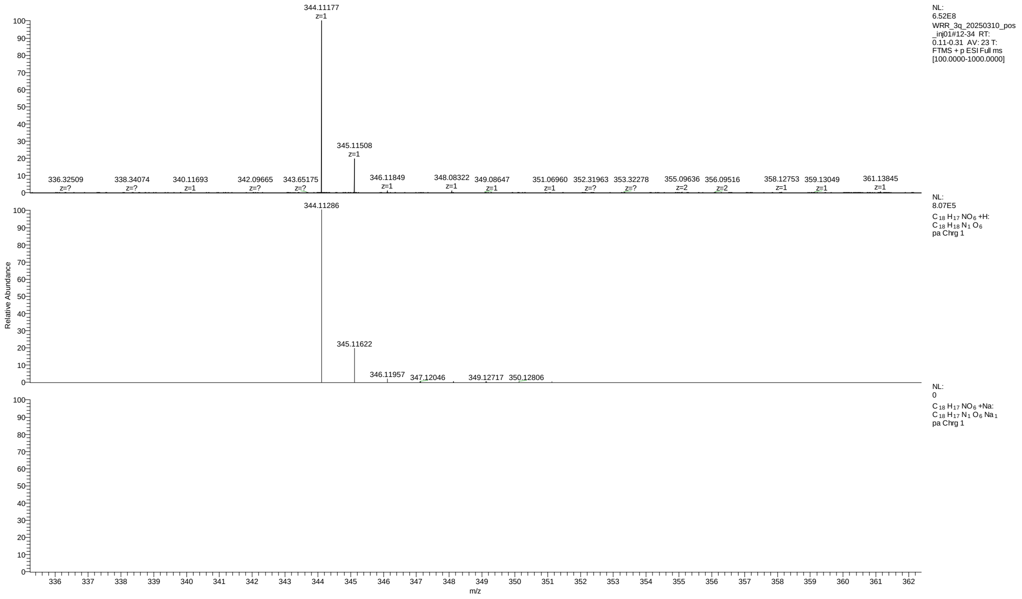

Figure S61. HRMS Spectrum of compound **3q**

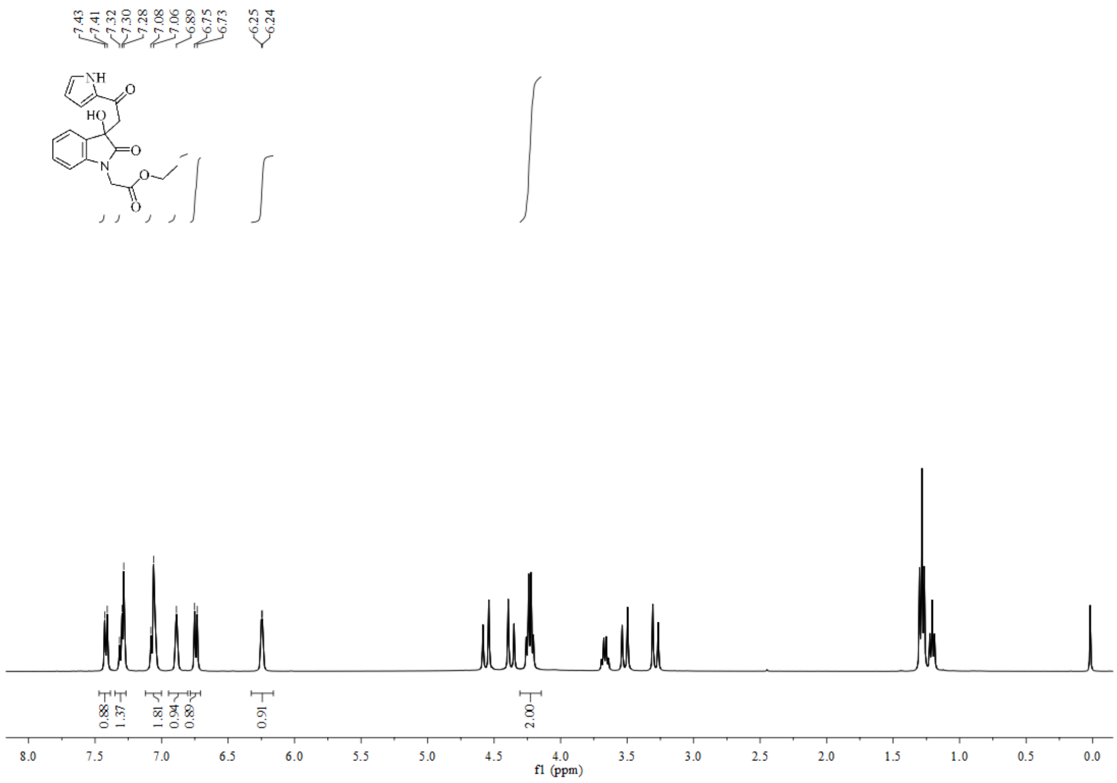

Figure S62. ^{1}H NMR Spectrum of compound **3r**

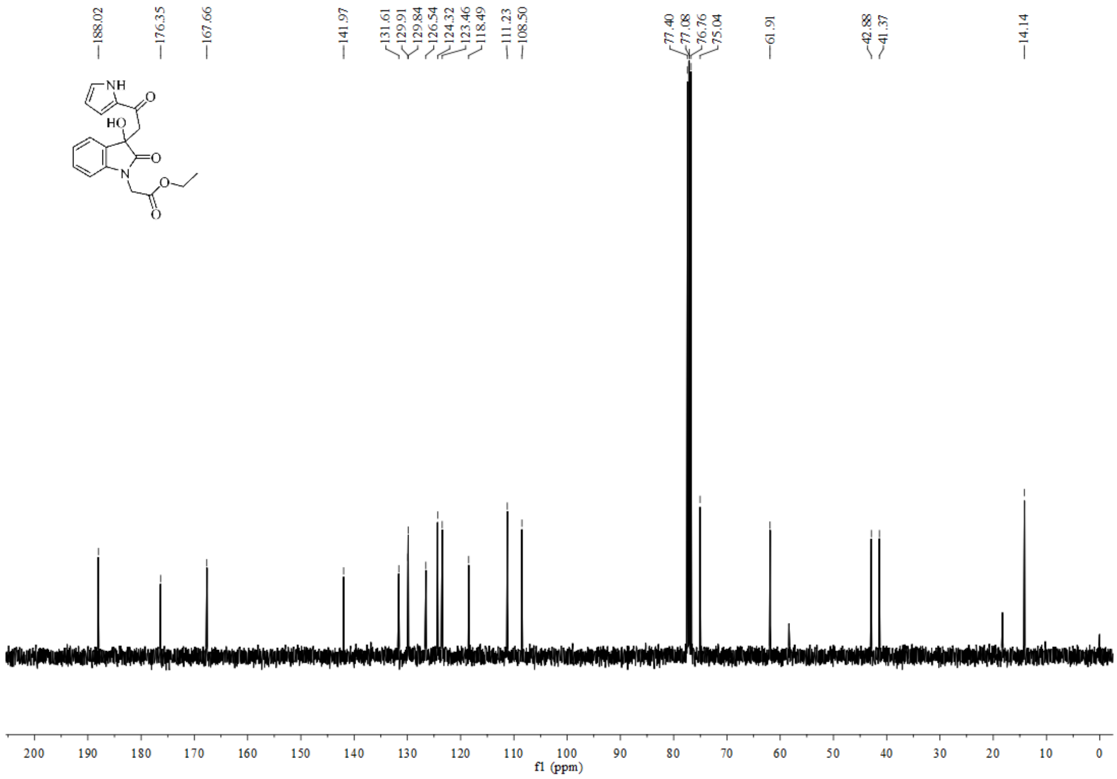

Figure S63. ^{13}C NMR Spectrum of compound **3r**

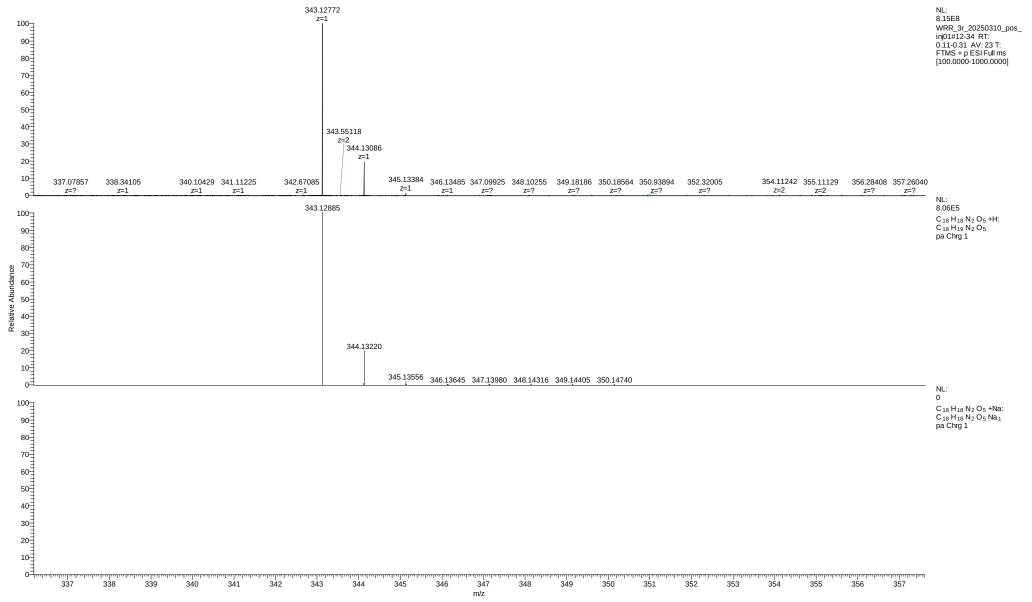

Figure S64. HRMS Spectrum of compound **3r**

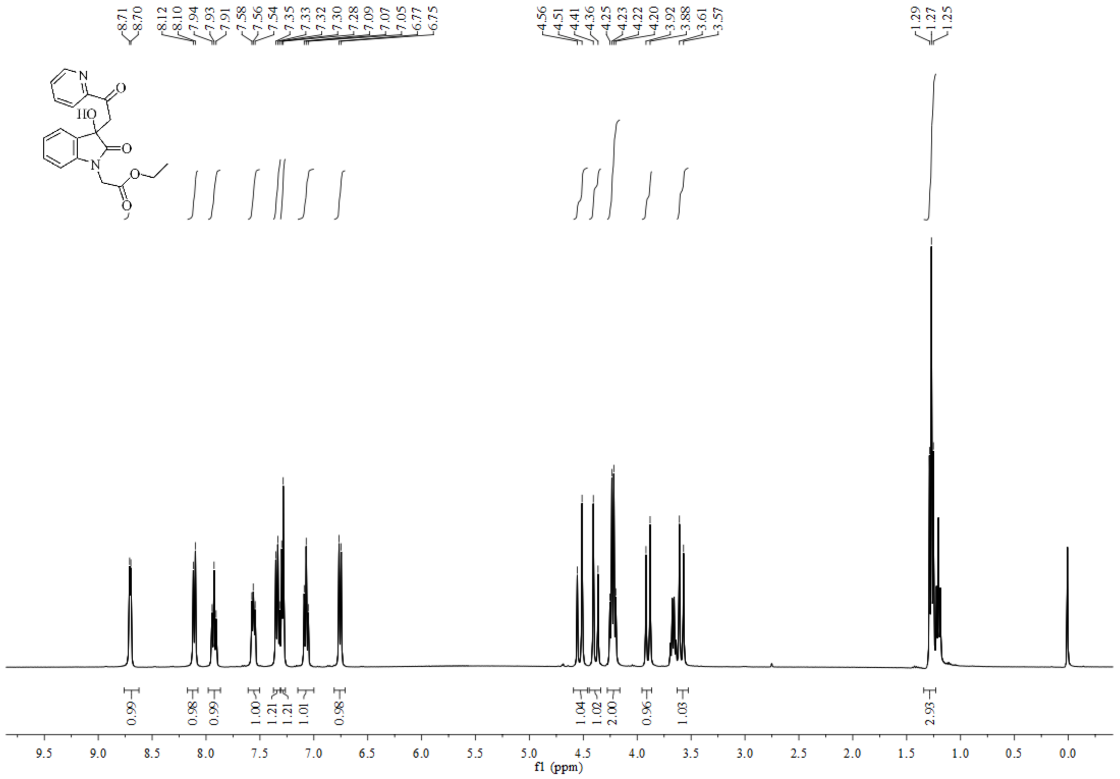

Figure S65. ^{1}H NMR Spectrum of compound **3s**

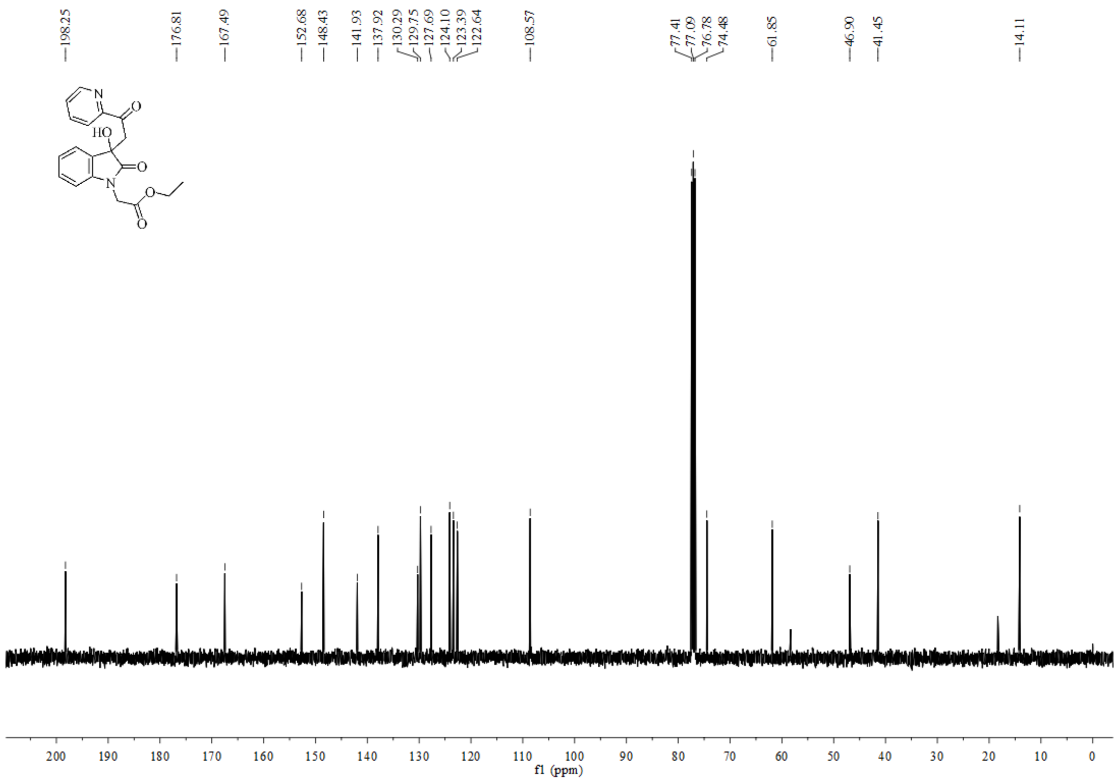

Figure S66. ^{13}C NMR Spectrum of compound **3s**

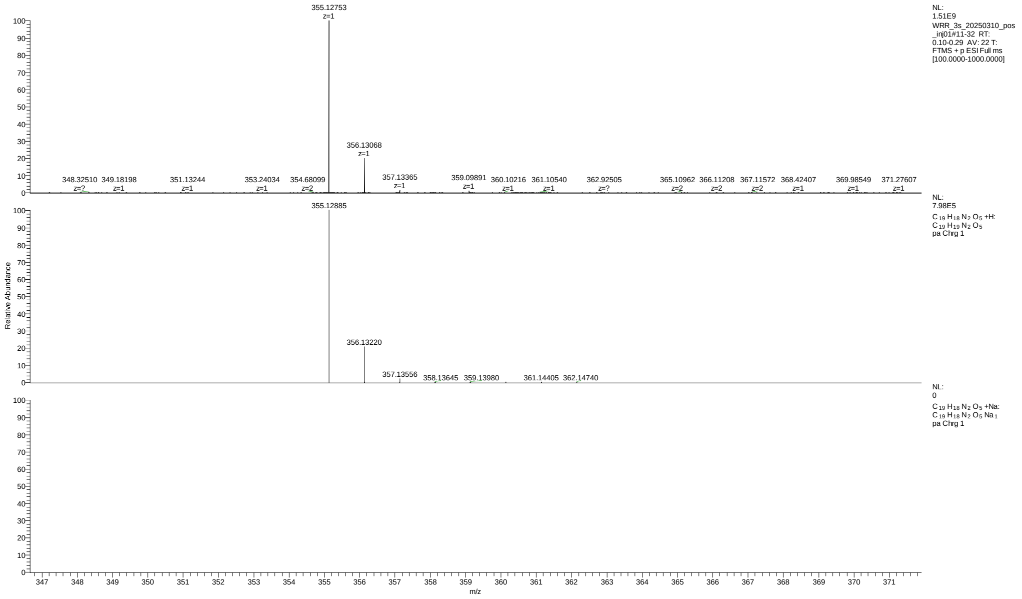

Figure S67. HRMS Spectrum of compound **3s**

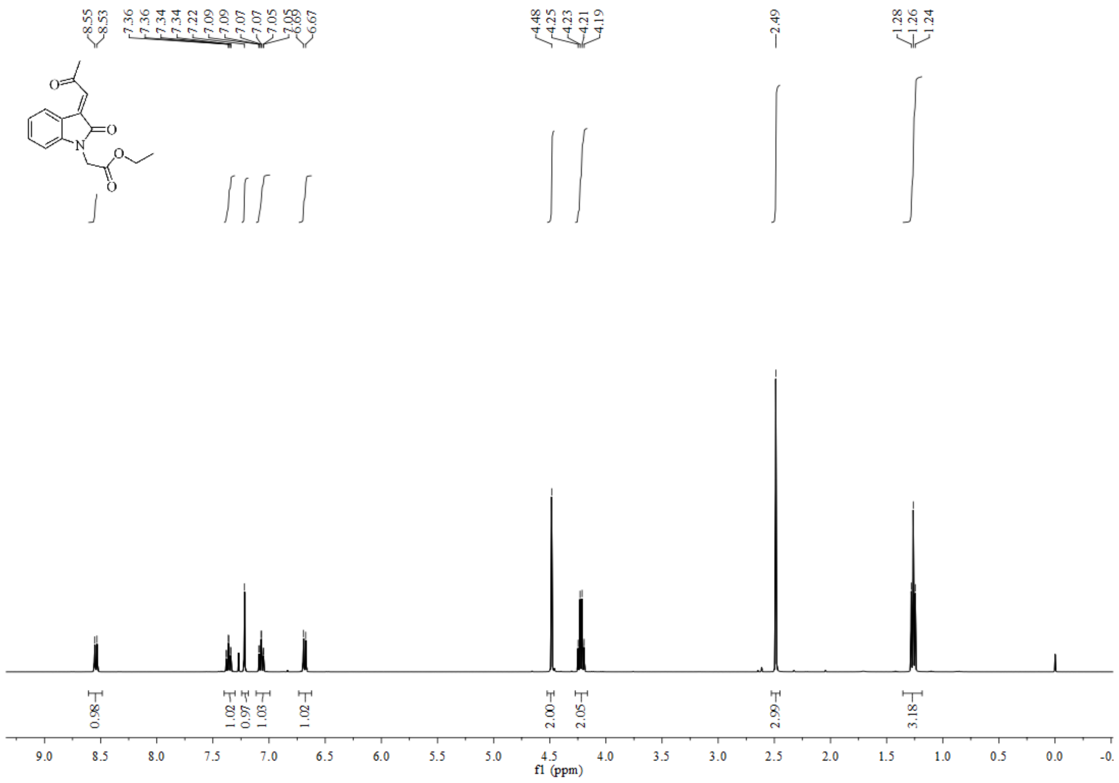

Figure S68. ^{1}H NMR Spectrum of compound **4a**

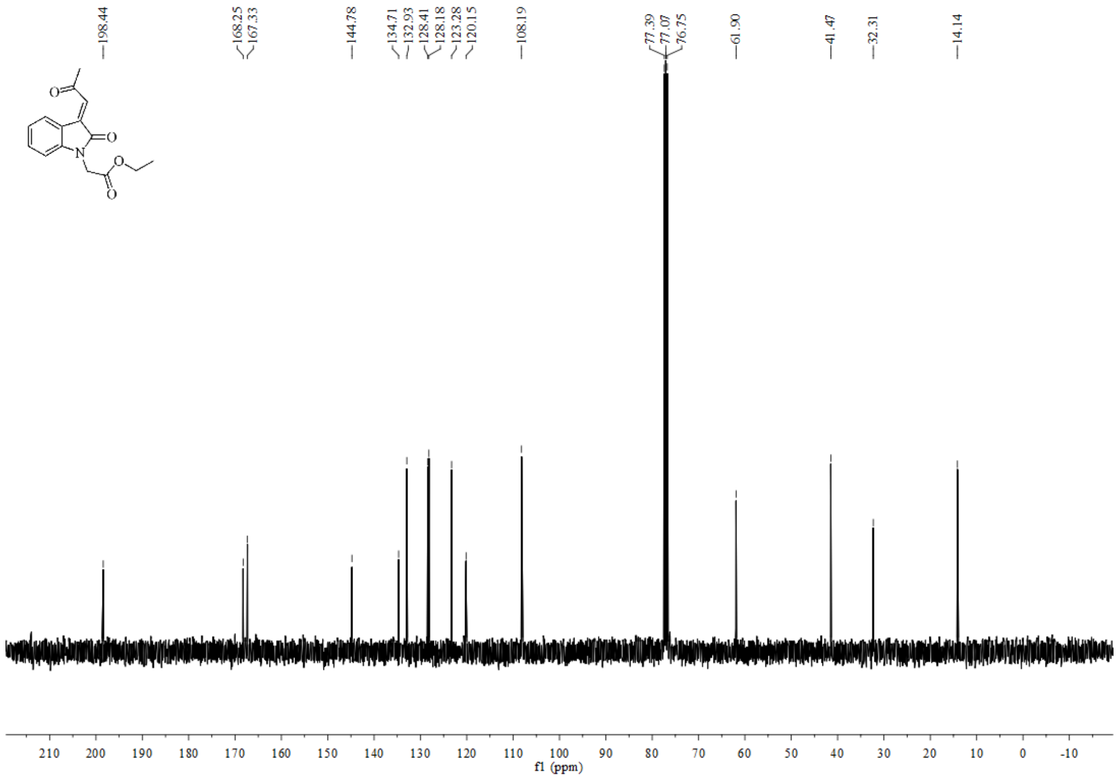

Figure S69. ^{13}C NMR Spectrum of compound **4a**

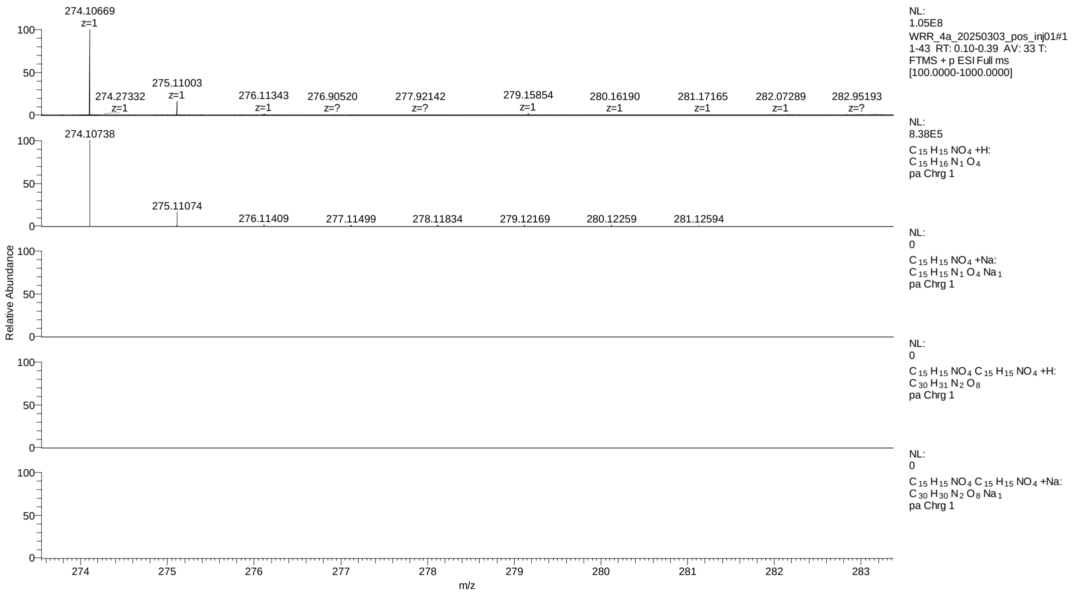

Figure S70. HRMS Spectrum of compound **4a**

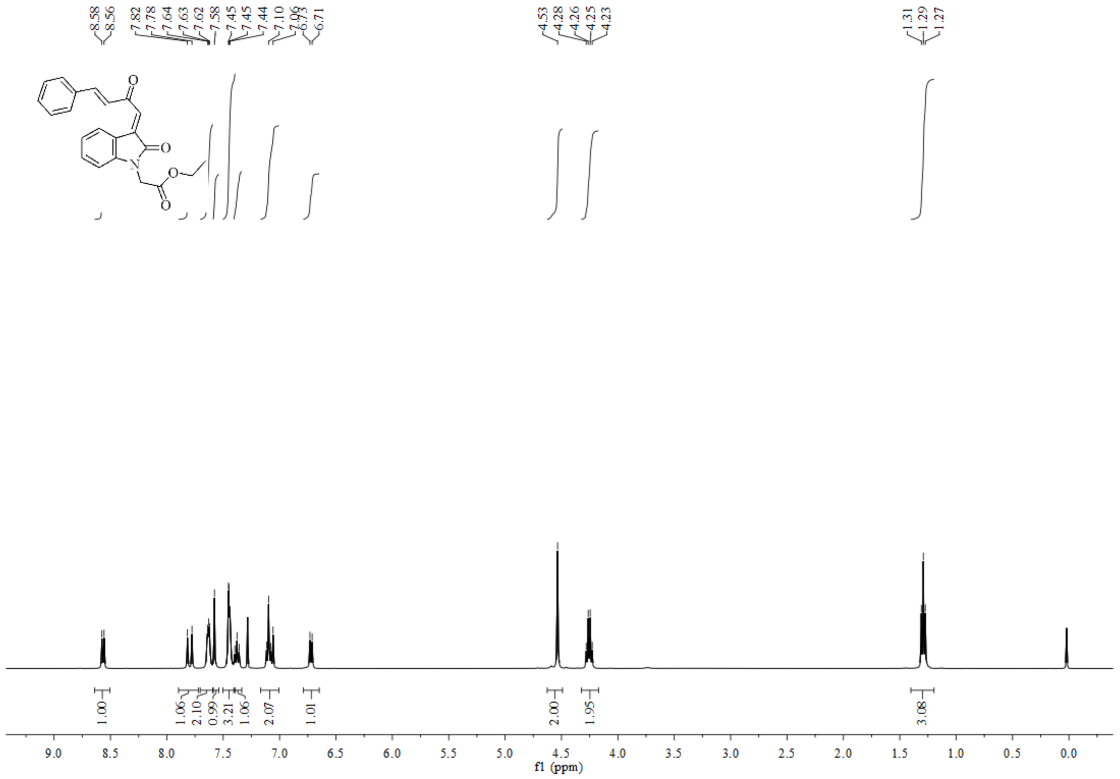

Figure S71. ^{1}H NMR Spectrum of compound **4b**

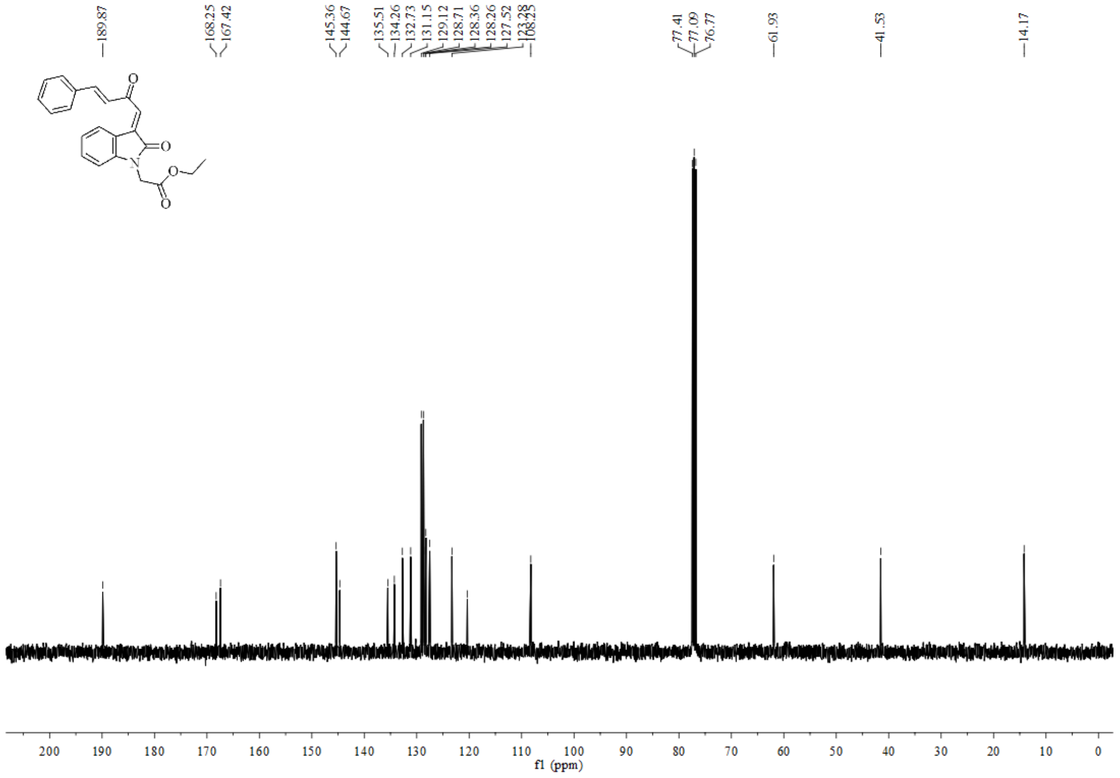

Figure S72. ^{13}C NMR Spectrum of compound **4b**

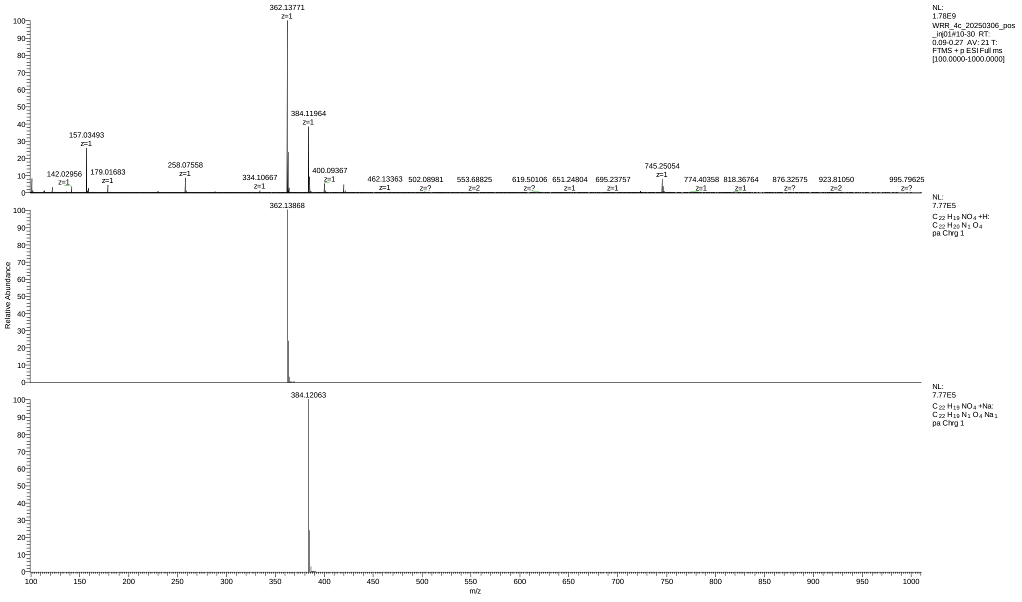

Figure S73. HRMS Spectrum of compound **4b**

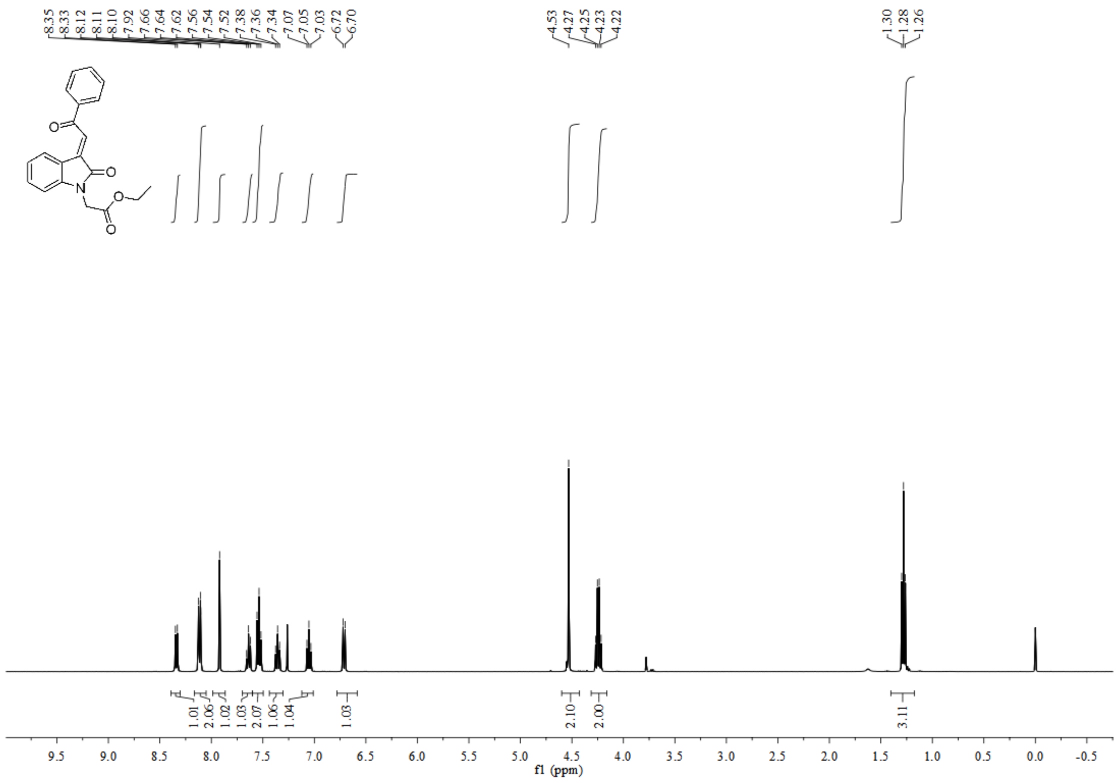

Figure S74. ^{1}H NMR Spectrum of compound **4c**

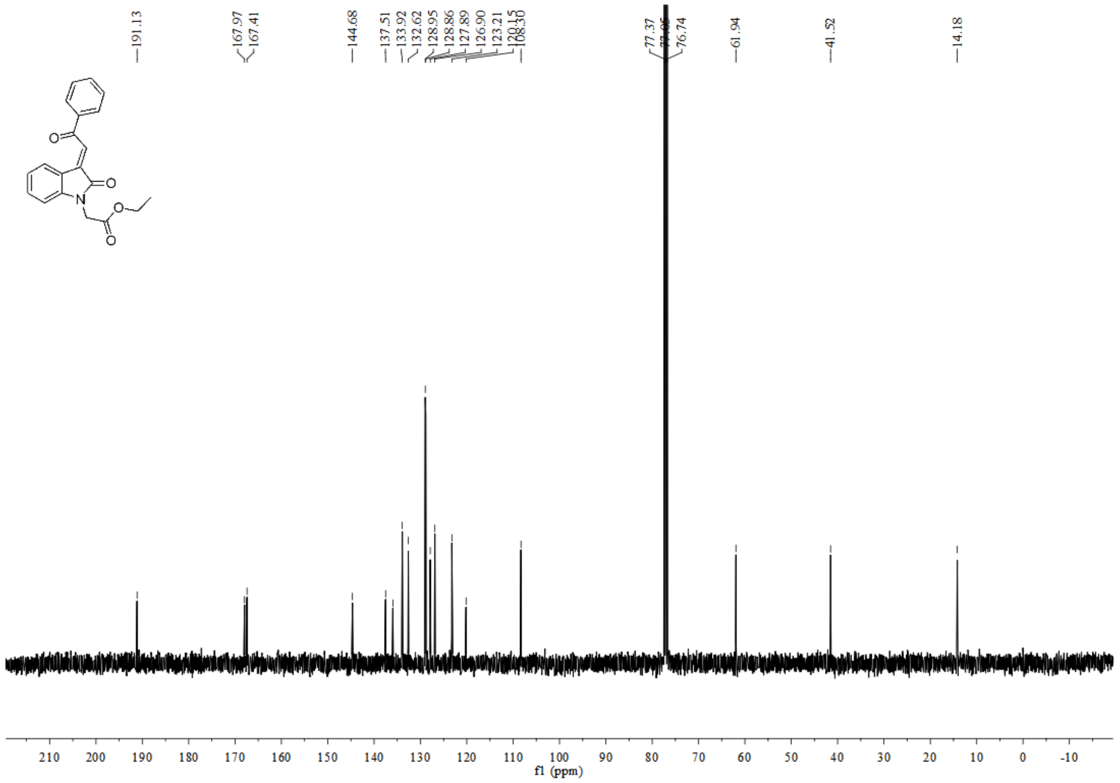

Figure S75. ^{13}C NMR Spectrum of compound **4c**

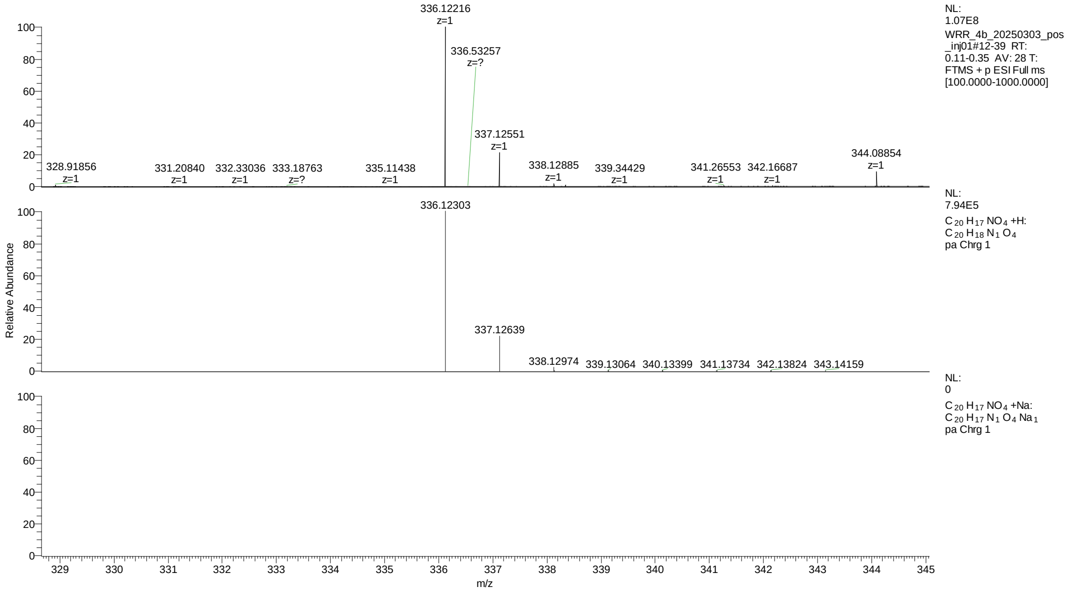

Figure S76. HRMS Spectrum of compound **4c**

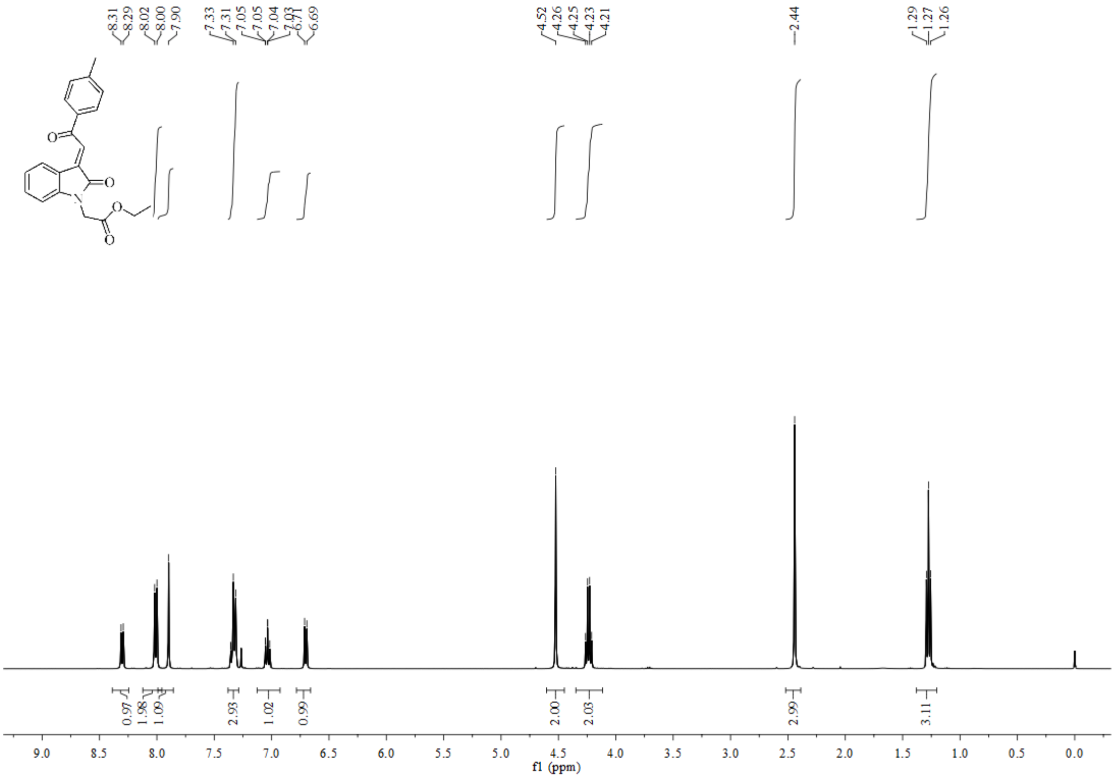

Figure S77. ^{1}H NMR Spectrum of compound **4d**

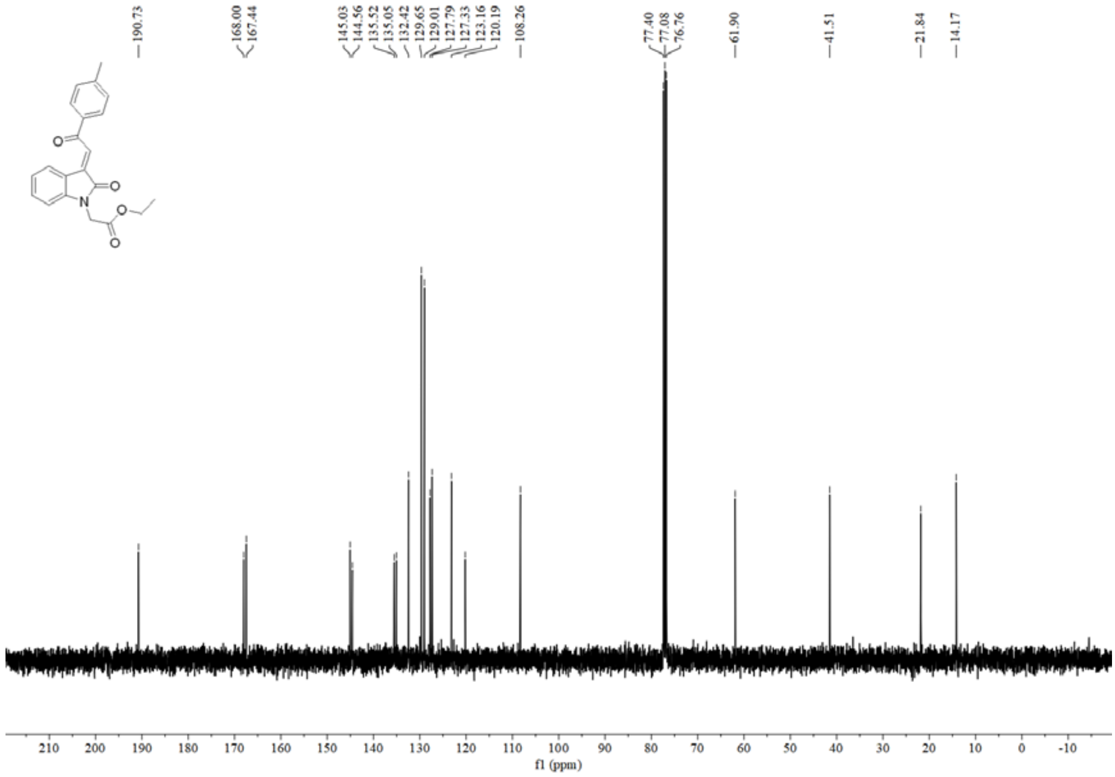

Figure S78. ^{13}C NMR Spectrum of compound **4d**

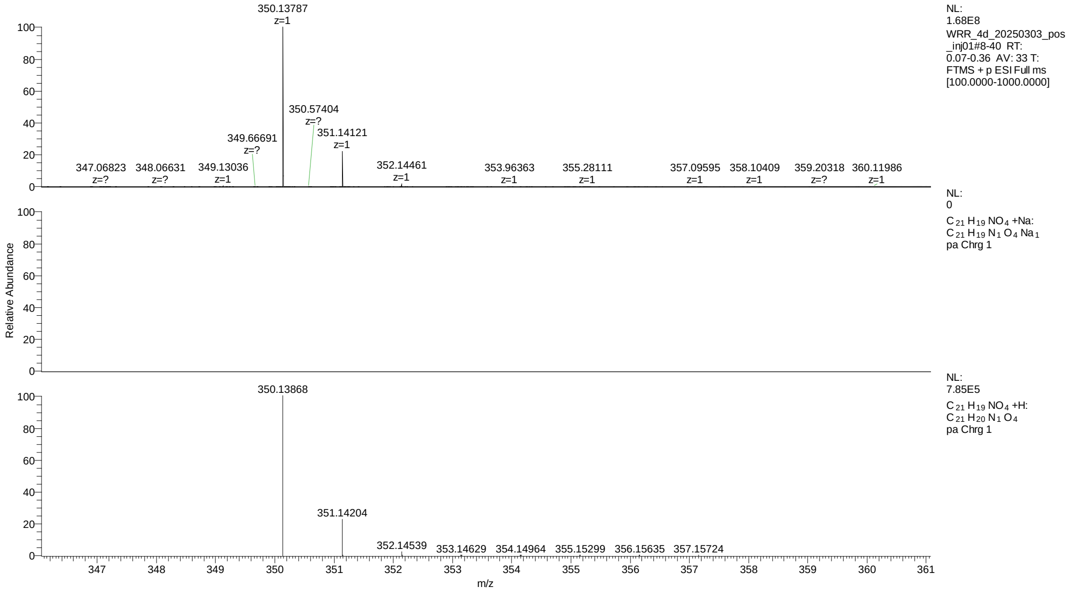

Figure S79. HRMS Spectrum of compound **4d**

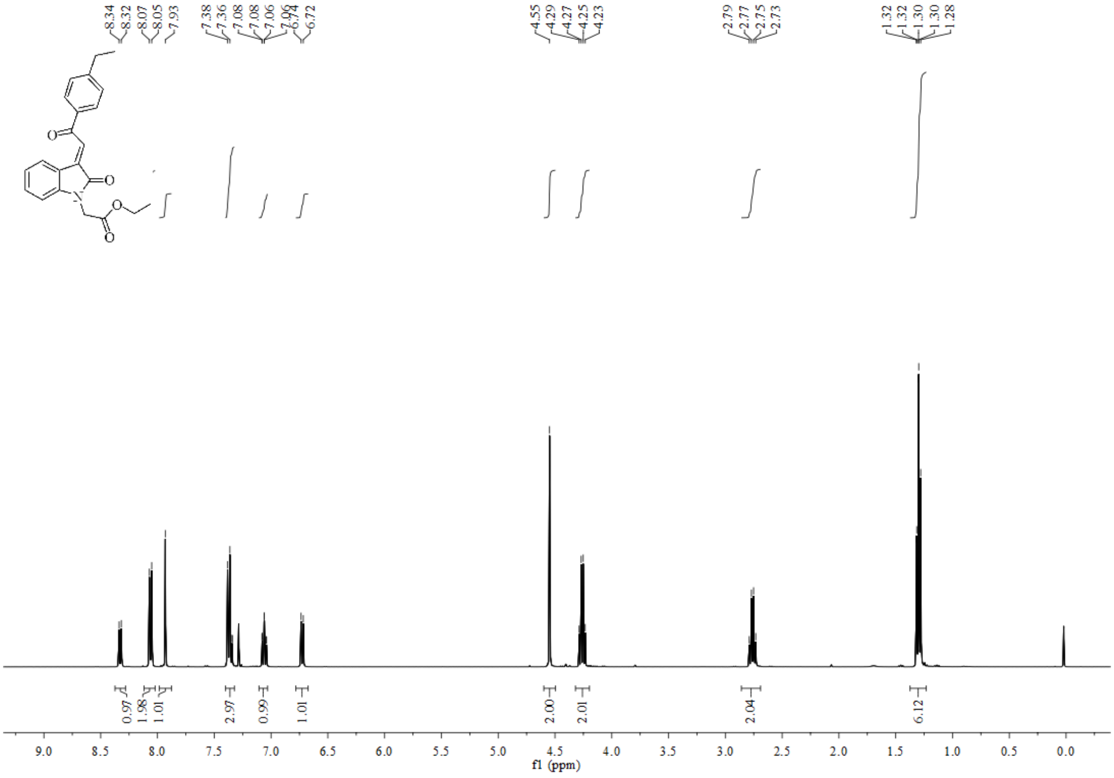

Figure S80. ^{1}H NMR Spectrum of compound **4e**

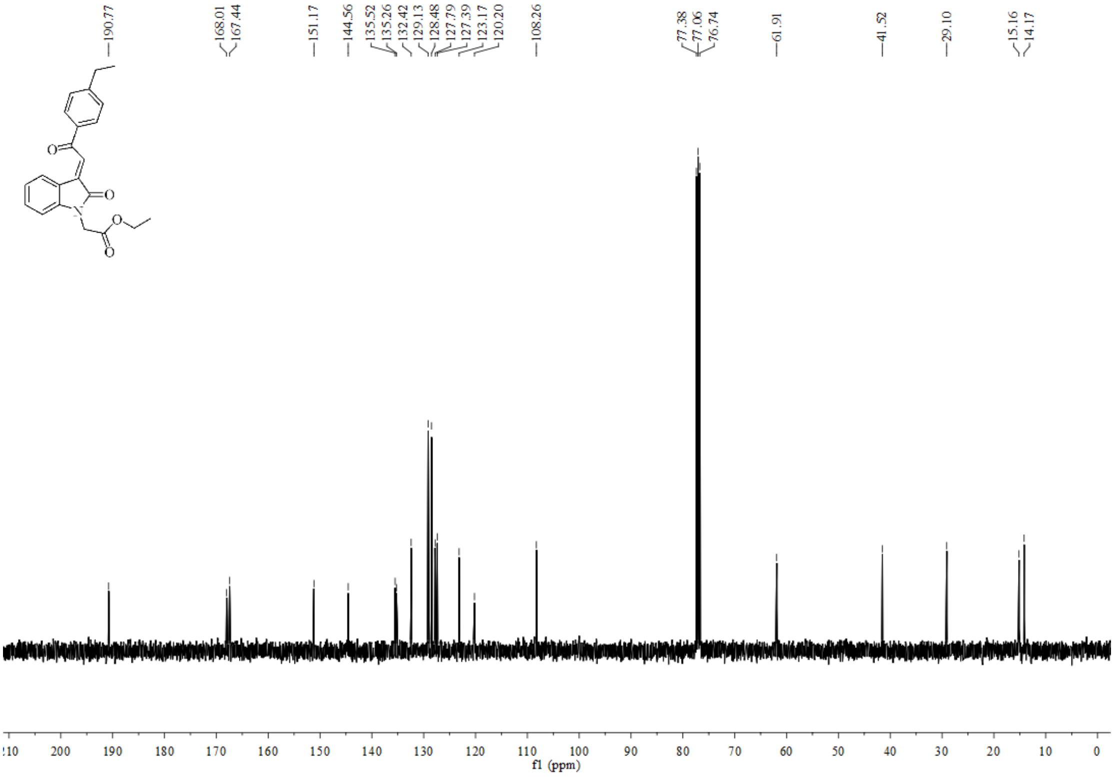

Figure S81. ^{13}C NMR Spectrum of compound **4e**

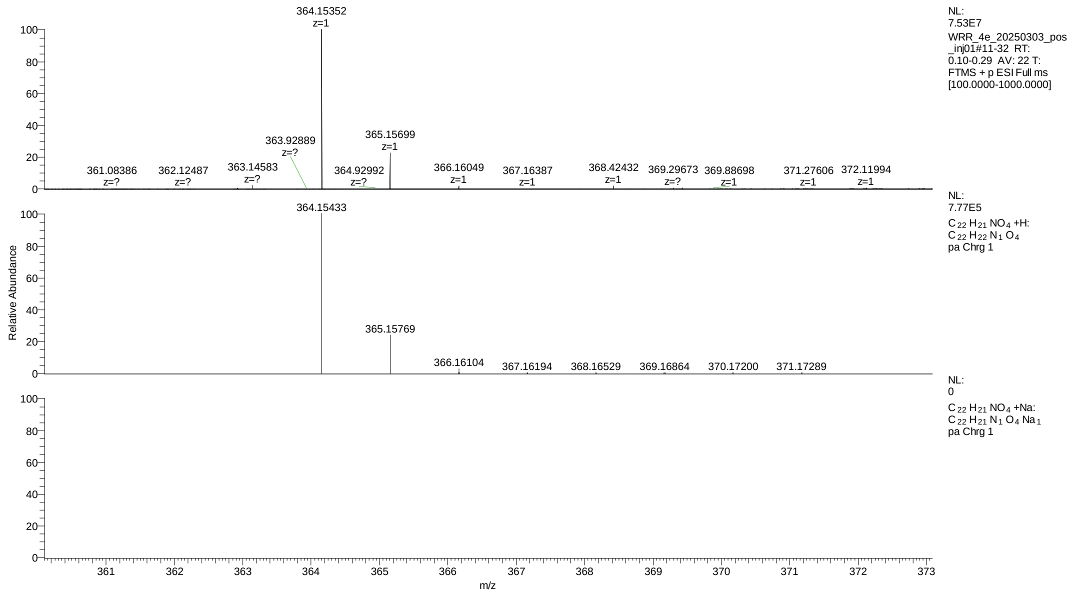

Figure S82. HRMS Spectrum of compound **4e**

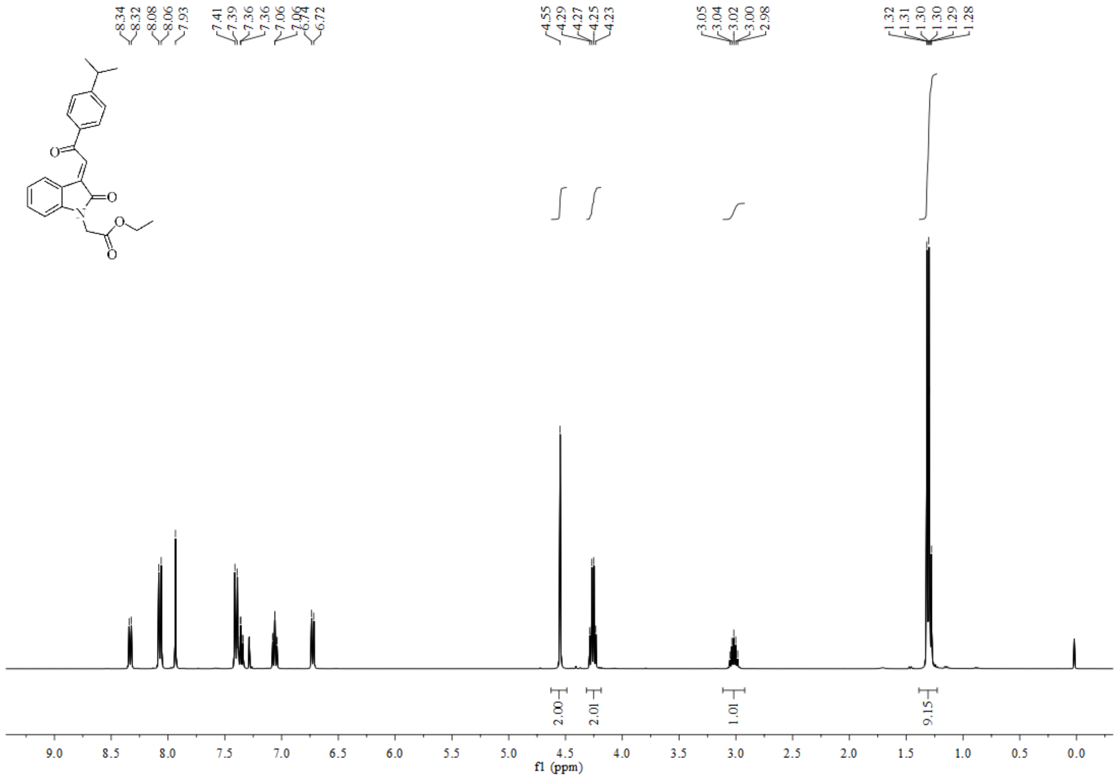

Figure S83. ^{1}H NMR Spectrum of compound **4f**

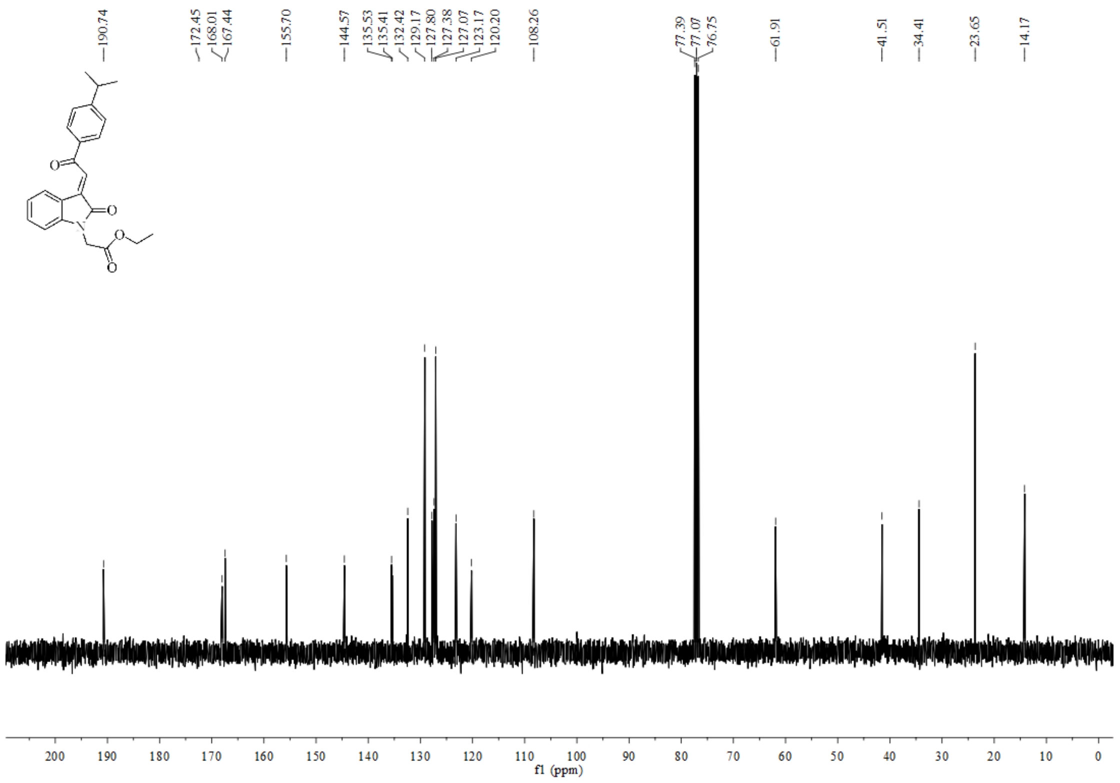

Figure S84. ^{13}C NMR Spectrum of compound **4f**

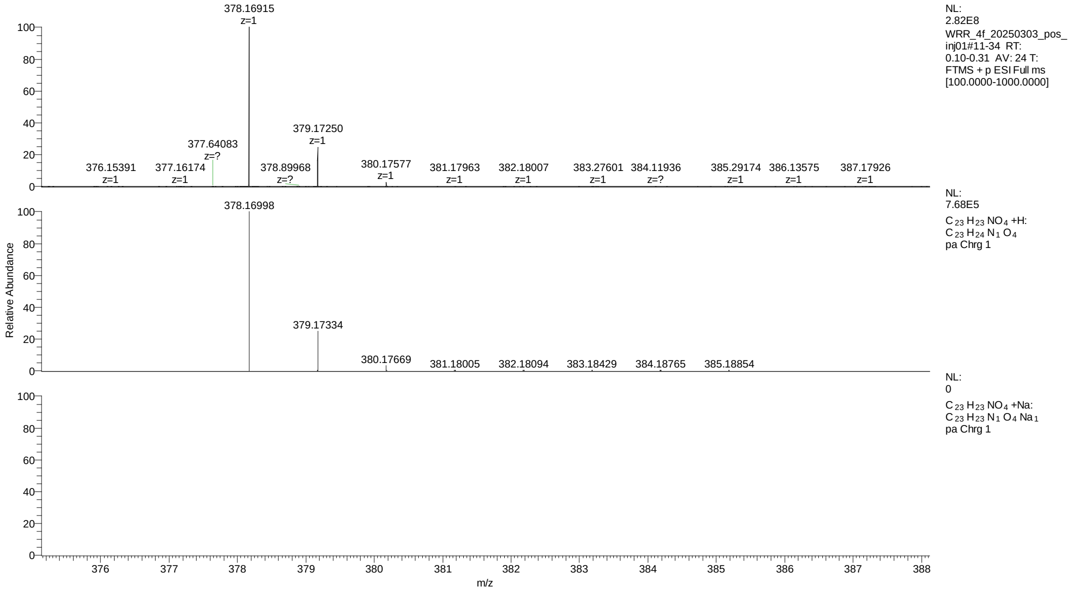

Figure S85. HRMS Spectrum of compound **4f**

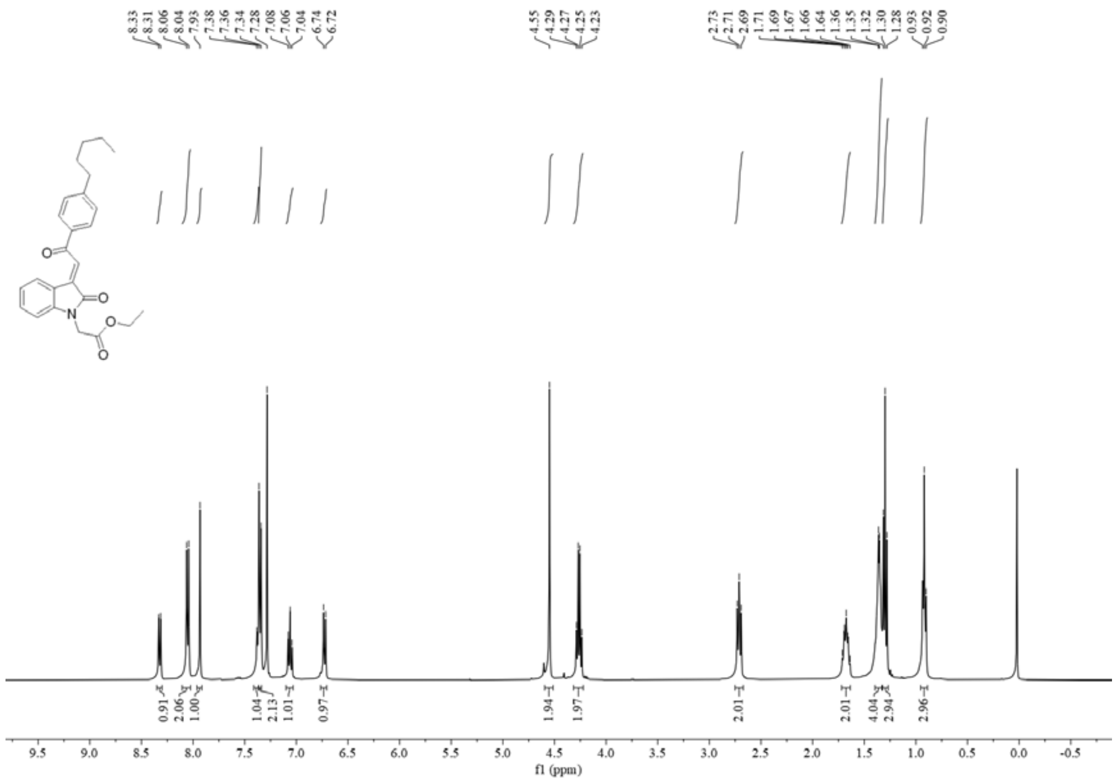

Figure S86. ^{1}H NMR Spectrum of compound **4g**

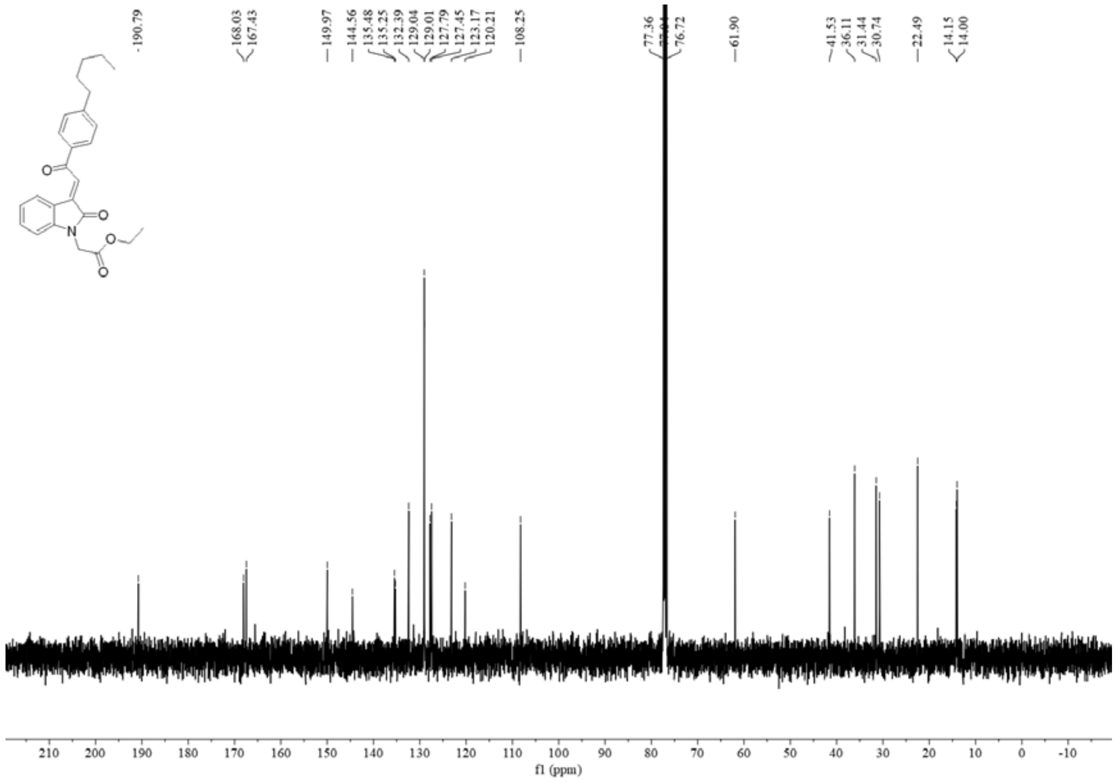

Figure S87. ^{13}C NMR Spectrum of compound **4g**

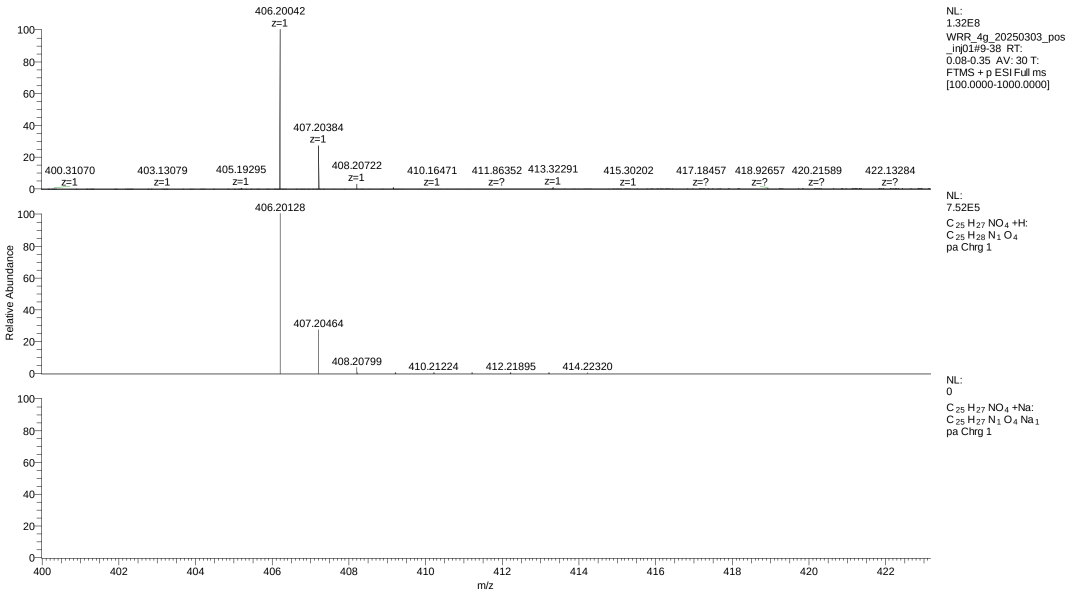

Figure S88. HRMS Spectrum of compound **4g**

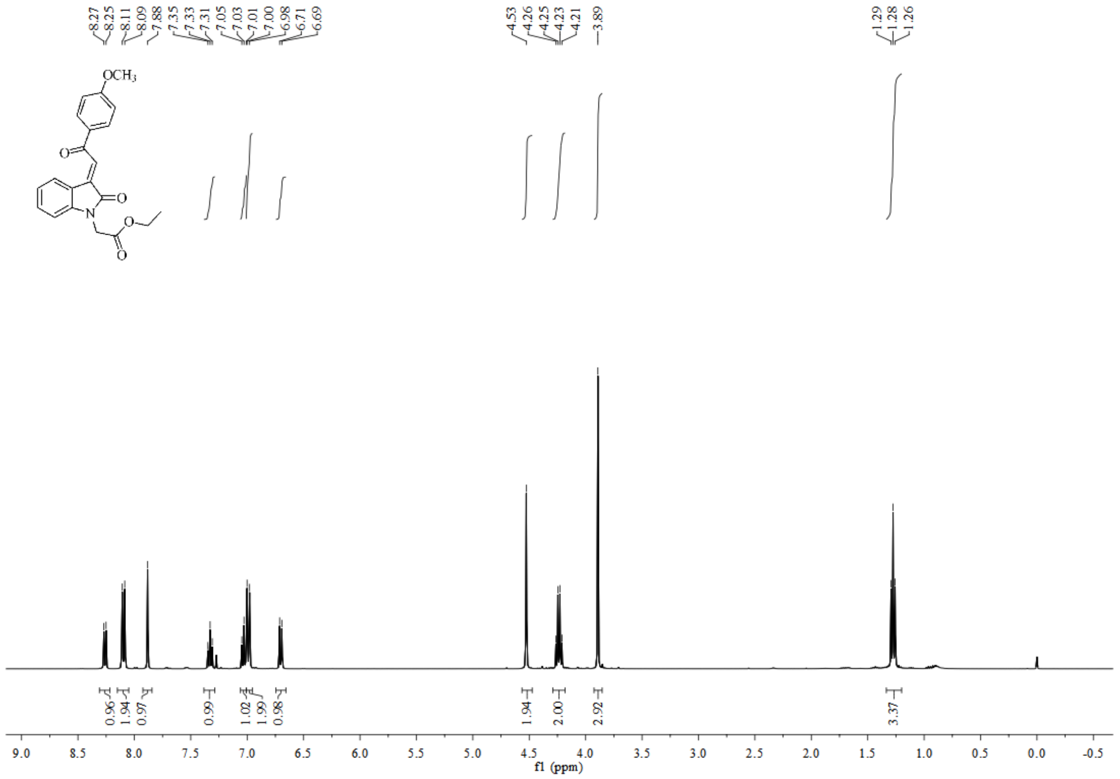

Figure S89. ^{1}H NMR Spectrum of compound **4h**

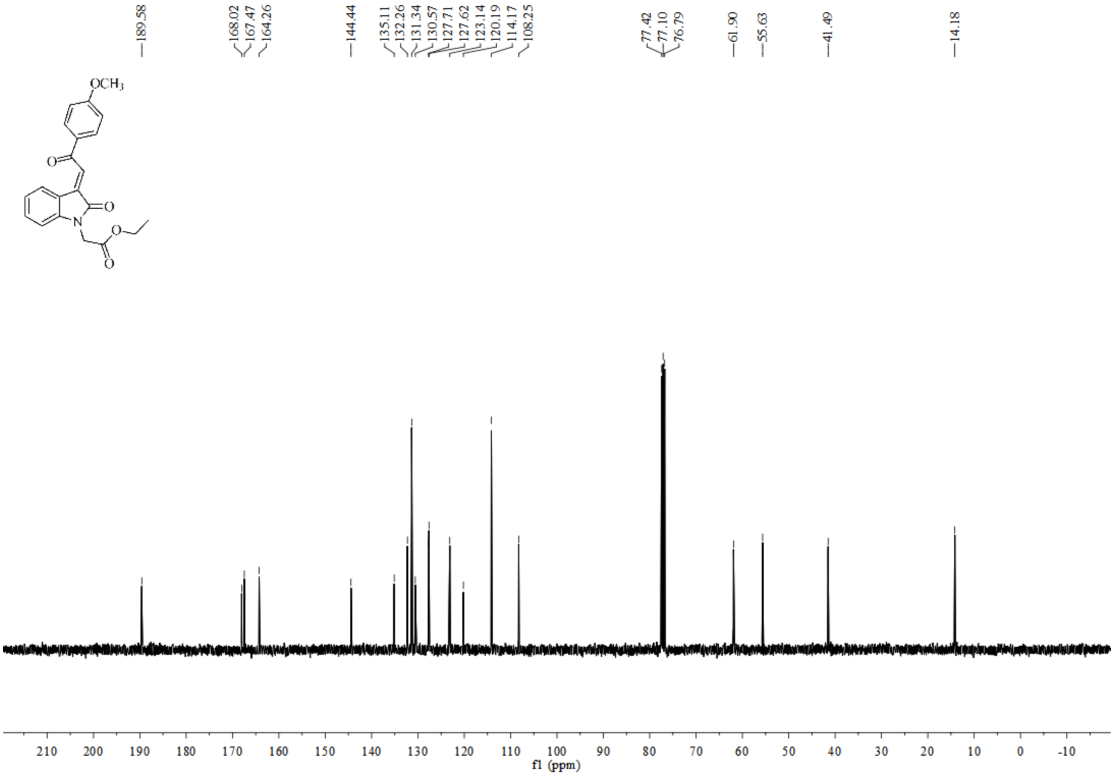

Figure S90. ^{13}C NMR Spectrum of compound **4h**

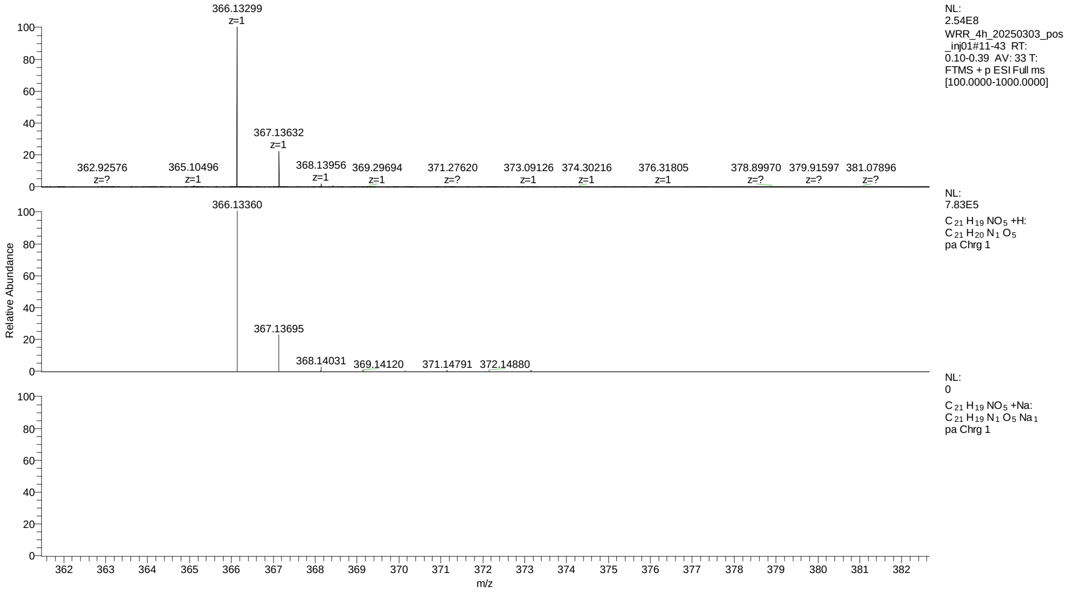

Figure S91. HRMS Spectrum of compound **4h**

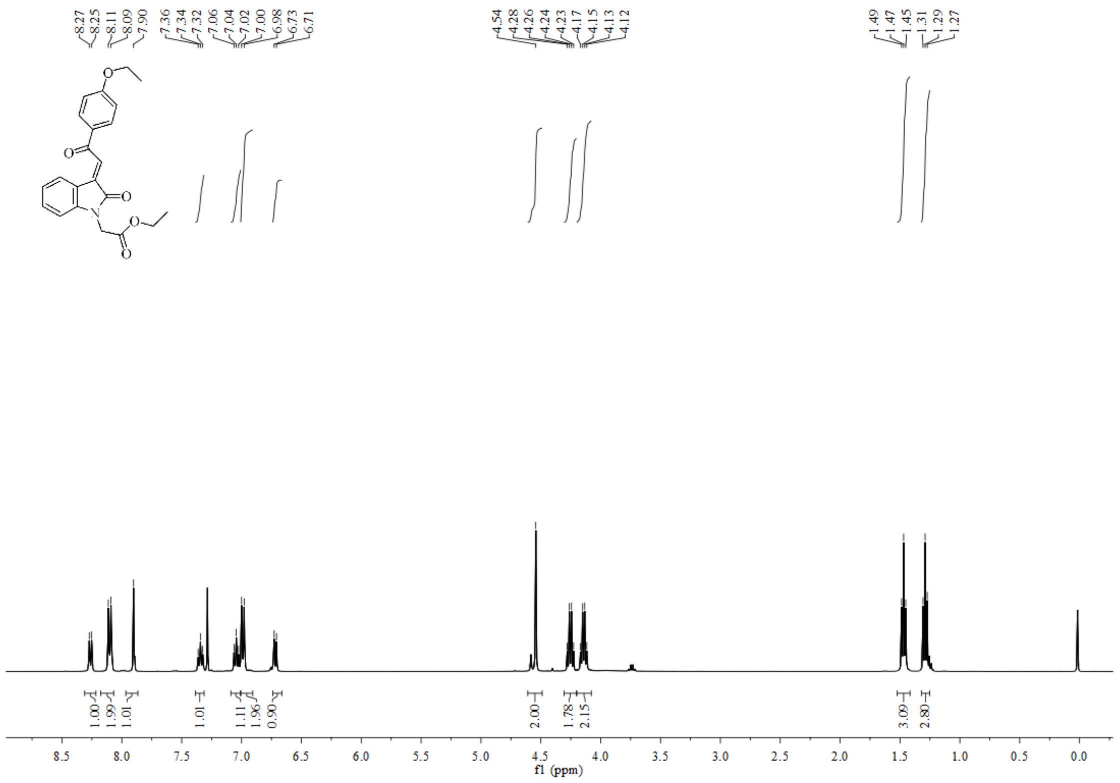

Figure S92. ^{1}H NMR Spectrum of compound **4i**

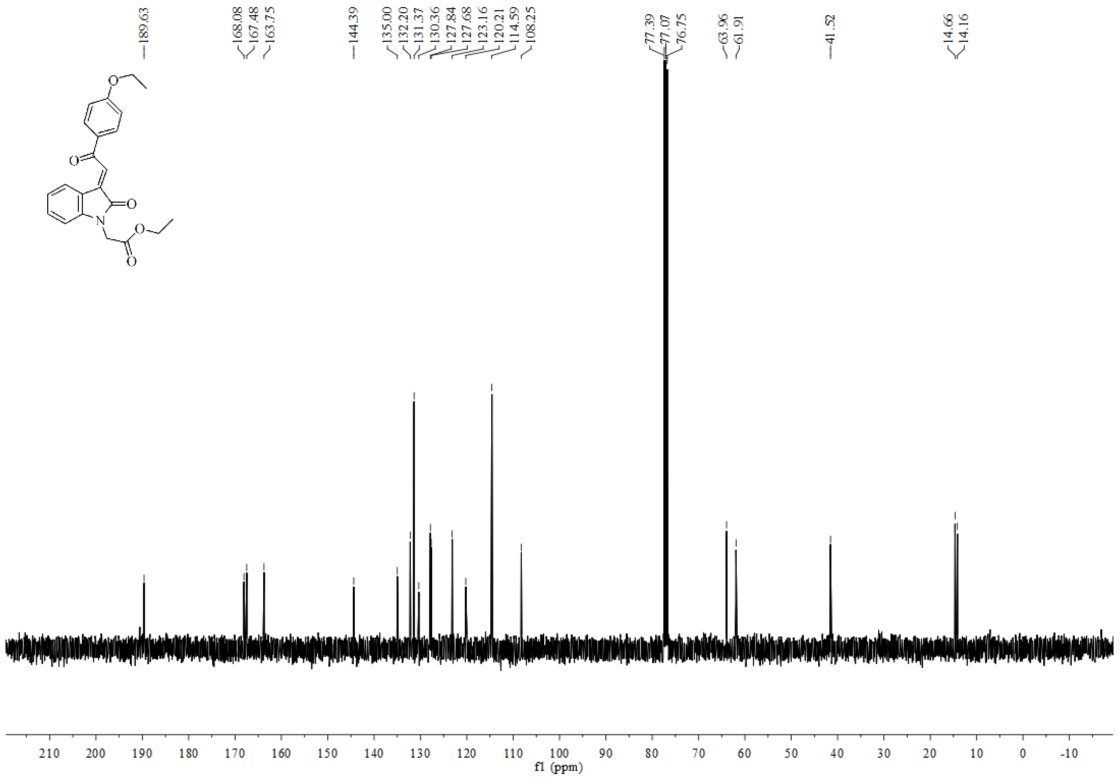

Figure S93. ^{13}C NMR Spectrum of compound **4i**

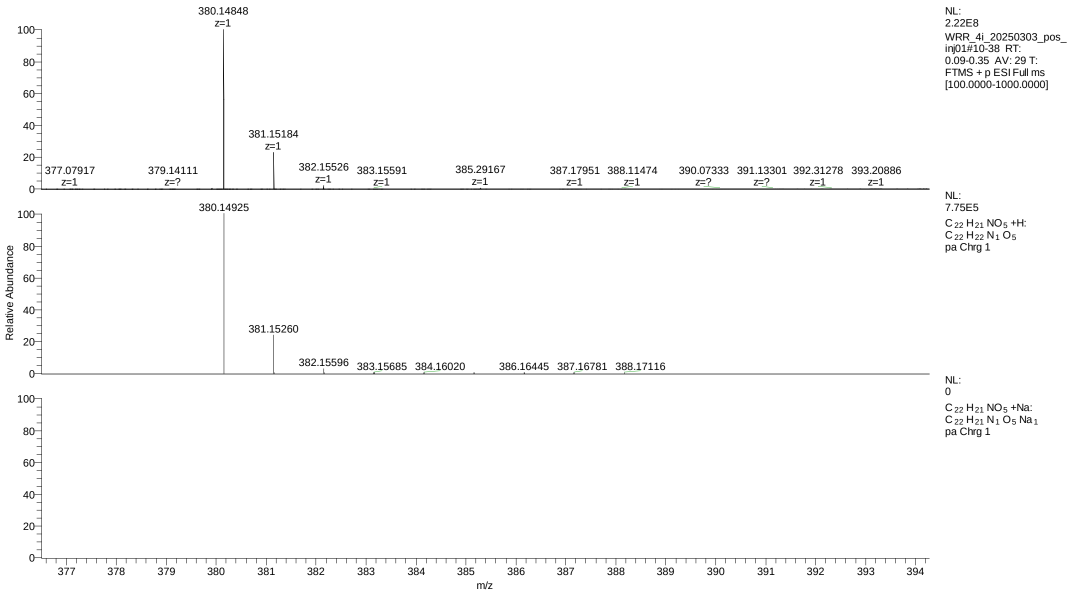

Figure S94. HRMS Spectrum of compound **4i**

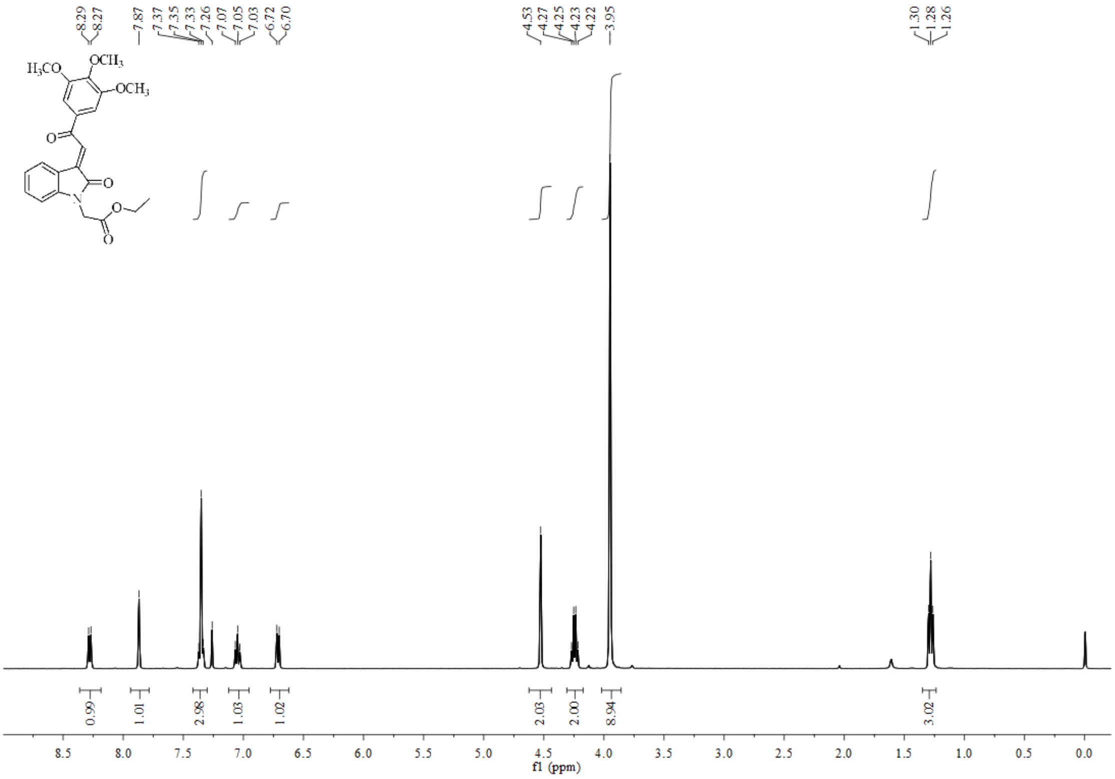

Figure S95. ^{1}H NMR Spectrum of compound **4j**

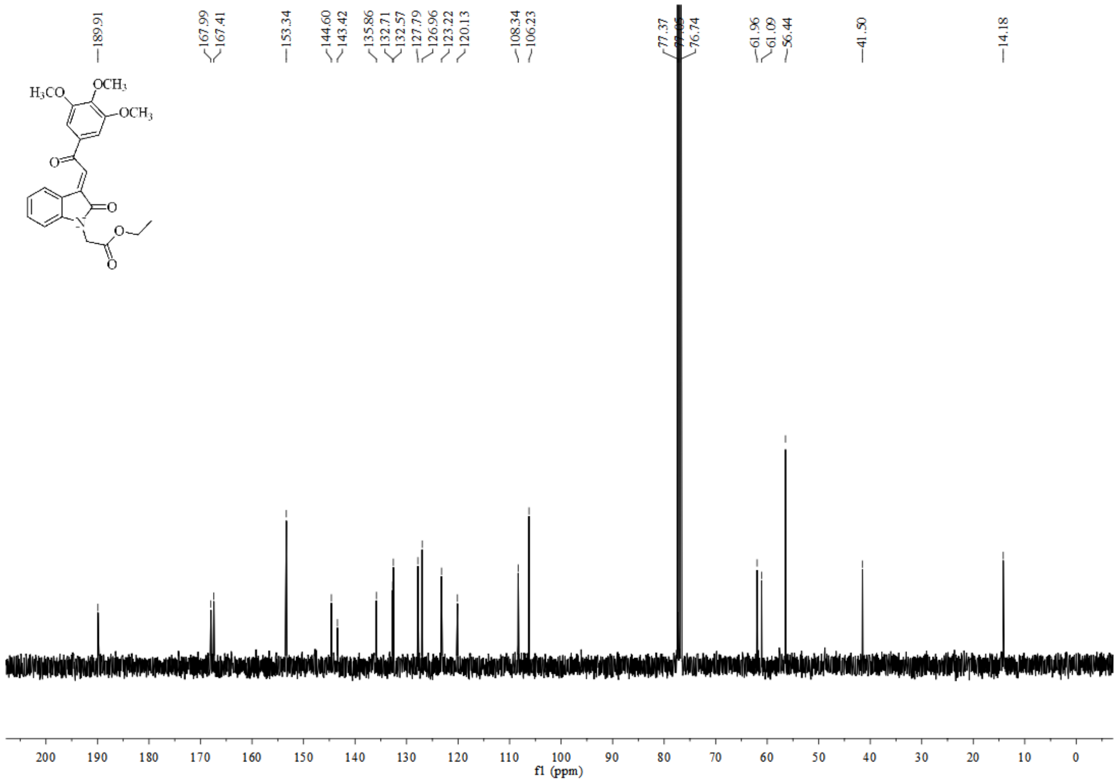

Figure S96. ^{13}C NMR Spectrum of compound **4j**

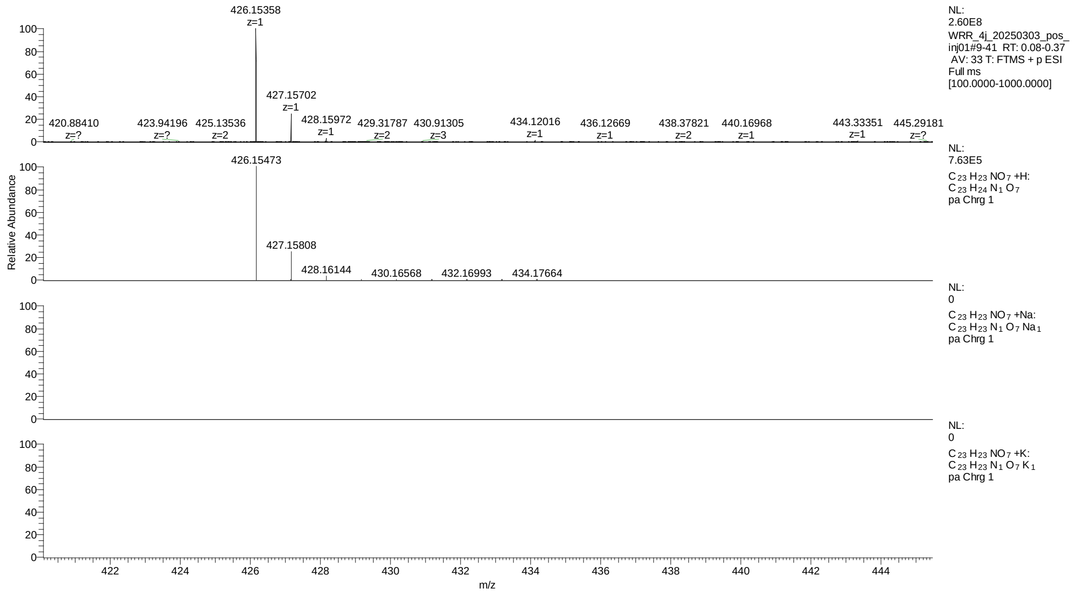

Figure S97. HRMS Spectrum of compound **4j**

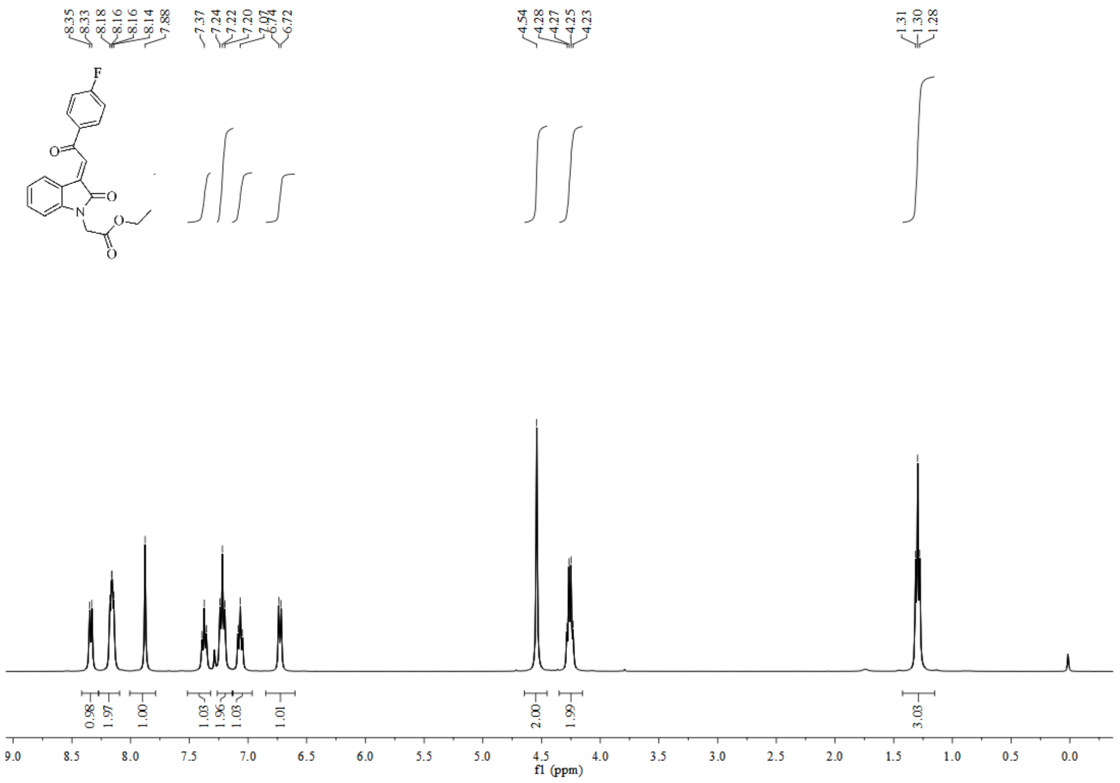

Figure S98. ^{1}H NMR Spectrum of compound **4k**

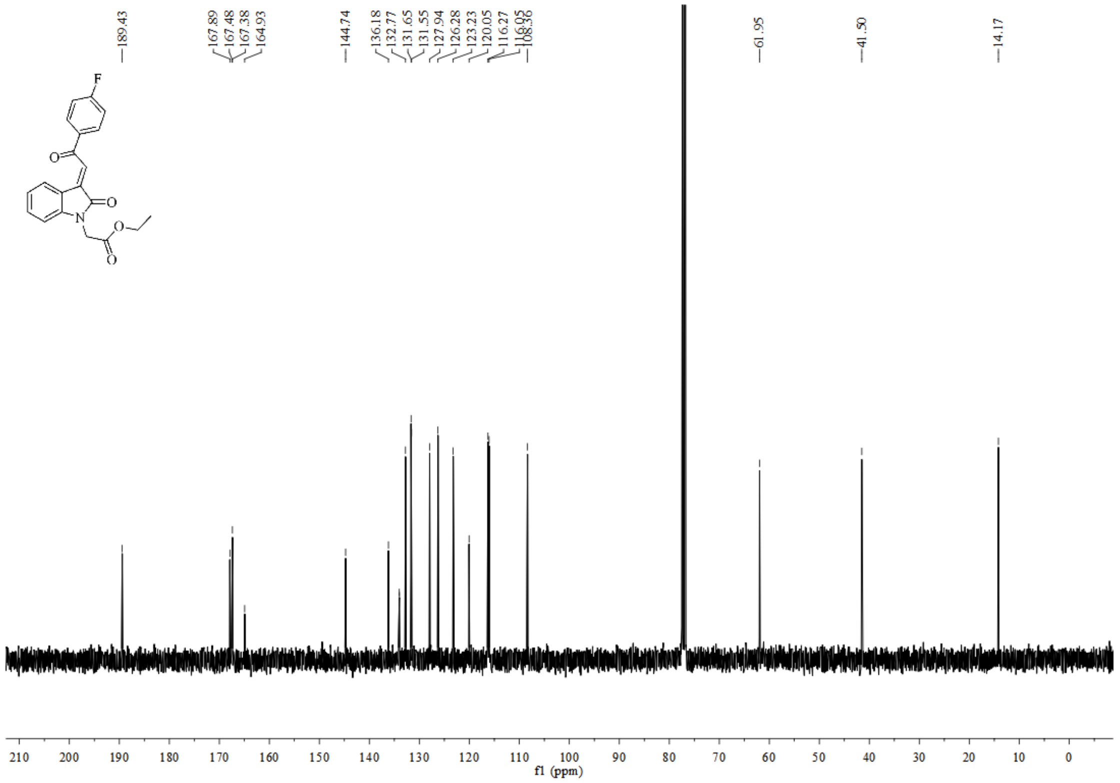

Figure S99. ^{13}C NMR Spectrum of compound **4k**

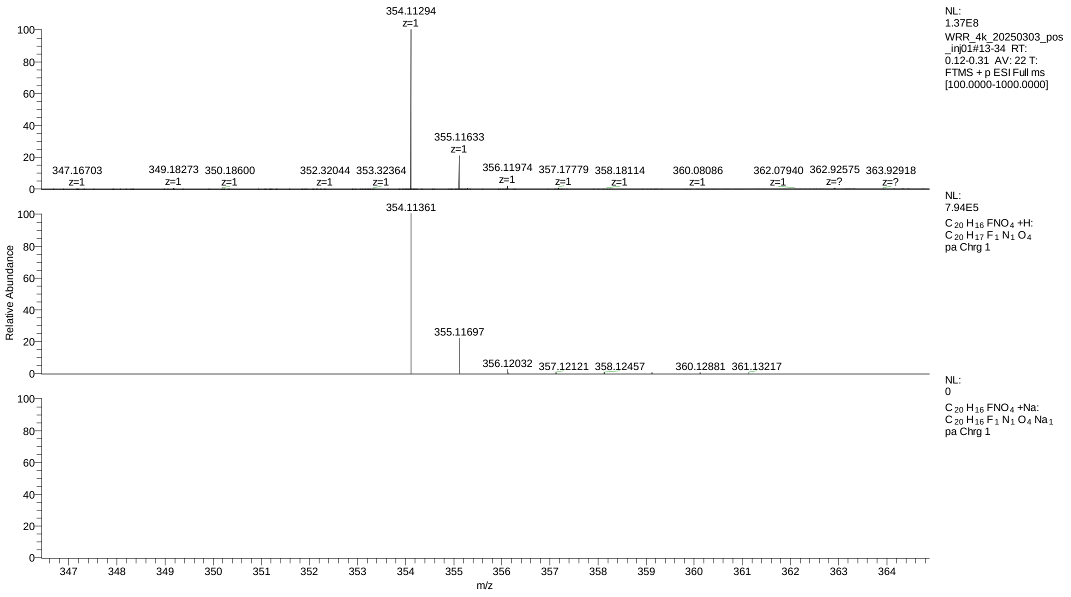

Figure S100. HRMS Spectrum of compound **4k**

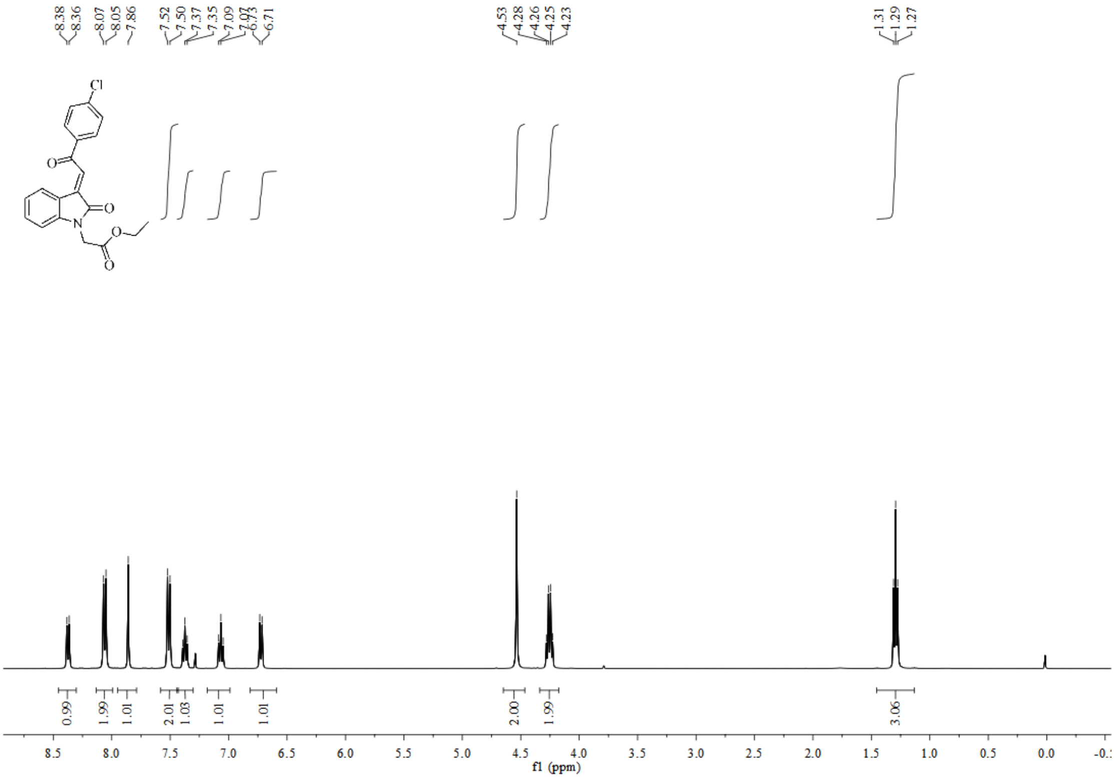

Figure S101. ^{1}H NMR Spectrum of compound **4l**

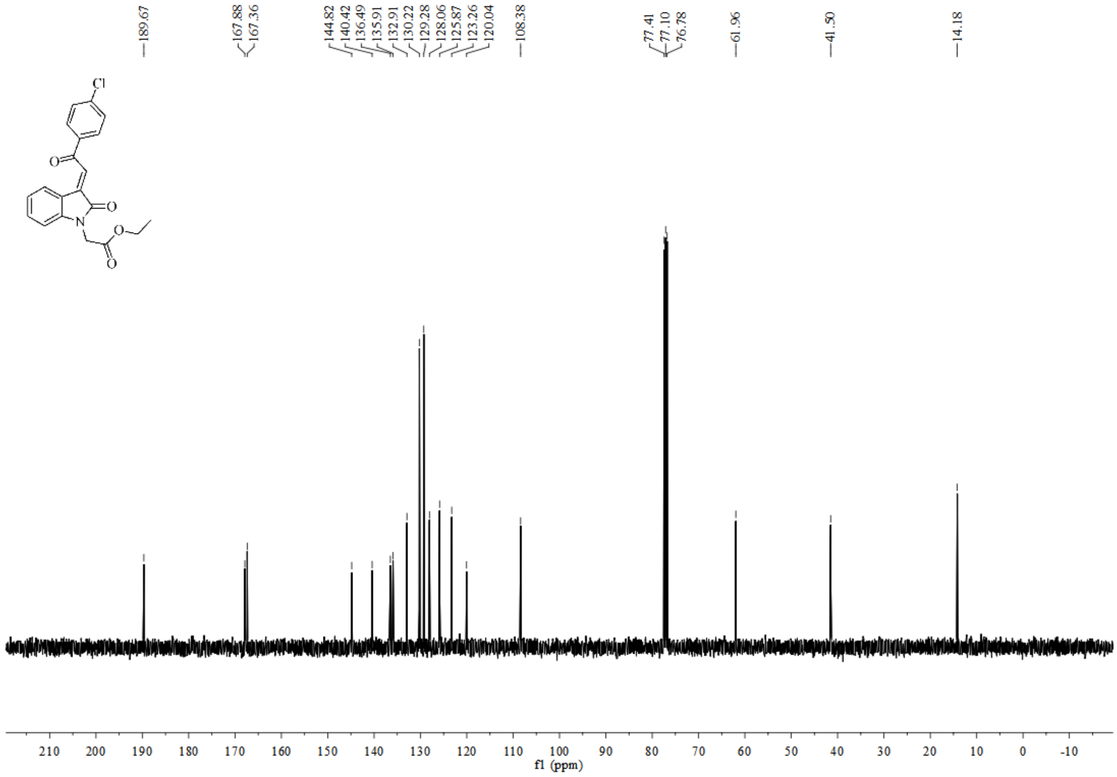

Figure S102. ^{13}C NMR Spectrum of compound **4l**

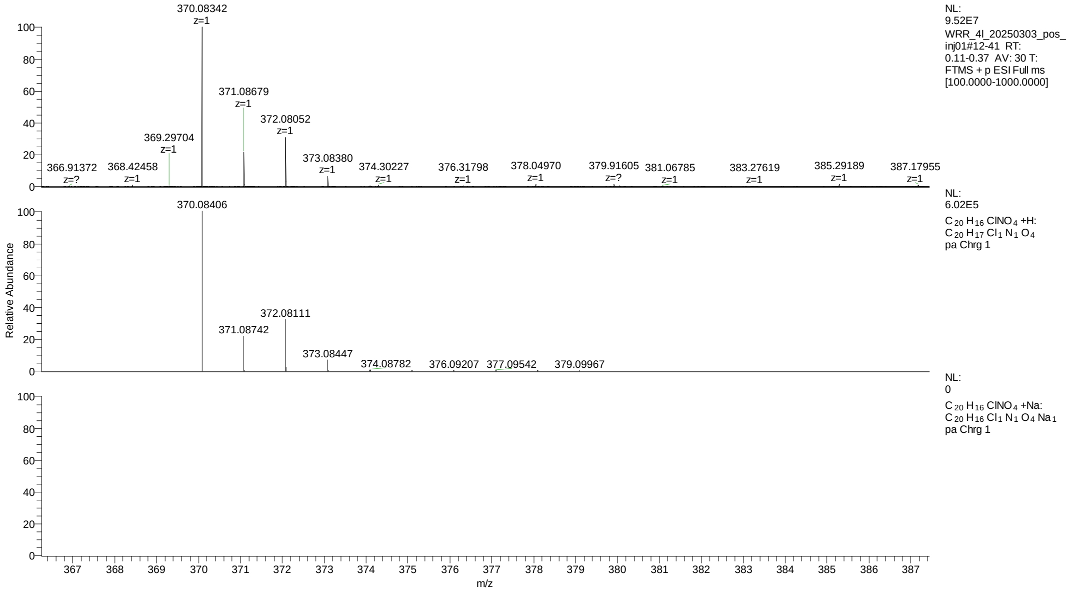

Figure S103. HRMS Spectrum of compound **4l**

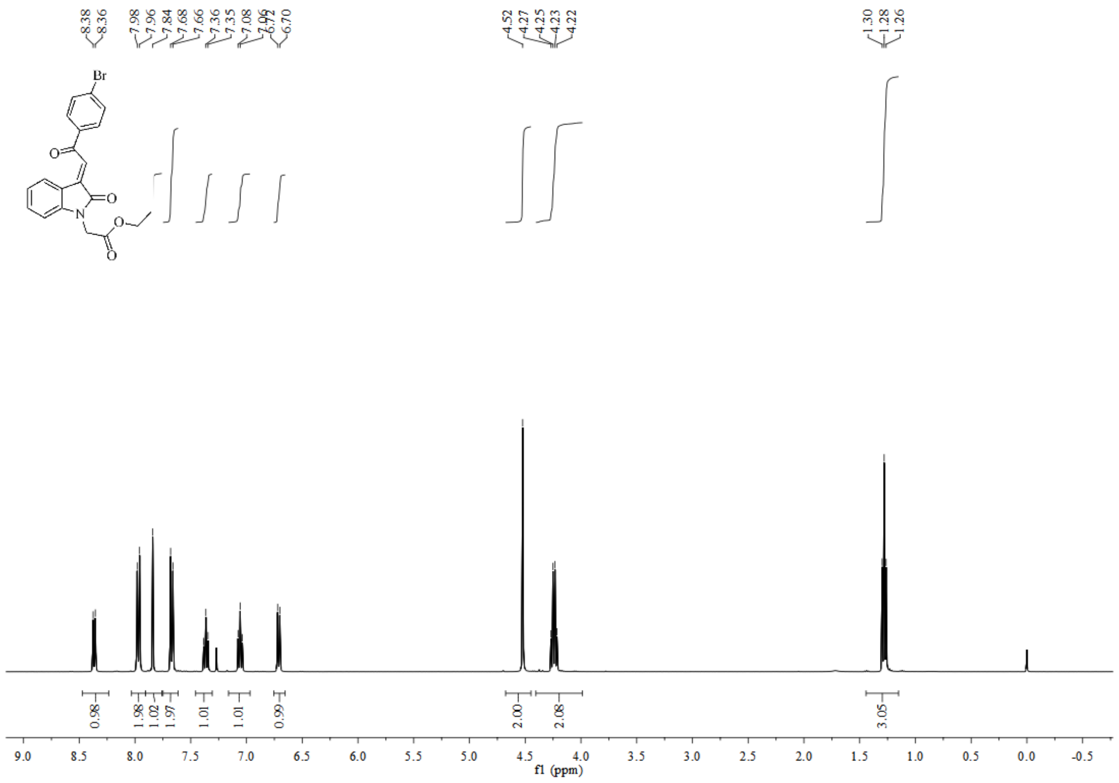

Figure S104. ^{1}H NMR Spectrum of compound **4m**

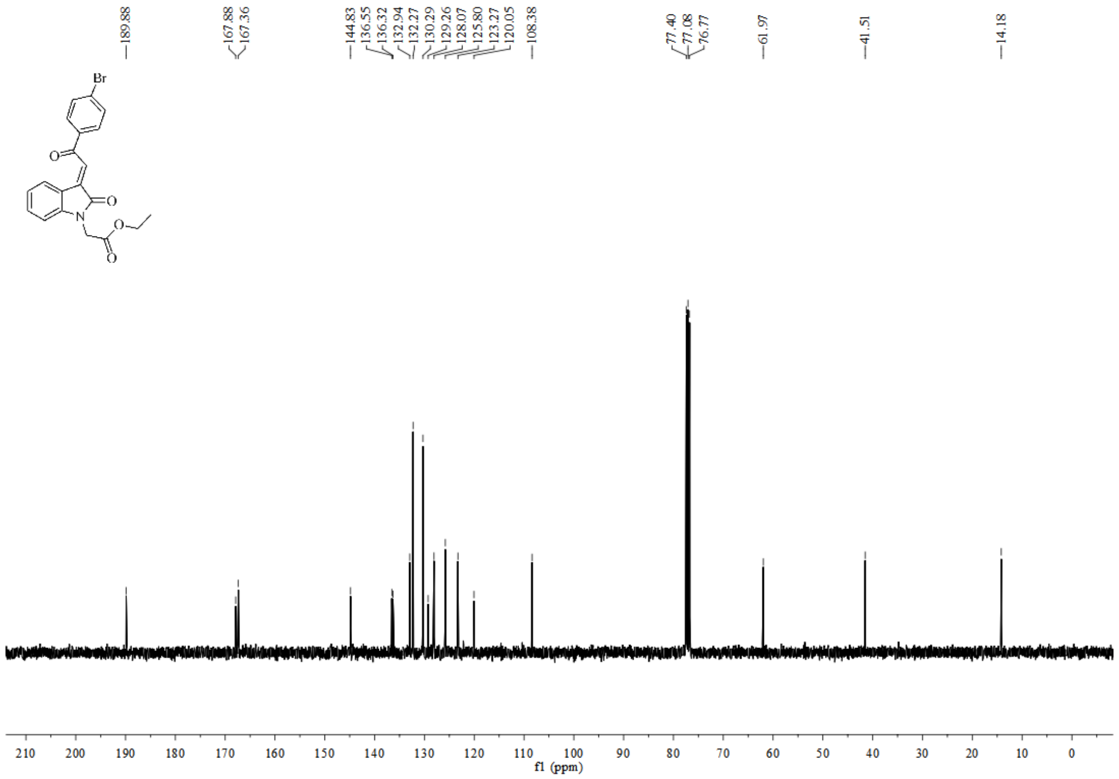

Figure S105. ^{13}C NMR Spectrum of compound **4m**

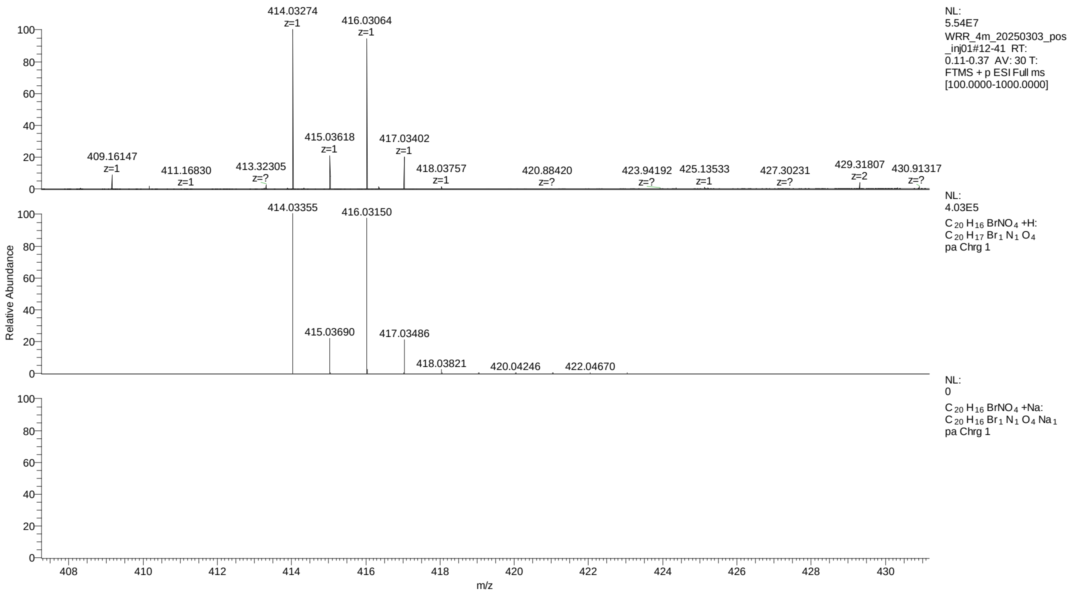

Figure S106. HRMS Spectrum of compound **4m**

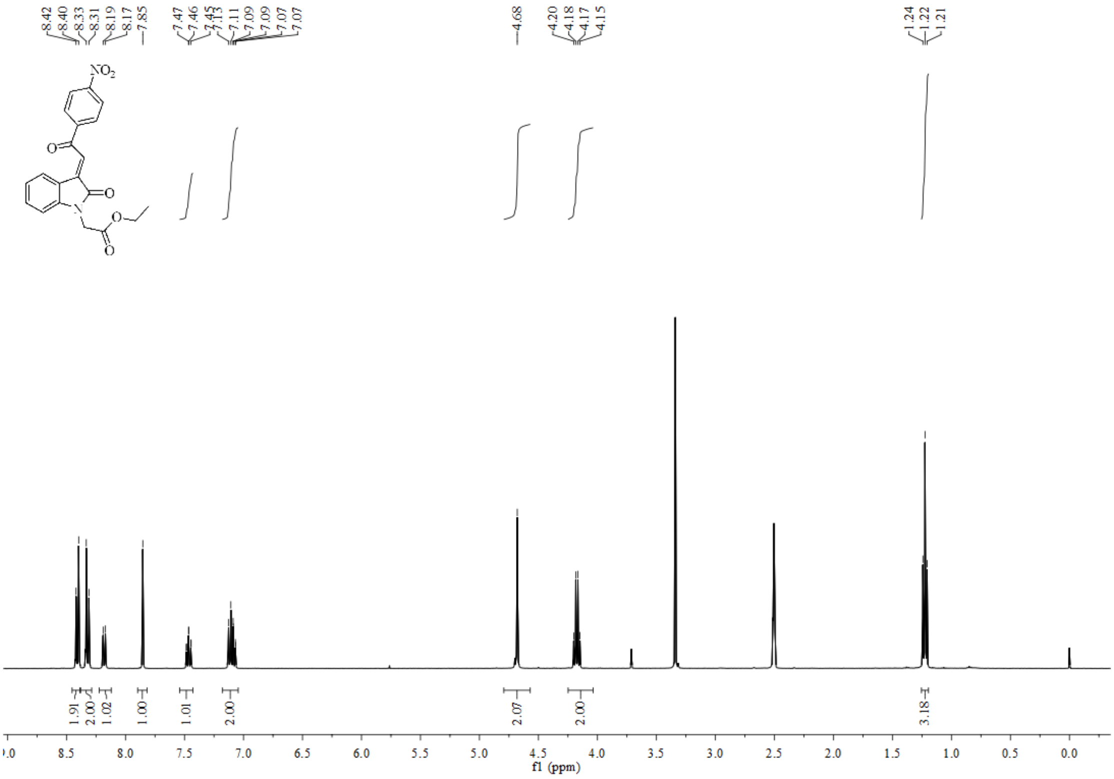

Figure S107. ^{1}H NMR Spectrum of compound **4n**

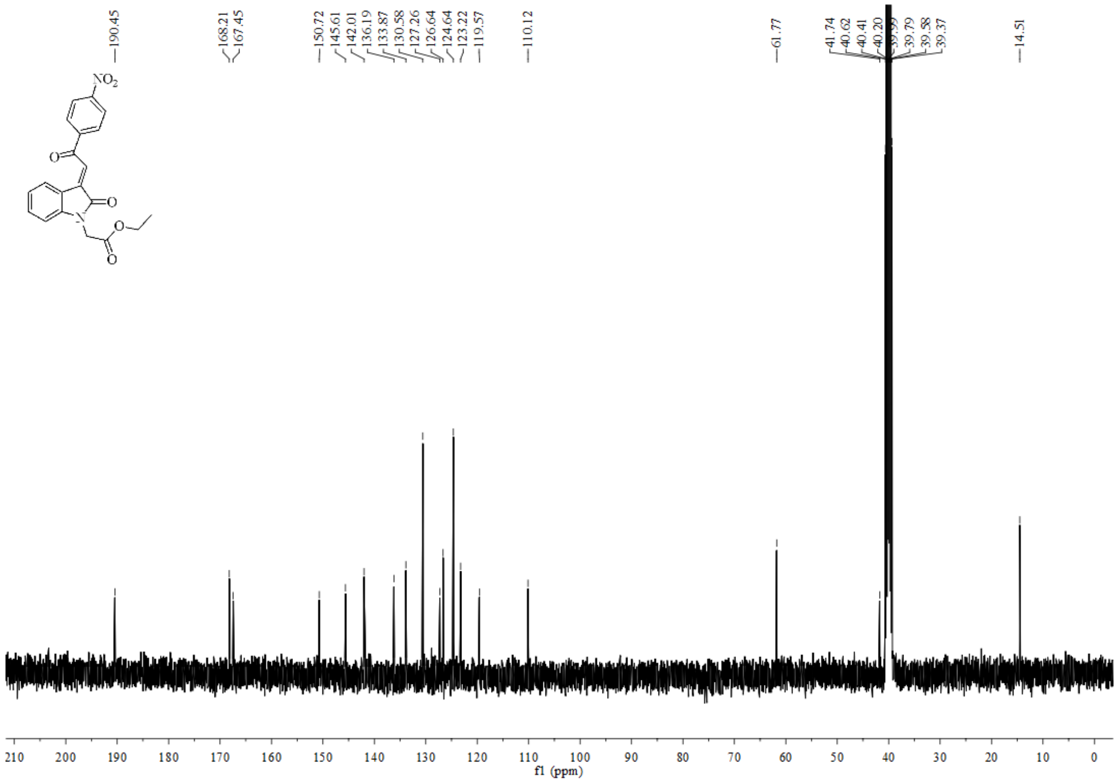

Figure S108. ^{13}C NMR Spectrum of compound **4n**

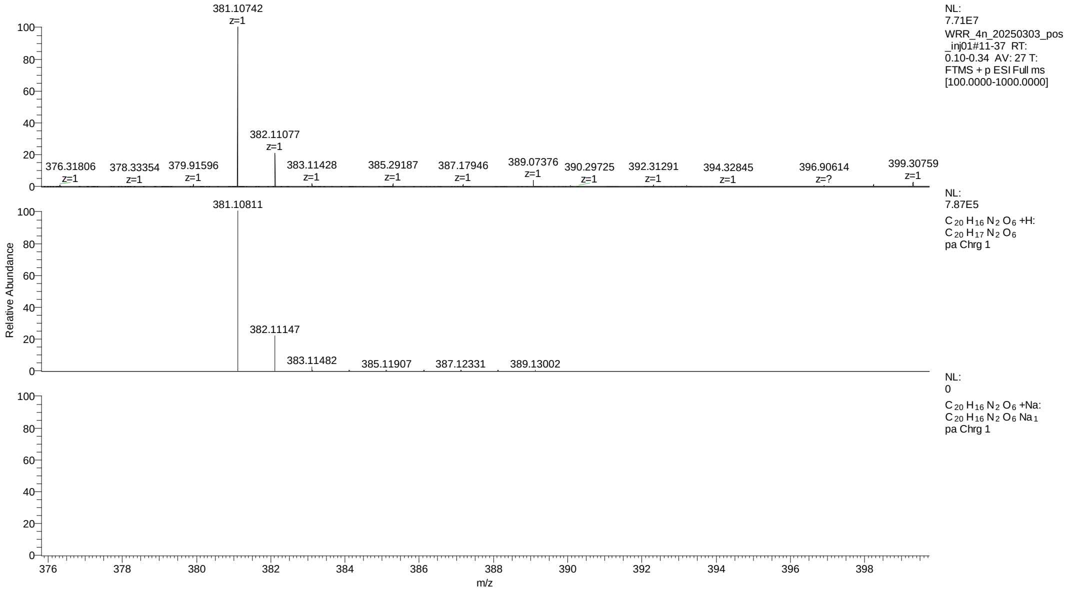

Figure S109. HRMS Spectrum of compound **4n**

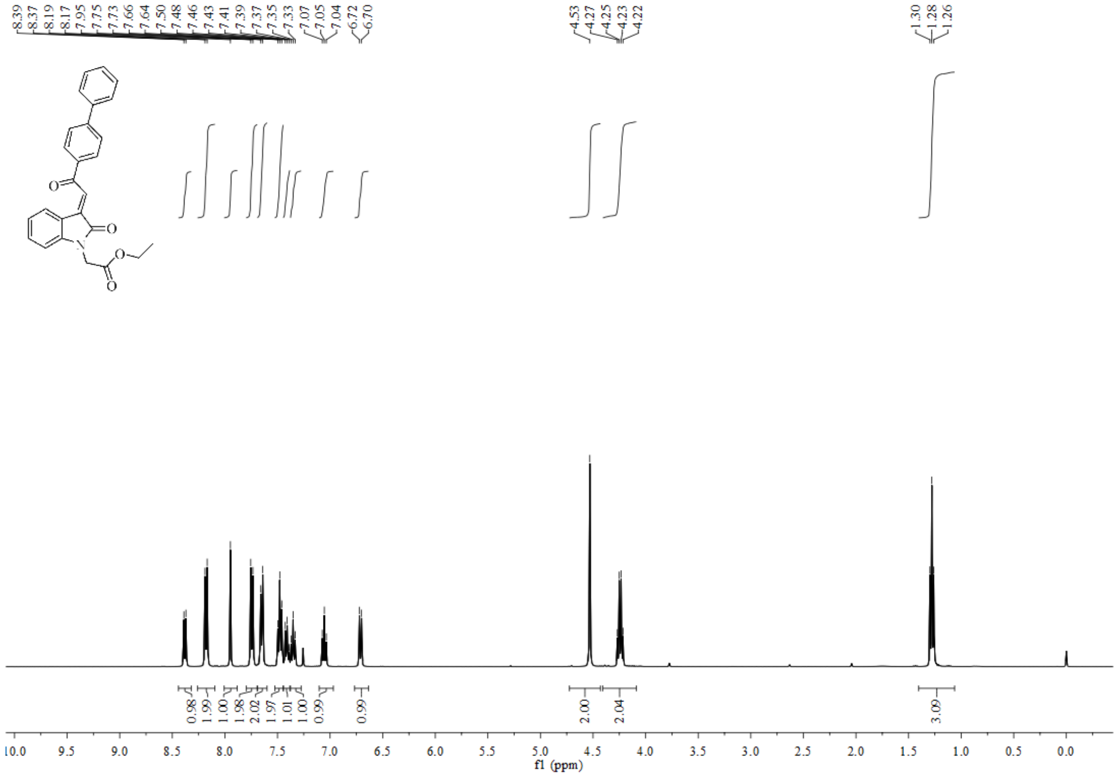

Figure S110. ^{1}H NMR Spectrum of compound **4o**

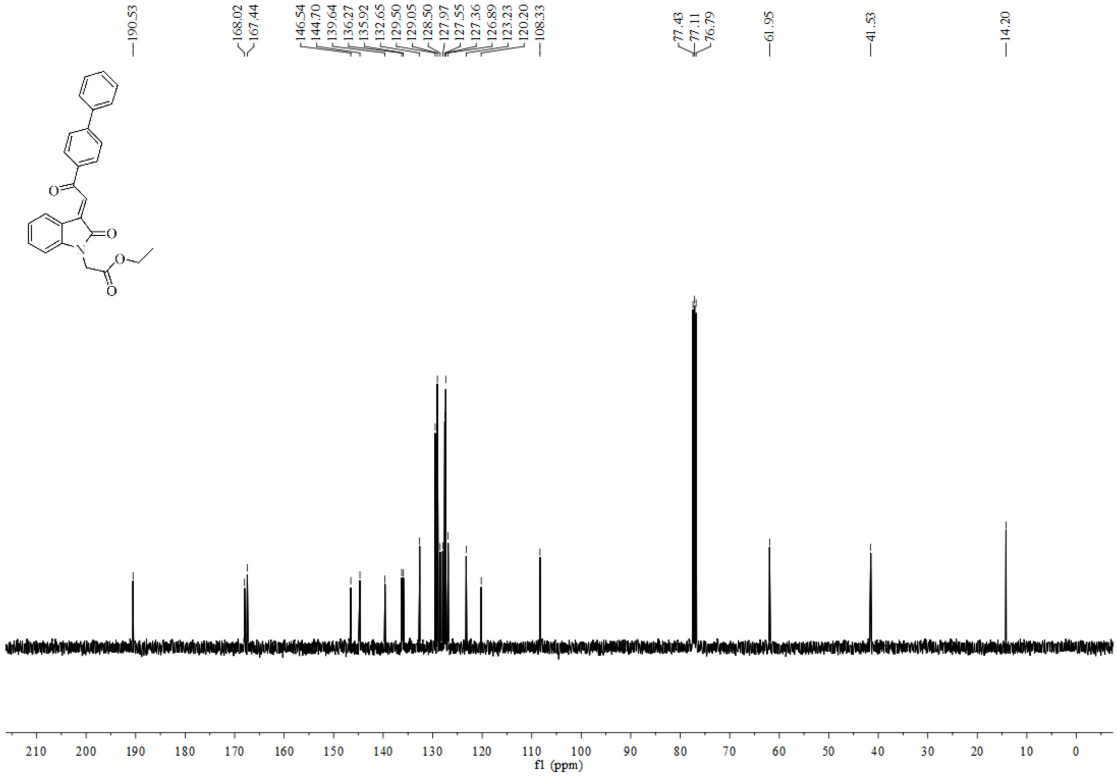

Figure S111. ^{13}C NMR Spectrum of compound **4o**

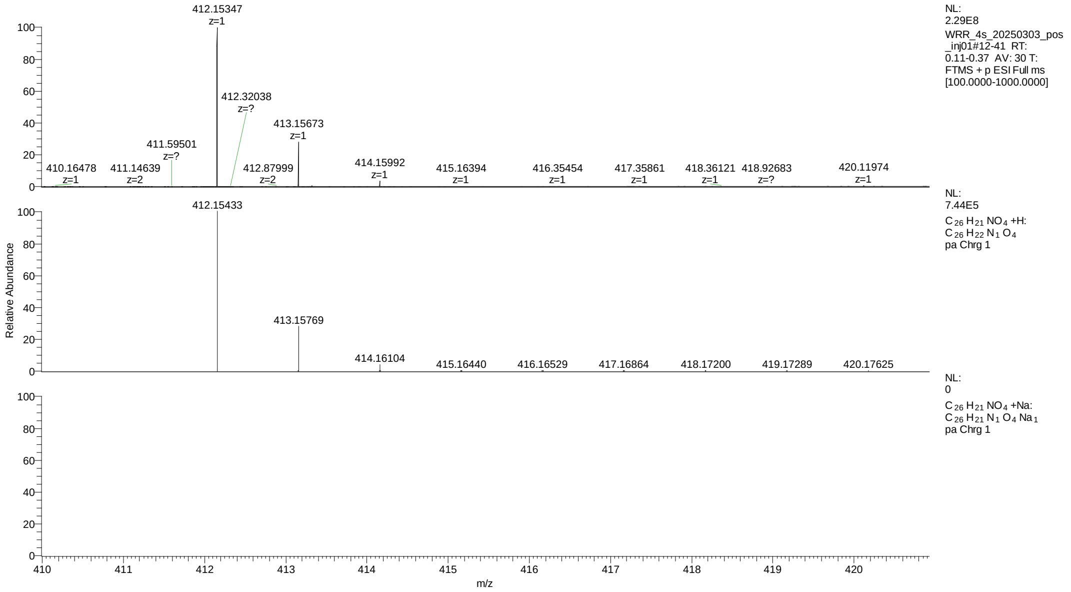

Figure S112. HRMS Spectrum of compound **4o**

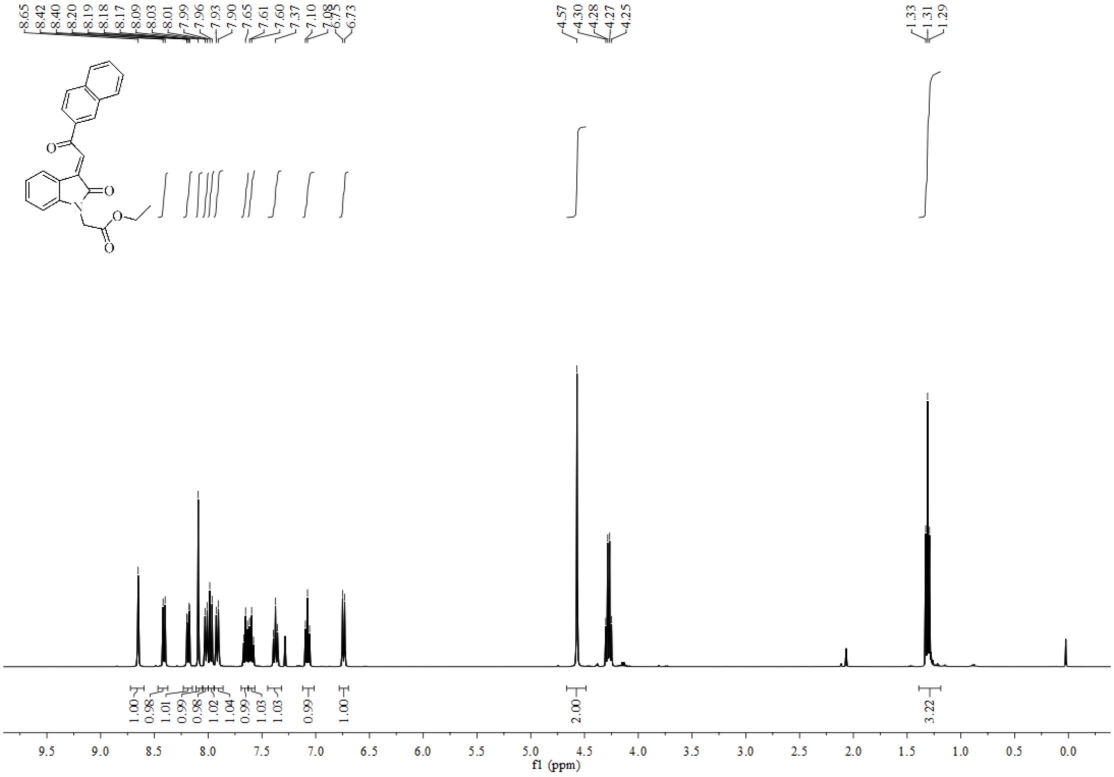

Figure S113. ^{1}H NMR Spectrum of compound **4p**

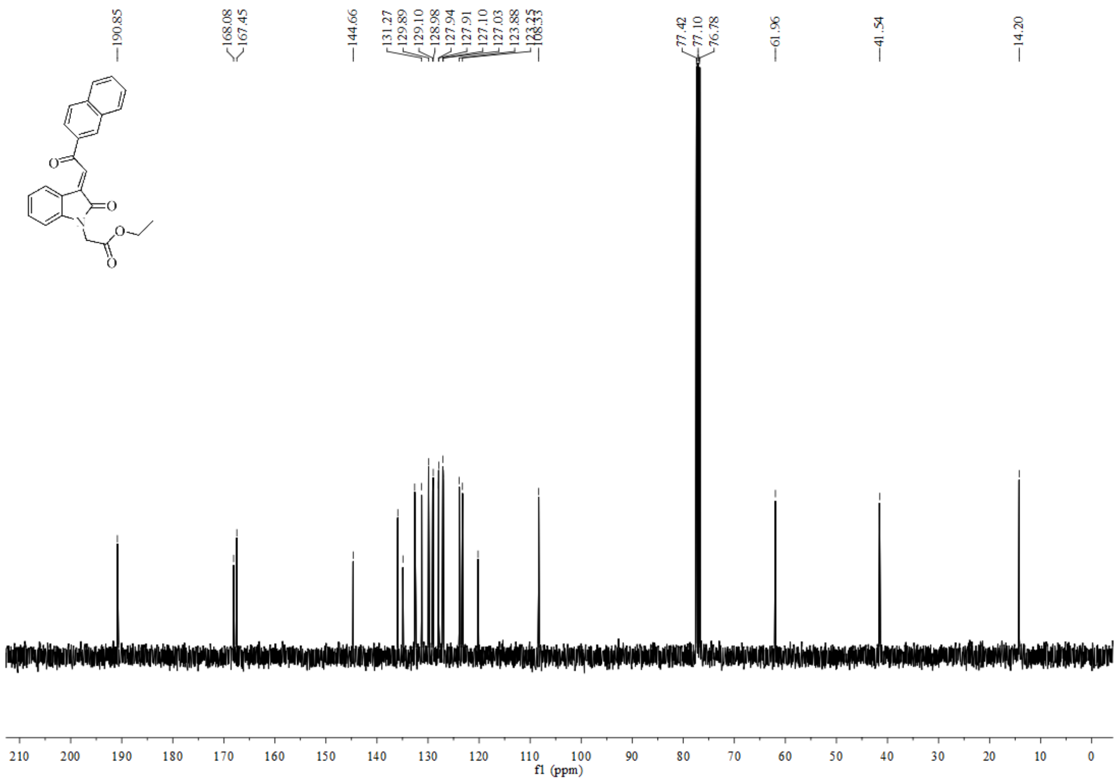

Figure S114. ^{13}C NMR Spectrum of compound **4p**

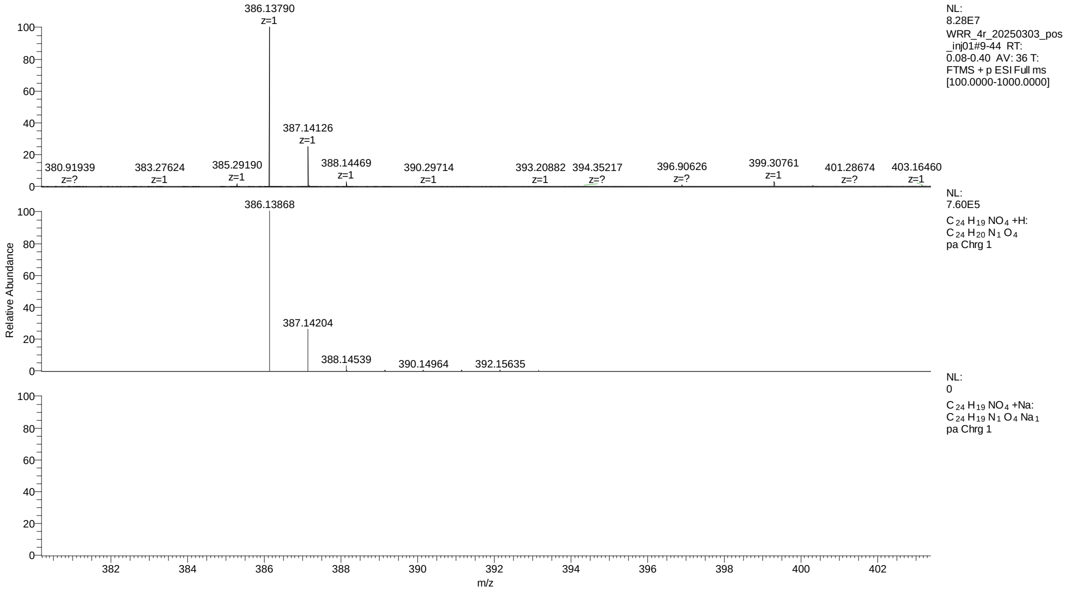

Figure S115. HRMS Spectrum of compound **4p**

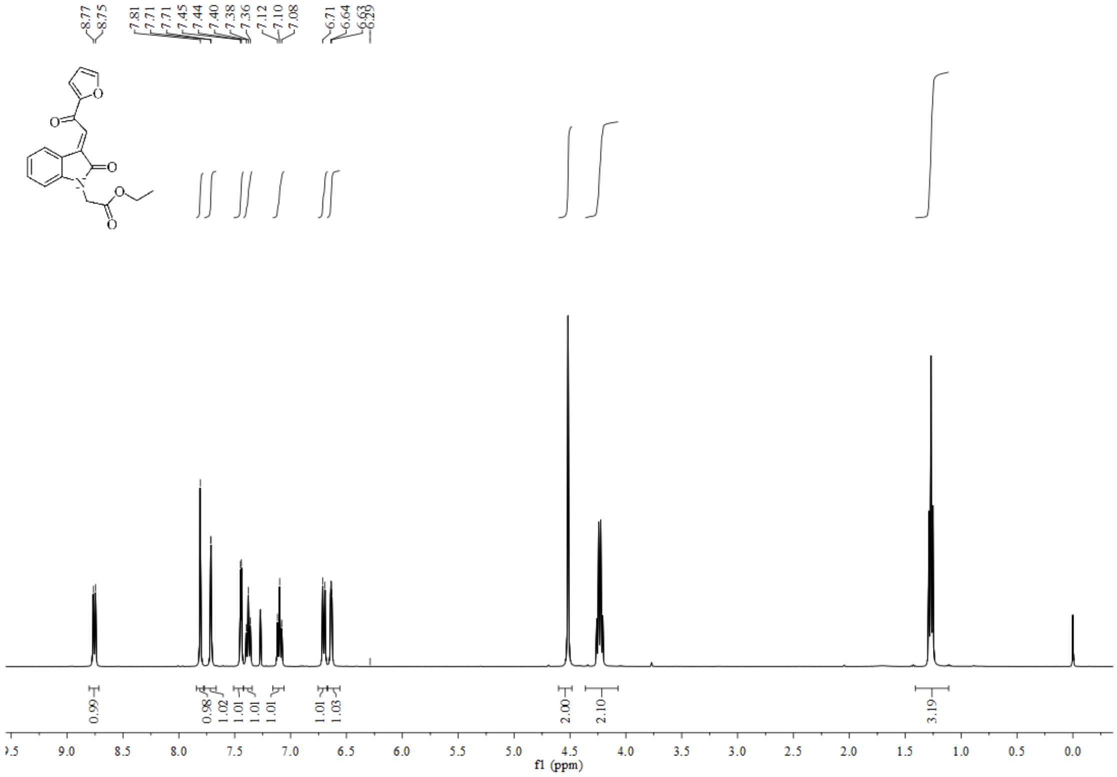

Figure S116. ^{1}H NMR Spectrum of compound **4q**

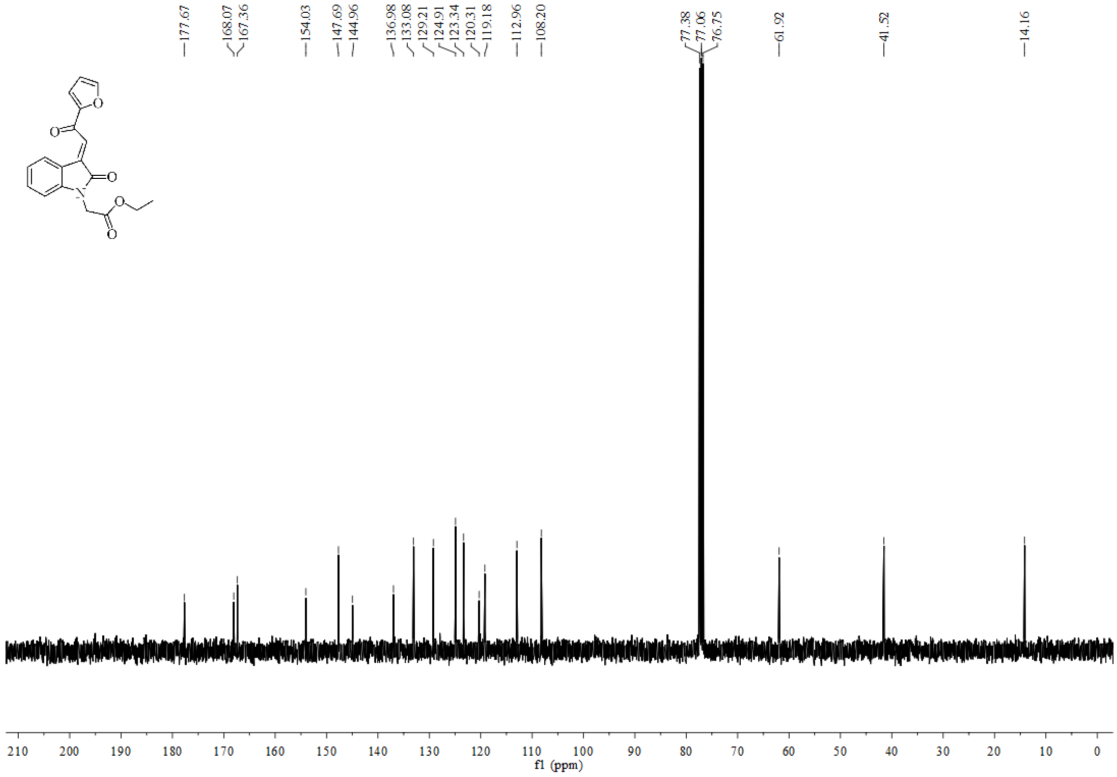

Figure S117. ^{13}C NMR Spectrum of compound **4q**

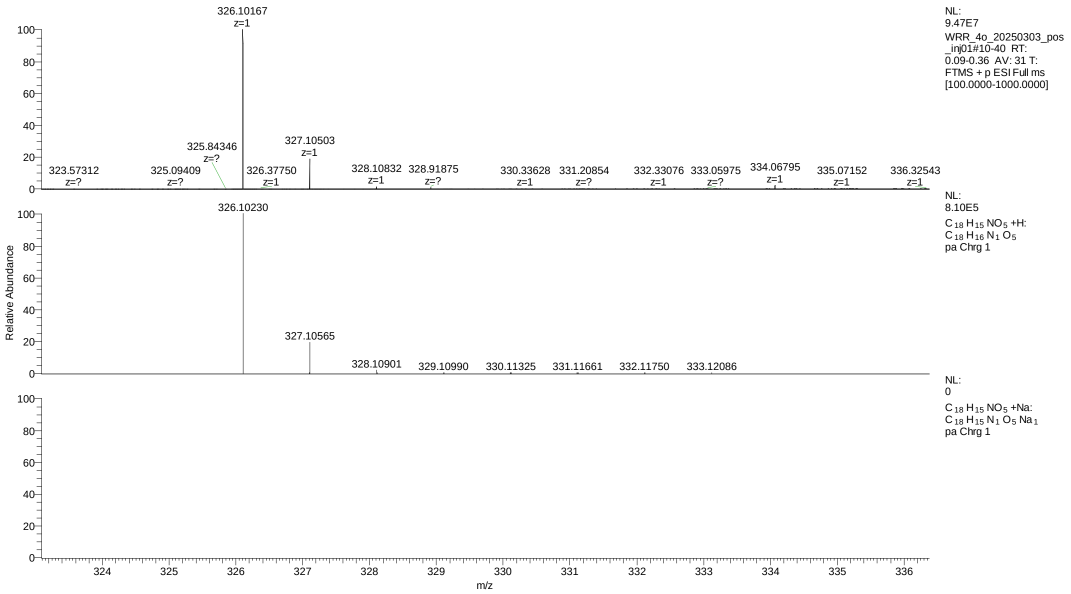

Figure S118. HRMS Spectrum of compound **4q**

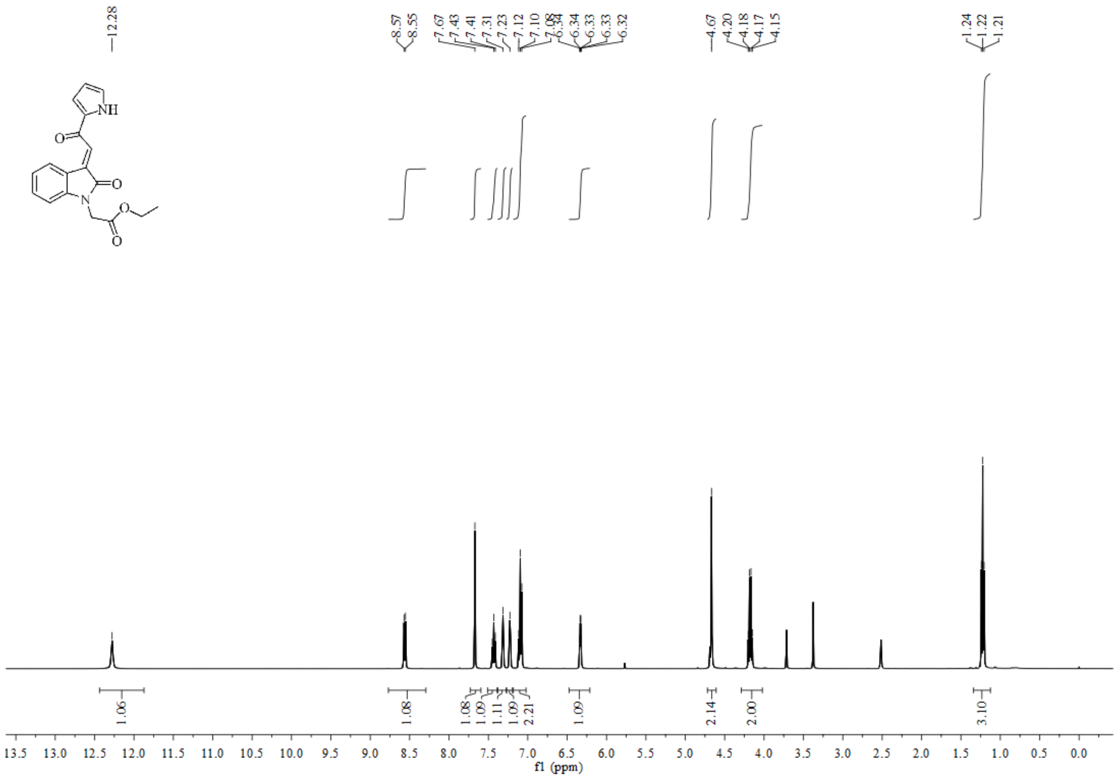

Figure S119. ^{1}H NMR Spectrum of compound **4r**

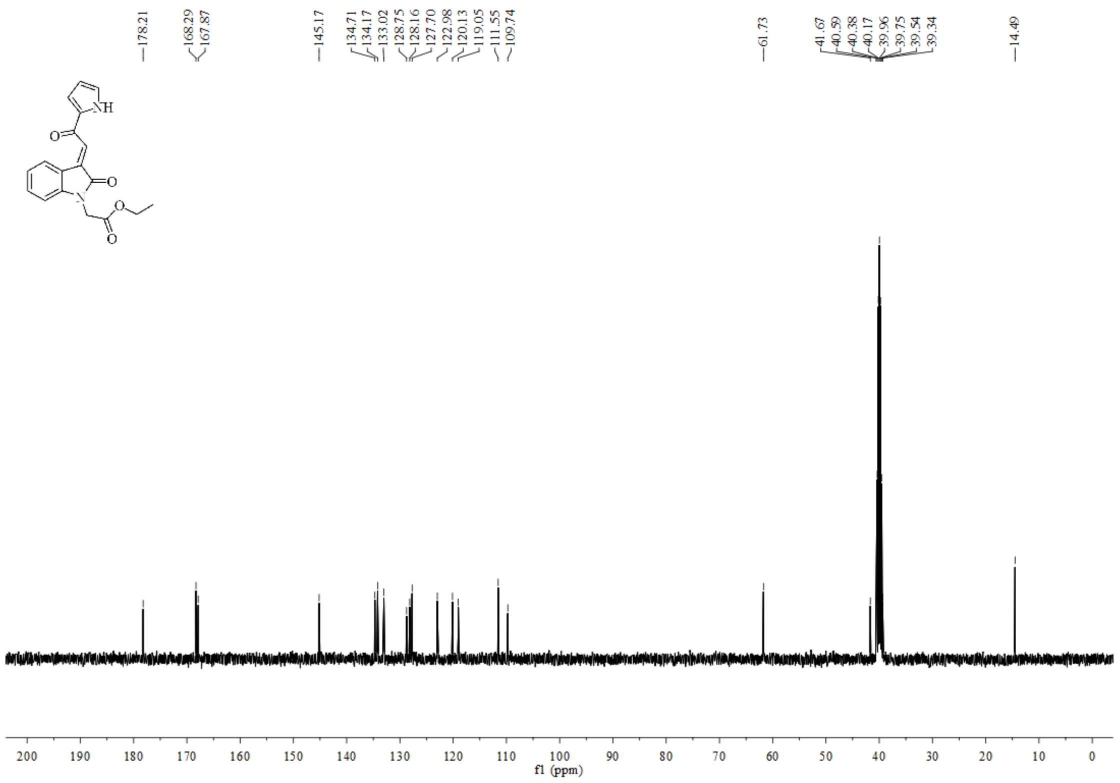

Figure S120. ^{13}C NMR Spectrum of compound **4r**

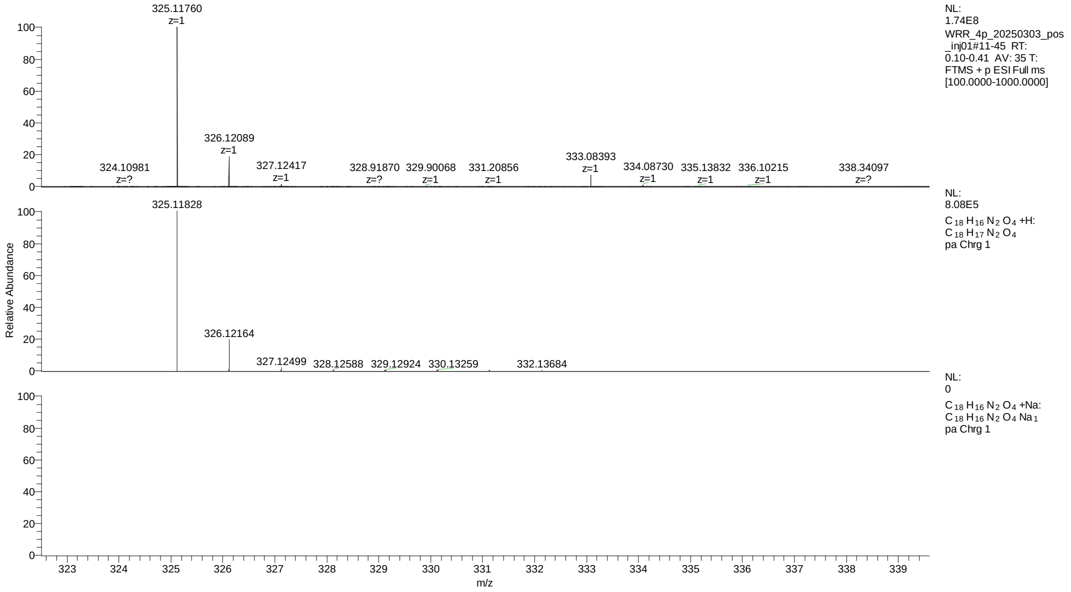

Figure S121. HRMS Spectrum of compound **4r**

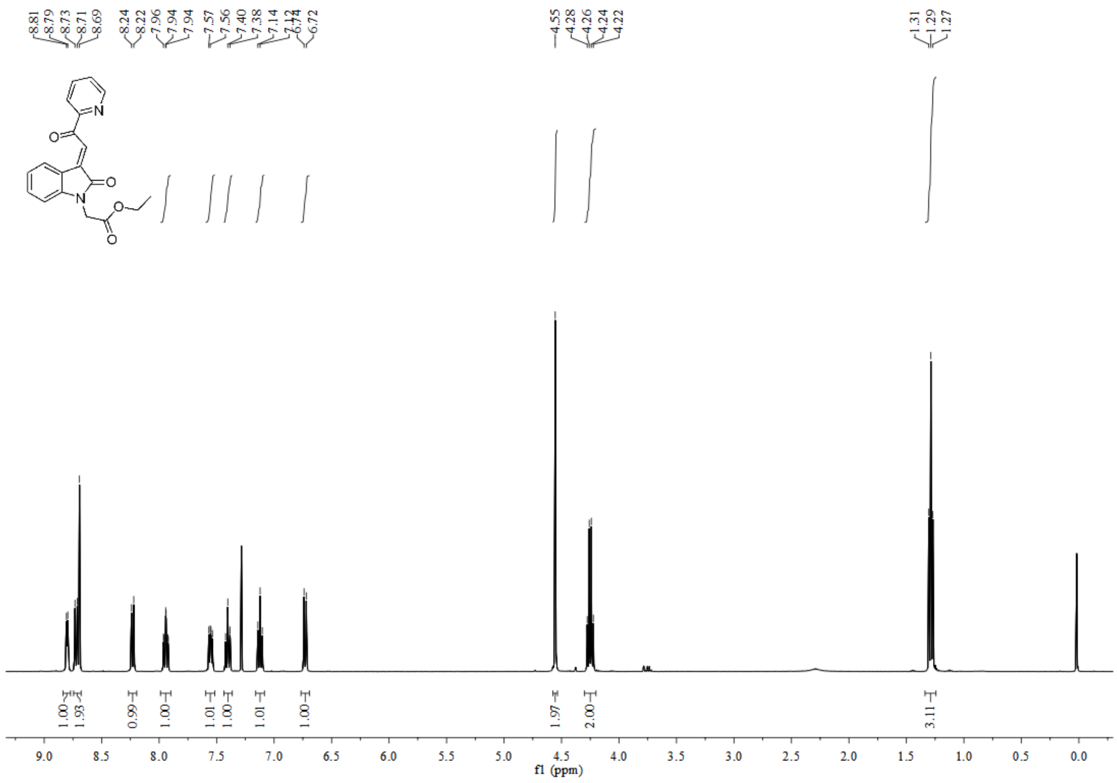

Figure S122. ^{1}H NMR Spectrum of compound **4s**

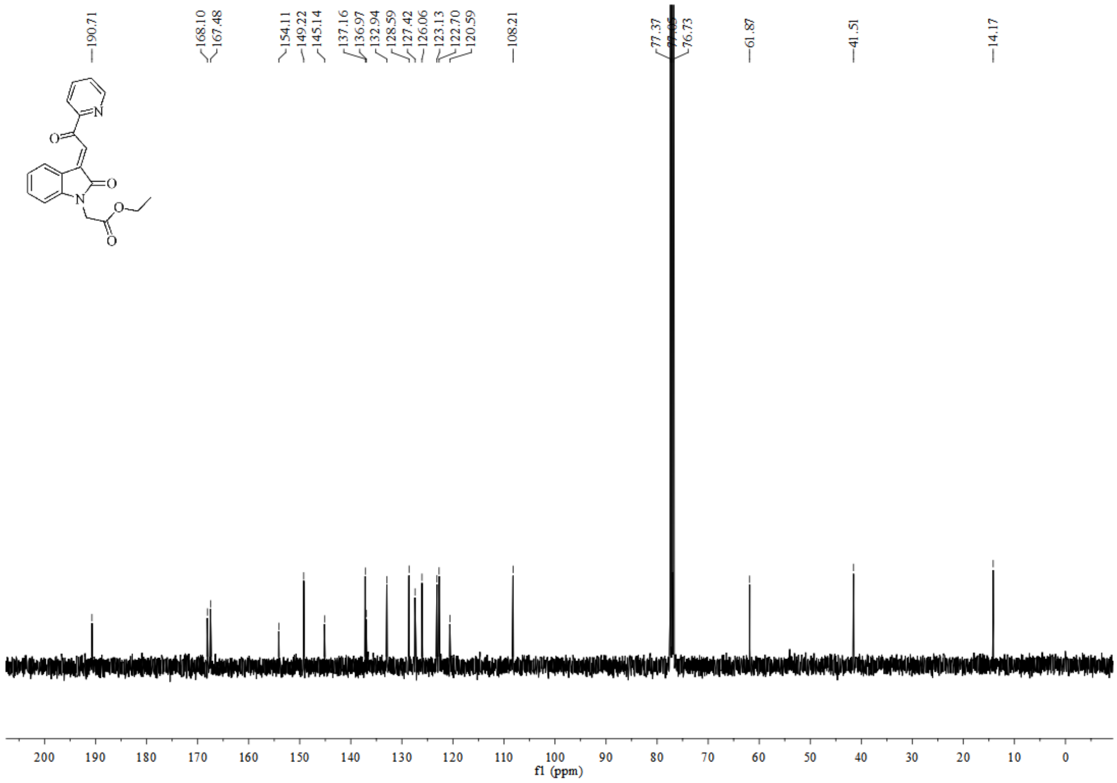

Figure S123. ^{13}C NMR Spectrum of compound **4s**

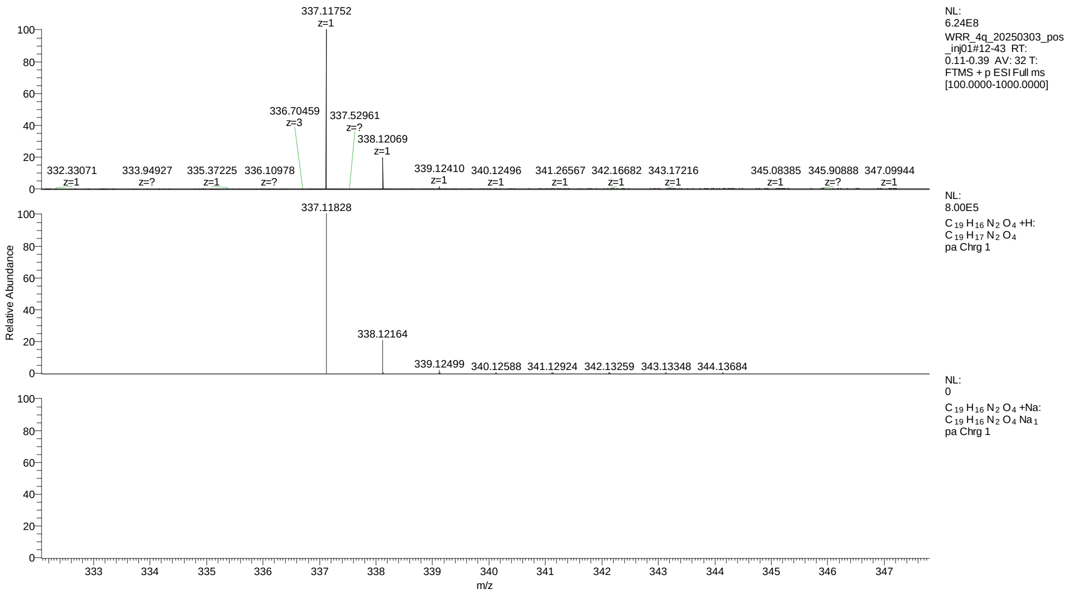

Figure S124. HRMS Spectrum of compound **4s**

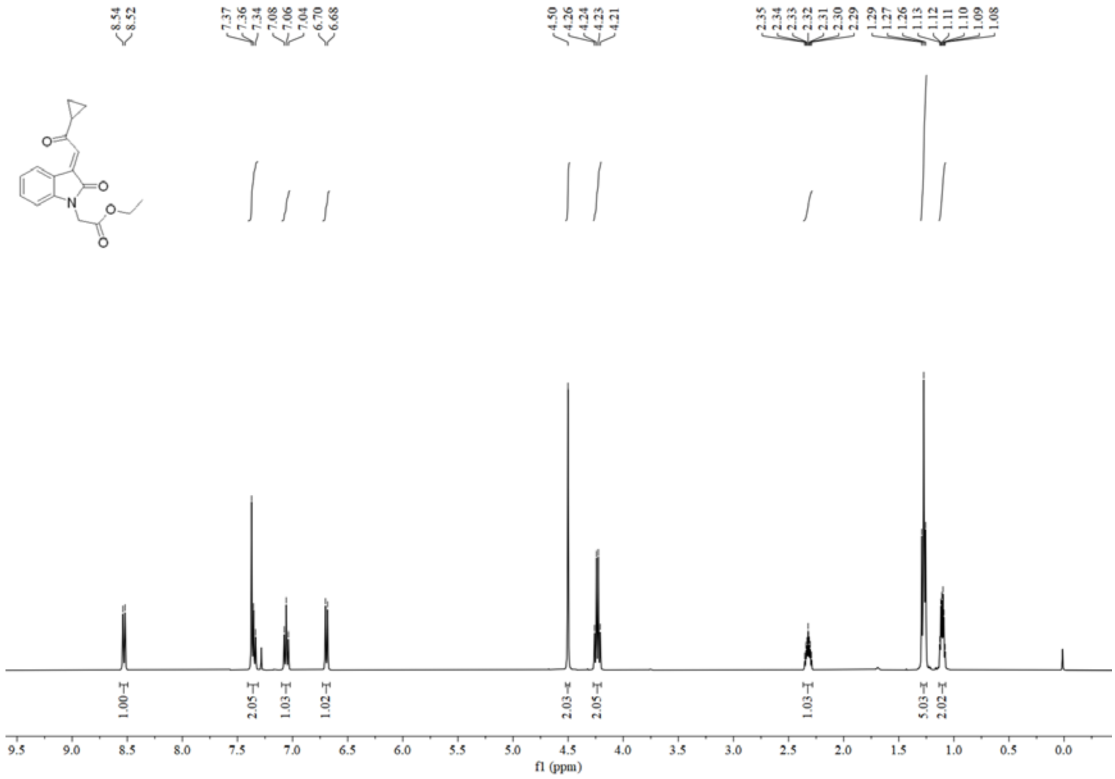

Figure S125. ^{1}H NMR Spectrum of compound **4t**

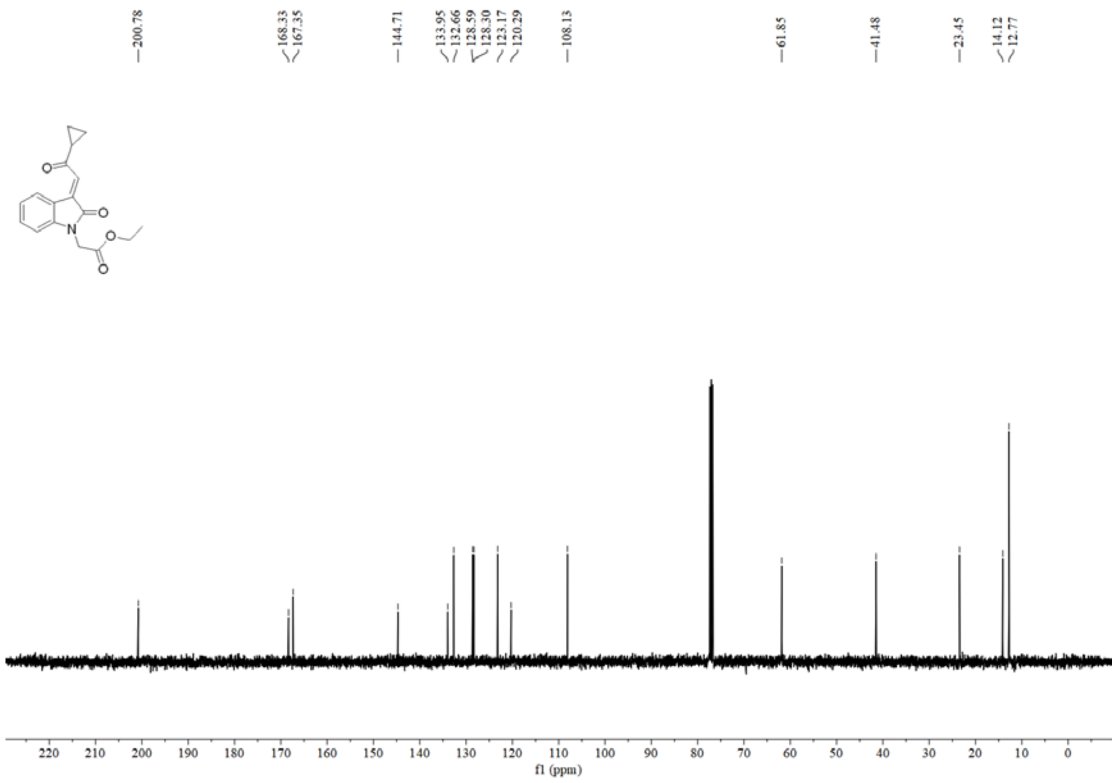

Figure S126. ^{13}C NMR Spectrum of compound **4t**

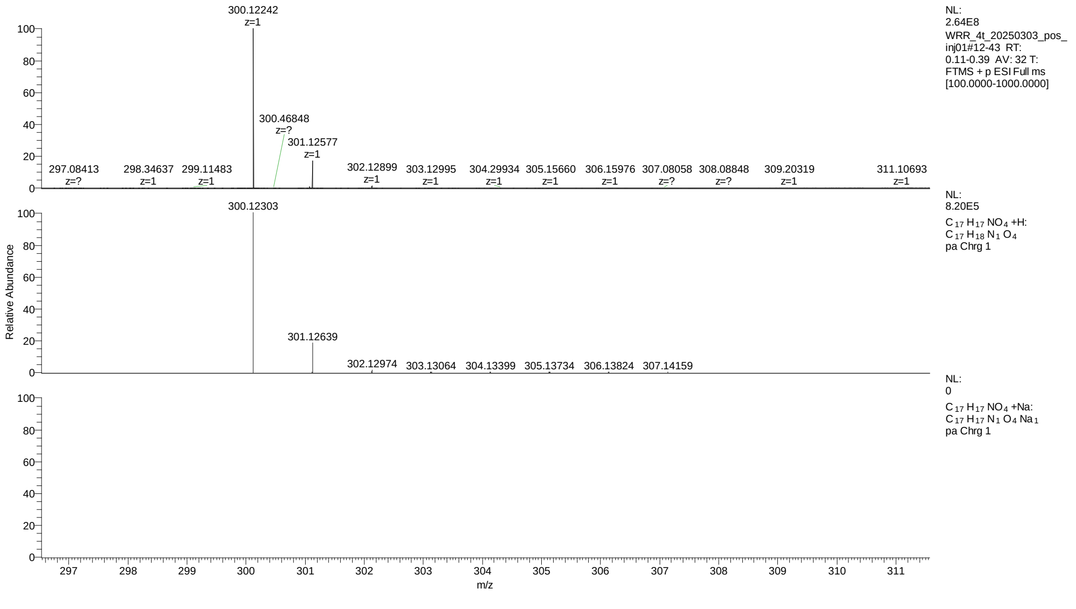

Figure S127. HRMS Spectrum of compound **4t**

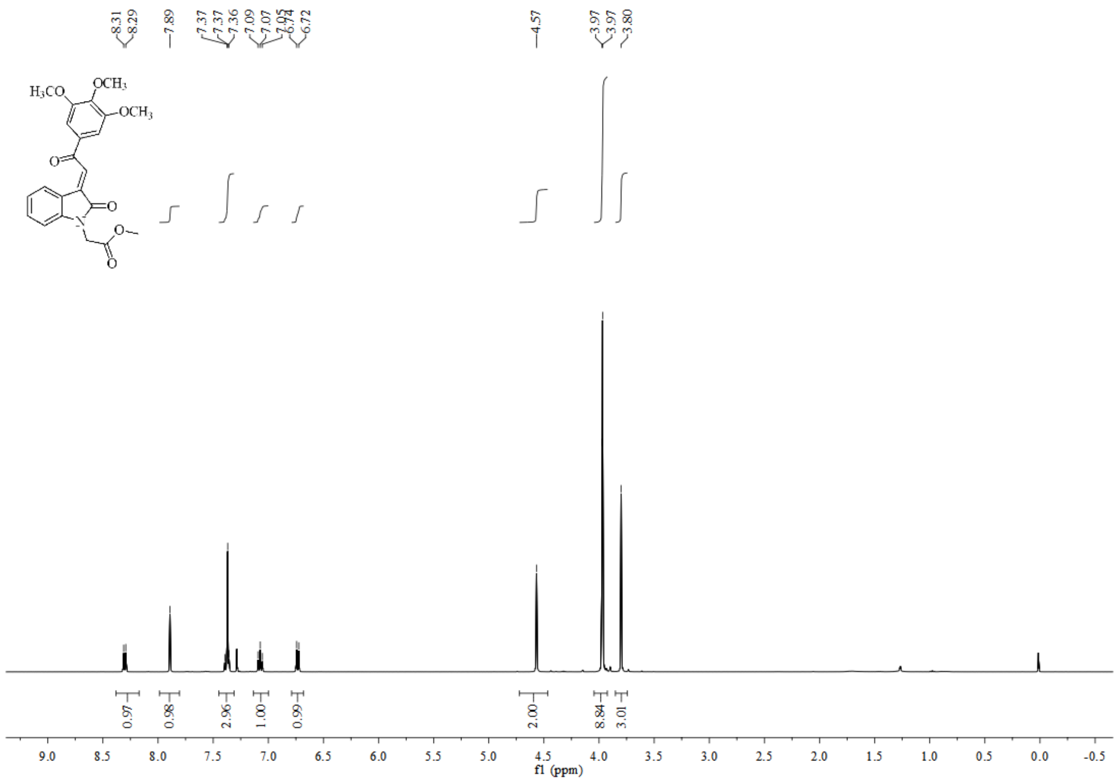

Figure S128. ^{1}H NMR Spectrum of compound **5a**

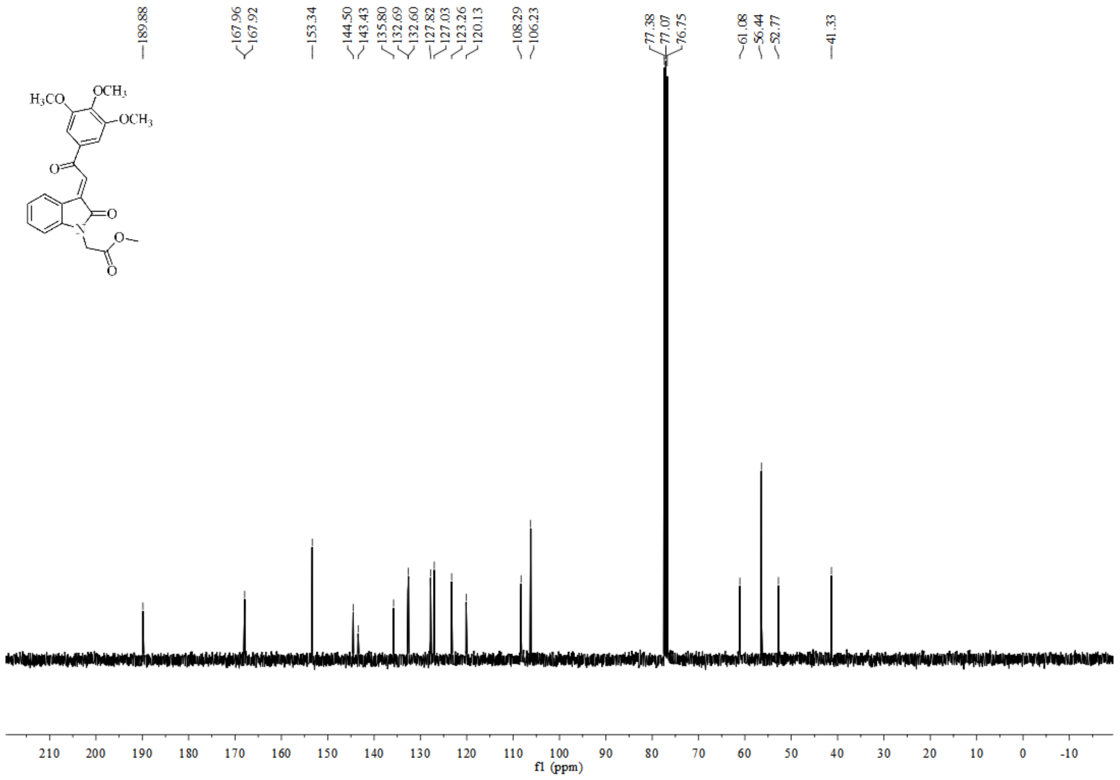

Figure S129. ^{13}C NMR Spectrum of compound **5a**

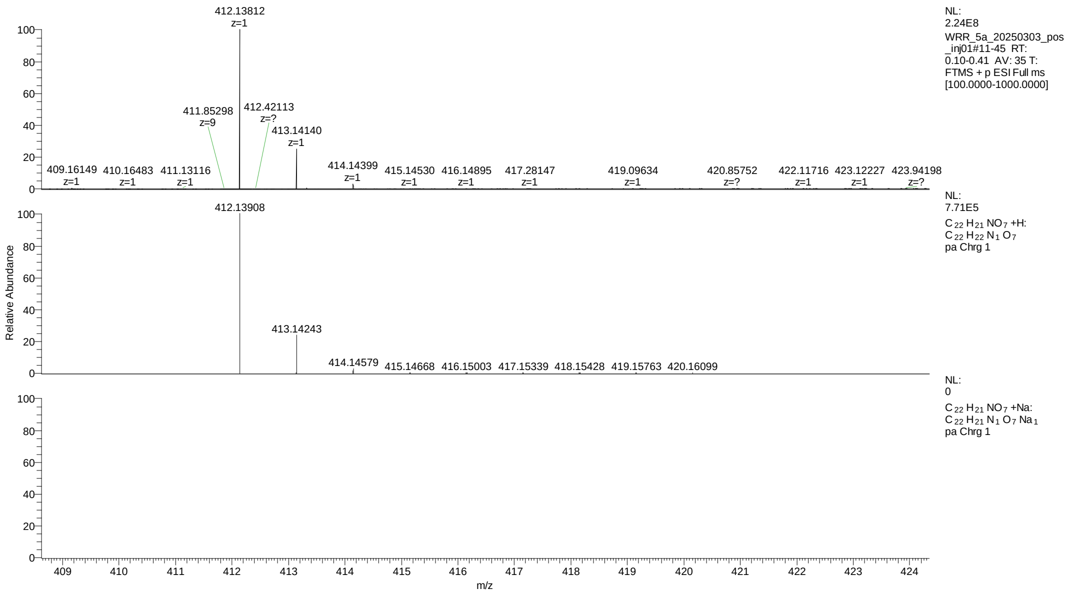

Figure S130. HRMS Spectrum of compound **5a**

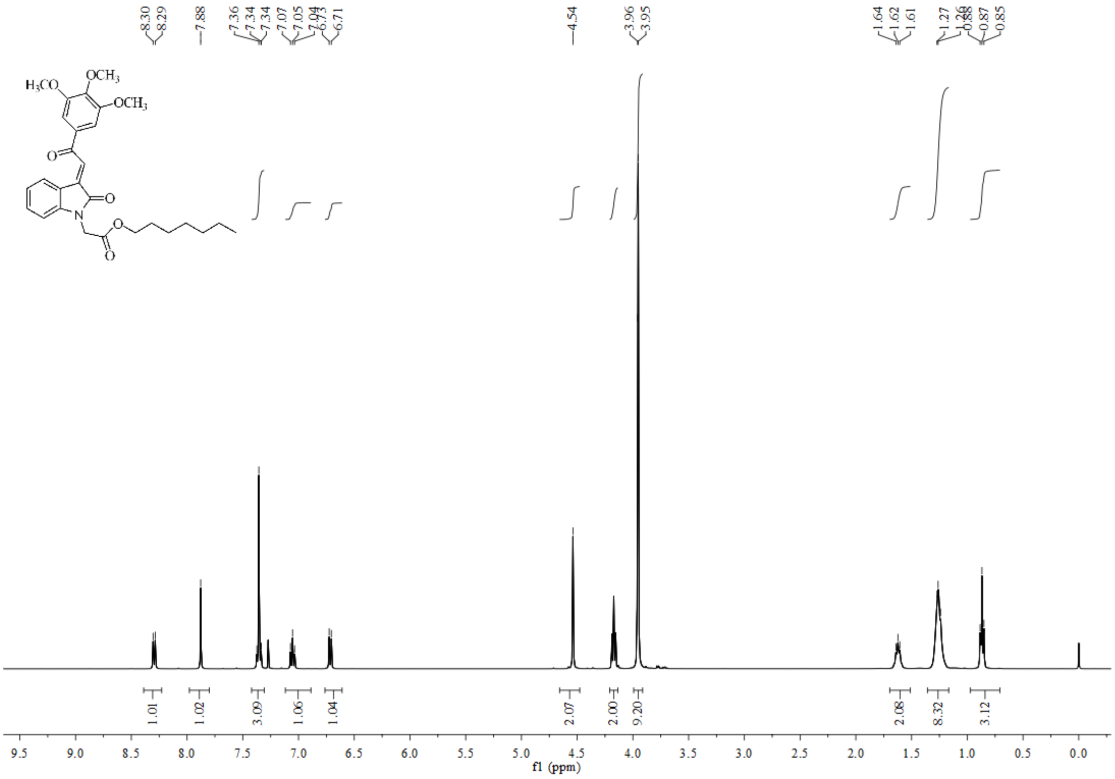

Figure S131. ^{1}H NMR Spectrum of compound **5b**

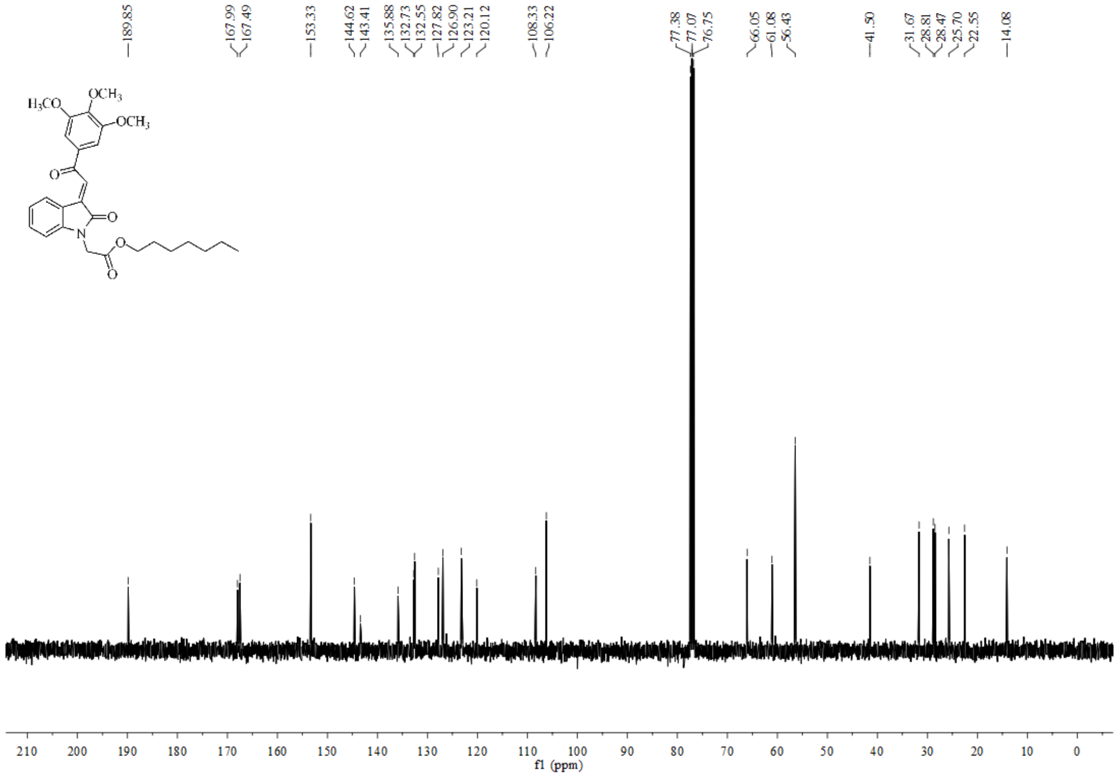

Figure S132. ^{13}C NMR Spectrum of compound **5b**

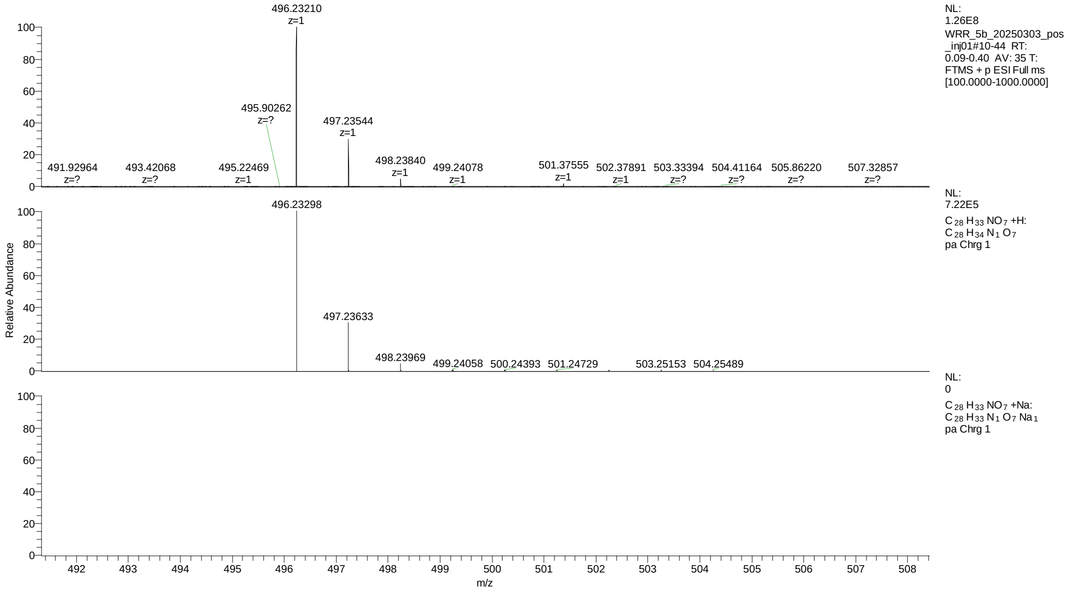

Figure S133. HRMS Spectrum of compound **5b**

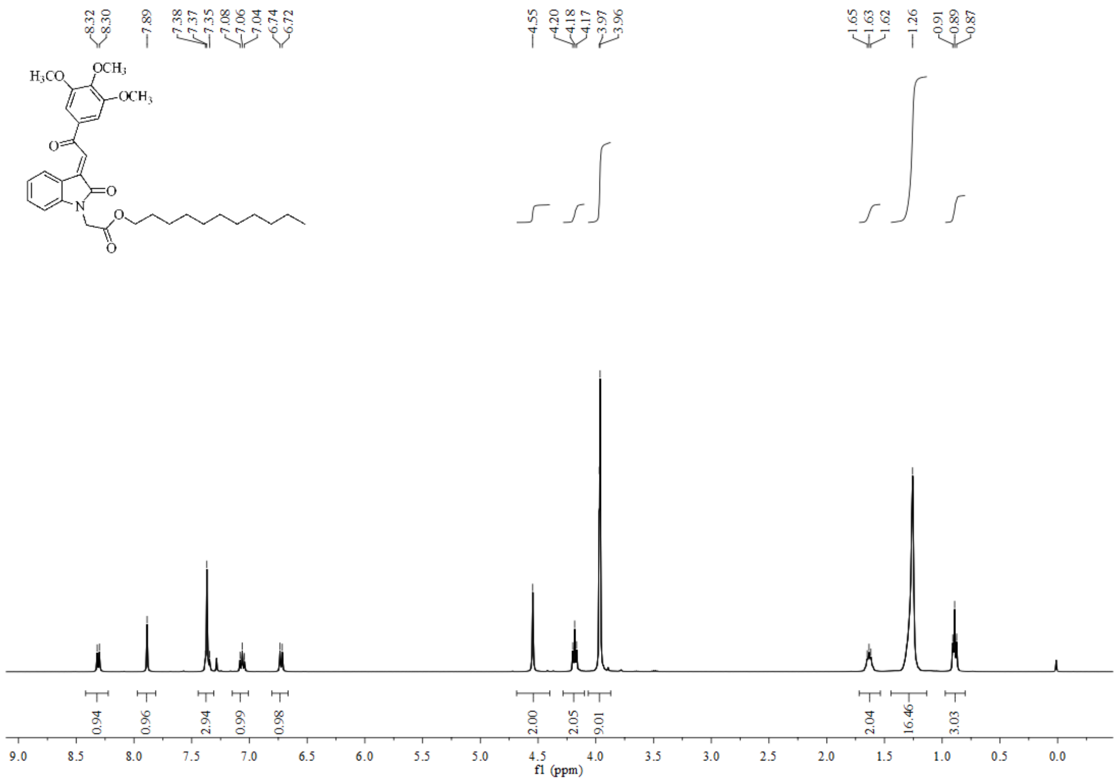

Figure S134. ^{1}H NMR Spectrum of compound **5c**

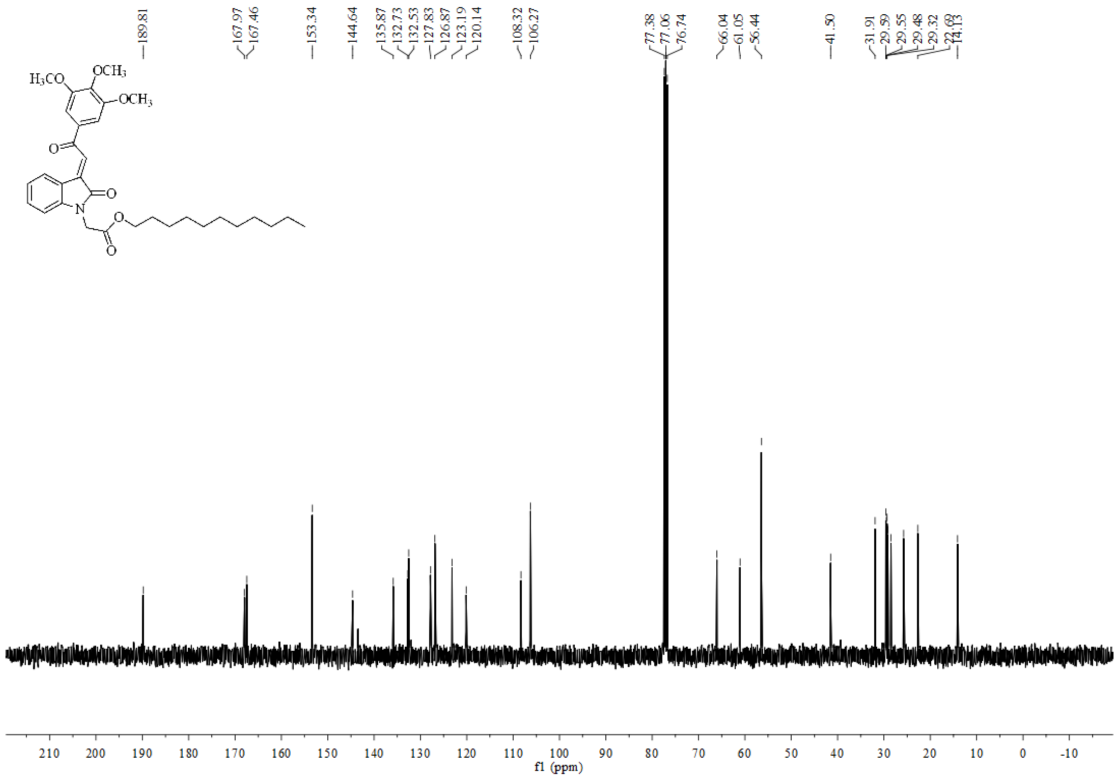

Figure S135. ^{13}C NMR Spectrum of compound **5c**

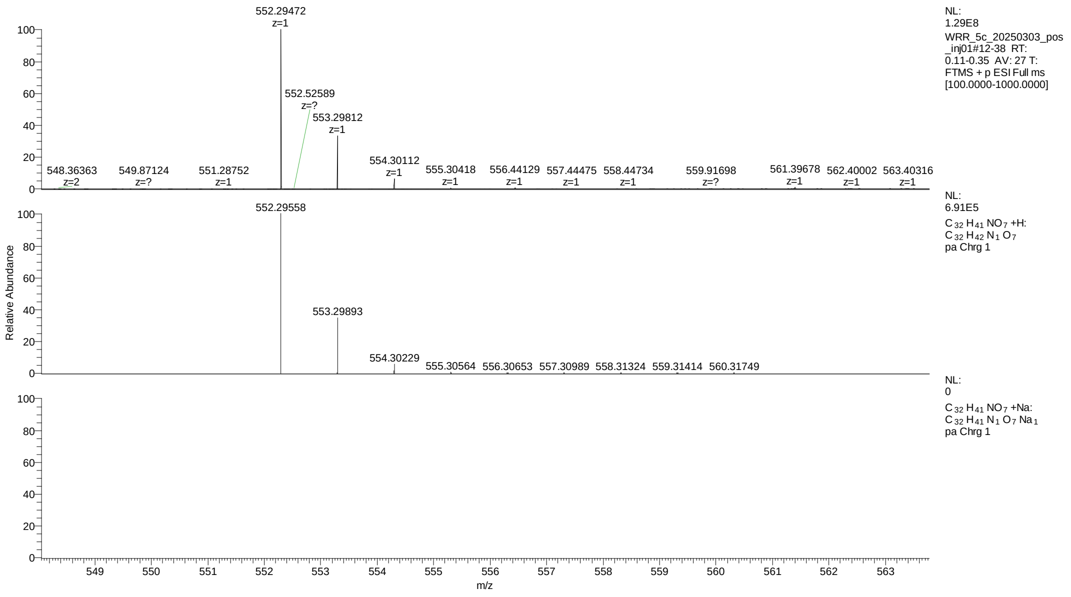

Figure S136. HRMS Spectrum of compound **5c**

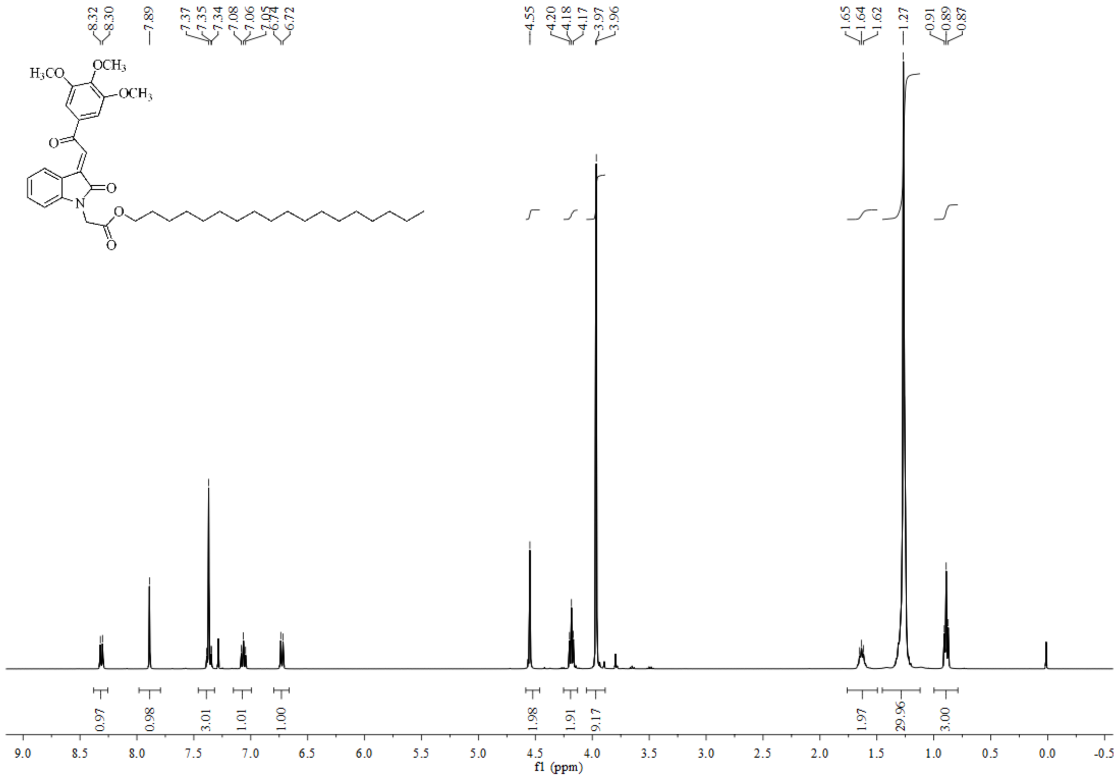

Figure S137. ^{1}H NMR Spectrum of compound **5d**

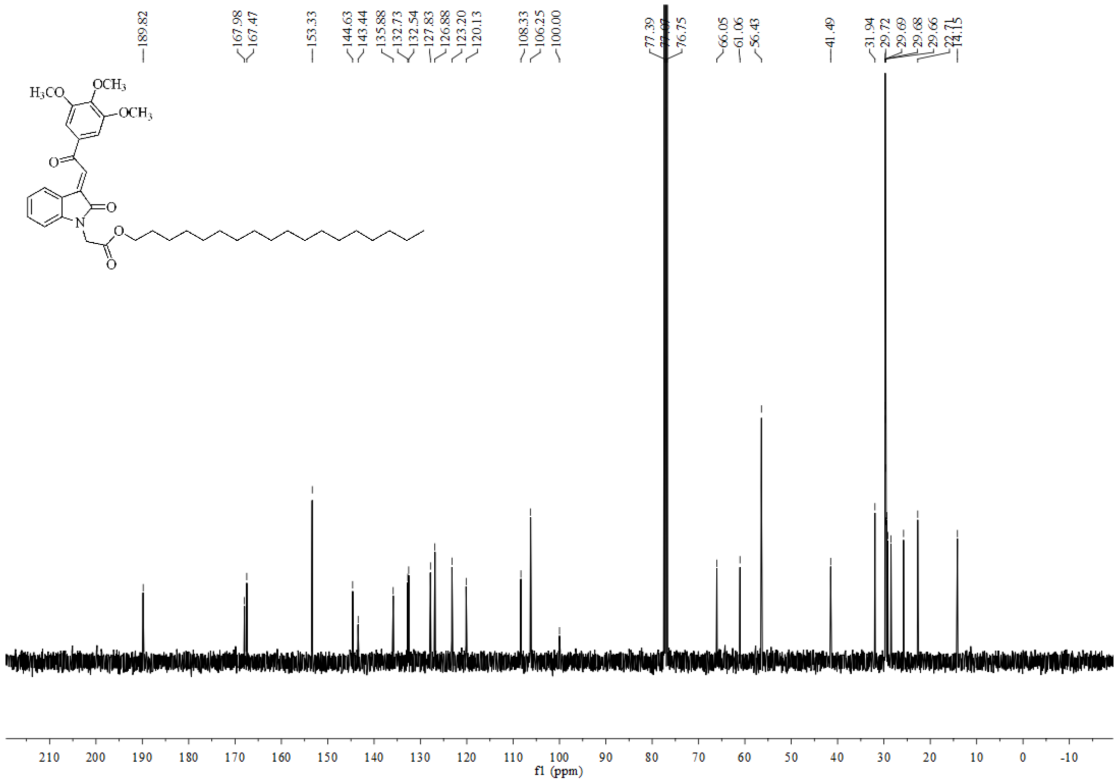

Figure S138. ^{13}C NMR Spectrum of compound **5d**

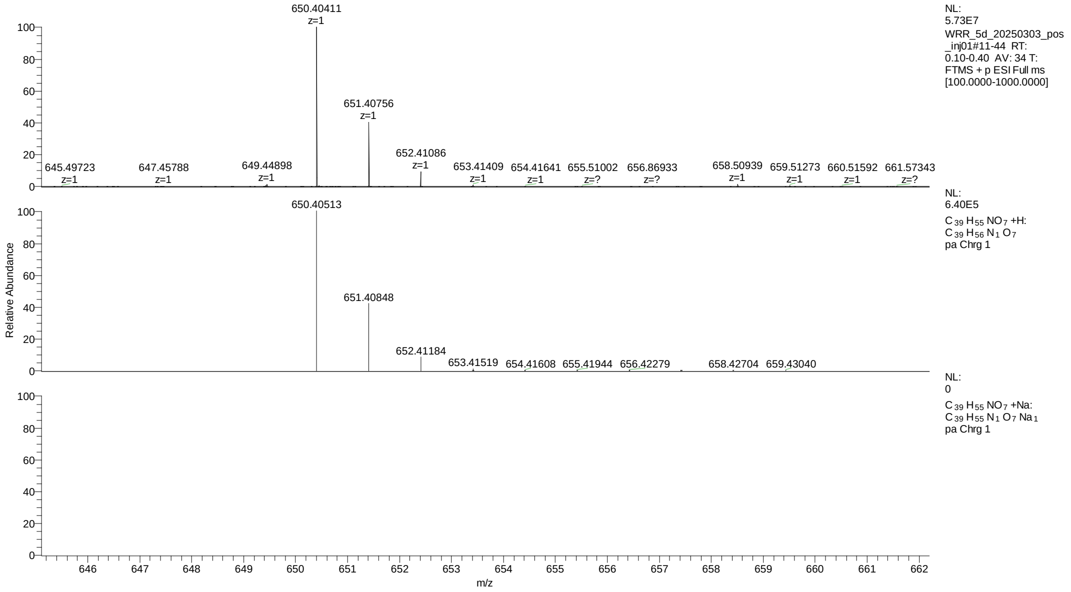

Figure S139. HRMS Spectrum of compound **5d**

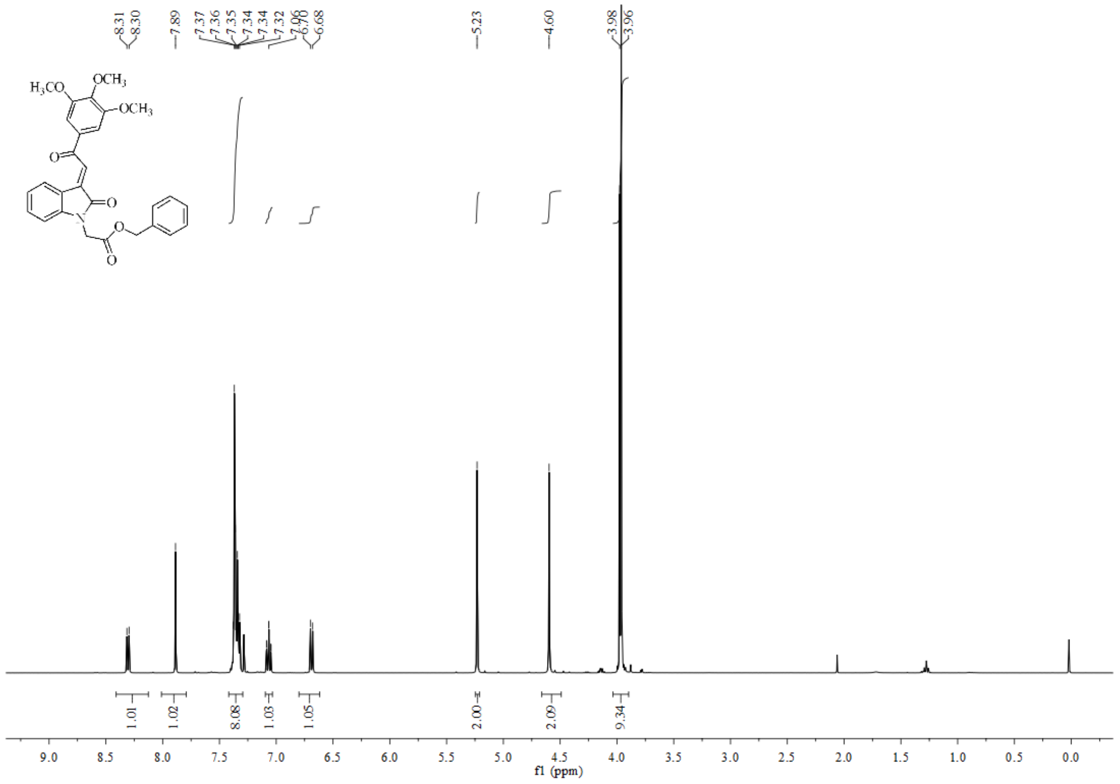

Figure S140. ^{1}H NMR Spectrum of compound **5e**

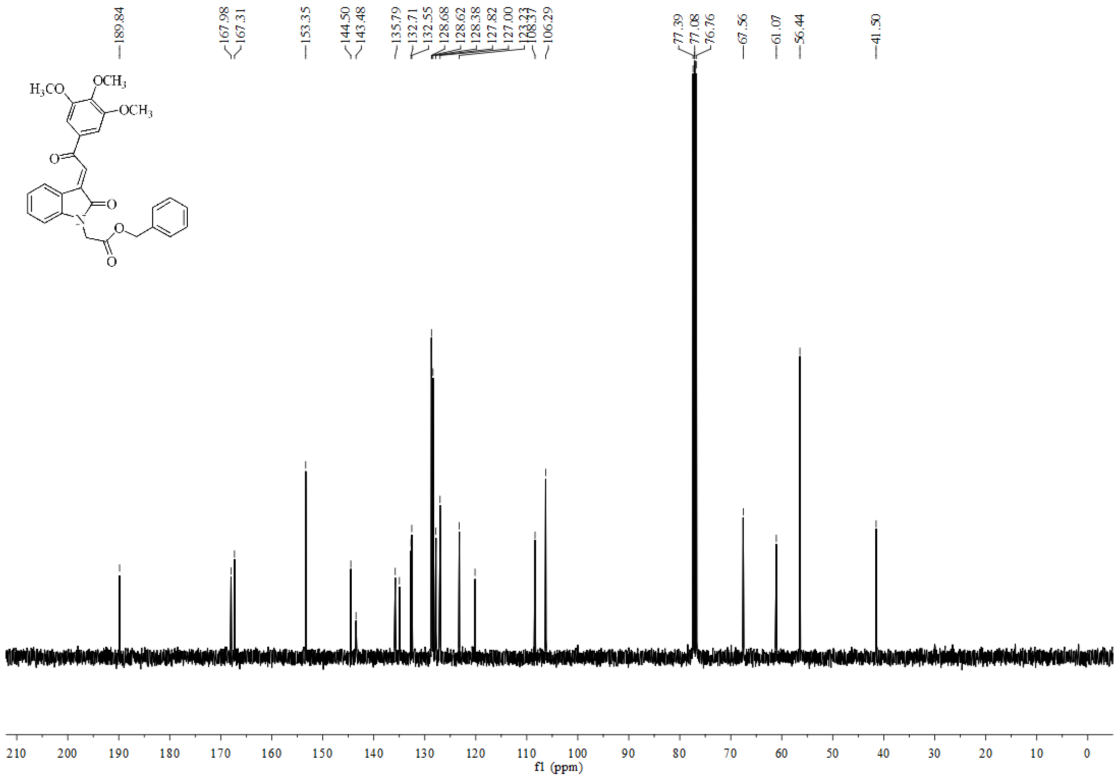

Figure S141. ^{13}C NMR Spectrum of compound **5e**

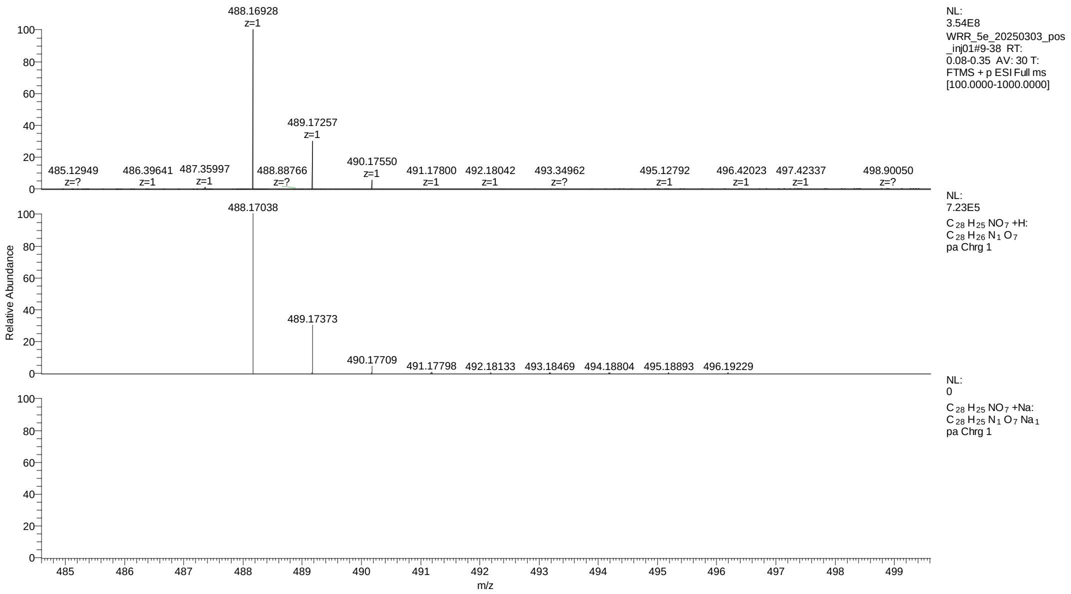

Figure S142. HRMS Spectrum of compound **5e**

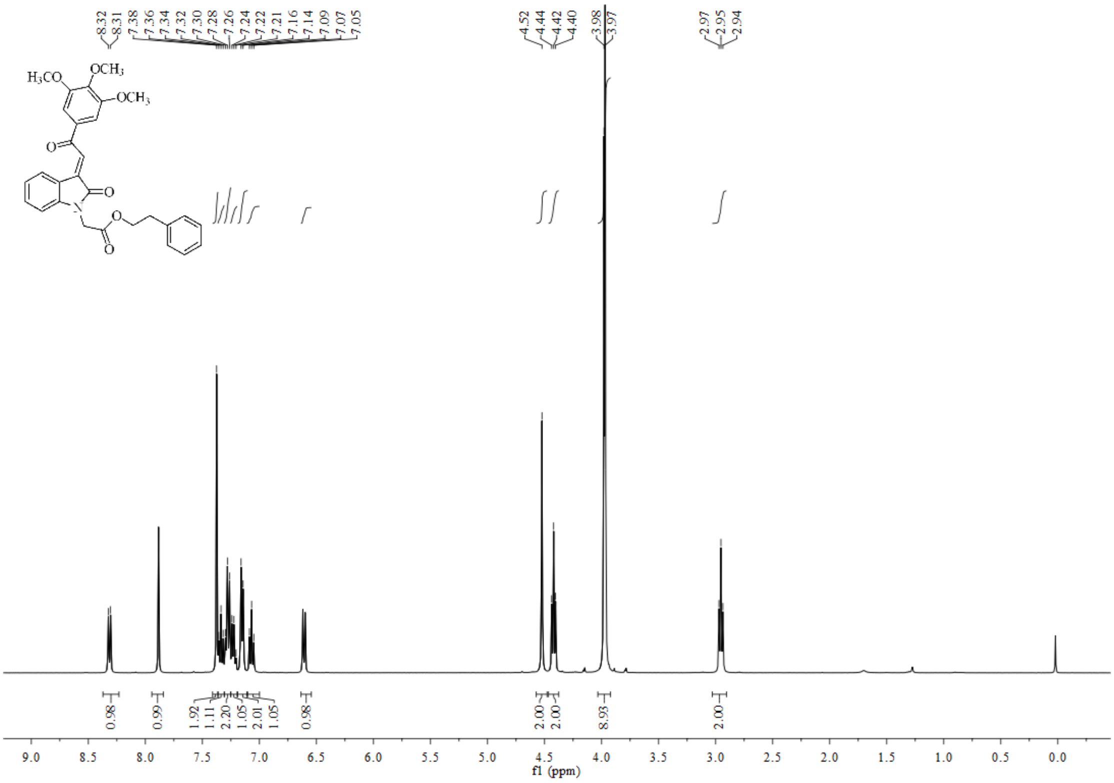

Figure S143. ^{1}H NMR Spectrum of compound **5f**

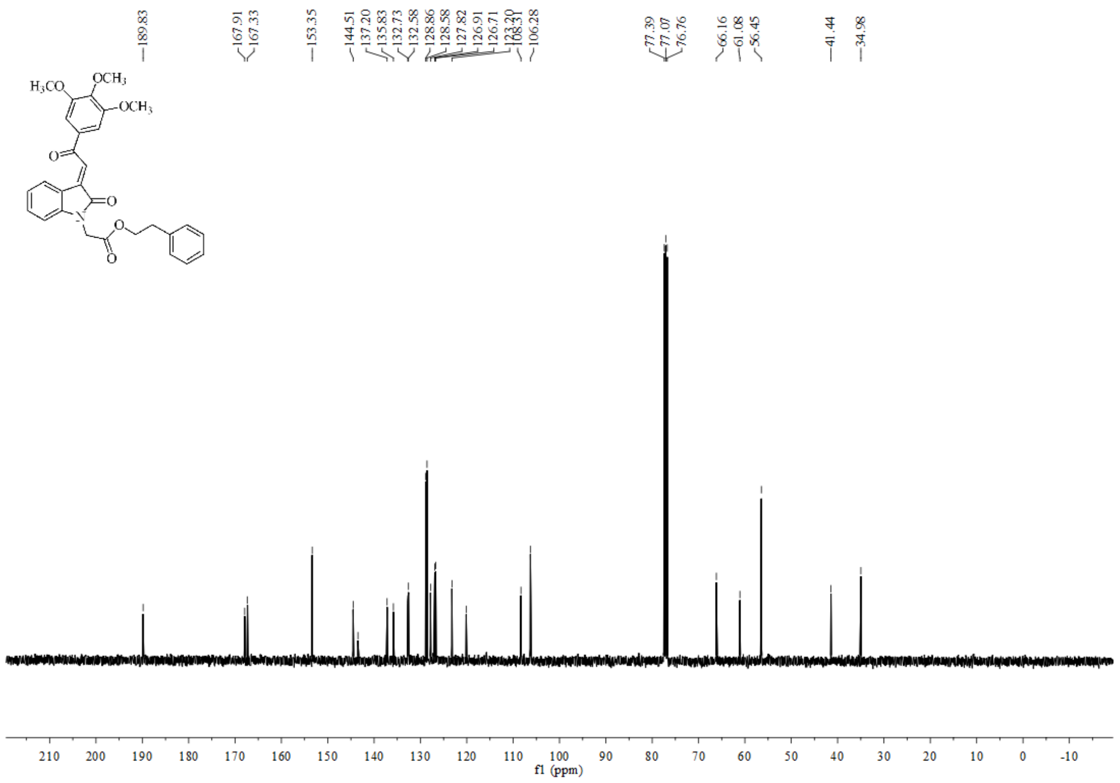

Figure S144. ^{13}C NMR Spectrum of compound **5f**

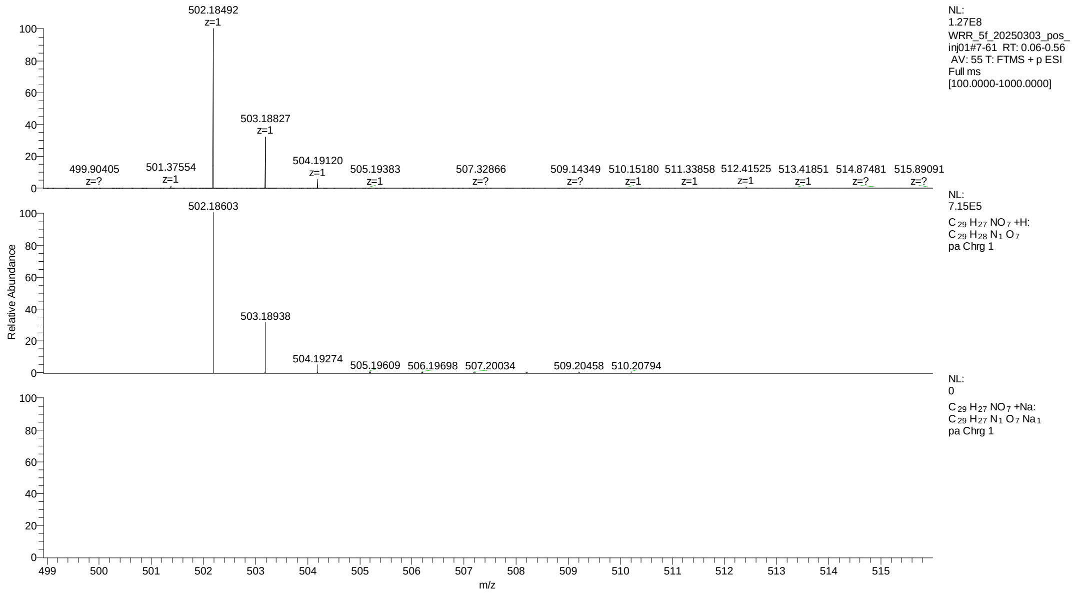

Figure S145. HRMS Spectrum of compound **5f**

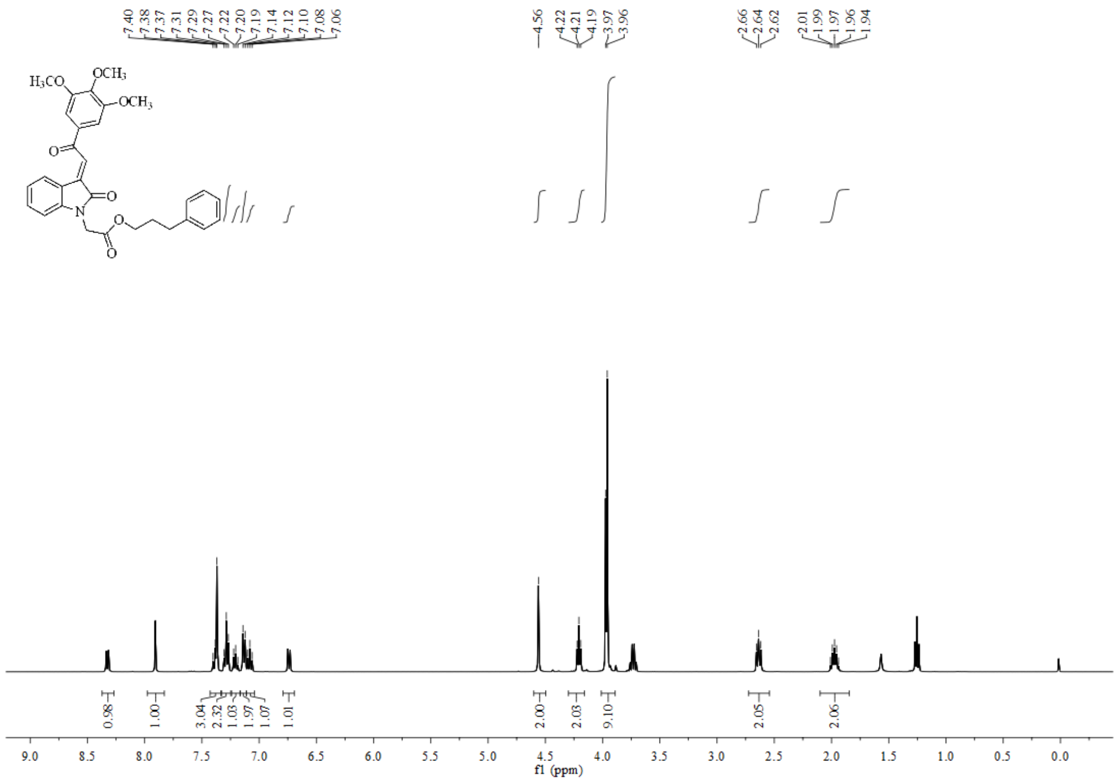

Figure S146. ^{1}H NMR Spectrum of compound **5g**

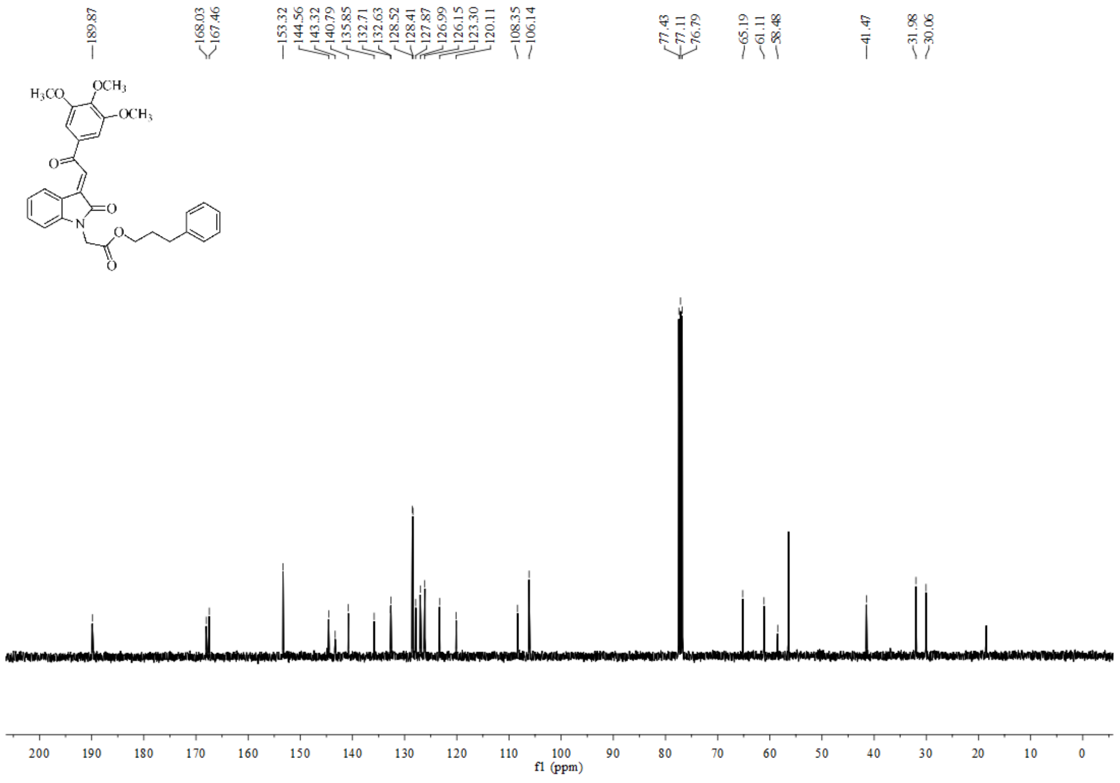

Figure S147. ^{13}C NMR Spectrum of compound **5g**

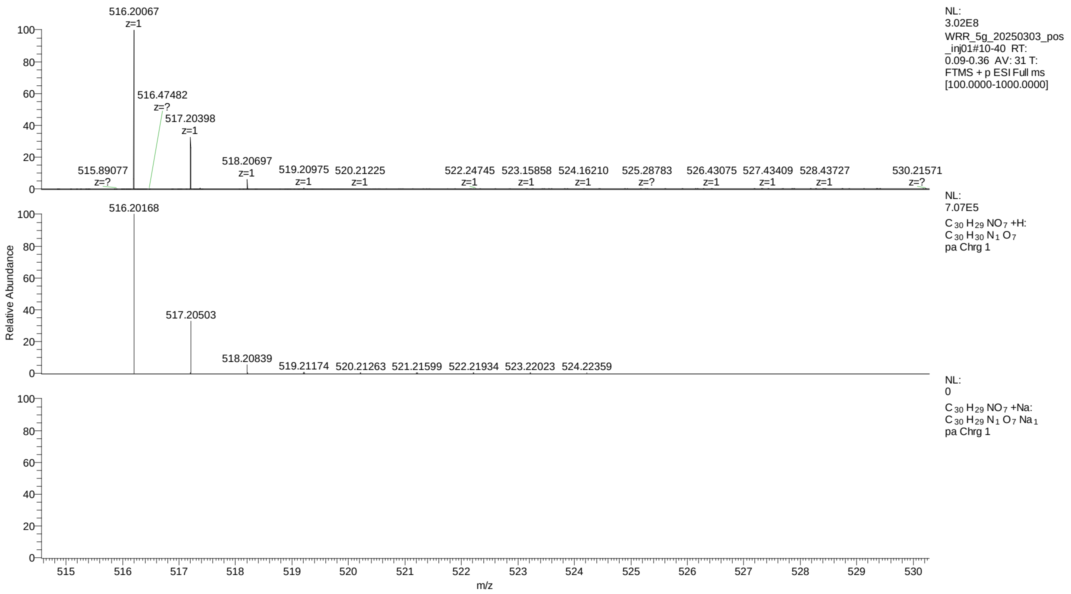

Figure S148. HRMS Spectrum of compound **5g**

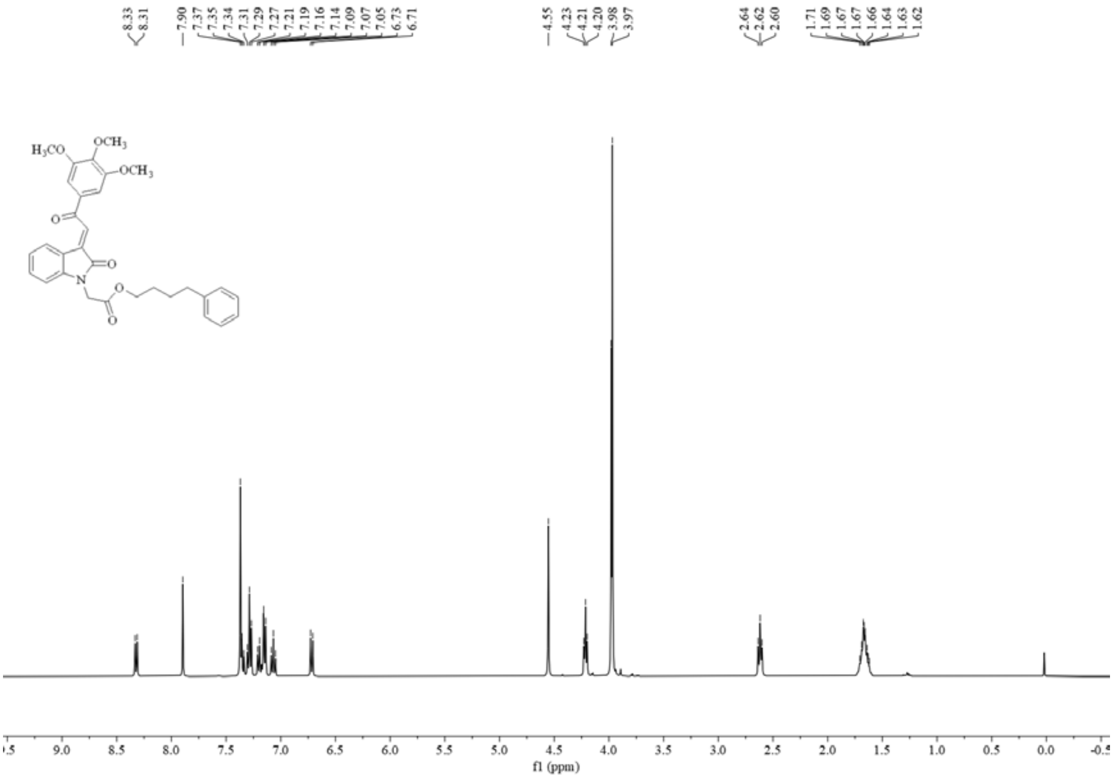

Figure S149. ^{1}H NMR Spectrum of compound **5h**

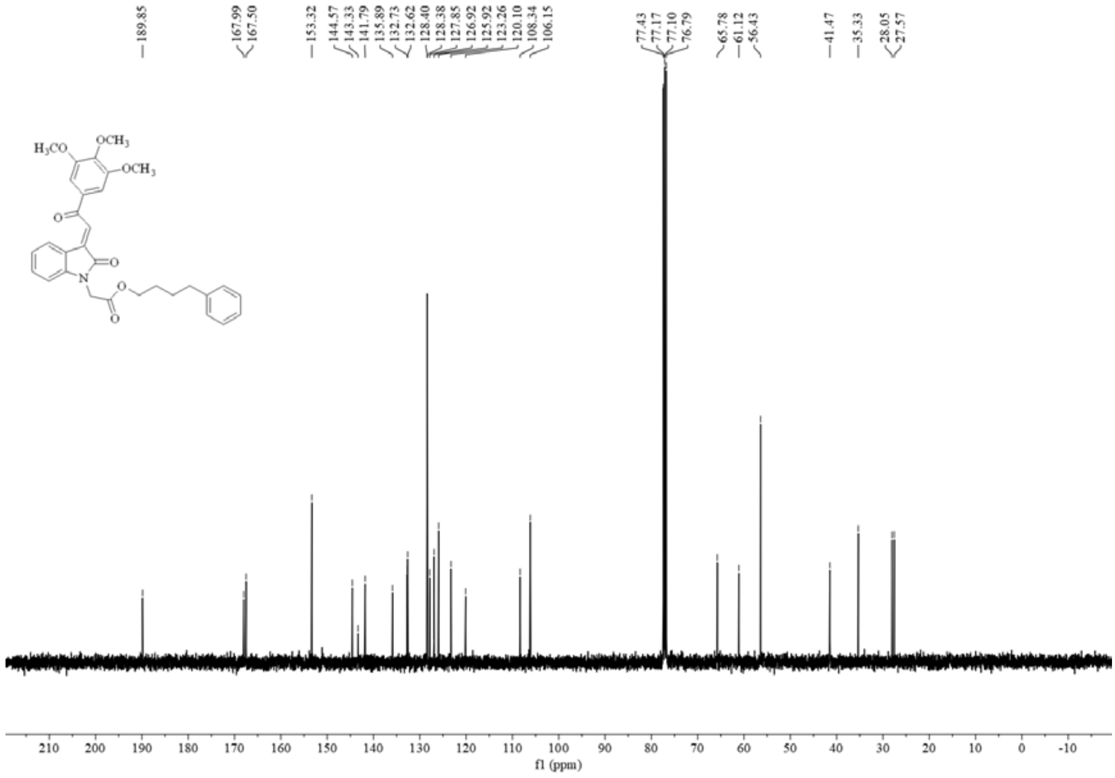

Figure S150. ^{13}C NMR Spectrum of compound **5h**

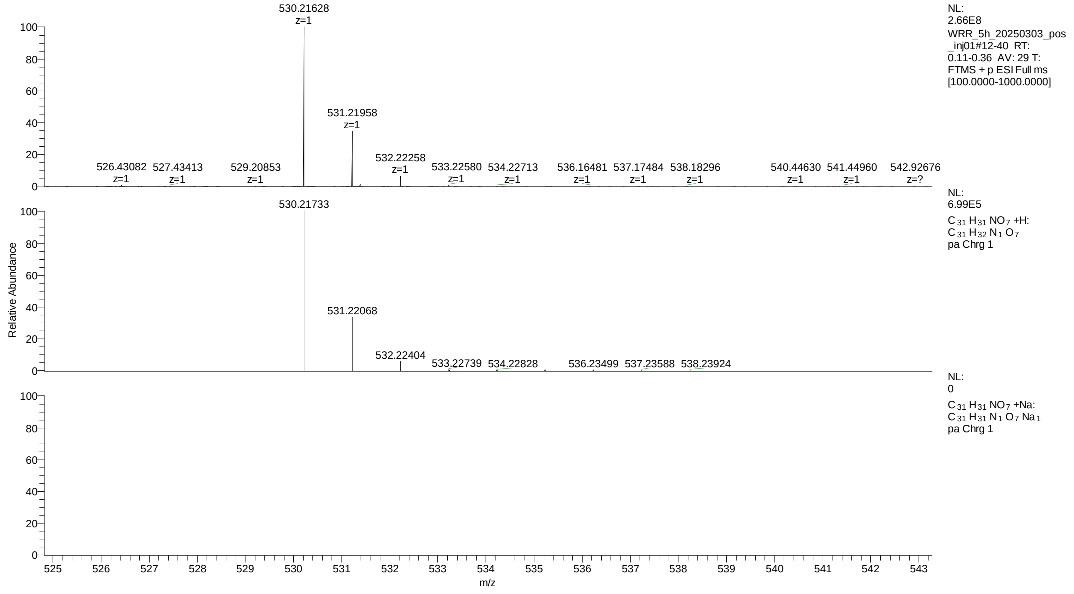

Figure S151. HRMS Spectrum of compound **5h**

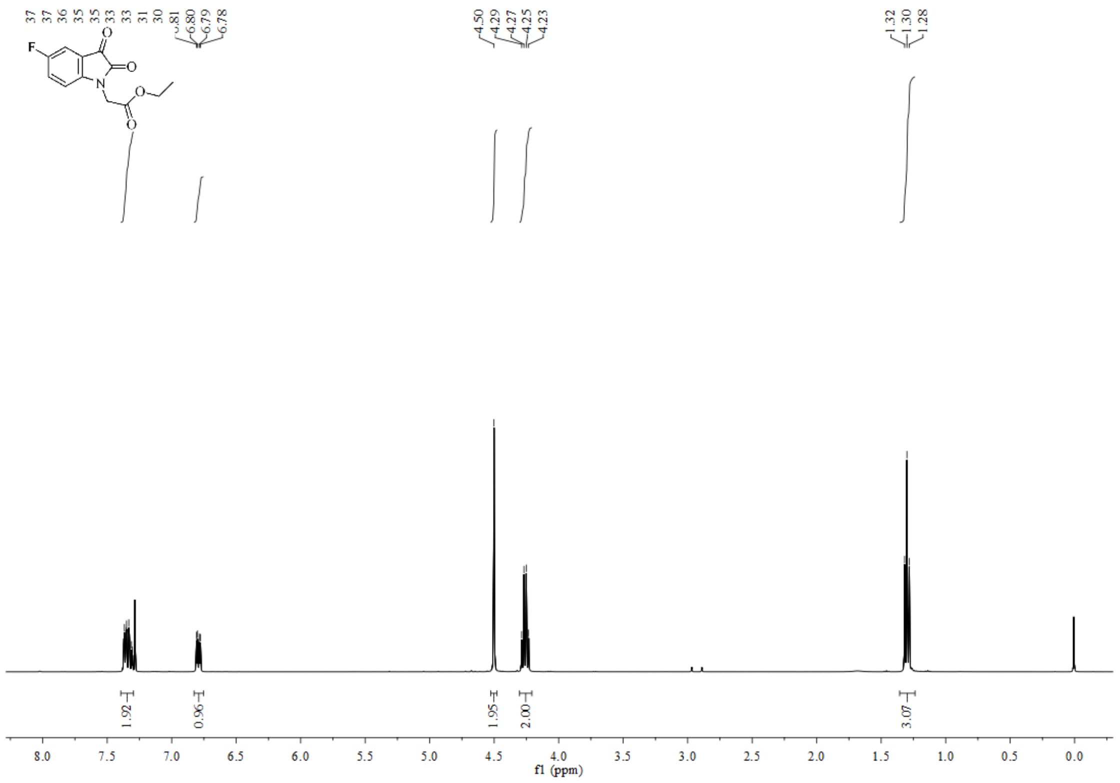

Figure S152. ^{1}H NMR Spectrum of compound **7a**

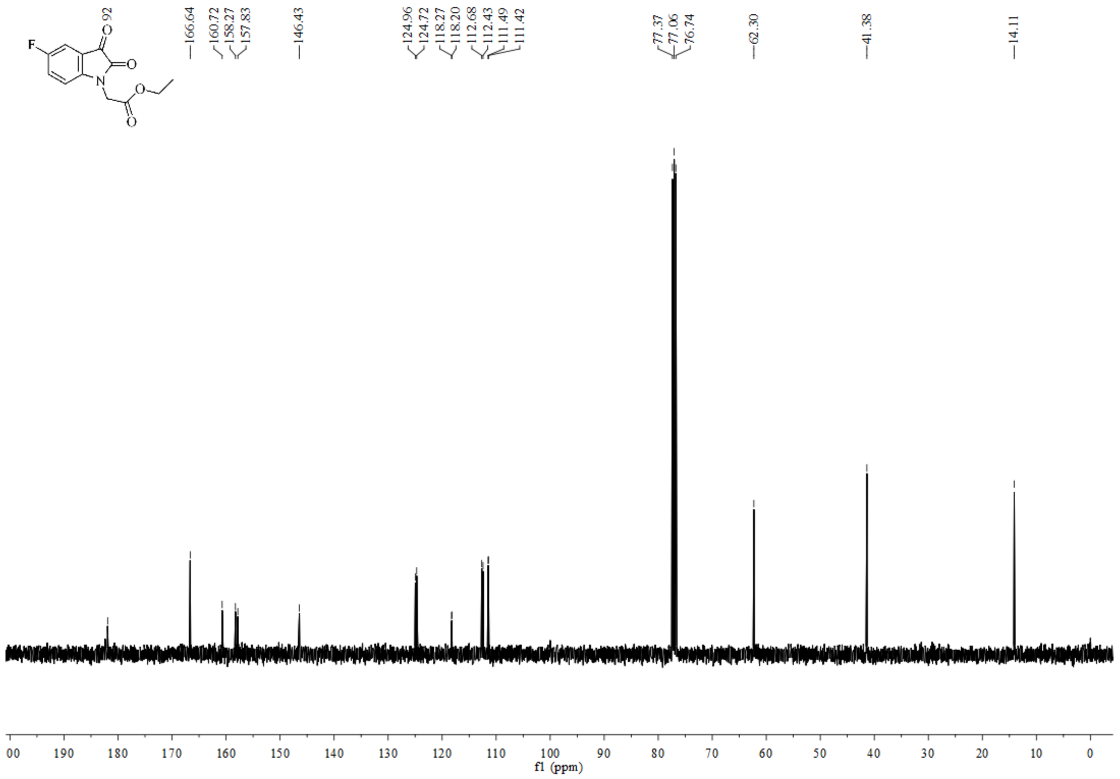

Figure S153. ^{13}C NMR Spectrum of compound **7a**

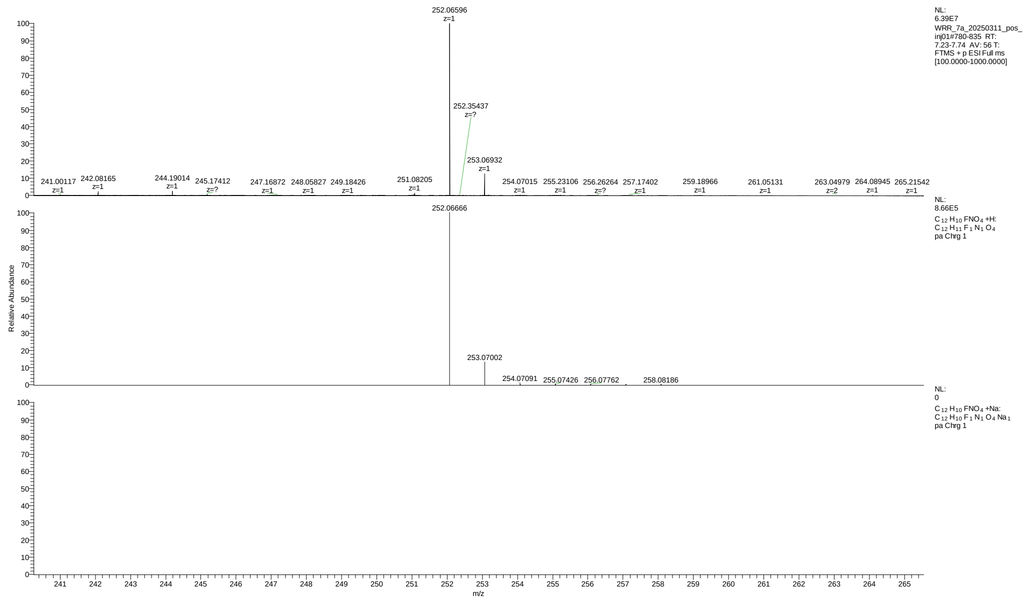

Figure S154. HRMS Spectrum of compound **7a**

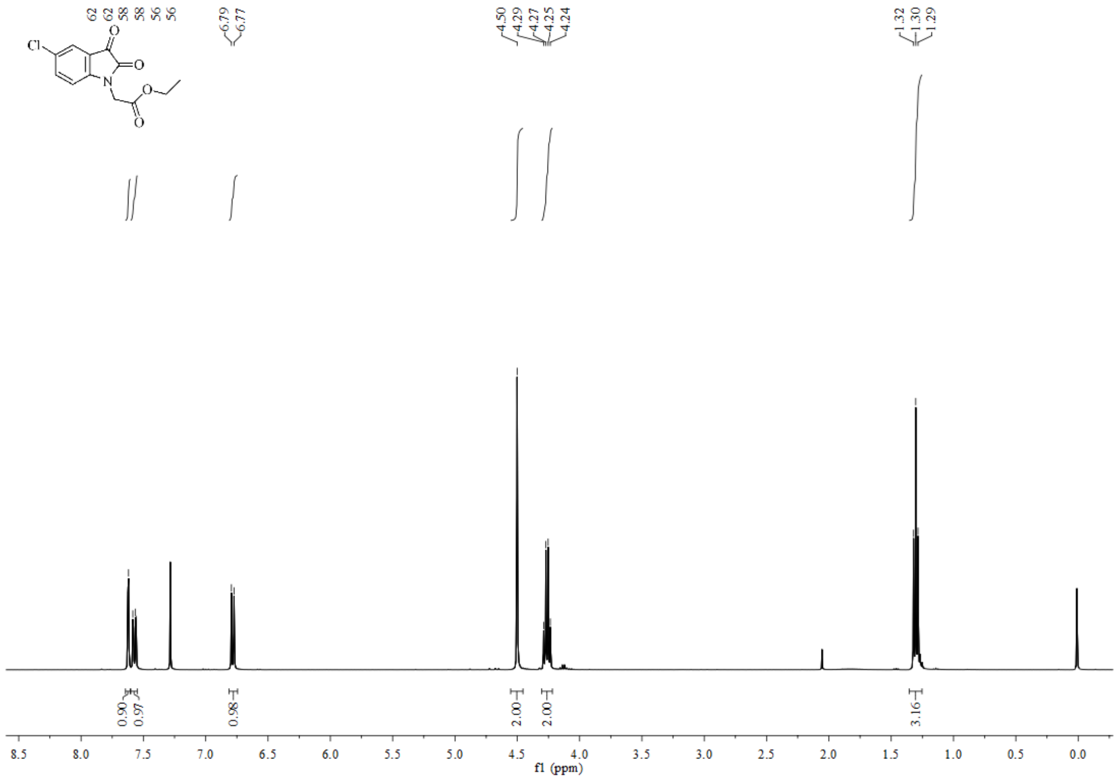

Figure S155. ^{1}H NMR Spectrum of compound **7b**

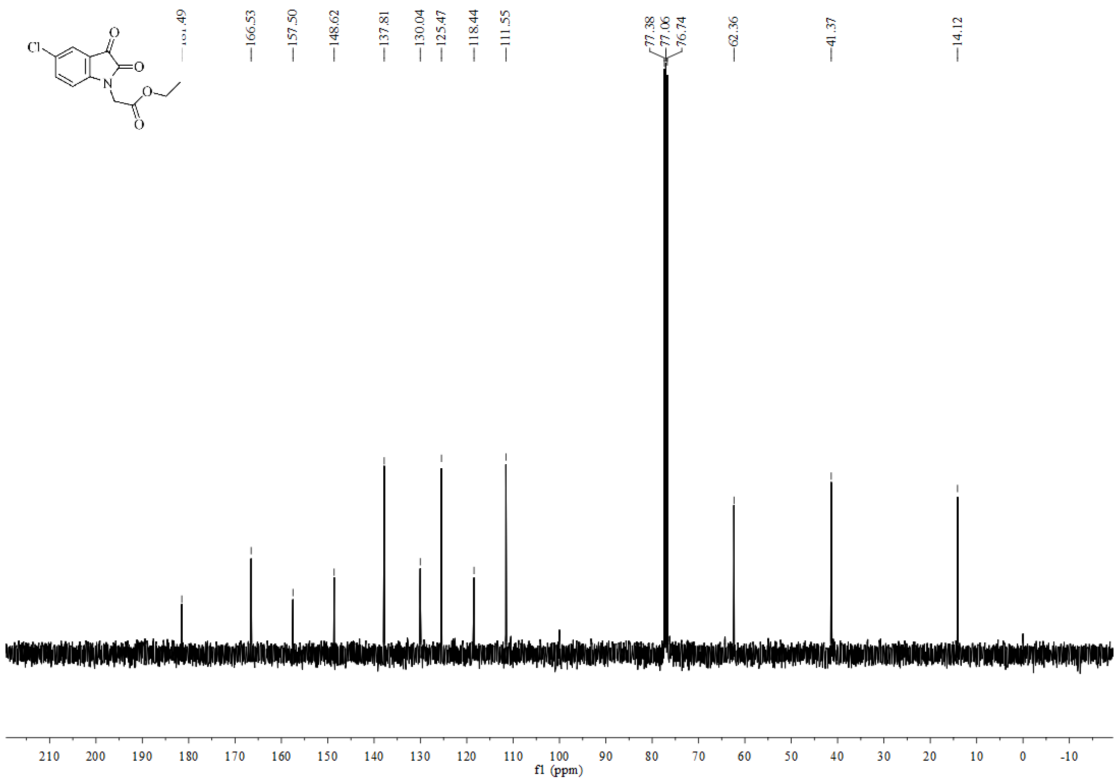

Figure S156. ^{13}C NMR Spectrum of compound **7b**

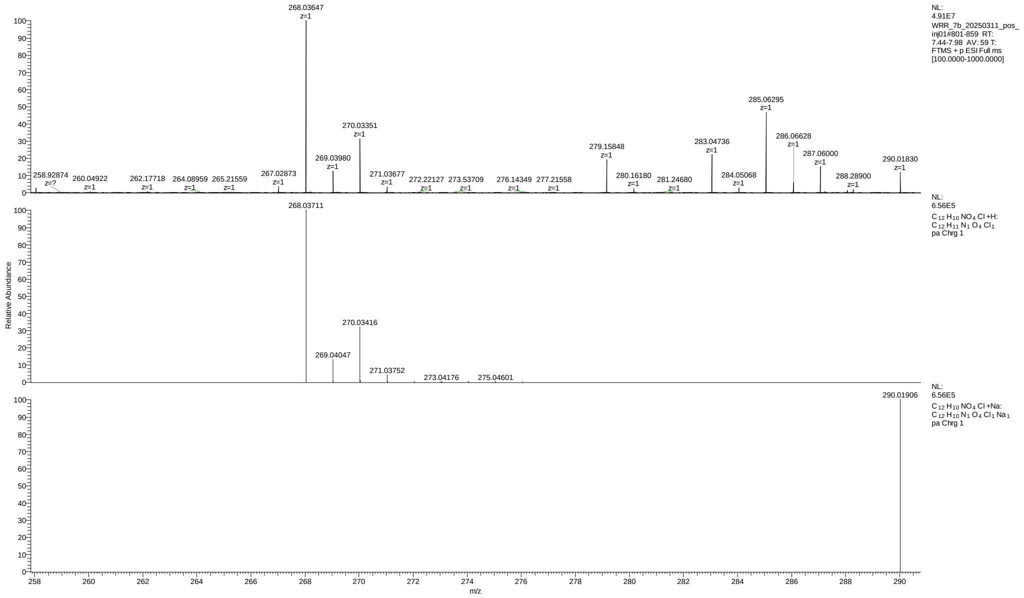

Figure S157. HRMS Spectrum of compound **7b**

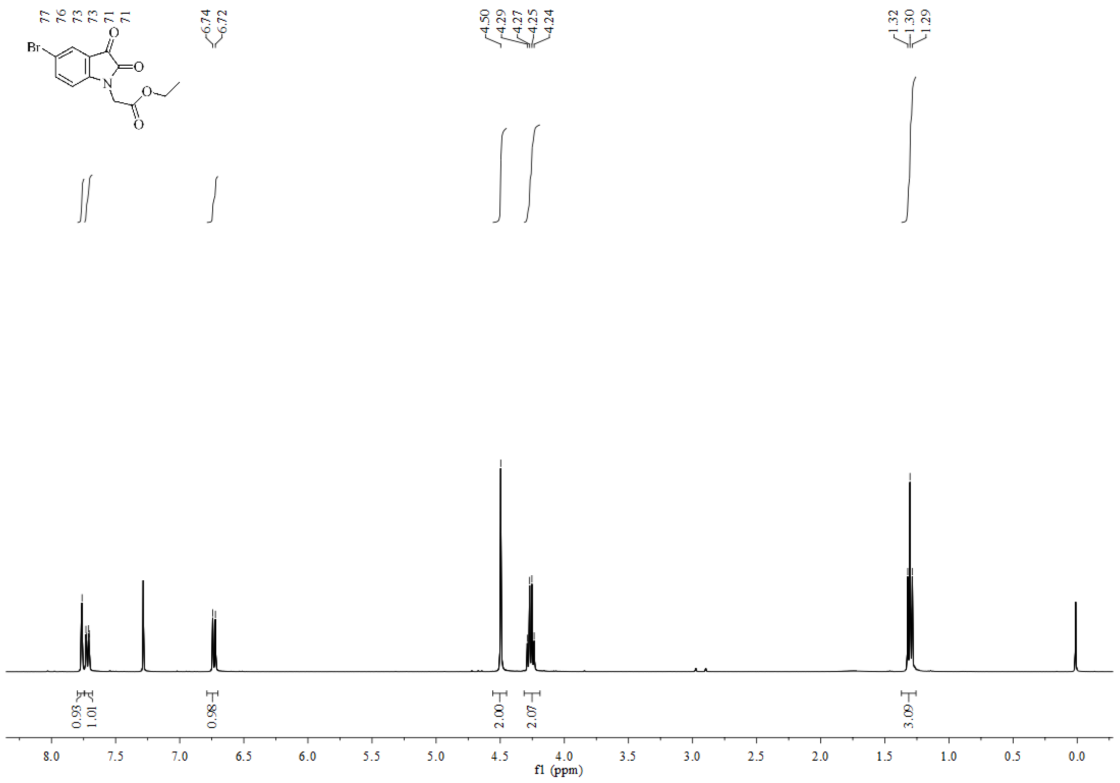

Figure S158. ^{1}H NMR Spectrum of compound **7c**

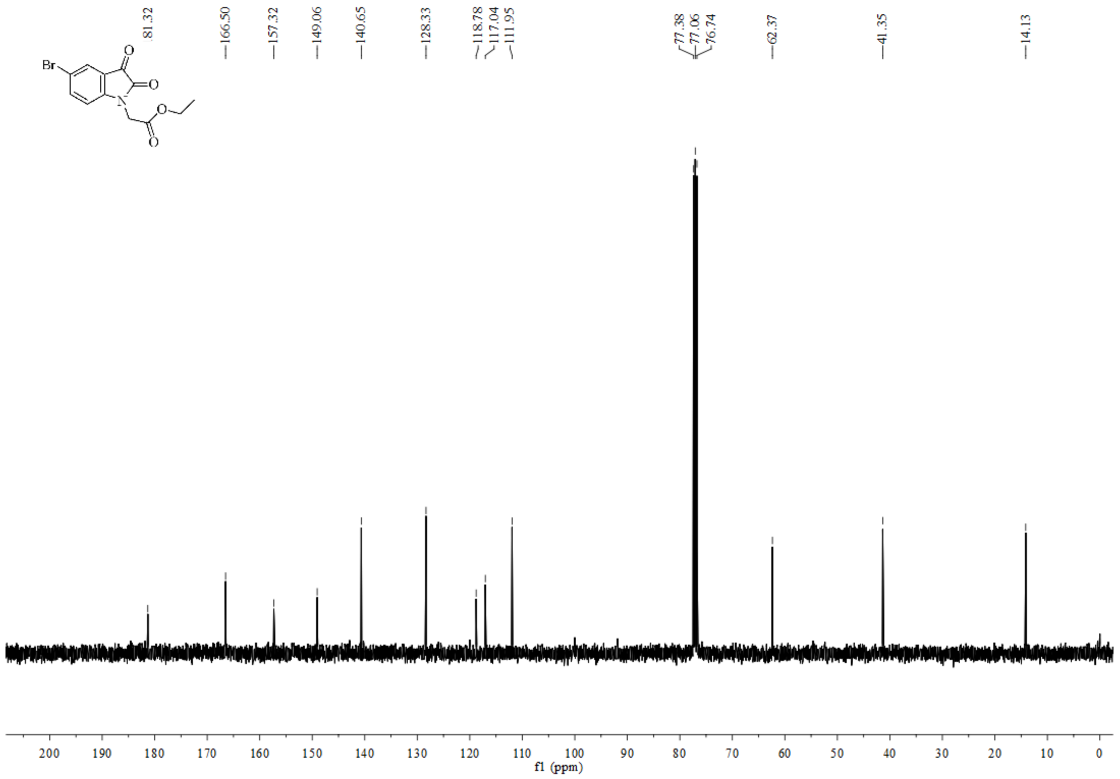

Figure S159. ^{13}C NMR Spectrum of compound **7c**

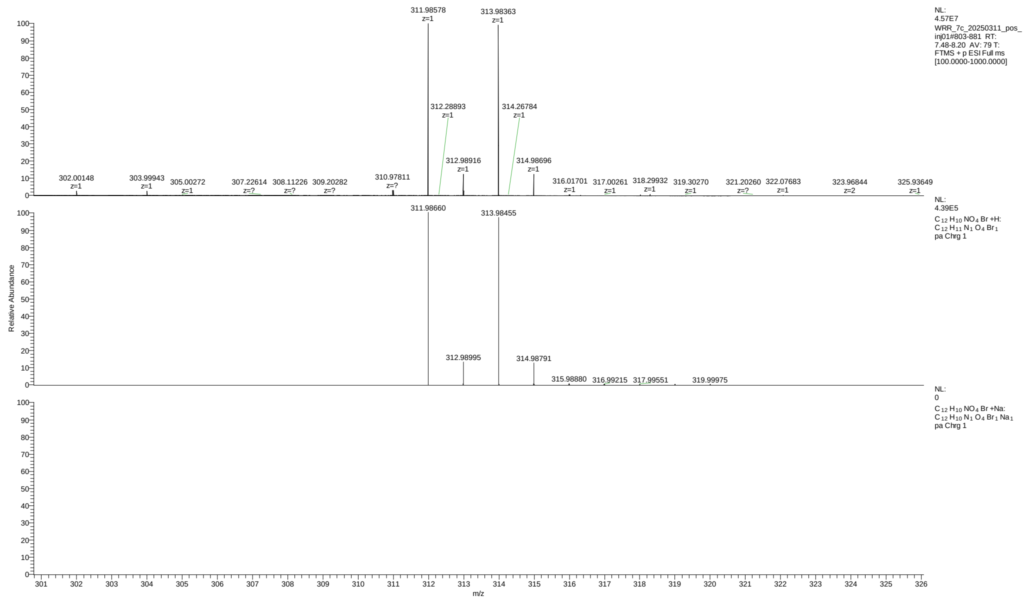

Figure S160. HRMS Spectrum of compound **7c**

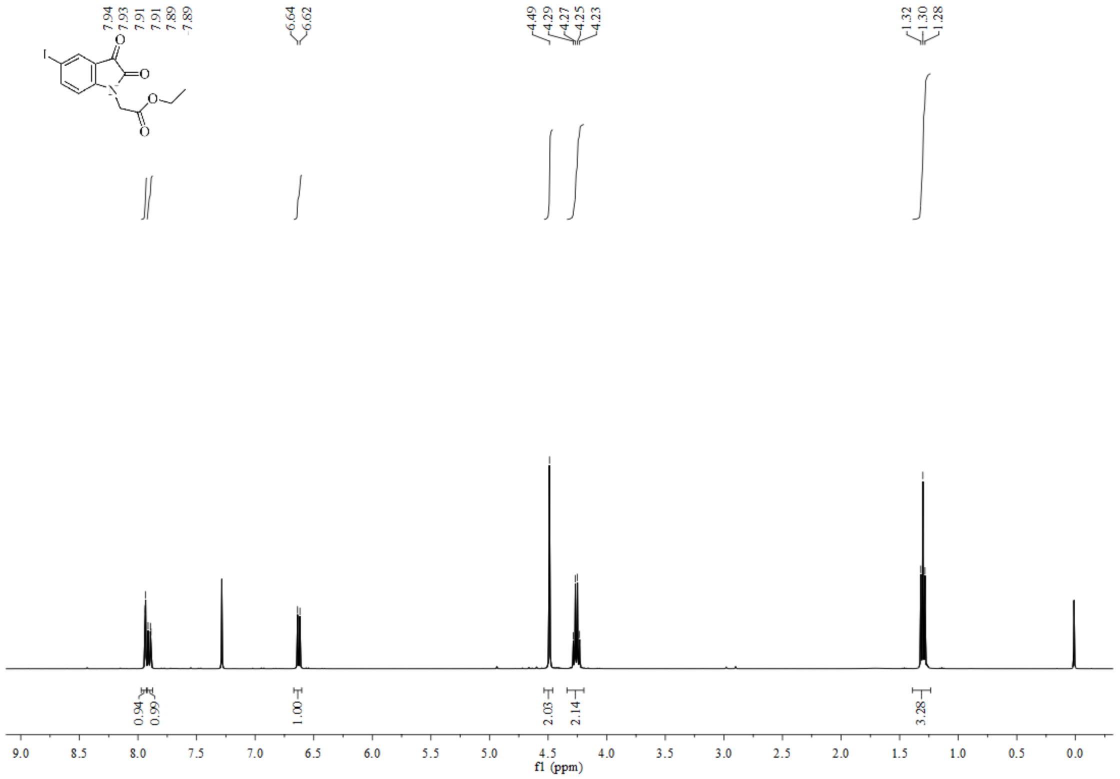

Figure S161. ^{1}H NMR Spectrum of compound **7d**

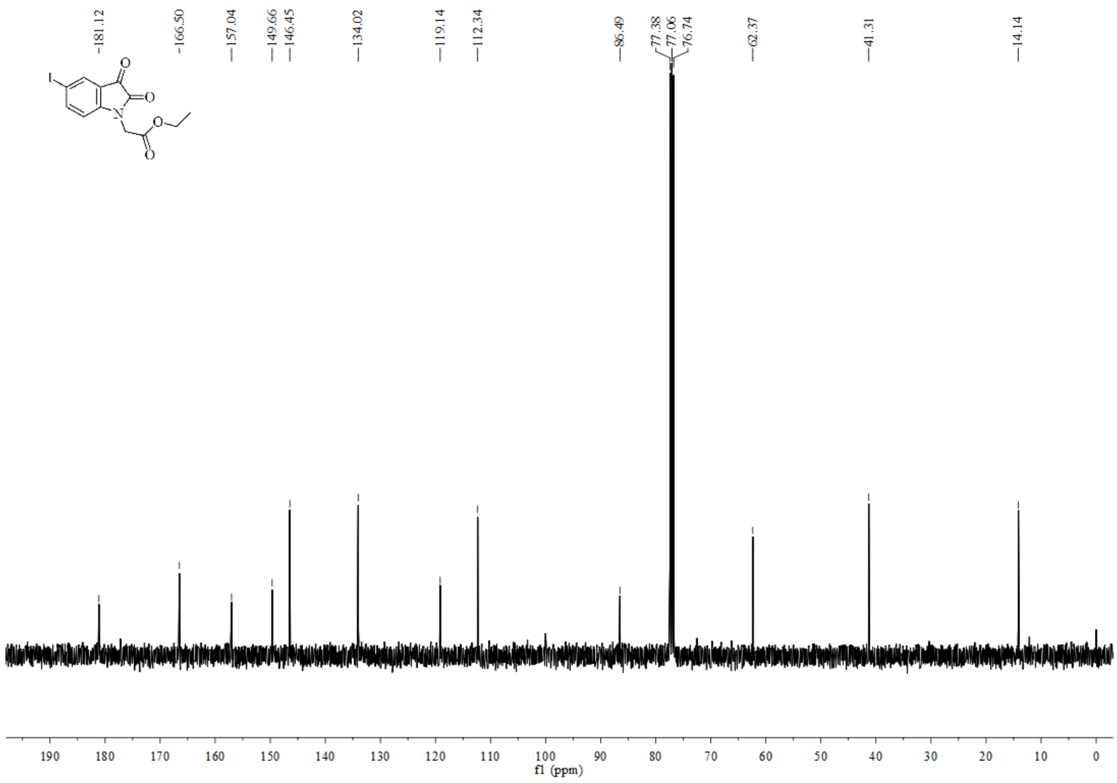

Figure S162. ^{13}C NMR Spectrum of compound **7d**

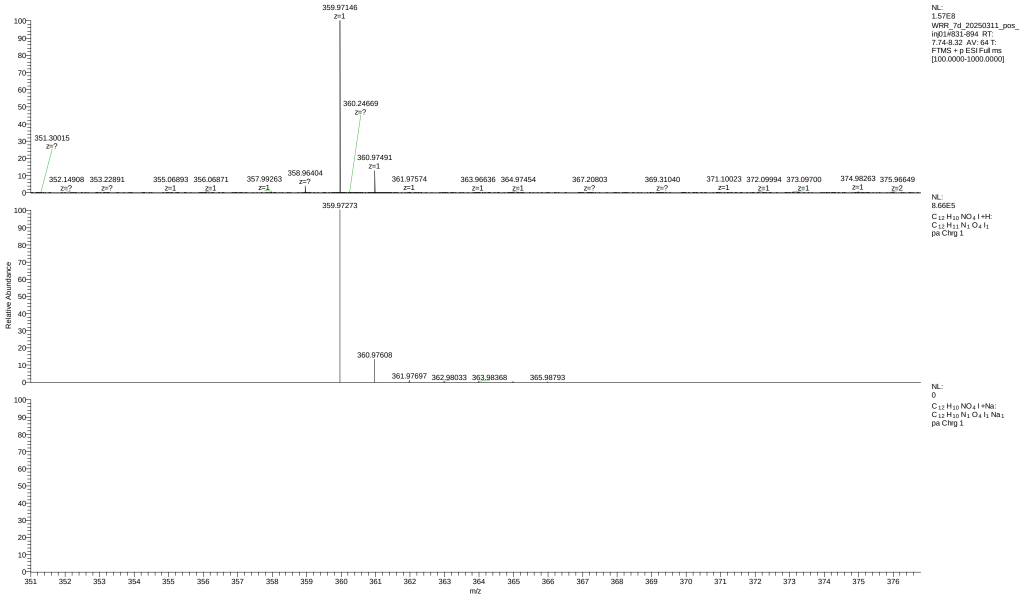

Figure S163. HRMS Spectrum of compound **7d**

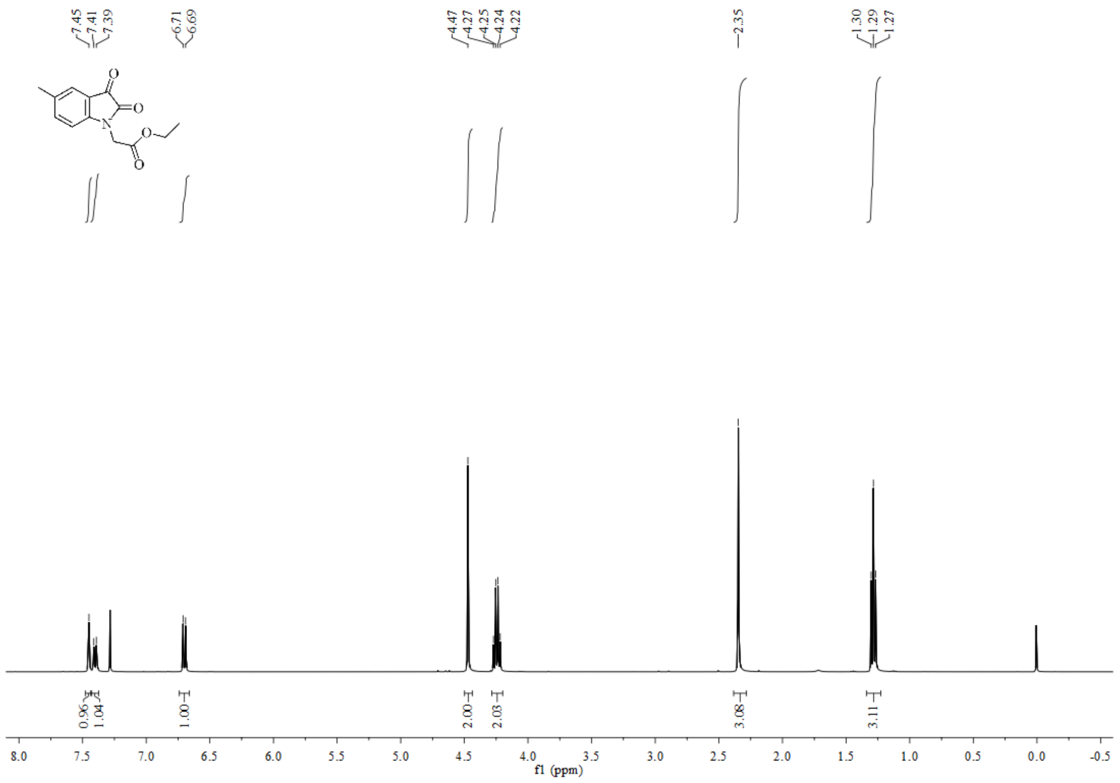

Figure S164. ^{1}H NMR Spectrum of compound **7e**

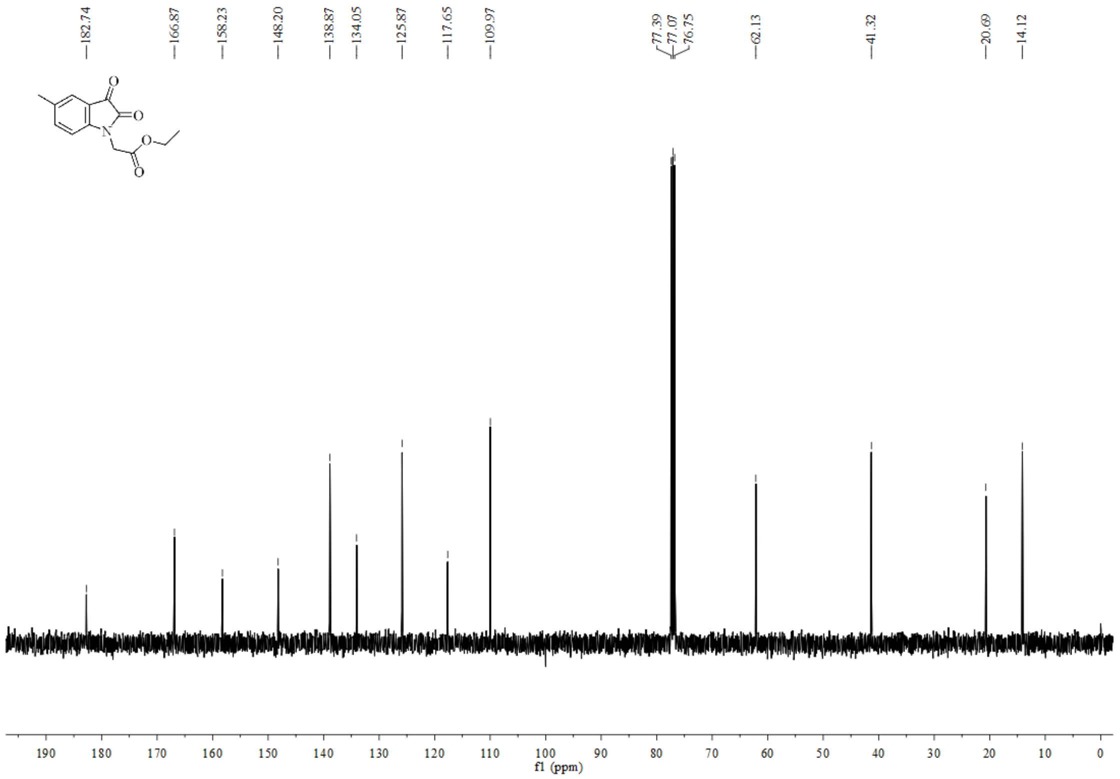

Figure S165. ^{13}C NMR Spectrum of compound **7e**

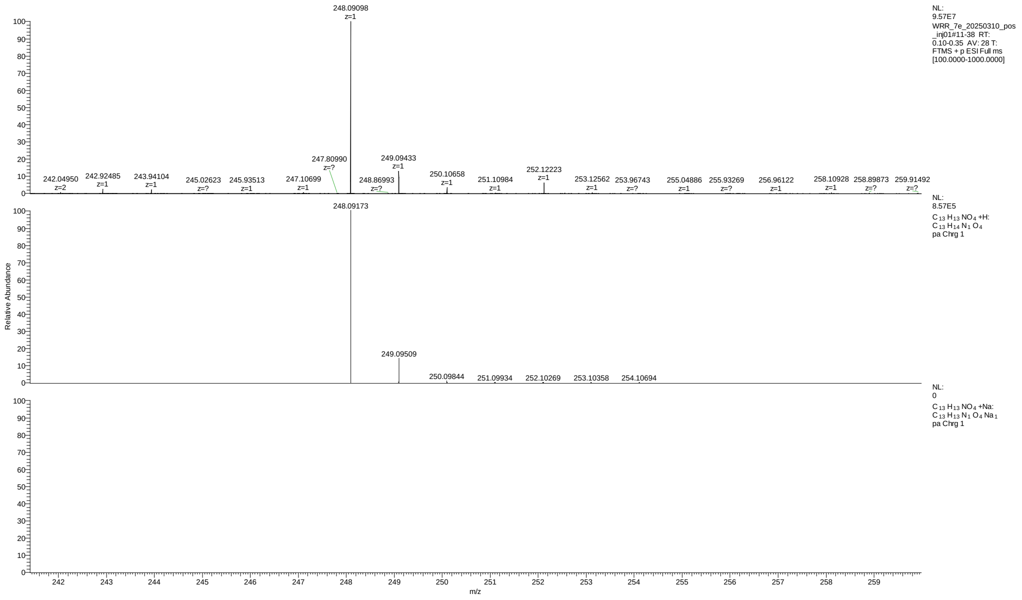

Figure S166. HRMS Spectrum of compound **7e**

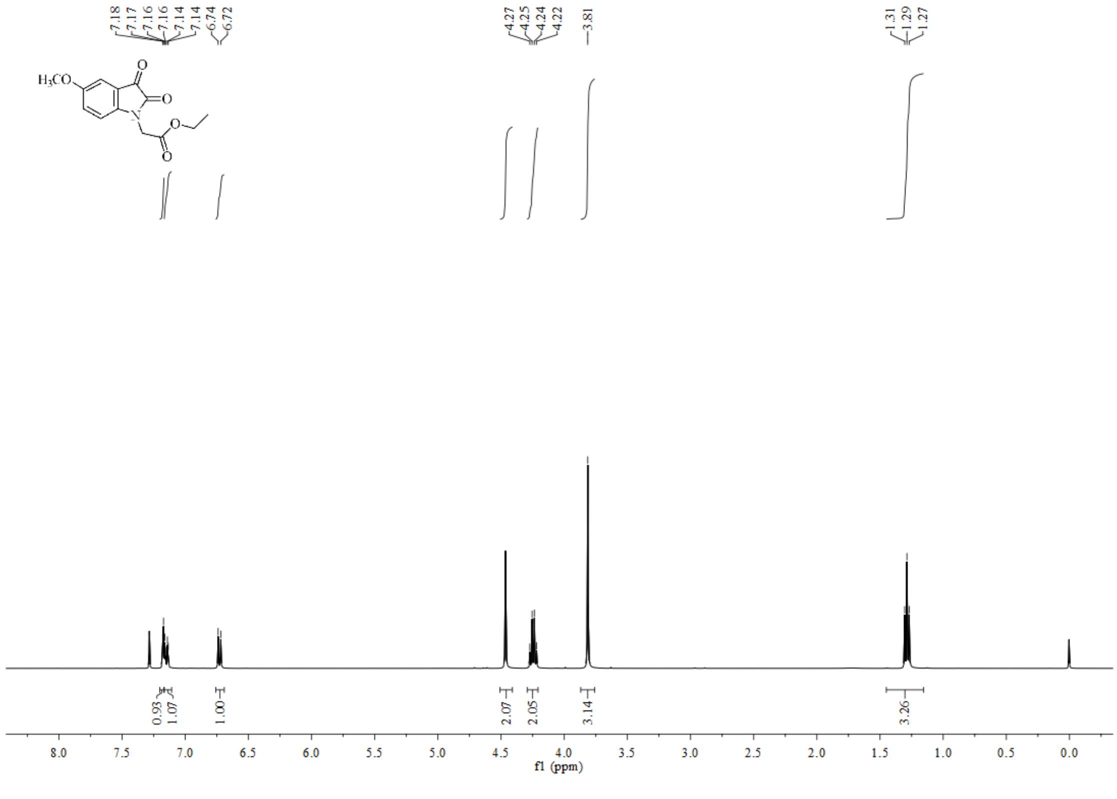

Figure S167. ^{1}H NMR Spectrum of compound **7f**

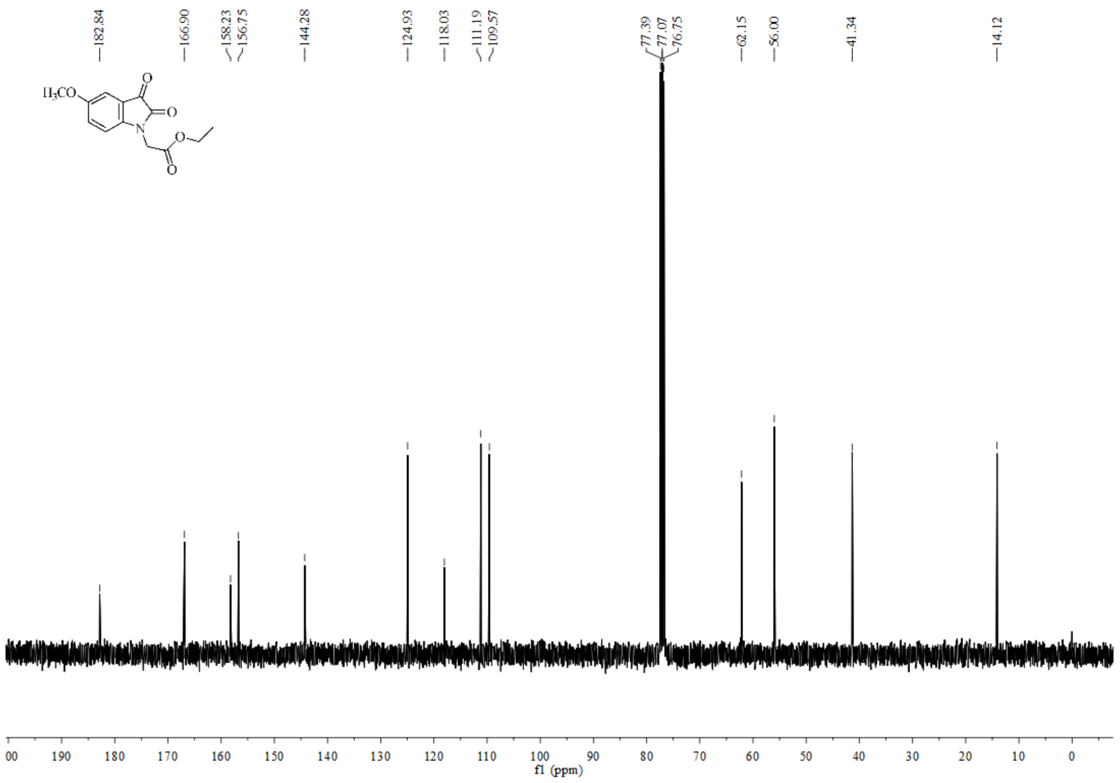

Figure S168. ^{13}C NMR Spectrum of compound **7f**

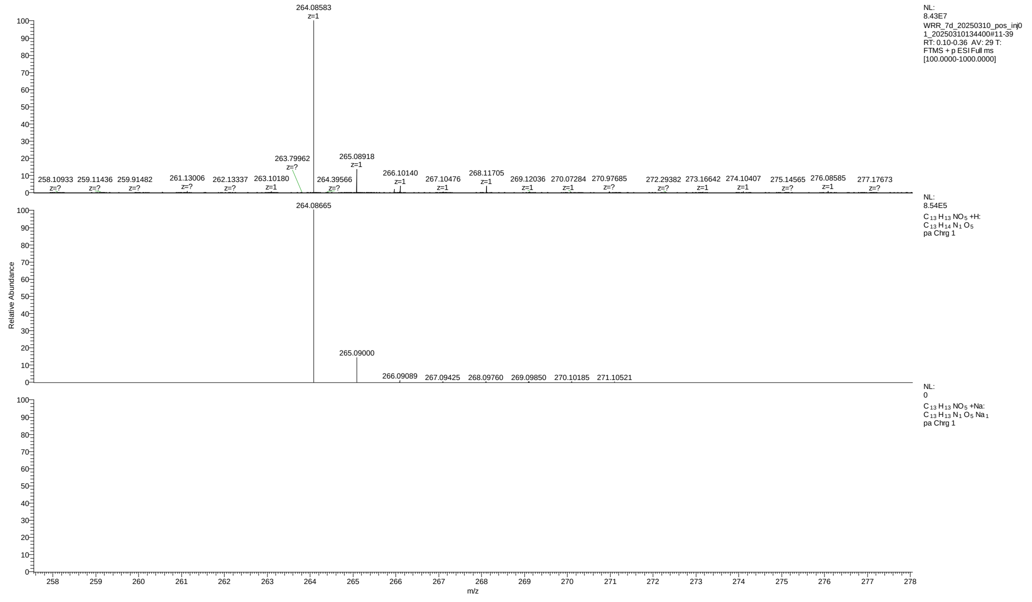

Figure S169. HRMS Spectrum of compound **7f**

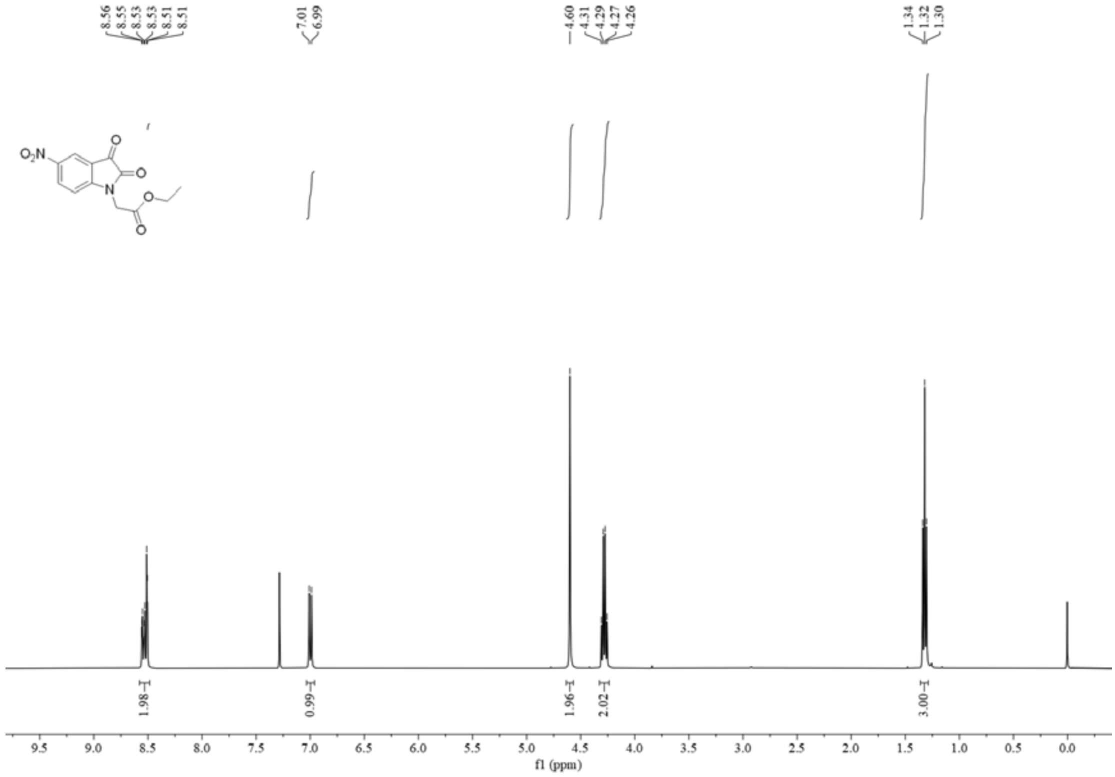

Figure S170. ^{1}H NMR Spectrum of compound **7g**

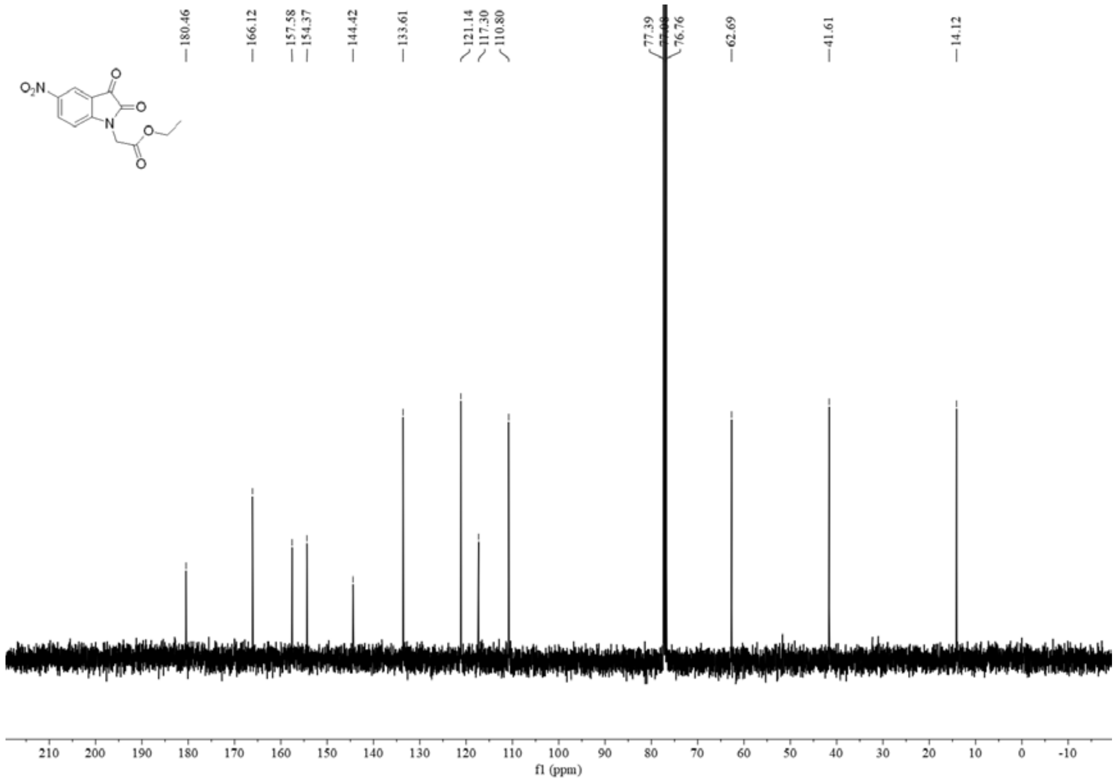

Figure S171. ^{13}C NMR Spectrum of compound **7g**

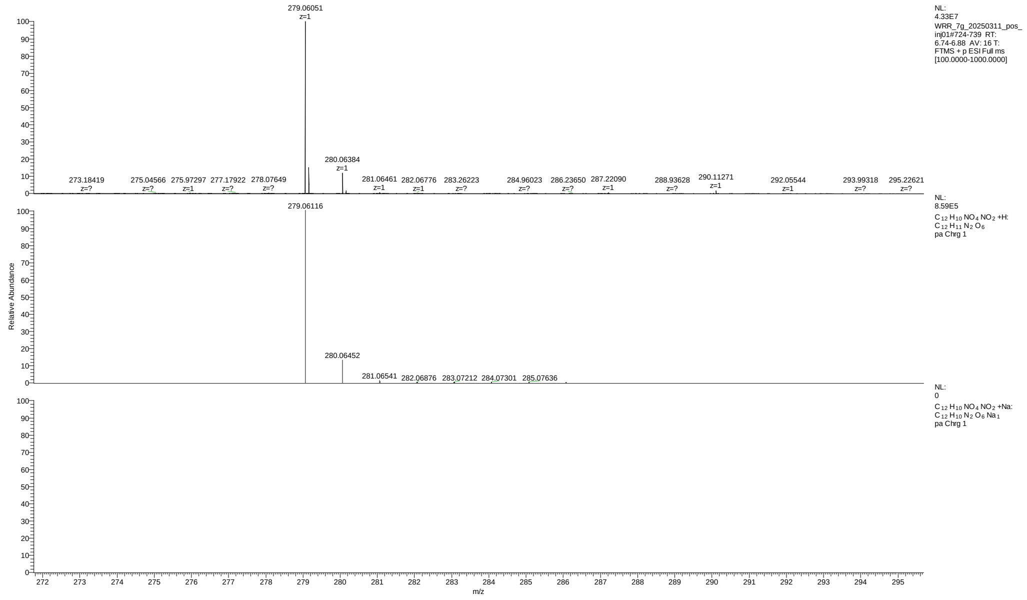

Figure S172. HRMS Spectrum of compound **7g**

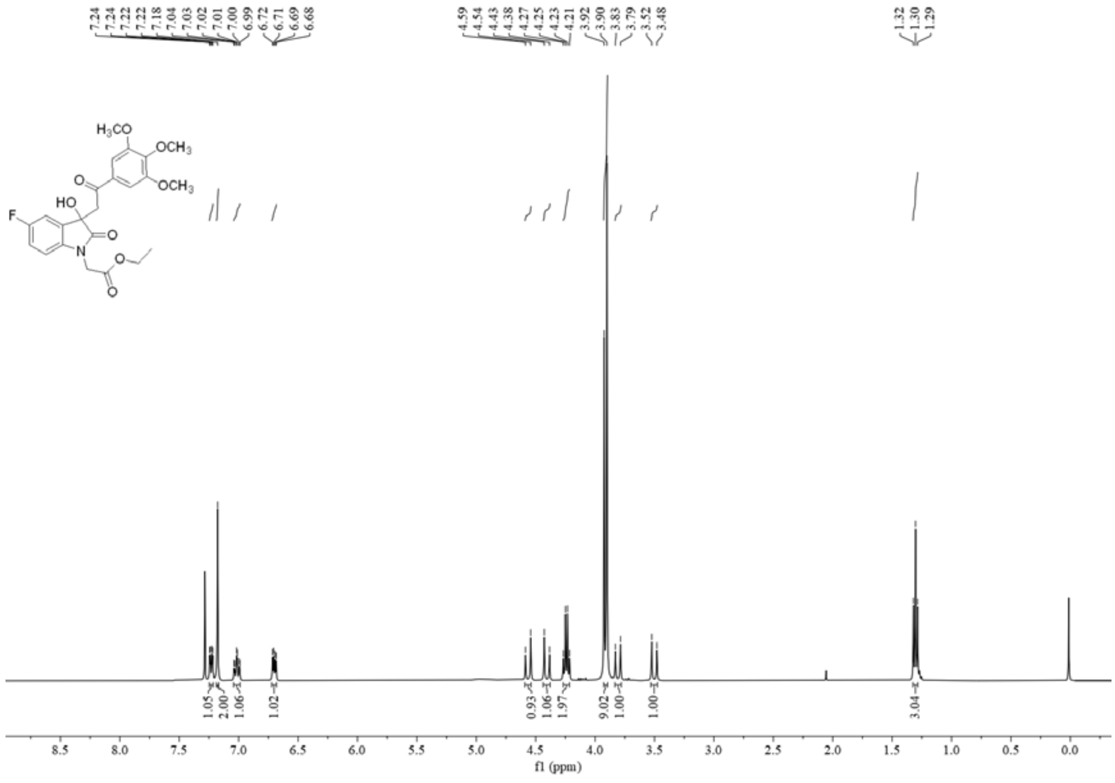

Figure S173. ^{1}H NMR Spectrum of compound **8a**

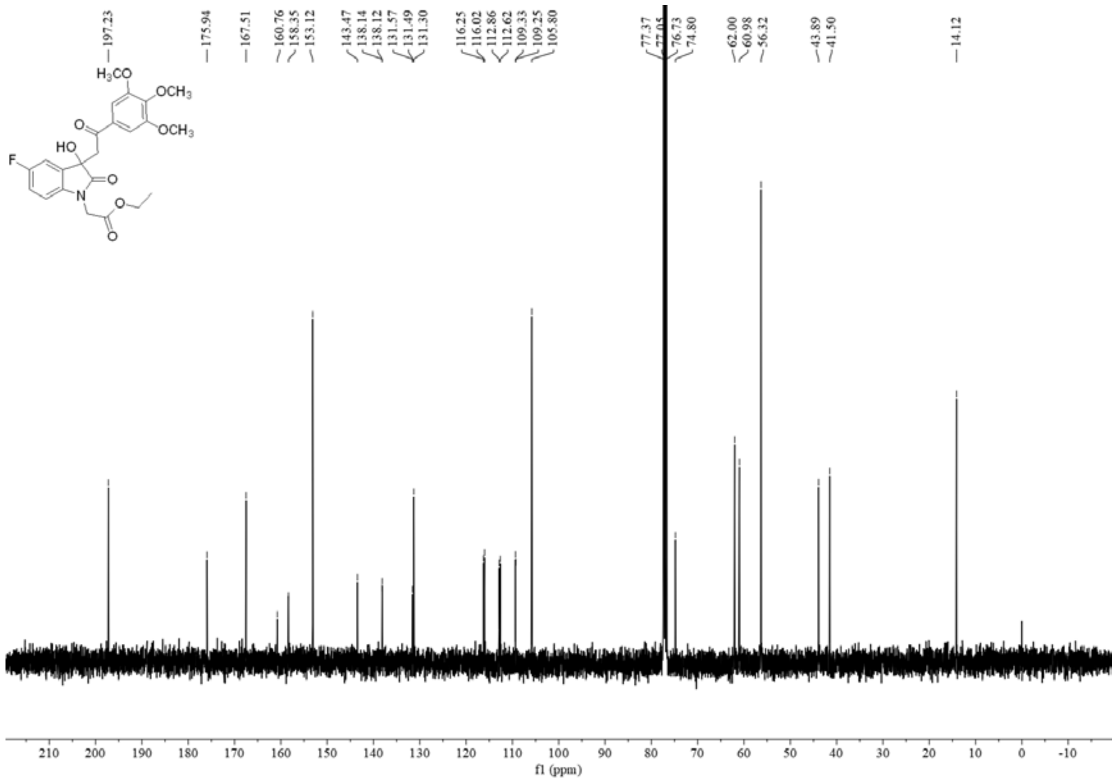

Figure S174. ^{13}C NMR Spectrum of compound **8a**

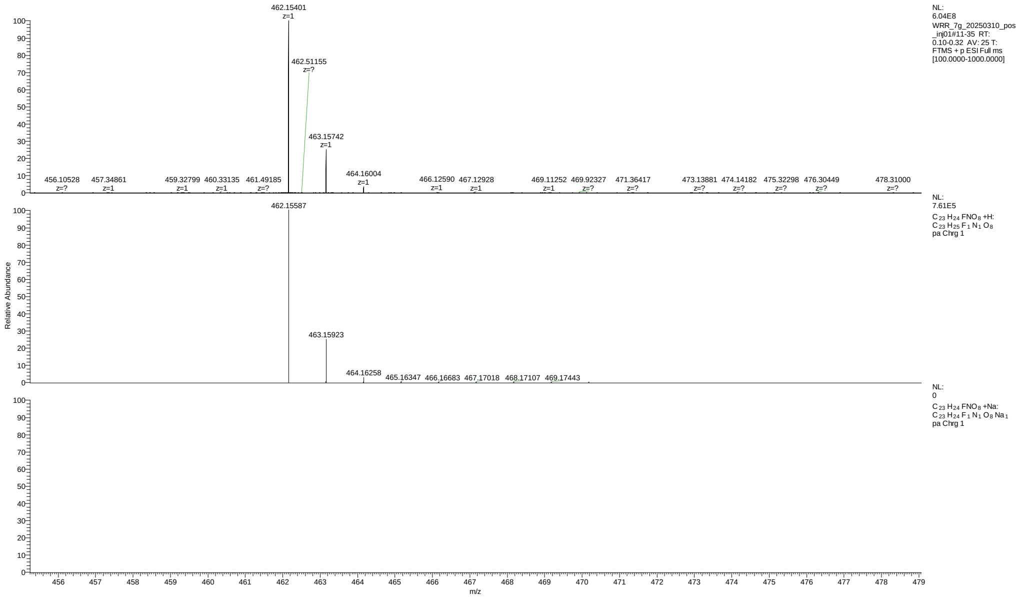

Figure S175. HRMS Spectrum of compound **8a**

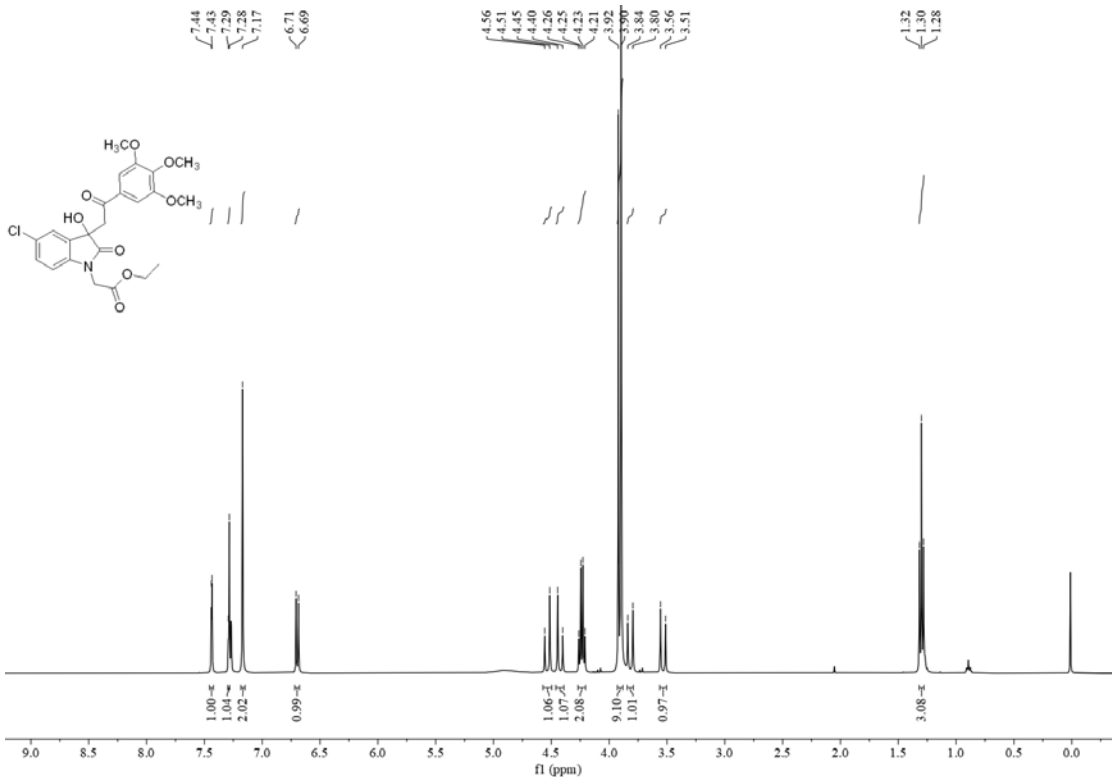

Figure S176. ^{1}H NMR Spectrum of compound **8b**

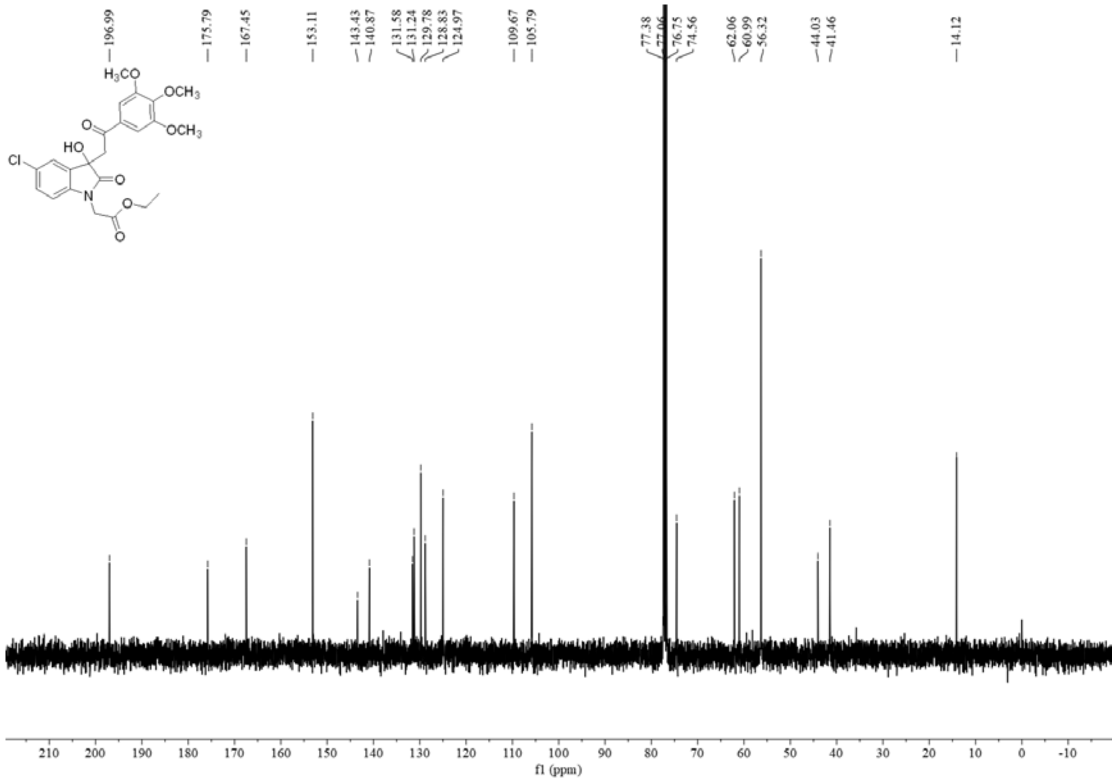

Figure S177. ^{13}C NMR Spectrum of compound **8b**

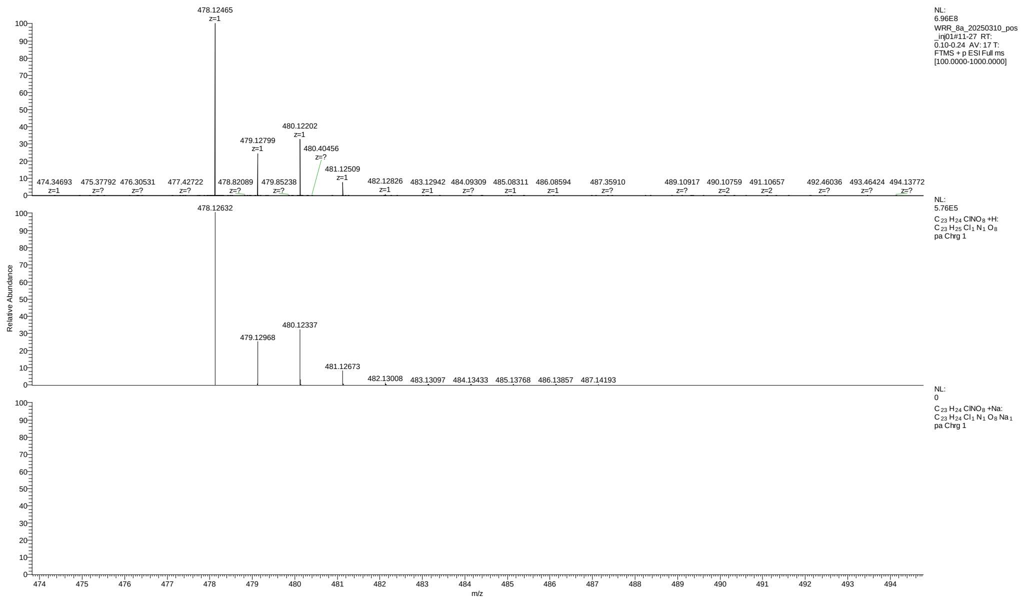

Figure S178. HRMS Spectrum of compound **8b**

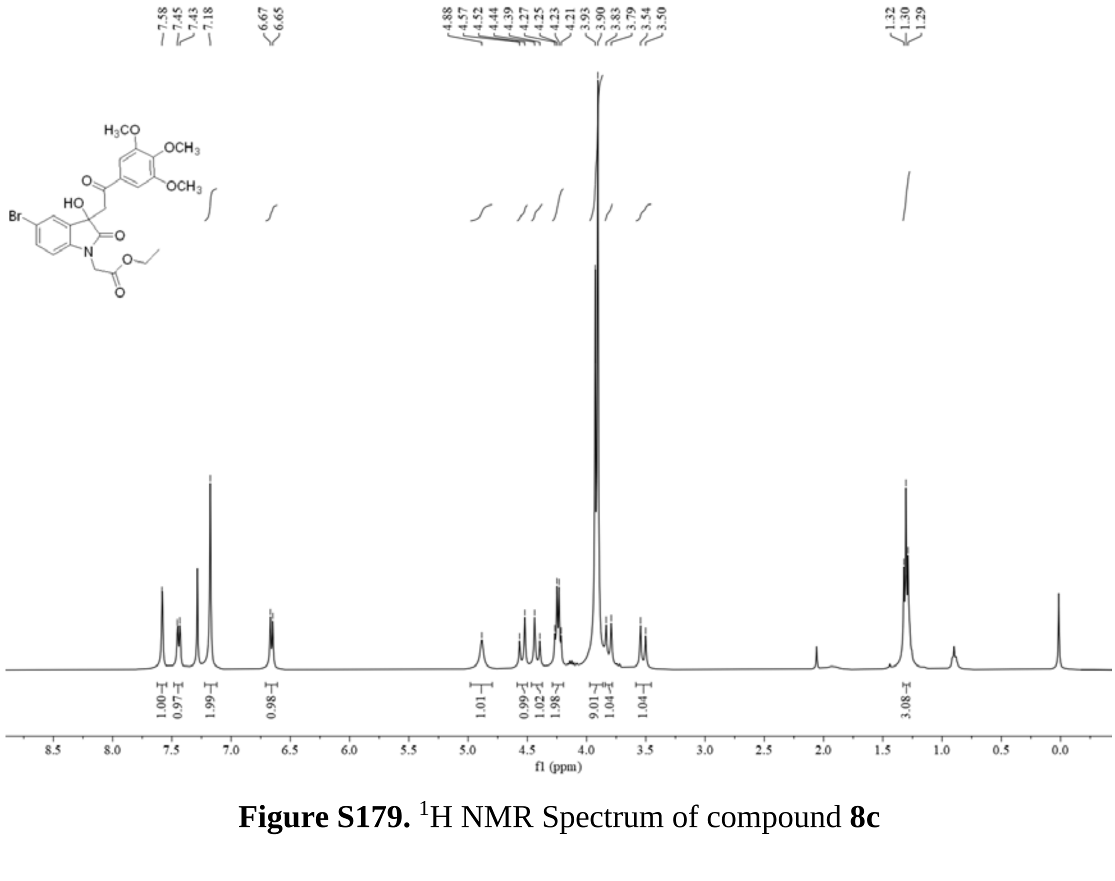

Figure S179. ^{1}H NMR Spectrum of compound **8c**

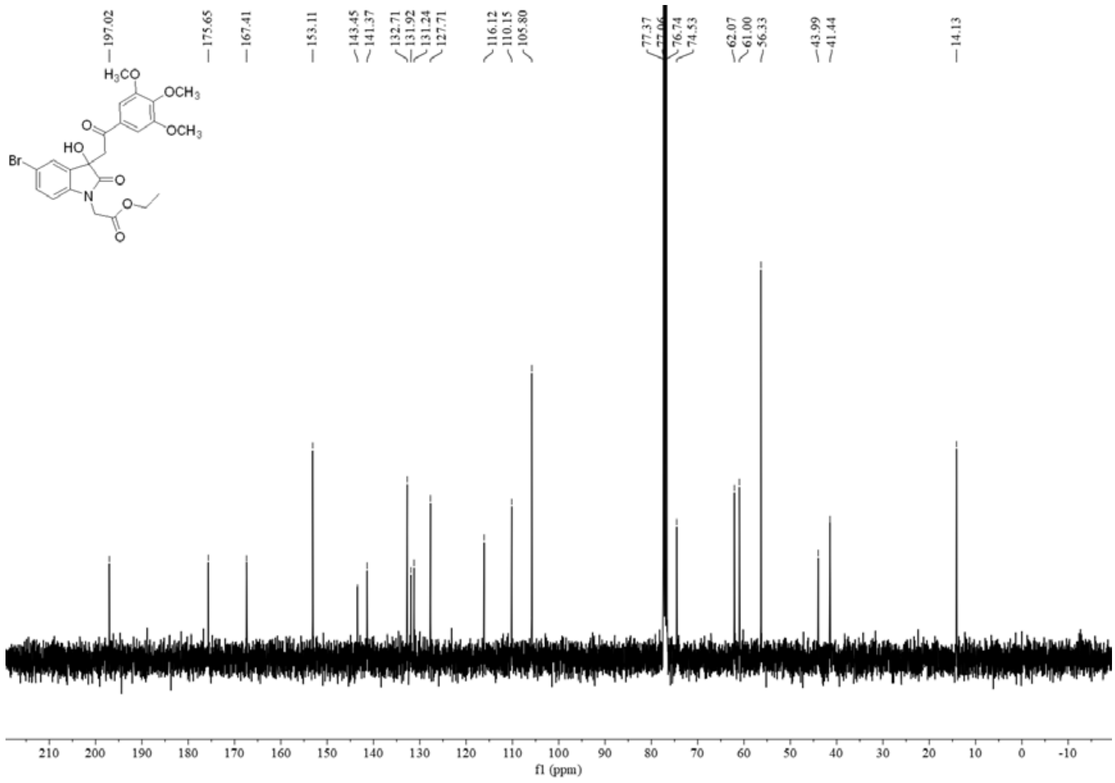

Figure S180. ^{13}C NMR Spectrum of compound **8c**

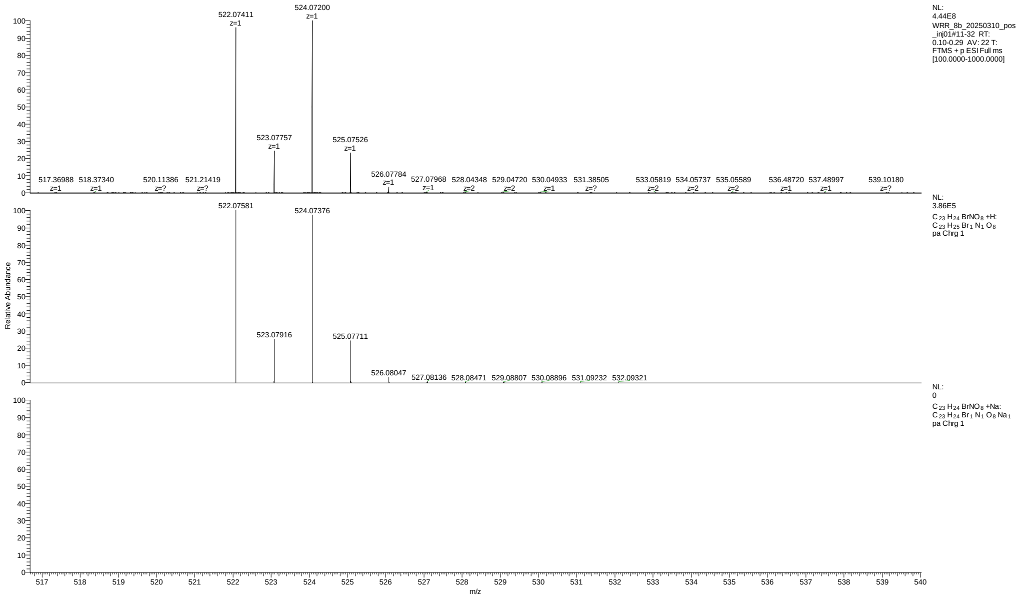

Figure S181. HRMS Spectrum of compound **8c**

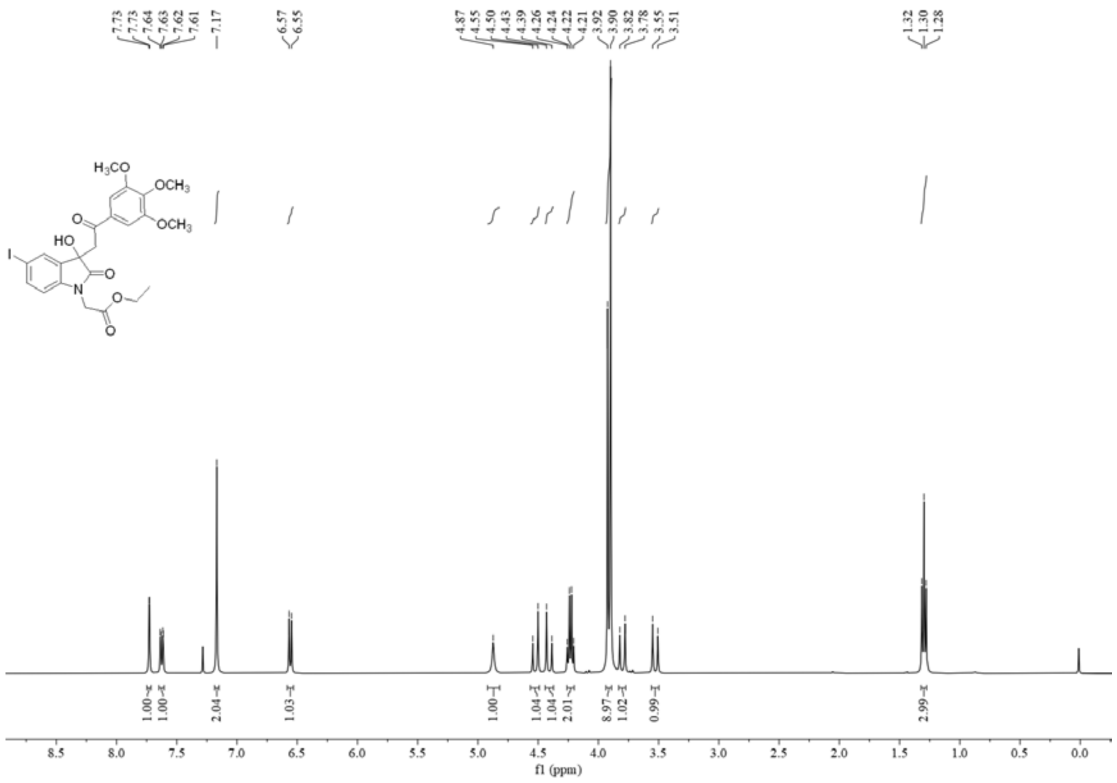

Figure S182. ^{1}H NMR Spectrum of compound **8d**

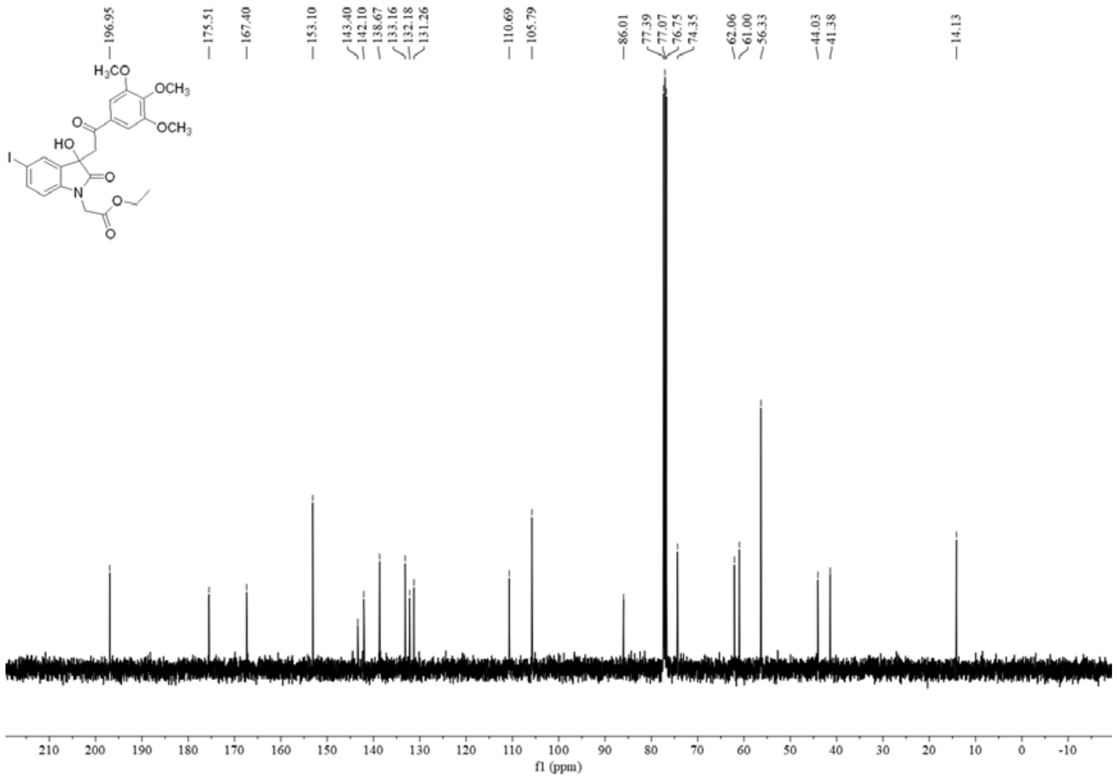

Figure S183. ^{13}C NMR Spectrum of compound **8d**

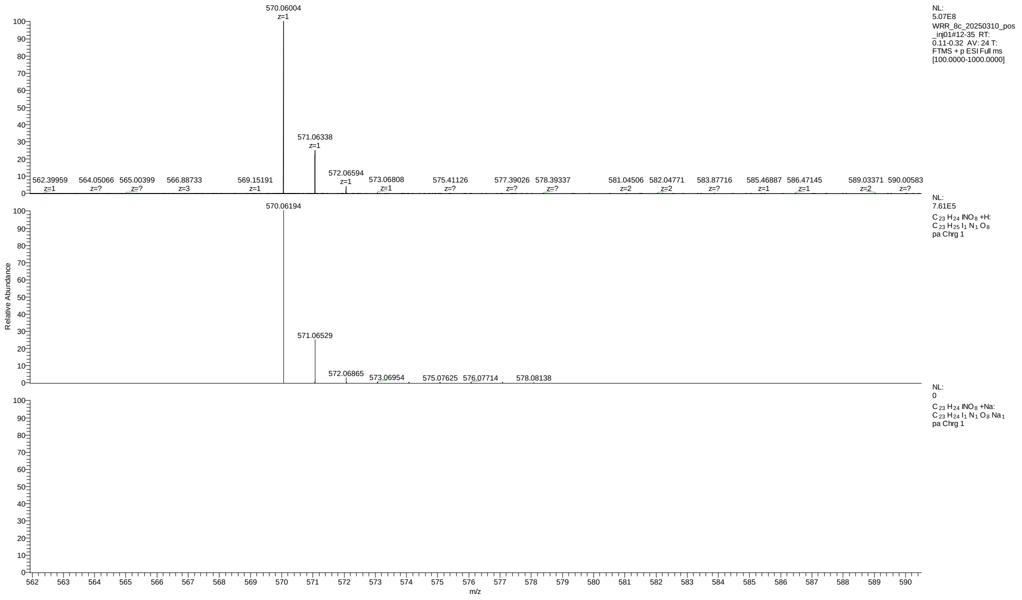

Figure S184. HRMS Spectrum of compound **8d**

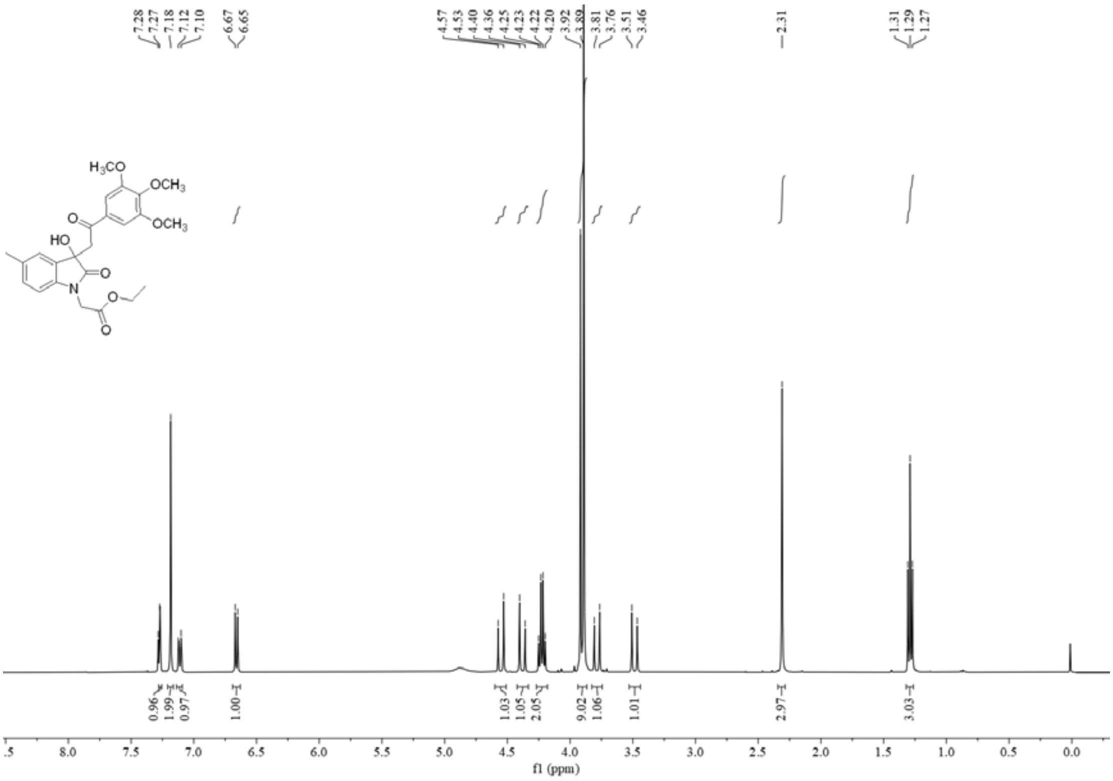

Figure S185. ^{1}H NMR Spectrum of compound **8e**

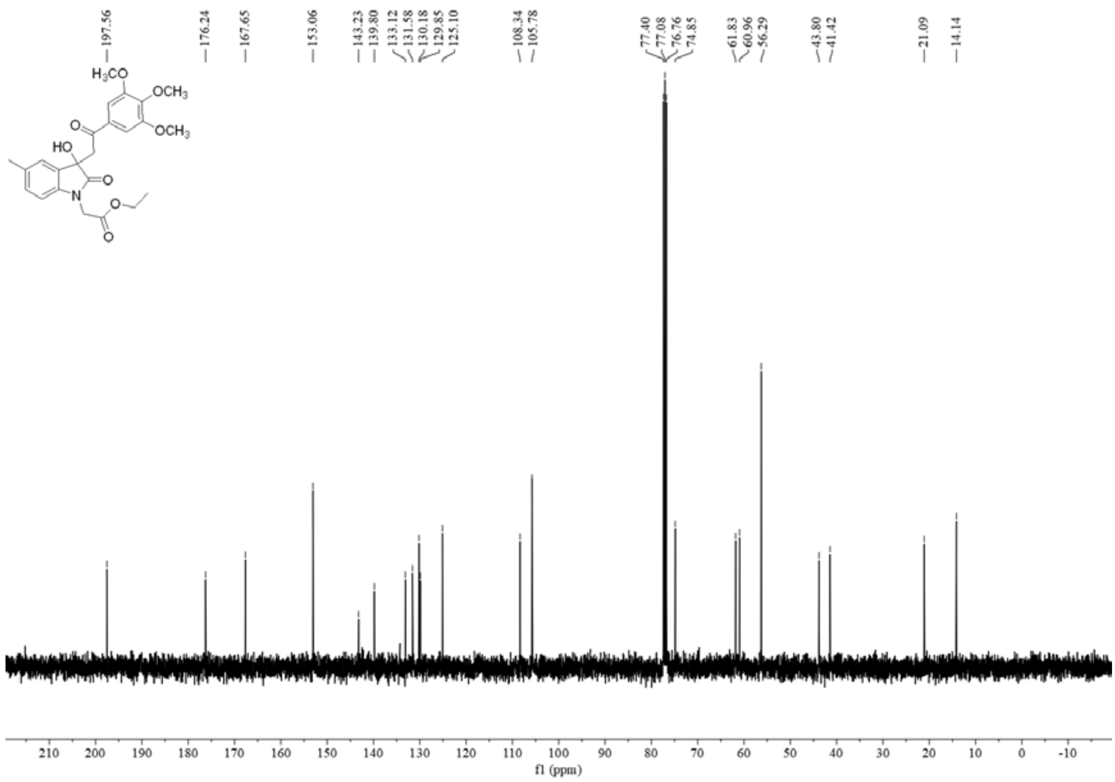

Figure S186. ^{13}C NMR Spectrum of compound **8e**

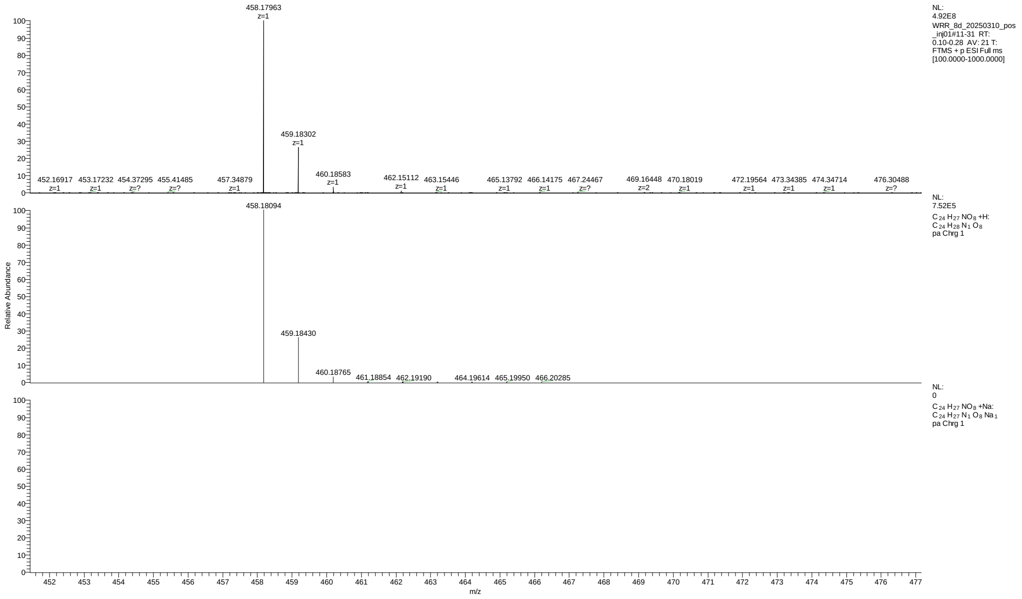

Figure S187. HRMS Spectrum of compound **8e**

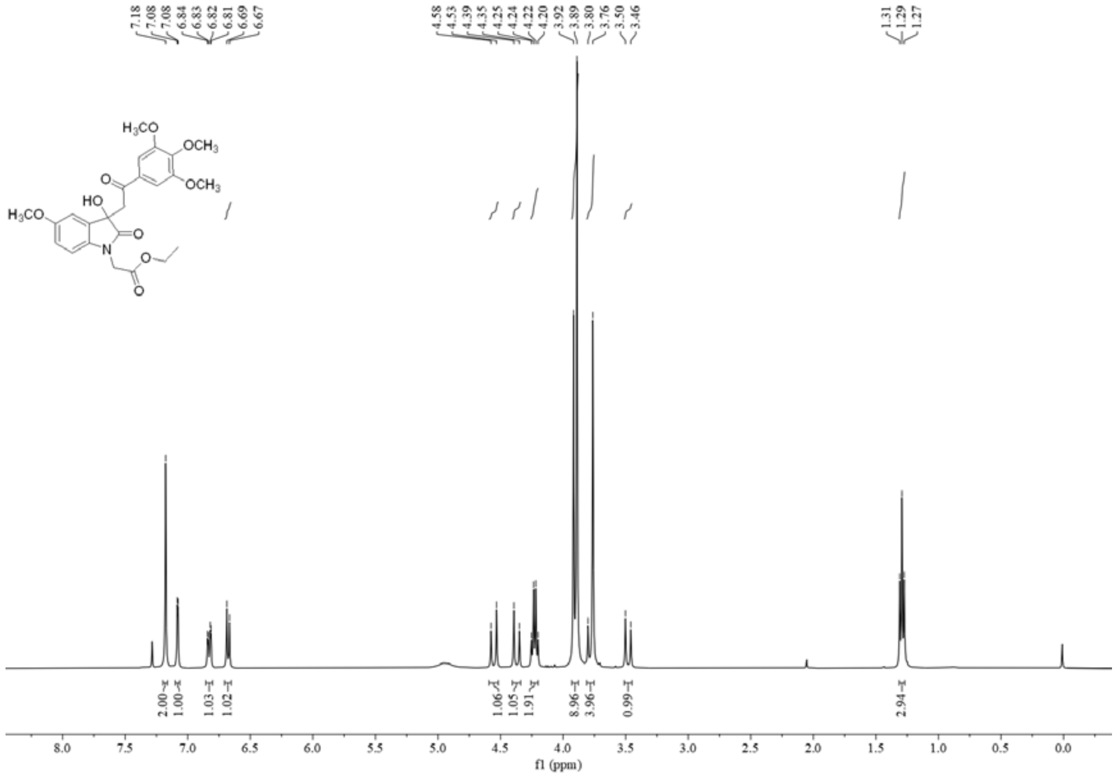

Figure S188. ^{1}H NMR Spectrum of compound **8f**

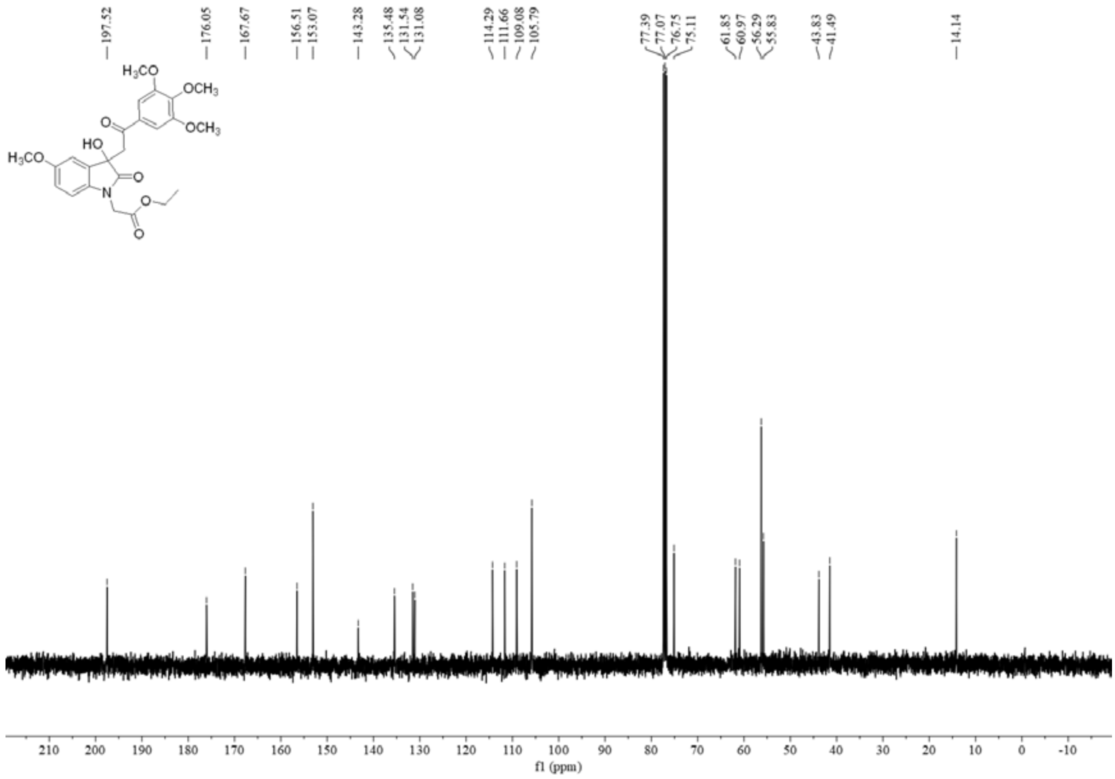

Figure S189. ^{13}C NMR Spectrum of compound **8f**

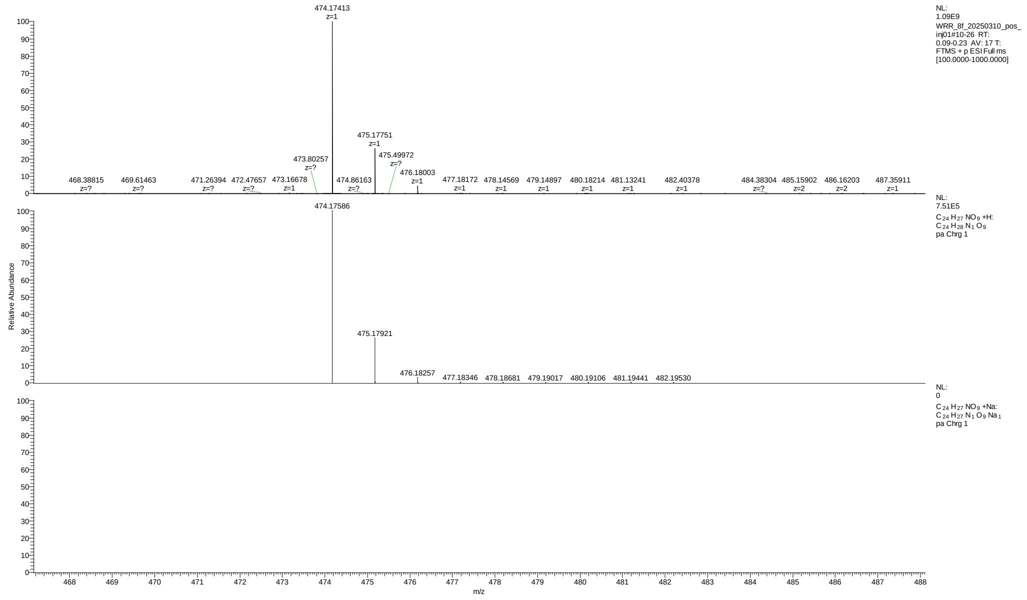

Figure S190. HRMS Spectrum of compound **8f**

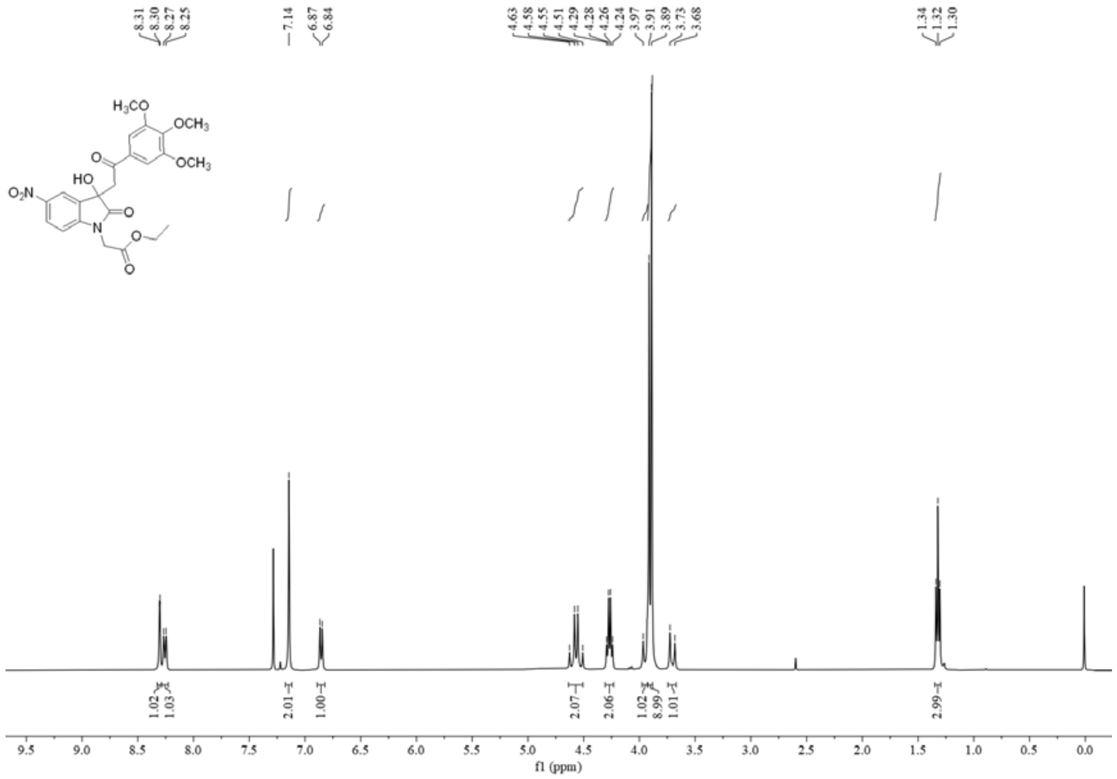

Figure S191. ^{1}H NMR Spectrum of compound **8g**

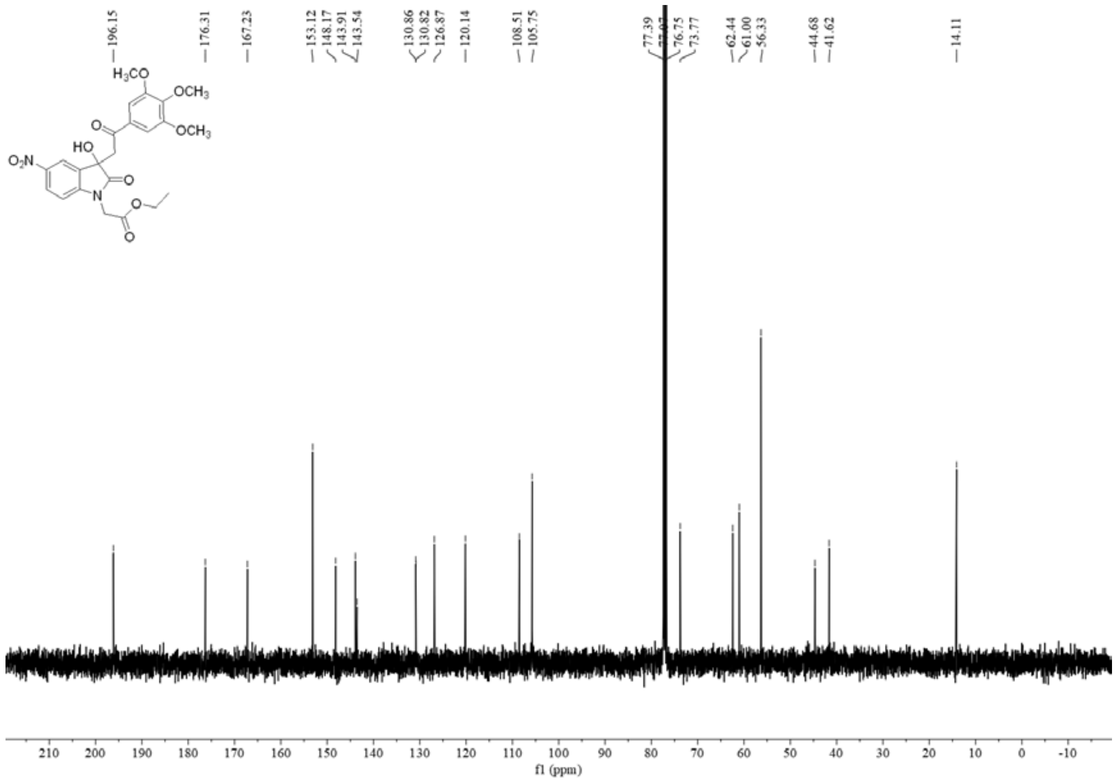

Figure S192. ^{13}C NMR Spectrum of compound **8g**

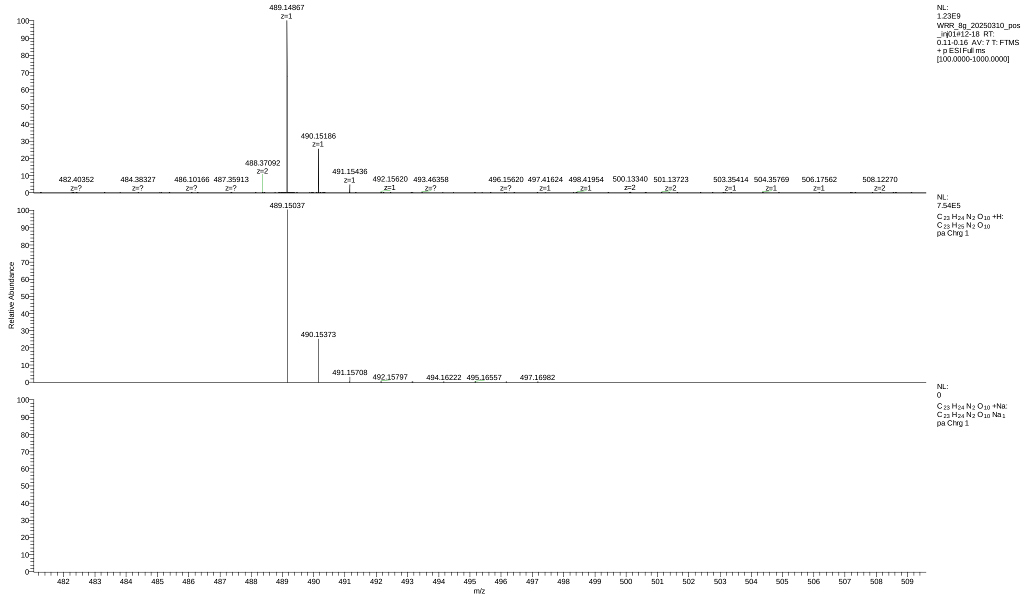

Figure S193. HRMS Spectrum of compound **8g**

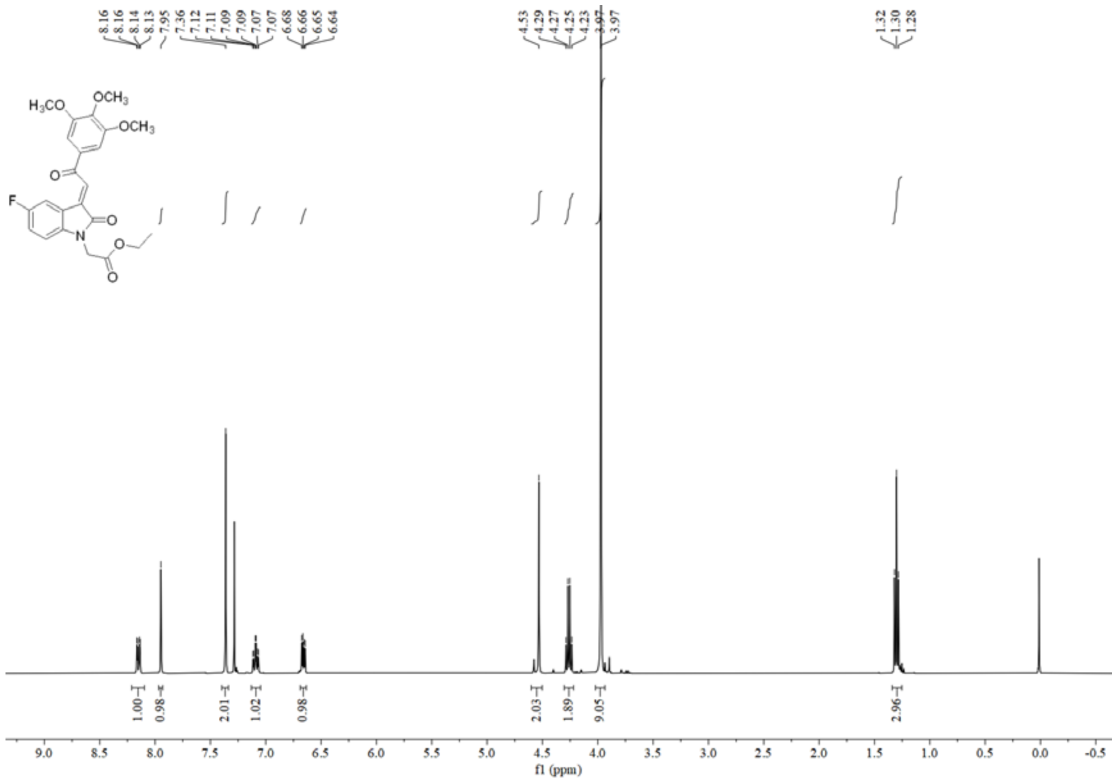

Figure S194. ^{1}H NMR Spectrum of compound **9a**

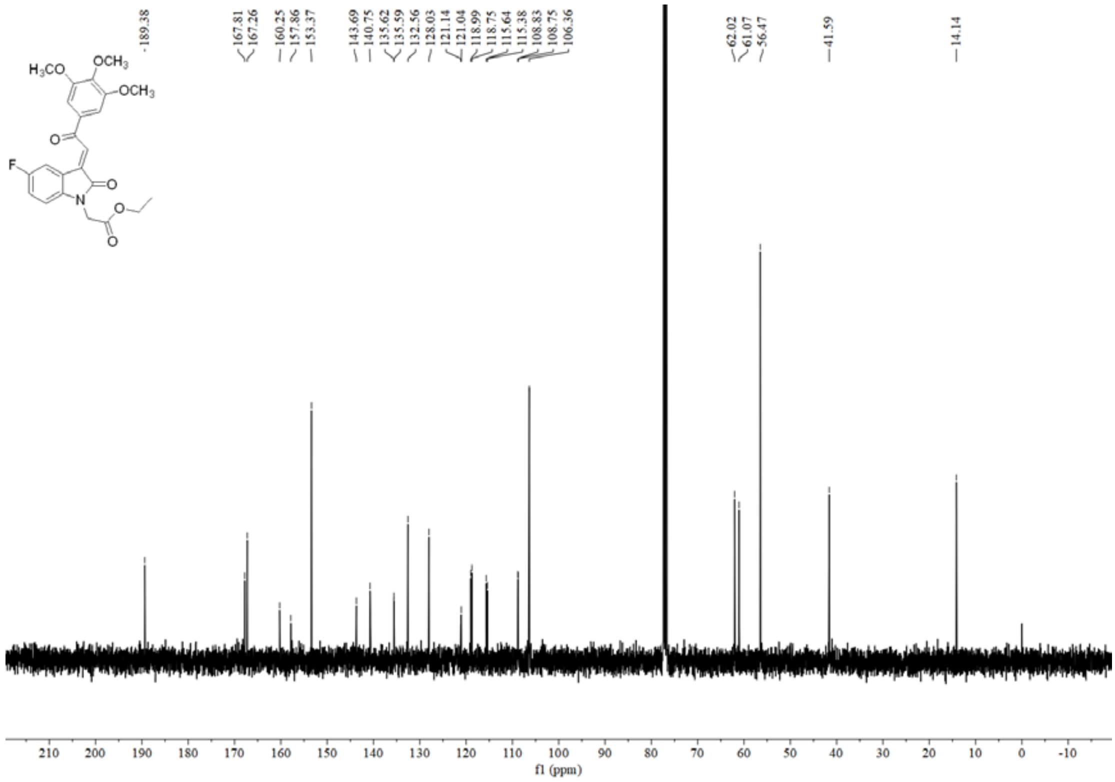

Figure S195. ^{13}C NMR Spectrum of compound **9a**

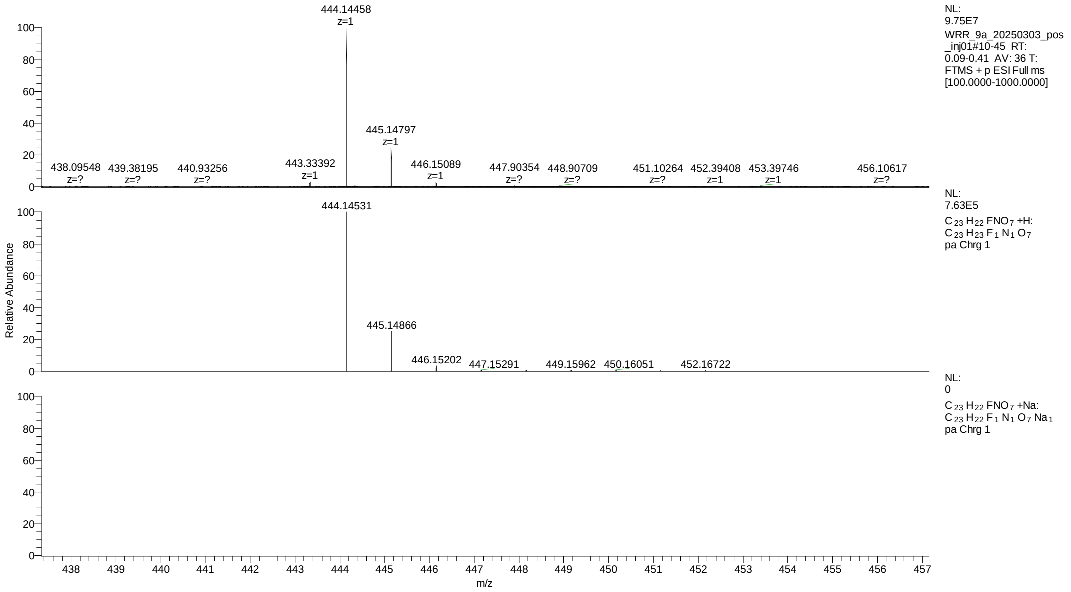

Figure S196. HRMS Spectrum of compound **9a**

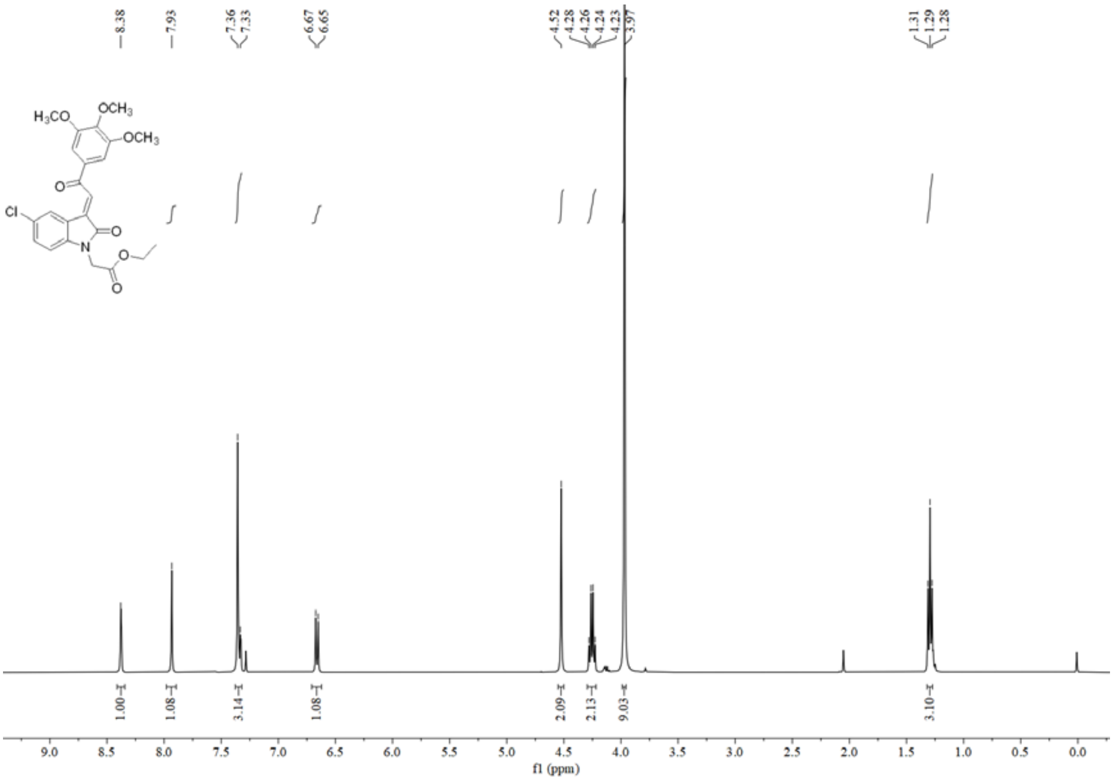

Figure S197. ^{1}H NMR Spectrum of compound **9b**

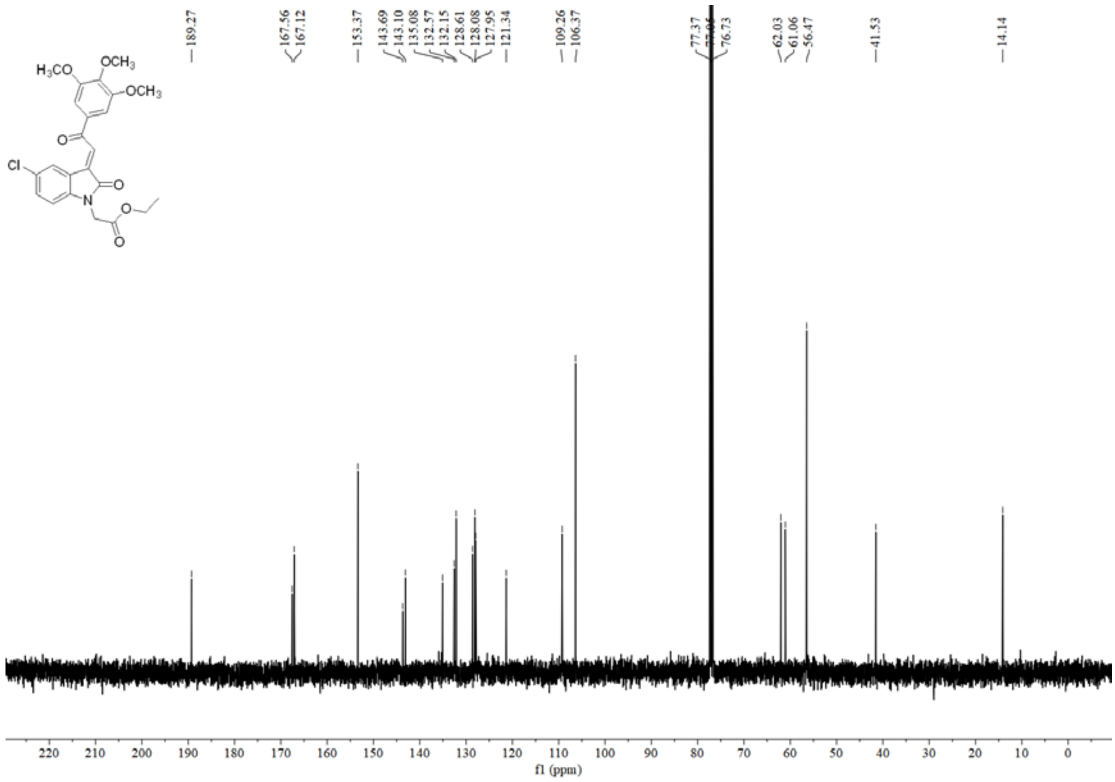

Figure S198. ^{13}C NMR Spectrum of compound **9b**

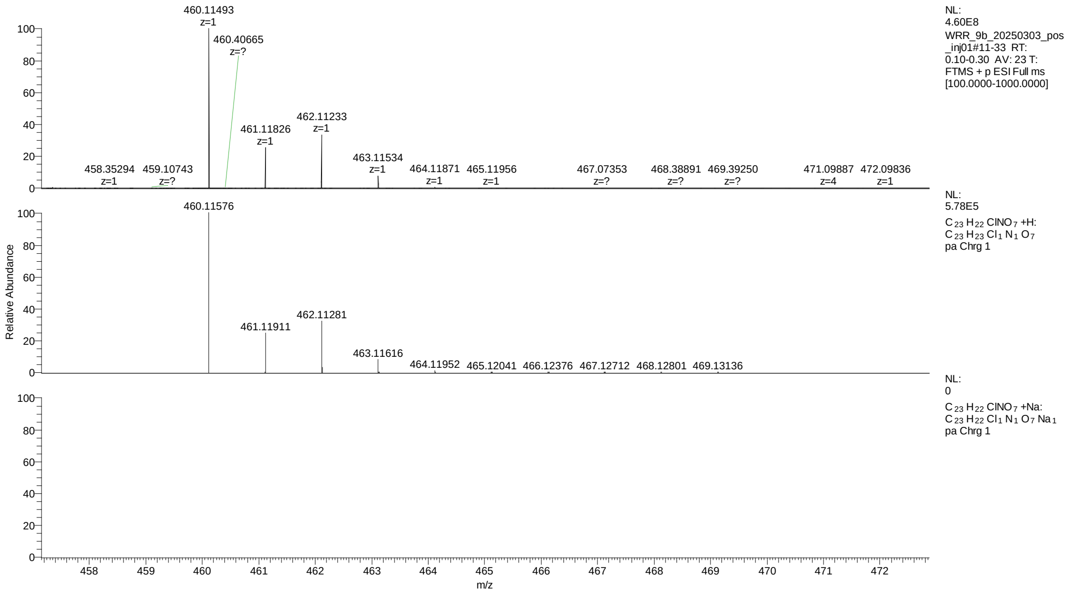

Figure S199. HRMS Spectrum of compound **9b**

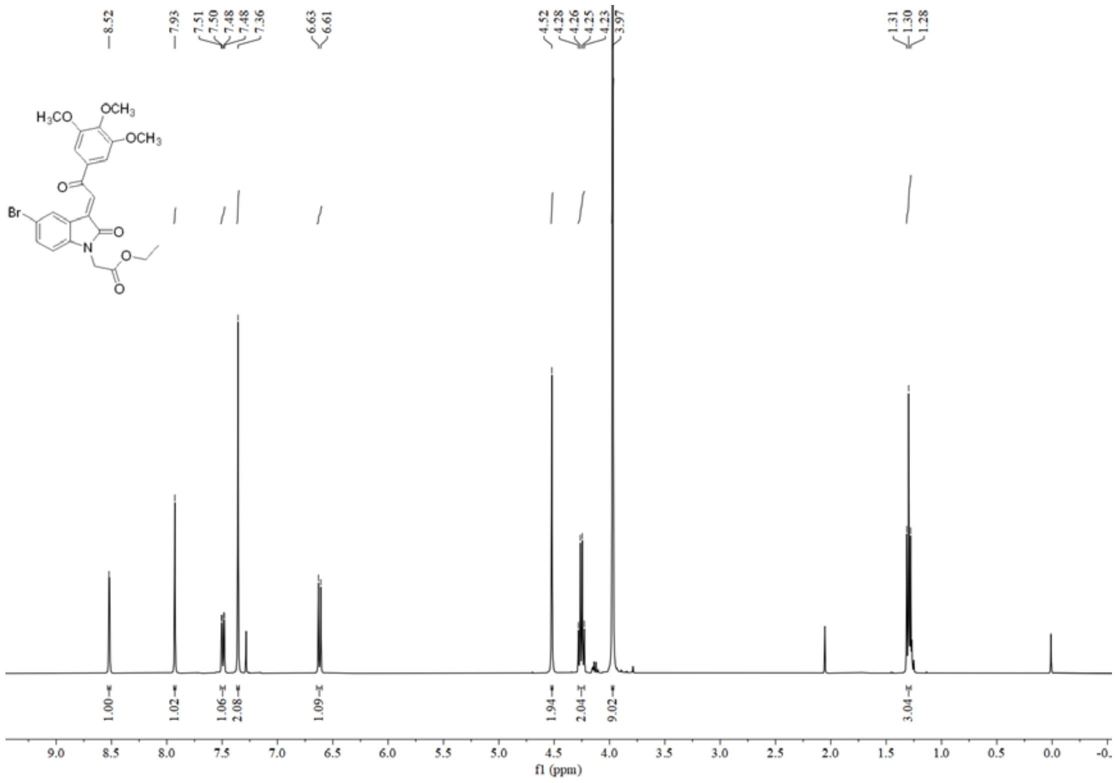

Figure S200. ^{1}H NMR Spectrum of compound **9c**

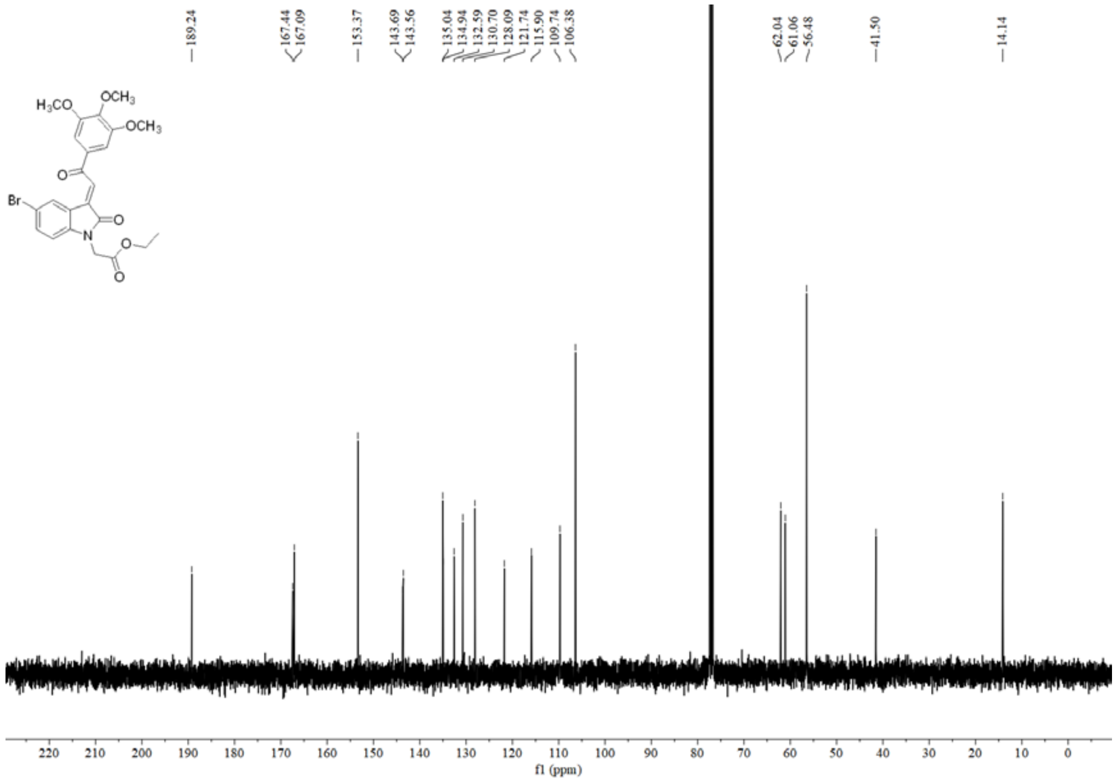

Figure S201. ^{13}C NMR Spectrum of compound **9c**

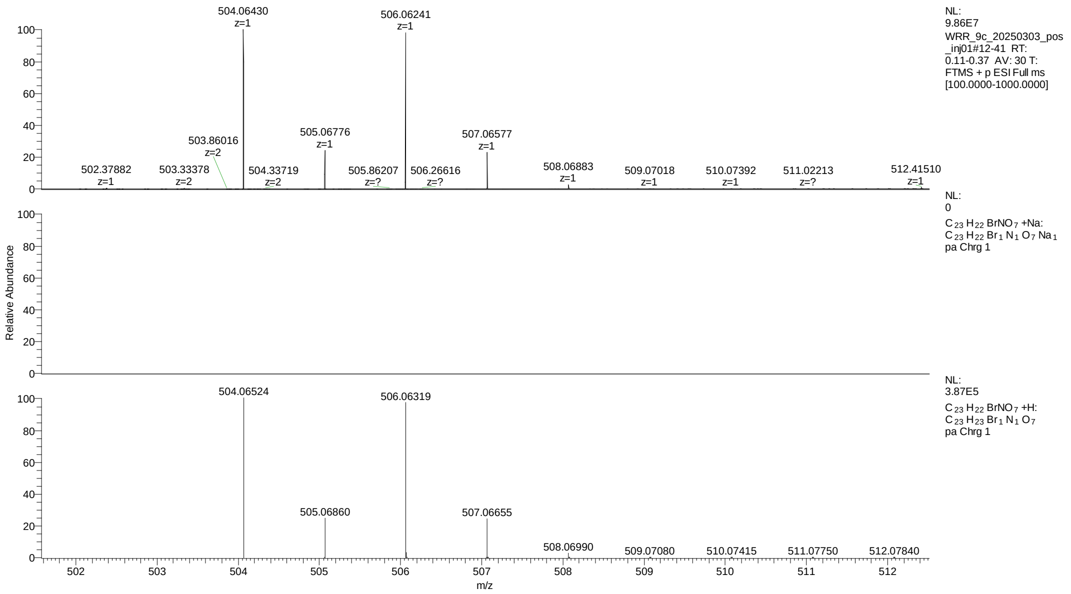

Figure S202. HRMS Spectrum of compound **9c**

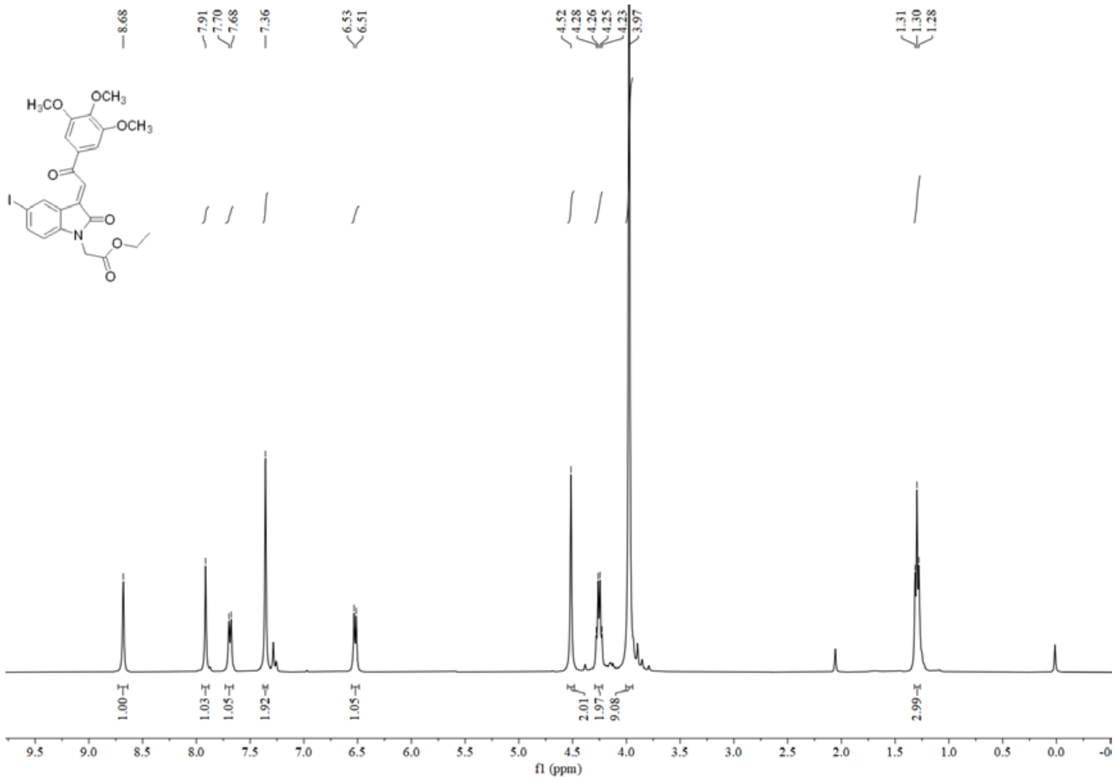

Figure S203. ^{1}H NMR Spectrum of compound **9d**

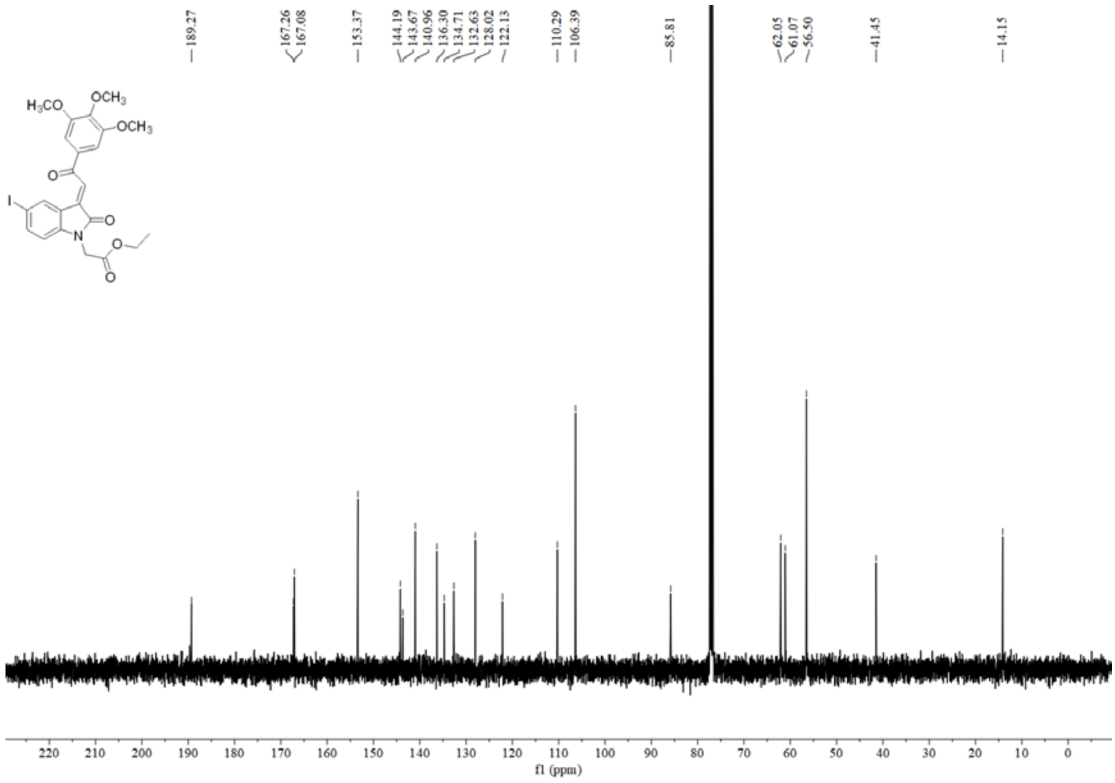

Figure S204. ^{13}C NMR Spectrum of compound **9d**

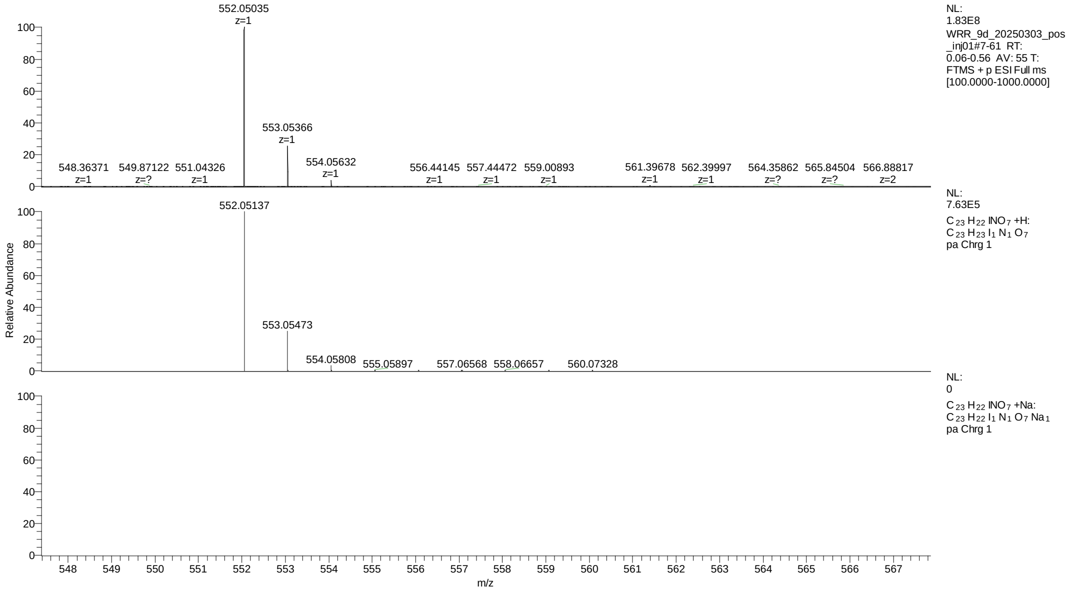

Figure S205. HRMS Spectrum of compound **9d**

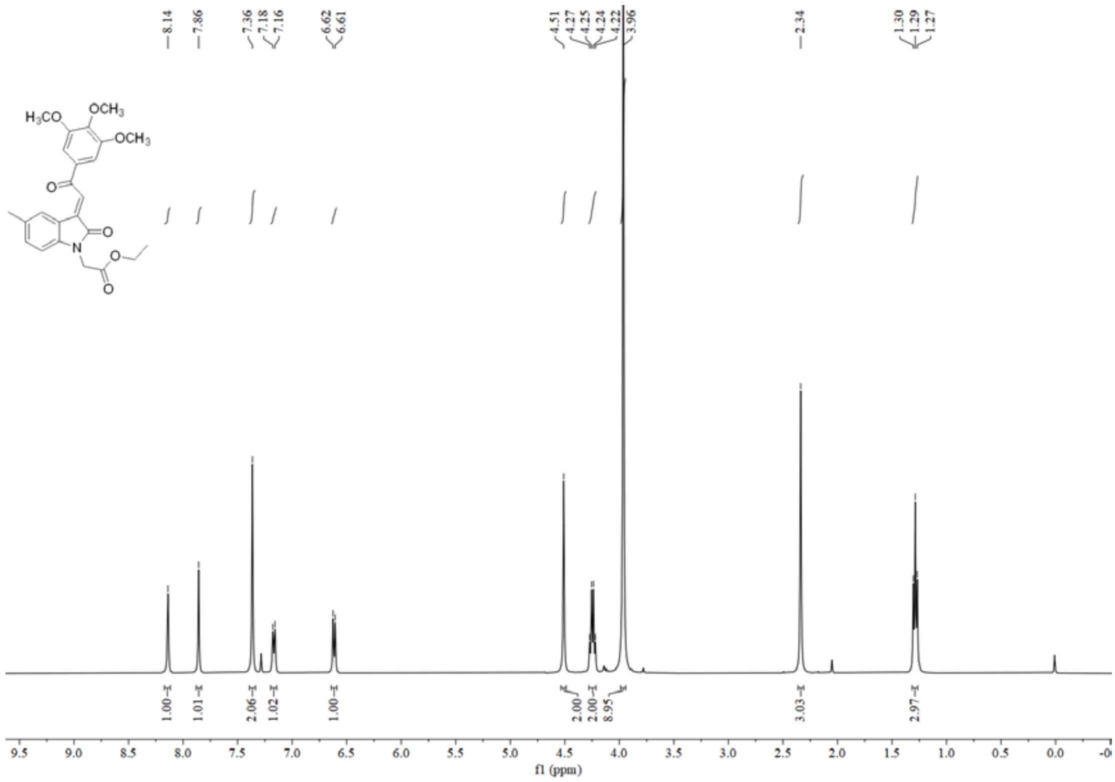

Figure S206. ^{1}H NMR Spectrum of compound **9e**

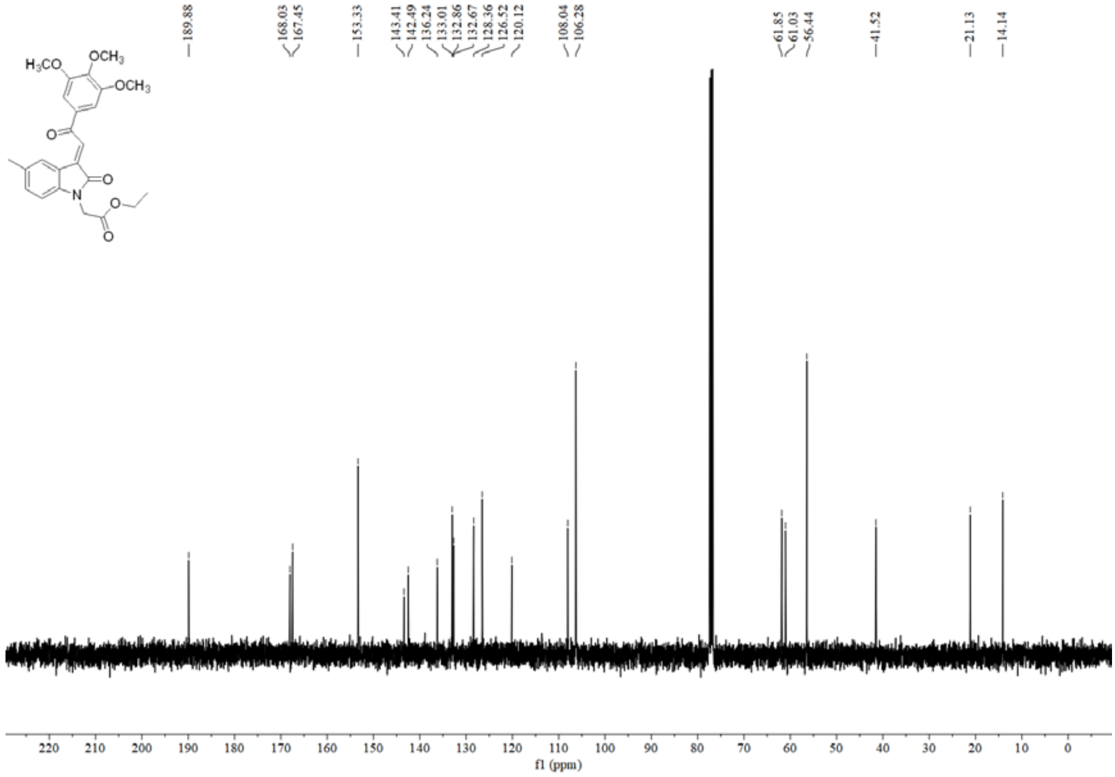

Figure S207. ^{13}C NMR Spectrum of compound **9e**

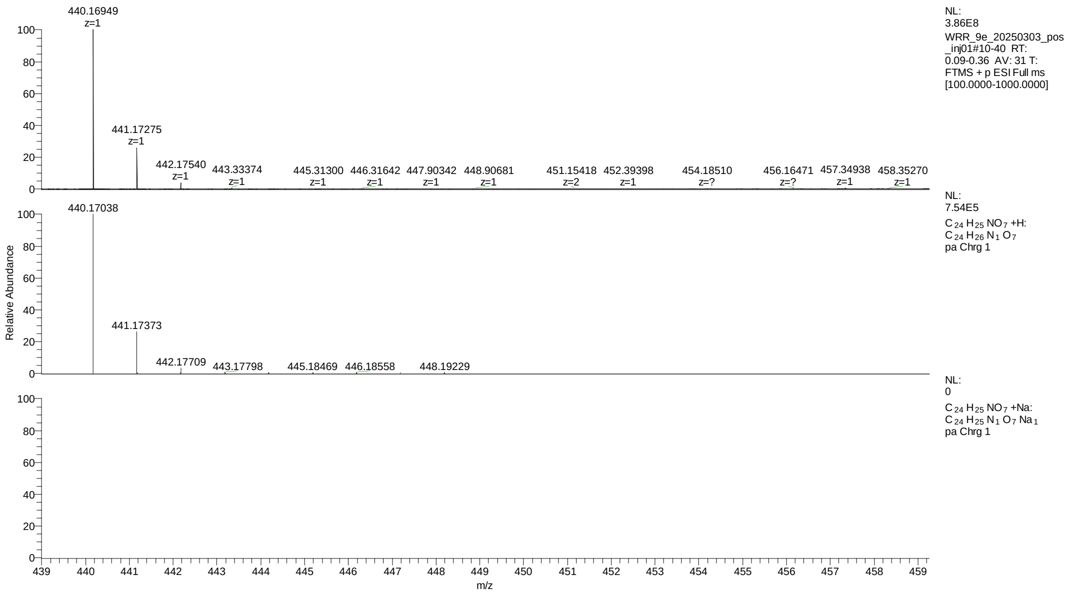

Figure S208. HRMS Spectrum of compound **9e**

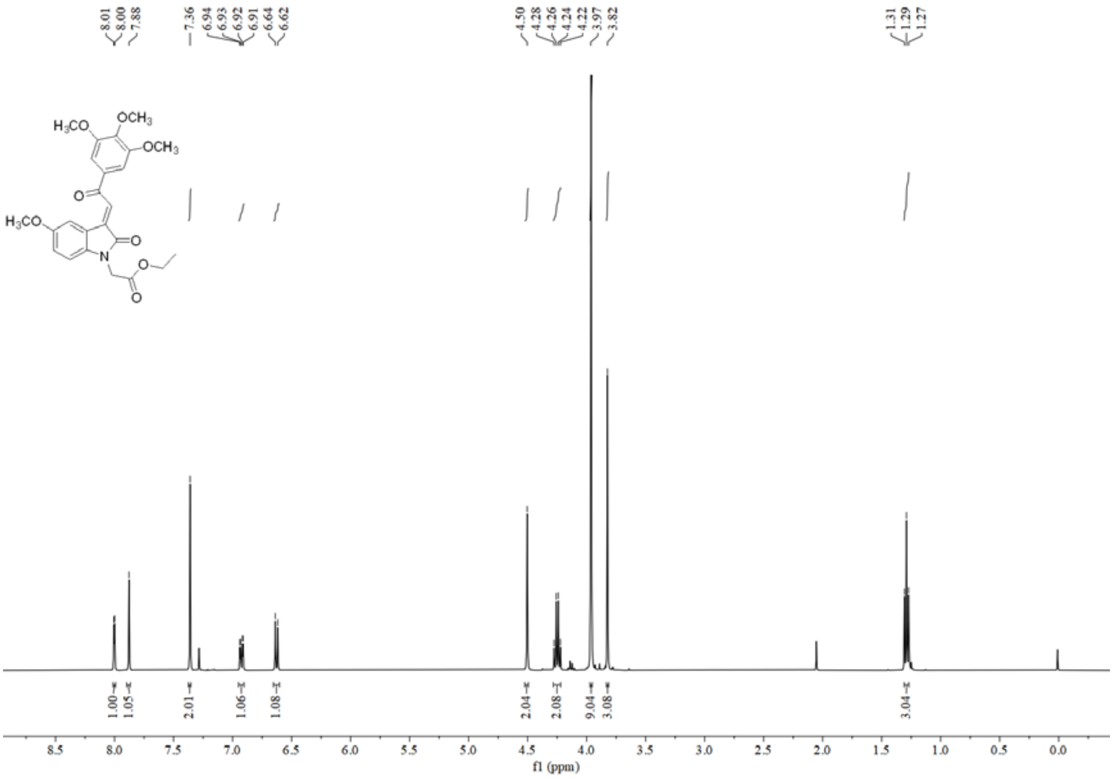

Figure S209. ^{1}H NMR Spectrum of compound **9f**

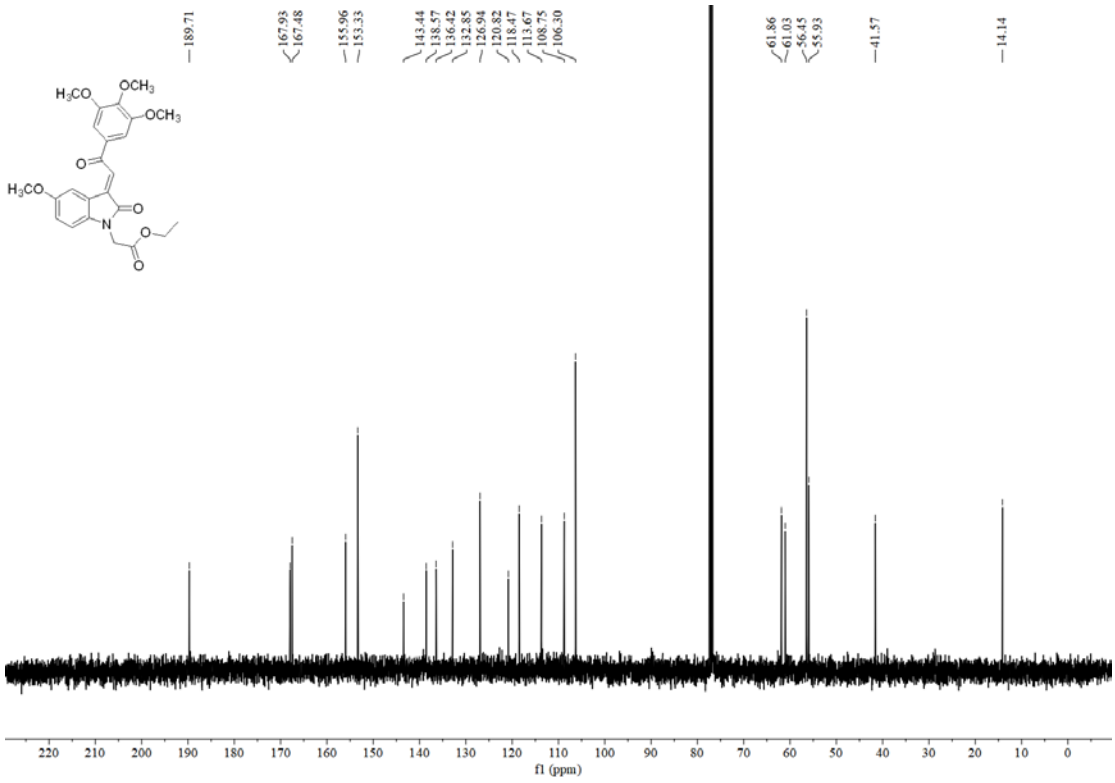

Figure S210. ^{13}C NMR Spectrum of compound **9f**

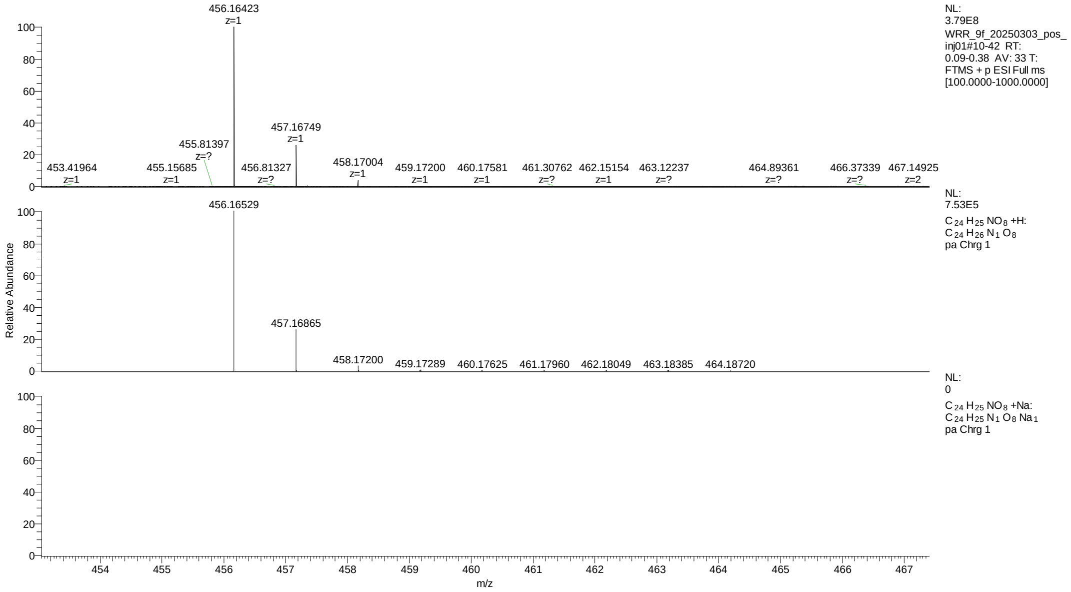

Figure S211. HRMS Spectrum of compound **9f**

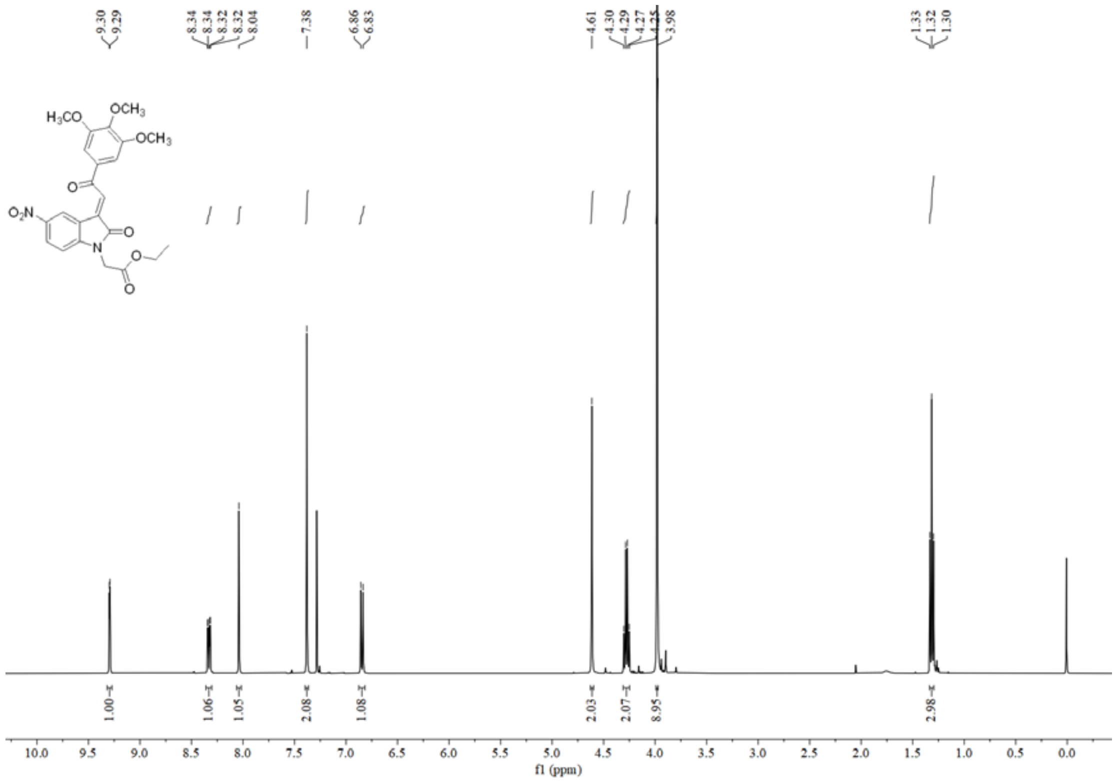

Figure S212. ^{1}H NMR Spectrum of compound **9g**

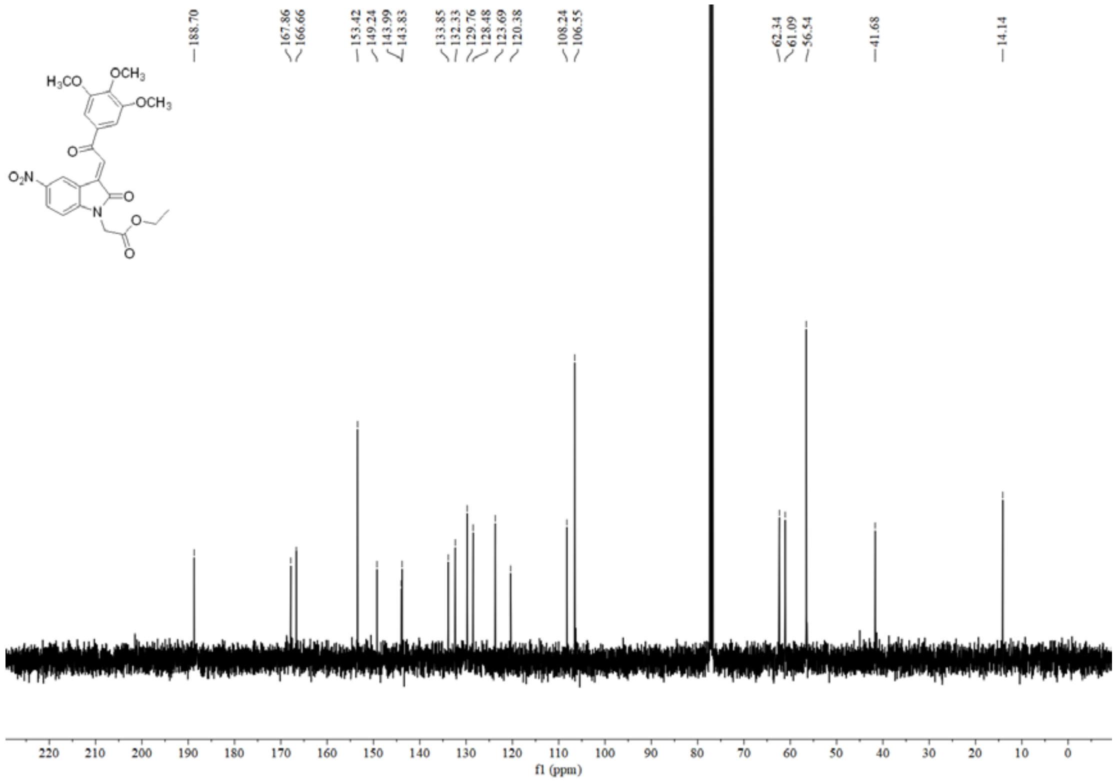

Figure S213. ^{13}C NMR Spectrum of compound **9g**

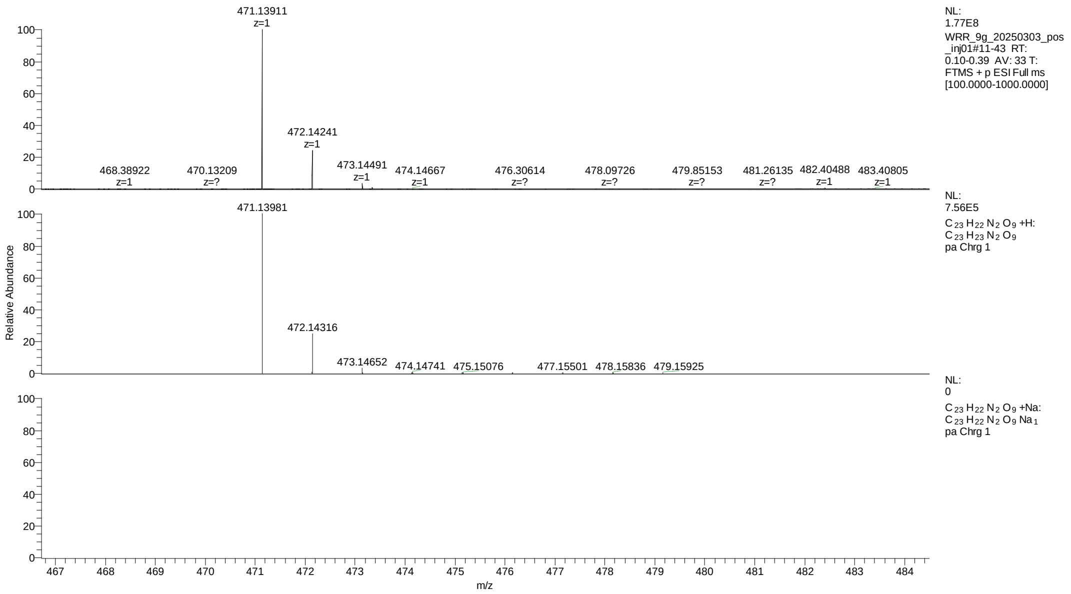

Figure S214. HRMS Spectrum of compound **9g**
